# Enantioselective [1,3] O-to-C rearrangement: dearomatization of alkyl 2-allyloxy/benzyloxy-1/3-naphthoates catalyzed by a chiral π–Cu(ii) complex†
†Electronic supplementary information (ESI) available. CCDC 1865509 and 1865510. For ESI and crystallographic data in CIF or other electronic format see DOI: 10.1039/c8sc05601c


**DOI:** 10.1039/c8sc05601c

**Published:** 2019-01-10

**Authors:** Lu Yao, Kazuaki Ishihara

**Affiliations:** a Graduate School of Engineering , Nagoya University , B2-3(611) Furo-cho, Chikusa , Nagoya 464-8603 , Japan . Email: ishihara@cc.nagoya-u.ac.jp

## Abstract

An unprecedented catalytic asymmetric [1,3] O-to-C rearrangement of alkyl 2-allyloxy/benzyloxy-1/3-naphthoates was realized under the catalysis of a chiral π–Cu(ii) complex (1–10 mol%). This dearomatization strategy provides facile access to highly functionalized β-naphthalenone derivatives bearing an all-carbon quaternary stereogenic center in high yield with excellent enantioselectivity. The π–cation interaction between the aromatic substituent of the ligand and the Cu(ii) center was proved by X-ray diffraction analysis and shown to be crucial for enantioselective control. Further preliminary mechanistic studies suggest that this intramolecular reaction proceeds through a contact ion pair intermediate.

## Abstract 2

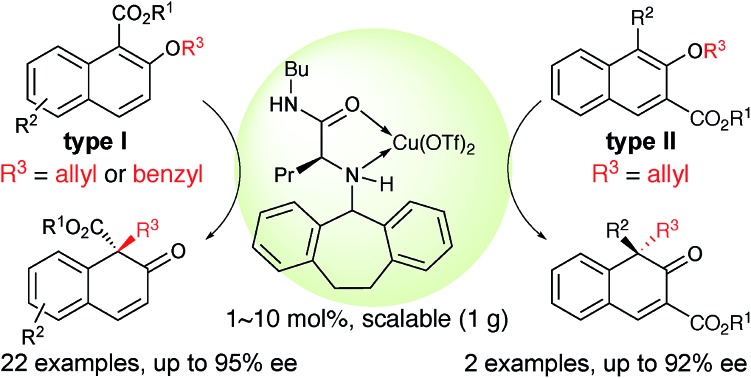
An asymmetric [1,3] O-to-C rearrangement of alkyl 2-allyloxy/benzyloxy-1/3-naphthoates was realized under the catalysis of a chiral π–Cu(ii) complex to produce naphthalenone derivatives bearing an all-carbon quaternary stereogenic center in good to high yield with excellent enantioselectivity. The π–cation interaction of the complex was proved by X-ray diffraction analysis.

## 1. Introduction

O-to-C rearrangements are among the most powerful C–C bond forming strategies in organic synthesis. One such well-known reaction is Claisen rearrangement,^1^ which has been shown to transfer stereochemistry from a cleaved C–O bond to a formed C–C bond *via* a concerted [3,3] sigmatropic pathway. In contrast, non-concerted [*m*,*n*] rearrangement such as [1,3] rearrangement has gained less attention. This lack of research is probably due to high activation barriers, since suprafacial [1,3]-sigmatropic rearrangement is an orbital symmetry-forbidden process, and typically requires harsh reaction conditions such as high temperature to proceed through radical intermediate.^2^ To overcome this limitation, tremendous effort has been devoted to the activation of a substrate by various catalytic systems (transition metal catalysis,^3^ nucleophilic catalysis^4^ and Lewis acid catalysis^5^). Among these systems, Lewis acid catalysis has been most intensively studied. Mechanistic studies have shown that, in general, the Lewis acid-promoted reaction proceeds with heterolytic cleavage of the O–C bond of the vinyl ether, in which the ion pair intermediates (the carbocation and the enolate counter anion) would be generated, and the recombination of the resulting intermediates would lead to the formation of the desired product. This mechanism suggests that an appropriate, well-designed substrate that can generate a stable ion pair, along with a carefully selected Lewis acid, might lead to successful [1,3] O-to-C rearrangement. Although significant effort has been made in this area, there is still room for the improvement of the current method. To the best of our knowledge, catalytic enantioselective [1,3] rearrangement has remained elusive to date.^4^

**Scheme 1:**
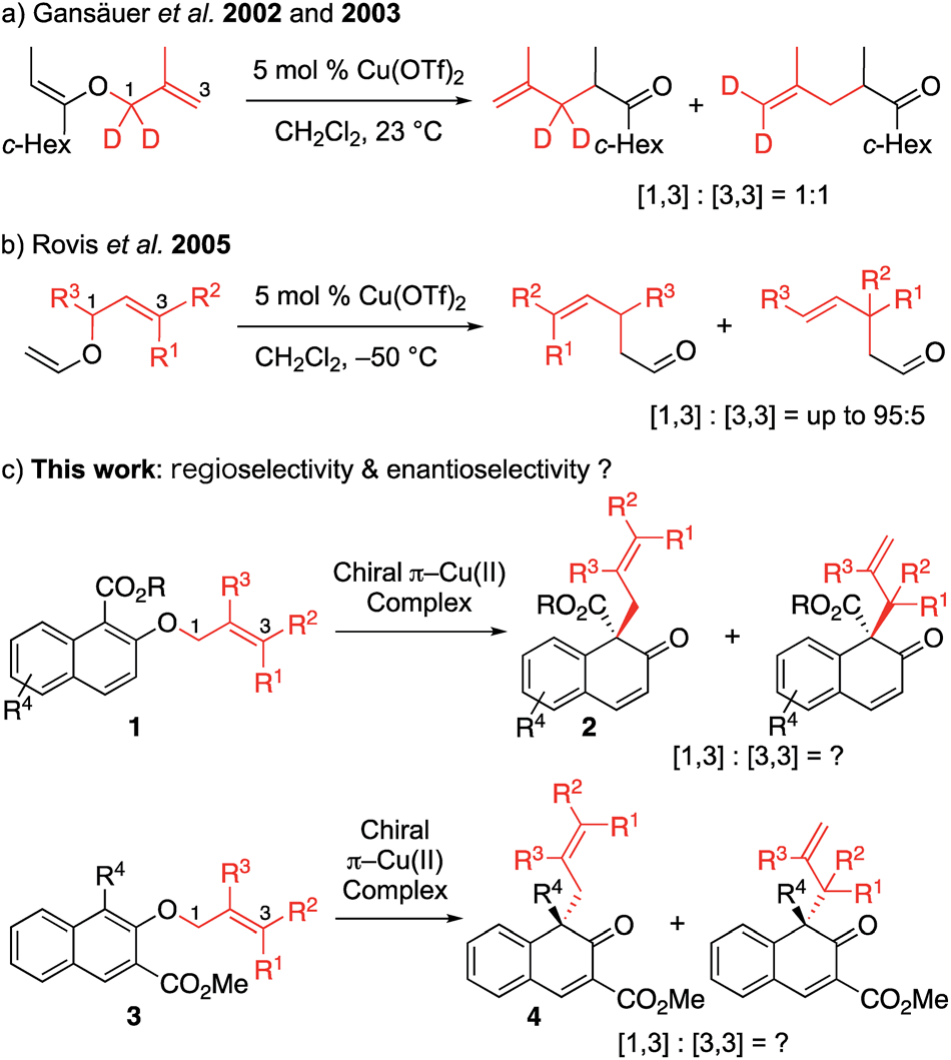
Cu(ii)-catalyzed [1,3] rearrangement reactions of allyl vinyl ethers or allyl naphthyl ether: (a) Gansäuer *et al.* 2002 and 2003. (b) Rovis *et al.* 2005. (c) This work: regioselectivity & enantioselectivity?

Our group recently developed a group of small-molecule Cu(ii) catalysts based on intramolecular π–cation interactions and has demonstrated their efficiency in several enantioselective cycloaddition reactions of α,β-unsaturated carboxamides.^6^ As part of our ongoing interest in extending the application of these chiral π–Cu(ii) catalysts, we started to consider its capability in the catalysis of [1,3] rearrangement reactions. In 2002 and 2003, Gansäuer demonstrated that, in the presence of catalytic amount of Cu(OTf)_2_, the vinyl ether could lead to [1,3] and [3,3] products in 1 : 1 ratio (Scheme 1a).^7^ In the year 2005, Rovis reported the regioselective [1,3] rearrangement of allyl vinyl ether catalyzed by aluminum and copper Lewis acids.^8^ They mentioned that the rearrangement of trisubstituted alkenes would preferentially produce the [1,3] adduct due to steric congestion for normal [3,3] rearrangement. Thus, as shown in Scheme 1b, the [1,3] product could be obtained in moderate to good yields with up to 95 : 5 regioselectivity. Inspired by these pioneering studies, we envisioned that catalytic enantioselective [1,3] O-to-C rearrangement might be realized with suitable functionalized substrates by using our catalytic system. Here we report the first enantioselective [1,3] O-to-C rearrangement reaction of alkyl 2-allyloxy/benzyloxy-1-naphthoates **1** and methyl 1-substituted 2-allyloxy-3-naphthoates **3** catalyzed by the chiral π–Cu(ii) complex (Scheme 1c).

## 2. Results and discussion

First, we sought to identify appropriate substrates. Considering that naphthols are readily available starting materials that can be used to access functionalized chiral naphthalenones, we decided to investigate the feasibility of naphthol-derived vinyl ethers as potential substrates.^9^ Recently, You^10^ and Zhong^11^ reported the catalytic asymmetric allylic dearomatization (CADA) of naphthol derivatives.^12^ We anticipate that the same product would be generated if allyl naphthyl ethers undergo [1,3] O-to-C rearrangement under catalysis by the Cu(ii) complex (Scheme 1c). If this works, our strategy could avoid the need to use precious metals such as palladium or iridium.

After a brief screening of substituents on both naphthalene rings and the allylic chain, we were very pleased to find that **1a** could undergo [1,3] O-to-C rearrangement smoothly under catalysis of Cu(OTf)_2_, affording the dearomatization product **2a** with an all-carbon quaternary stereogenic center in low yield. However, no [3,3] rearrangement product was observed during the process.^13^ This result encouraged us to perform further ligand screening.^14^ Initial optimization showed that the *N*-dibenzosuberyl substituted group is crucial for enantioselective control, which is consistent with our original intention to introduce π–cation interaction into the catalyst design. Hence, we decided to keep this moiety intact while evaluating other substituted groups on the chiral ligand (Table 1). The R^1^ group that originated from l-amino acids had noticeable effects on the enantioselectivity; the results of **L1** to **L5** indicate that the steric hindrance of this substituent should neither be too large nor too small (entries 1–5). l-Norvaline-derived ligand **L4** (R^1^ = propyl) gave the desired product **2a** in 85% NMR yield with 89% ee (entry 4). The amide moiety of the ligand was also examined. The structure of the amide moiety strongly influenced the reaction rate. For example, tertiary amide- and bulky secondary amide-substituted ligands, such as **L4**, **L9** and **L10**, were not so effective for promoting the reaction (entries 4, 9 and 10). In comparison, the reaction was complete within only 6 hours when less-bulky secondary amide-substituted ligands, such as **L6–L8**, were used at – 20 °C (entries 6–8). Finally, we found that the desired [1,3] O-to-C rearrangement product **2a** could be obtained in 90% isolated yield with excellent enantioselectivity (91% ee) at –30 °C using 10 mol% l-norvaline-derived chiral π–Cu(ii) complex as a catalyst in dichloromethane^15^ (entry 11).

**Table 1:** Ligand optimization^*a*^

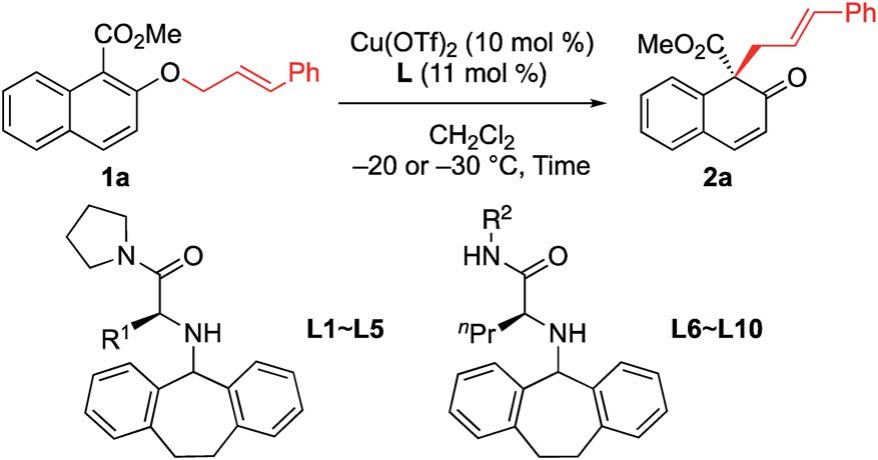					
Entry	Ligand	Time (h)	**2a**		
			Conv.^*b*^ (%)	Yield^*b*^ (%)	Ee^*c*^ (%)
1	**L1** (R^1^ = Me)	14	96	74	73
2	**L2** (R^1^ = Et)	14	96	77	86
3	**L3** (R^1^ = *i*Pr)	14	72	55	77
4	**L4** (R^1^ = Pr)	14	94	85	89
5	**L5** (R^1^ = Bu)	14	93	72	83
6	**L6** (R^2^ = Et)	6	99	83	85
7	**L7** (R^2^ = Pr)	6	98	80	90
8	**L8** (R^2^ = Bu)	6	99	82	90
9	**L9** (R^2^ = CH_2_*t*Bu)	12	98	80	90
10	**L10** (R^2^ = *i*Pr)	60	86	68	78
11^*e*^	**L8** (R^2^ = Bu)	24	99	90^*d*^	91

^*a*^Unless otherwise noted, all reactions were carried out with 0.15 mmol of **1a** in dichloromethane (0.75 mL) at –20 °C.

^*b*^Yield of **2a** based on ^1^H NMR. Small amount of methyl 3-cinnamyl-2-hydroxy-naphthoate (**2a′**) was included in crude products.

^*c*^Determined by HPLC analysis.

^*d*^Isolated yield of **2a**.

^*e*^The reaction was carried out at –40 °C.

With the optimized conditions in hand, we started to evaluate the generality of this [1,3] rearrangement reaction. First, we assessed the substituent effect on the allyl moiety. As revealed in Table 2, cinnamyl groups bearing electron-rich (entries 2–4), electron-neutral (entry 1), and electron-deficient (entries 5 and 6) substituents were all well-accommodated and gave the corresponding products **2** in good to high yield with excellent enantioselectivity. Additionally, naphthyl-substituted substrates were also tolerated in this reaction, and the desired products were obtained in excellent yield and with high enantioselectivity (entries 7 and 8). We were very pleased to find that the rearrangement of heteroaryl-substituted substrates was also successful, and gave the corresponding β-naphthalenones in moderate to good yield with high ee values (entries 9 and 10). Disubstituted substrates were compatible with this rearrangement protocol. The desired products were obtained in high yield with high enantioselectivity when **1k** and **1l** were used in this reaction (entries 11 and 12), while prenyl naphthyl ether (**1m**) gave the desired product **2m** in only 58% yield with 68% ee (entry 13). These results indicate that at least one aryl group is needed to achieve both high yield and high enantioselectivity.

**Table 2:** Substrate scope^*a*^

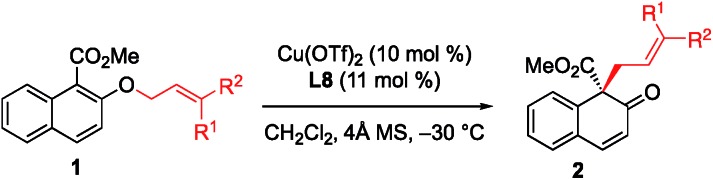					
Entry	**1** (R^1^, R^2^)	Time (h)	**2**		
				Yield^*b*^ (%)	Ee^*c*^ (%)
1	**1a** (H, Ph)	24	**2a**	90	91
2	**1b** (H, *o*-MeC_6_H_4_)	16	**2b**	90	95
3	**1c** (H, *m*-MeC_6_H_4_)	20	**2c**	91	92
4	**1d** (H, *p*-MeC_6_H_4_)	10	**2d**	87	90
5	**1e** (H, *p*-BrC_6_H_4_)	60	**2e**	76	91
6	**1f** (H, *p*-ClC_6_H_4_)	60	**2f**	78	92
7	**1g** (H, 1-naphthyl)	10	**2g**	95	92
8	**1h** (H, 2-naphthyl)	10	**2h**	86	92
9^*d*^	**1i** (H, 3-furyl)	14	**2i**	63	96
10	**1j** (H, 3-thienyl)	1	**2j**	85	94
11^*e*^	**1k** (Me, Ph)	10	**2k**	92	94
12^*e*^	**1l** (R^1^ = R^2^: *p*-ClC_6_H_4_)	36	**2l**	89	90
13	**1m** (Me, Me)	24	**2m**	58	68

^*a*^All reactions were carried out with 0.15 mmol of **1** in dichloromethane (0.75 mL) at –30 °C.

^*b*^Isolated yield.

^*c*^Determined by HPLC analysis.

^*d*^The reaction was carried out at –60 °C.

^*e*^The reaction was carried out at –40 °C.

The substituent effect on the naphthalene ring was also investigated (Table 3). Various substituents were well-tolerated at the 3- and 6-positions on the naphthol framework. Rearrangement of allyl naphthyl ethers containing bromo (**1n** and **1o**), *para*-methylphenyl (**1q**) and phenylethynyl (**1r**) gave the corresponding products **2** in excellent yields and with high enantioselectivities. Single-crystal X-ray analysis of compound **2l** allowed us to establish the stereogenic center has an *S* configuration (see the ESI†). The investigation of the substrate scope revealed that substrates with an electron-donating substituent on R^2^ or an electron-withdrawing substituent on the naphthalene ring that is capable of stabilizing the allylic cation or the counter anion would undergo rearrangement smoothly and give the products **2** in high yield (entries 1–5). In addition, we found that the reactions of benzyl naphthyl ethers (**1s**, **1t** and **1u**) with a benzyl group bearing strong electron-donating substituents proceeded smoothly to give the corresponding products **2** in high yield with enantioselectivity (entries 6–8).

**Table 3:** Substrate scope^*a*^

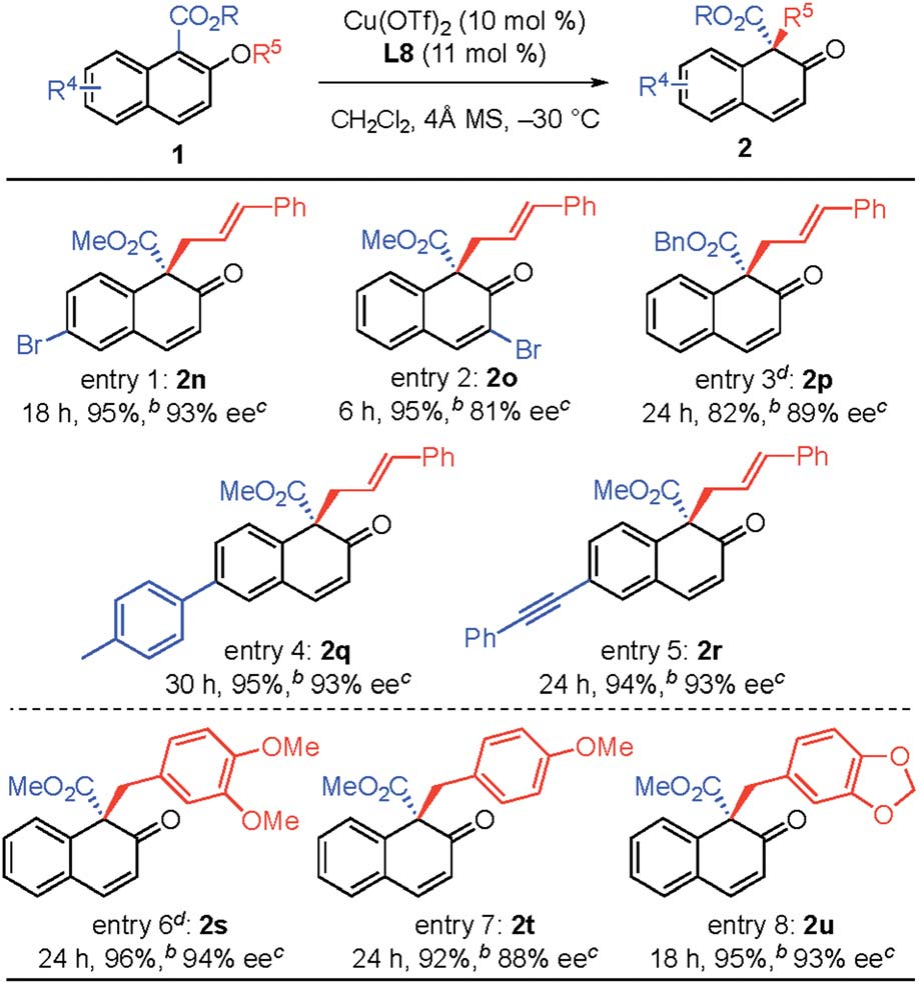

^*a*^All reactions were carried out with 0.15 mmol of **1** in dichloromethane (0.75 mL).

^*b*^Isolated yield.

^*c*^Determined by HPLC analysis.

^*d*^Carried out at –40 °C.

**Scheme 2:**
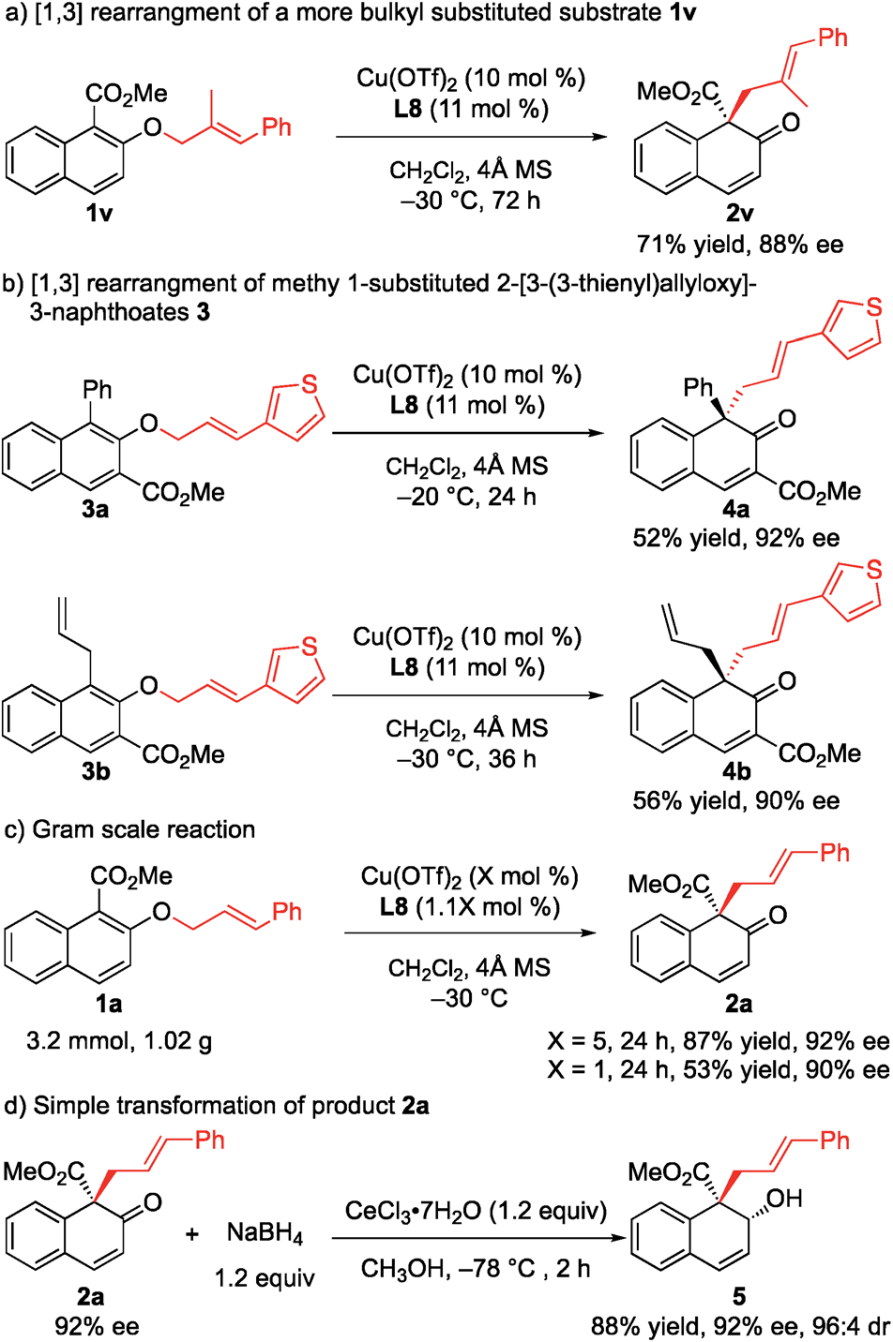
Further synthetic study of the rearrangement reaction: (a) [1,3] rearrangement of a more bulky substituted substrate **1v**. (b) [1,3] rearrangement of methyl 1-substituted 2-[3-(3-thienyl)allyloxy]-3-naphthoates 3. (c) Gram scale reaction. (d) Simple transformation of product **2a**.

To further demonstrate the utility of this strategy, a more challenging substrate, methyl (*E*)-2-[(2-methyl-3-phenylallyl)oxy]-1-naphthoate (**1v**), was used. To our delight, the rearrangement reaction proceeded smoothly to give **2v** in 71% yield with 88% ee (Scheme 2a). Furthermore, the rearrangement of methyl 1-substituted 2-[3-(3-thienyl)allyloxy]-3-naphthoates **3a** and **3b** gave the desired products **4a** and **4b** with 92% ee and 90% ee, respectively (Scheme 2b). The rearrangement reaction of **1a** was conducted on a gram scale, and **2a** was obtained in 87% yield with 92% ee when only 5 mol% catalyst was used. Notably, when the amount of catalyst was reduced to 1 mol%, the ee value was maintained while only moderate yield was observed (Scheme 2c). Treatment of **2a** with NaBH_4_ and cerium(iii) chloride heptahydrate in MeOH at –78 °C for 2 h gave the corresponding allyl alcohol **5** in 88% yield and 96 : 4 diastereoselectivity with the retention of enantiomeric purity (Scheme 2d).

**Fig. 1:**
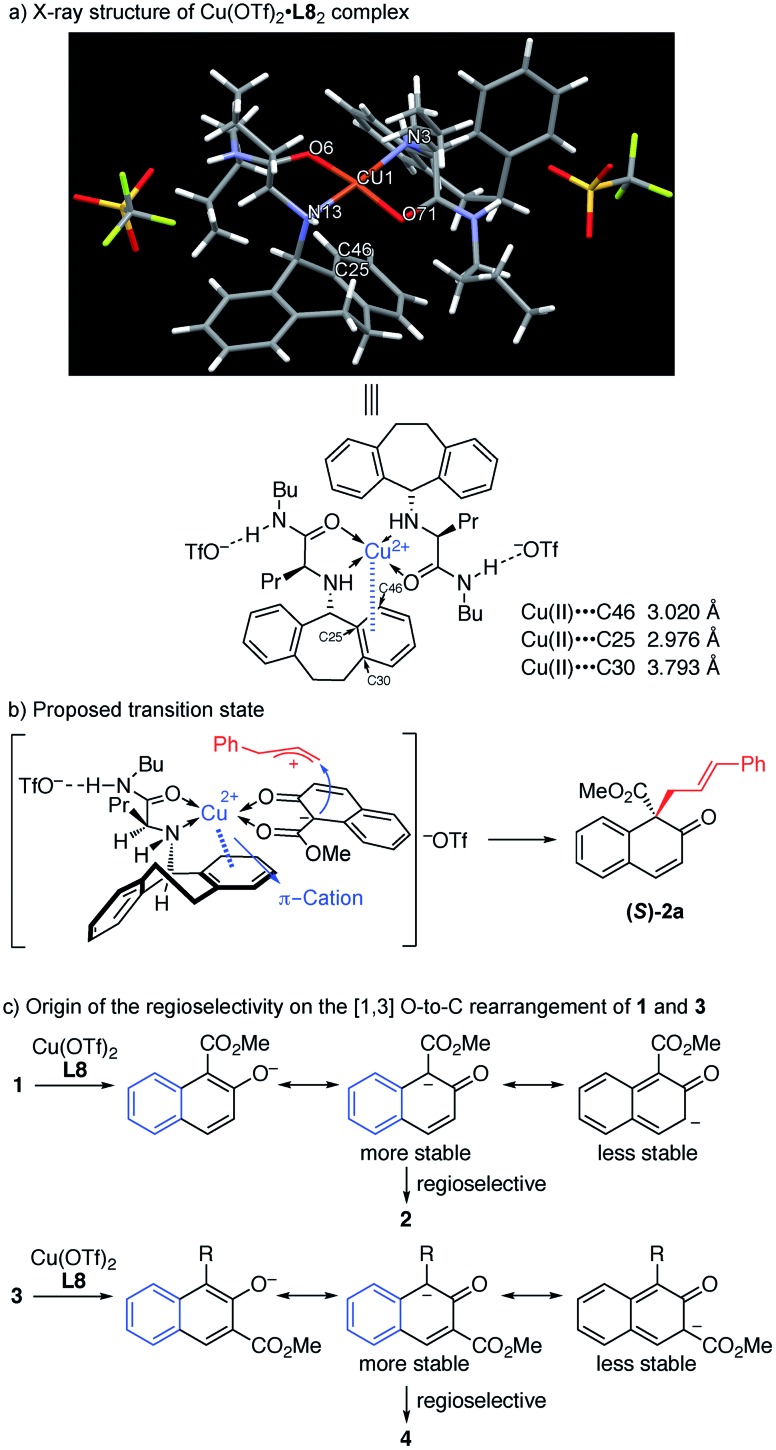
(a) X-ray structure of Cu(OTf)_2_·L8 complex. (b) Proposed transition state. (c) Origin of the regioselectivity on the [1,3] O-to-C rearrangement of **1** and **3**.

As observed in the above reaction, the *N*-5-dibenzosuberyl-substituted group of the ligand proved to be crucial in regulating asymmetric induction. To get a clear understanding of the exact role that the *N*-5-dibenzosuberyl-substituted group plays in this reaction, we tried to cultivate a single crystal of the copper complex. To our delight, we successfully obtained a single crystal of copper and the ligand in a 1 : 2 ratio. X-ray crystallographic analysis indicated that the close contact between the Cu(ii) center and the benzene ring of the ligand should be a result of π–cation interaction,^16^ which we believe is responsible for the asymmetric induction (Fig. 1a).

**Scheme 3:**
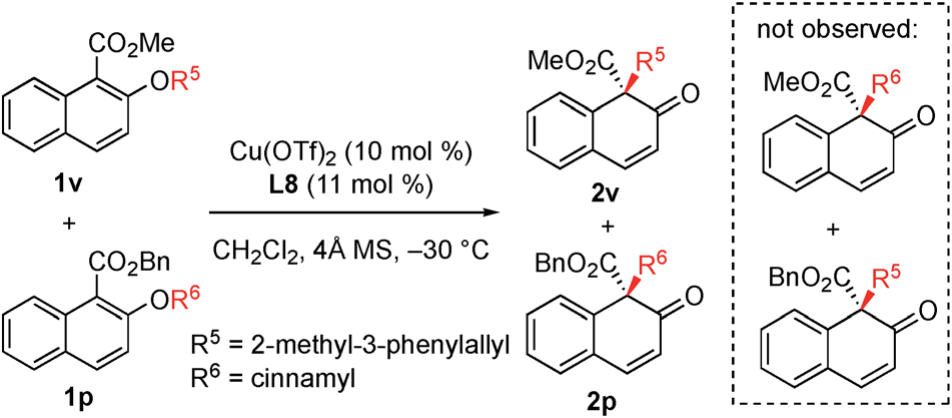
Crossover experiments.

In order to gain insight into the reaction mechanism, a crossover reaction was carried out using substrates **1v** and **1p**. As shown in Scheme 3, products **2v** and **2p** were obtained cleanly while no crossover products were detected. This indicates that the rearrangement is an intramolecular reaction and should involve a tight ion pair intermediate. The regioselectivity in the rearrangement of **1** and **3** can be understood by the resonance stability of naphthoxy anions (Fig. 1c).

In the light of these observations, we proposed the following transition state for the π–Cu(ii) complex-catalyzed [1,3] O-to-C rearrangement of allyl naphthyl ethers (Fig. 1b). The square-planar Cu(ii) center is coordinated with the N and O atoms from ligand **L8** and the two O atoms from substrate **1a** in *trans* chelation to avoid steric hindrance between the *N*-5-dibenzosuberyl group of **L8** and the cinnamyl group of **1a**.^15^ Simultaneously, substrate **1a** undergoes heterolytic C–O cleavage to form a reactive tight ion pair intermediate between the allyl cation and the counter enolate anion. The π–cation interactions between the Cu center and the *N*-5-dibenzosuberyl group of **L8** lead to the folding of the *N*-5-dibenzosuberyl group, which perfectly shields the *re* face of the naphthalenolate. The enolate then uses its *si* face to approach the allyl cation, affording the rearrangement product **2a** in *S* configuration.

## 3. Conclusions

In conclusion, we reported the successful development of enantioselective [1,3] O-to-C rearrangement of methyl 2-allyloxy/benzyloxy-1/3-naphthoates using a chiral π–Cu(ii) catalyst (1–10 mol%) for the first time. This method is practical and scalable (1 g scale). X-ray crystallographic analysis of the Cu(ii) complex provided solid evidence for the existence of π–cation interaction, which was shown to be crucial for inducing enantioselectivity. Based on the experimental results and mechanistic studies, we proposed a possible transition state involving a tight ion pair intermediate. We believe that this catalytic system may be useful for inducing asymmetry in other types of orbital symmetry-forbidden [*m*,*n*] rearrangement reactions. Studies along these lines are underway.

## 4. Methods

The ESI† contains details of X-ray crystallographic analysis of the π–Cu(ii) complex, experiments, product analyses and spectra of all characterized compounds.

## Conflicts of interest

The authors declare no competing financial interest.

## Supplementary Material

Supplementary information

Crystal structure data
