# Supplementary material for: Enantioselective [1,3] O-to-C rearrangement: dearomatization of alkyl 2-allyloxy/benzyloxy-1/3-naphthoates catalyzed by a chiral π–Cu(ii) complex
Source: Chem Sci. 2019 Jan 10;10(8):2259–63. doi: 10.1039/c8sc05601c (PMC6385897; doi:10.1039/c8sc05601c)
Supplement: Supplementary file 1 [file SC-010-C8SC05601C-s001.pdf]

Electronic Supplementary Material (ESI) for Chemical Science.
This journal is © The Royal Society of Chemistry 2019

Supporting Information

for Enantioselective [1,3] O-to-C Rearrangement: Dearomatization of Akyl 2-Allyloxy/Benzyloxy-1/3-naphthoates Catalyzed by a Chiral π–Cu(II) Complex

Lu Yao and Kazuaki Ishihara*

Graduate School of Engineering, Nagoya University, B2-3(611), Furo-cho, Chikusa, Nagoya 464-8603, Japan

E-mail: ishihara@cc.nagoya-u.ac.jp

Contents

1. General Remarks S2
2. General Procedure for the Synthesis of Ligands S2
3. General Procedure for the Synthesis of Methyl 2-Allyloxy- and 2-Benzyloxy-1-naphthoates.. S6
4. General Procedure for Catalytic Asymmetric [1,3] Rearrangement of Methyl 2-Allyloxy- and 2-Benzyloxy-1-naphthoates S13
5. Prospect for Appropriate Substrate and Optimization of the Reaction Conditions S14
6. Spectral Characterization Data for the Products S16
7. Synthetic Application of 2a S24
8. X-ray Structure of Product (*S*)-2l and Cu(II) Complex S26
9. References S28
10. ^{1}H NMR and ^{13}C NMR Spectra S29
11. HPLC Chromatograms S149

1. General Remarks

^{1}H NMR spectra were recorded on a JEOL ECS400 400 MHz spectrometer or a Bruker 500 MHz spectrometer in $CDCl_3$. Chemical shifts are reported in ppm with the internal TMS signal at 0.0 ppm as a standard. The data are reported as (s = single, d = double, t = triple, q = quartet, m = multiple or unresolved, brs = broad single, coupling constant(s) in Hz, integration). ^{13}C NMR spectra were recorded on a JEOL ECS400 100 MHz or a Bruker 125 MHz spectrometer in $CDCl_3$. Chemical shifts are reported in ppm with the internal chloroform signal at 77.0 ppm. Commercially obtained reagents were used without further purification. All reactions were monitored by TLC with silica gel-coated plates. Infrared (IR) spectra were recorded on a JASCO FT/IR 460 plus spectrometer. Enantiomeric ratios were determined by HPLC, using a chiralpak AS-3 column, chiralpak AD-3 column and chiralpak IA-3 column with hexane and *i*-PrOH as solvents. The racemic adducts were attained by using the complex of $Cu(OTf)_2$ and racemic ligand as the catalyst. The absolute configuration of **2l** was determined unequivocally according to the X-ray diffraction analysis, and those of other adducts were deduced on the basis of this result.

2. General Procedure for the Synthesis of Ligands

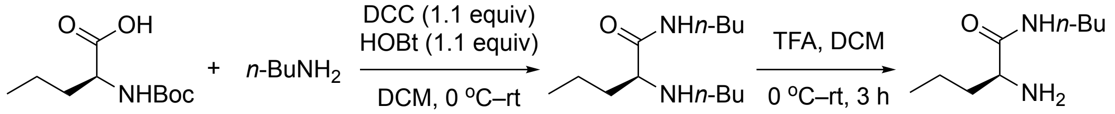

To a solution of *N*-(*tert*-butoxycarbonyl)-L-norvaline (1.08 g, 5.0 mmol) in dichloromethane (DCM, 25 mL) were added 1-hydroxybenzotriazole (HOBt, 743 mg, 5.5 mmol), DCC (1.10 g 5.5 mmol) and butylamine (0.6 mL, 6.0 mmol) at 0 °C. Then the mixture was stirred at room temperature for 16 h before quenched with 10% w/w citric acid. After the mixture was filtered, the organic layer was separated and the aqueous layer was extracted with DCM. The combined organic layer was washed with brine, dried over Na_2SO_4, filtered and then concentrated under reduced pressure. *tert*-Butyl (*S*)-(1-(butylamino)-1-oxopentan-2-yl)carbamate was obtained and directly used in next step without further purification.

To a solution of the crude *tert*-butyl (*S*)-(1-(butylamino)-1-oxopentan-2-yl)carbamate in DCM (6.4 mL) was added trifliroacetic acid (TFA, 3.2 mL) dropwise at 0 °C. The mixture was then stirred at room temperature for 3 h. The mixture was neutralized with NaOH (1 *M*). The organic layer was separated, the aqueous layer was extracted with DCM. The combined organic layer was washed with brine, dried over Na_2SO_4, filtered and then concentrated under reduced pressure. (*S*)-2-amino-*N*-butylpentanamide was obtained and directly used in next step without further purification.

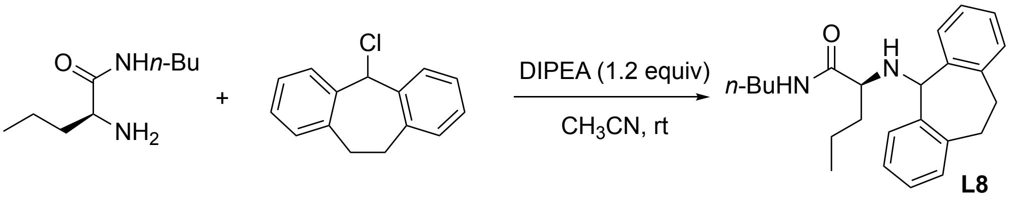

To a solution of the crude (*S*)-2-amino-*N*-butylpentanamide and *N*,*N*-diisopropylethylamine (DIPEA, 1.0 mL, 6.0 mmol) in MeCN (20 mL) was added dibenzosuberyl chloride (1.2 g, 5.25 mmol). The mixture was reacted at room tempertaure until the reaction completed (monitored by TLC). The reaction mixture was filtered through a pad of celite and washed with ethyl acetate. The filtrate was concentrated under reduced pressure. The crude product was purified by silica gel column chromatography (ethyl acetate/hexane = 1/5, v/v) to afford the ligand **L8**.

(*S*)-*N*-Butyl-2-[(10,11-dihydro-5*H*-dibenzo[a,d][7]annulen-5-yl)amino]pentanamide (L8): 60% overall yield; white solid; m.p. = 89.1 °C; $[\alpha]^{23}_{D}$ = –15.9 (*c* 1.00, $CHCl_3$); IR (film) 3304, 2957, 2929, 2871, 1644, 1552, 1445, 760 cm^{-1}; ^1H NMR (500 MHz, $CDCl_3$): δ 7.24 – 7.11 (m, 8H), 6.64 (s, 1H), 4.64 (s, 1H), 3.65 (m, 2H), 3.16 (dt, *J* = 20.1, 6.7 Hz, 1H), 3.08 – 2.96 (m, 4H), 1.66 (m, 1H), 1.57 – 1.53 (m, 2H), 1.39 – 1.26 (m, 6H), 0.91 (t, *J* = 7.2 Hz, 3H), 0.84 (t, *J* = 7.2 Hz, 3H); ^{13}C NMR (125 MHz, $CDCl_3$): ^{13}C NMR (125 MHz, $CDCl_3$) δ 174.3, 130.4, 130.3, 127.7, 127.6, 126.1, 125.9, 61.4, 38.5, 35.8, 32.8, 32.4, 31.5, 19.9, 19.0, 13.7, 13.6; HRMS (ESI+) *m*/*z* calcd. for $C_{24}H_{33}N_2O$: 365.2587, found: 365.2589.

The ligands used in screening were synthesized using the same procedure of synthesizing **L8**.

(*S*)-2-[(10,11-Dihydro-5*H*-dibenzo[a,d][7]annulen-5-yl)amino]-1-(pyrrolidin-1-yl)propan-1-one (L1):

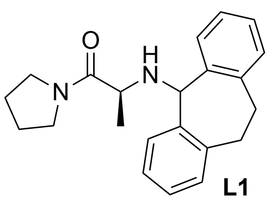

57% overall yield; colorless viscous liquid; $[\alpha]^{25}_{D}$ = +59.3 (*c* 0.71, $CHCl_3$); IR (neat) 2971, 2874, 1638, 1427, 764 cm^{-1}; ^1H NMR (400 MHz, $CDCl_3$) δ 7.34 (dd, *J* = 7.2, 1.6 Hz, 1H), 7.18 – 7.05 (m, 7H), 4.72 (s, 1H), 4.27 (brs, 1H), 3.61 – 3.47 (m, 3H), 3.13 (d, *J* = 6.6 Hz, 1H), 3.06 – 2.99 (m, 1H), 2.93 (t, *J* = 12.7 Hz, 1H), 2.87 – 2.81 (m, 1H), 2.75 (m, 1H), 2.27 (m, 1H), 1.85 – 1.82 (m, 4H), 1.13 (d, *J* = 6.9 Hz, 3H); ^{13}C NMR (100 MHz, $CDCl_3$) δ 173.5, 130.7, 129.5, 127.6, 127.1, 125.6, 125.2, 52.1, 45.4, 45.3, 33.1, 31.3, 25.7, 23.9, 18.7; HRMS (ESI+) *m*/*z* calcd. for $C_{22}H_{27}N_2O$: 335.2118, found: 335.2110.

(*S*)-2-[(10,11-Dihydro-5*H*-dibenzo[a,d][7]annulen-5-yl)amino]-1-(pyrrolidin-1-yl)butan-1-one (L2):

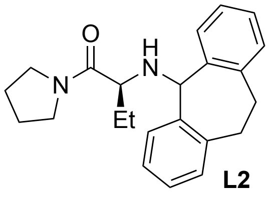

54% overall yield; yellow viscous liquid; $[\alpha]^{26}_{D}$ = +57.8 (*c* 0.71, $CHCl_3$); IR (neat) 2967, 2871, 1636, 1424, 762 cm^{-1}; ^1H NMR (400 MHz, $CDCl_3$) δ 7.33

(d, J = 7.0 Hz, 1H), 7.20 – 7.05 (m, 7H), 4.67 (s, 1H), 4.35 (brs, 1H), 3.63 – 3.47 (m, 3H), 3.08 – 2.74 (m, 5H), 2.17 (m, 1H), 1.85 (m, 4H), 1.47 (td, J = 13.8, 6.9 Hz, 2H), 0.89 (t, J = 7.2 Hz, 3H); ^{13}C NMR (100 MHz, $CDCl_3$) δ 173.3, 130.9, 129.6, 127.6, 127.2, 125.7, 125.2, 58.5, 45.5, 45.4, 33.2, 31.3, 26.5, 25.8, 24.0, 10.8; HRMS (ESI+) m/z calcd. for $C_{23}H_{29}N_2O$: 349.2274, found: 349.2265.

(*S*)-2-[(10,11-Dihydro-5*H*-dibenzo[a,d][7]annulen-5-yl)amino]-3-methyl-1-(pyrrolidin-1-yl)butan-1-one (L3):

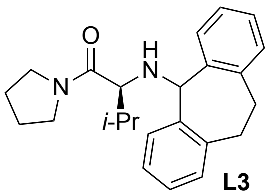

L3

61% overall yield; white solid; m.p. = 89.8 °C; $[\alpha]^{26}_{D}$ = +74.3 (c 0.71, $CHCl_3$); IR (film) 2956, 2871, 1637, 1492, 1422, 767 cm^{-1}; ^{1}H NMR (400 MHz, $CDCl_3$) δ 7.32 (d, J = 6.8 Hz, 1H), 7.18 – 7.03 (m, 7H), 4.58 (s, 1H), 4.46 (brs, 1H), 3.68 – 3.51 (m, 3H), 3.09 (dt, J = 9.6, 6.8 Hz, 1H), 2.91 – 2.72 (m, 4H), 2.23 (m, 1H), 1.89 – 1.84 (m, 4H), 1.71 (s, 1H), 0.93 – 0.82 (m, 6H); ^{13}C NMR (125 MHz, $CDCl_3$) δ 173.2, 131.0, 129.6, 127.8, 127.3, 125.9, 125.3, 62.8, 45.9, 45.5, 33.4, 31.7, 31.2, 26.0, 24.2, 20.0, 18.4; HRMS (ESI+) m/z calcd. for $C_{24}H_{30}N_2NaO$: 385.2250, found: 385.2241.

(*S*)-2-[(10,11-Dihydro-5*H*-dibenzo[a,d][7]annulen-5-yl)amino]-1-(pyrrolidin-1-yl)pentan-1-one (L4):

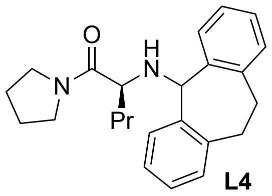

L4

63% overall yield; yellow viscous liquid; $[\alpha]^{27}_{D}$ = +49.8 (c 0.71, $CHCl_3$); IR (neat) 2956, 2871, 1637, 1493, 1422, 768 cm^{-1}; ^{1}H NMR (400 MHz, $CDCl_3$) δ 7.34 (d, J = 6.8 Hz, 1H), 7.20 – 7.04 (m, 7H), 4.66 (s, 1H), 4.36 (brs, 1H), 3.63 – 3.50 (m, 3H), 3.06 – 2.84 (m, 4H), 2.73 (m, 1H), 2.21 (m, 1H), 1.84 – 1.78 (m, 4H), 1.51 – 1.27 (m, 4H), 0.75 (t, J = 6.9 Hz, 3H); ^{13}C NMR (125 MHz, $CDCl_3$) δ 173.4, 130.8, 129.5, 127.6, 127.1, 125.7, 125.1, 56.4, 45.4, 45.3, 35.3, 33.2, 31.2, 25.8, 23.9, 19.0, 13.4; HRMS (ESI+) m/z calcd. for $C_{24}H_{30}N_2NaO$: 385.2250, found: 385.2247.

(*S*)-2-[(10,11-Dihydro-5*H*-dibenzo[a,d][7]annulen-5-yl)amino]-1-(pyrrolidin-1-yl)hexan-1-one (L5):

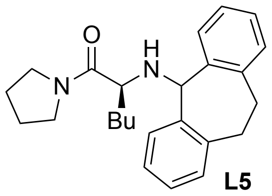

L5

62% overall yield; yellow viscous liquid; $[\alpha]^{27}_{D}$ = +45.6 (c 0.71, $CHCl_3$); IR (neat) 2952, 2865, 1638, 1421, 765 cm^{-1}; ^{1}H NMR (400 MHz, $CDCl_3$) δ 7.34 (d, J = 6.9 Hz, 1H), 7.20 – 7.05 (m, 7H), 4.67 (s, 1H), 4.37 (brs, 1H), 3.64 – 3.50 (m, 3H), 3.06 – 3.02 (m, 4H), 2.74 (m, 1H), 2.12 (m, 1H), 1.86 – 1.81 (m, 4H), 1.41 – 1.14 (m, 6H), 0.81 (t, J = 7.2 Hz, 3H); ^{13}C NMR (125 MHz, $CDCl_3$) δ 173.5, 130.9, 129.5, 127.6, 127.2, 125.8, 125.2, 56.8, 45.5, 45.4, 33.3, 33.0, 31.3, 28.1, 25.9, 24.0, 22.2, 13.8; HRMS (ESI+) m/z calcd. for $C_{25}H_{32}N_2NaO$: 399.2407, found: 399.2398.

(*S*)-2-[(10,11-Dihydro-5*H*-dibenzo[a,d][7]annulen-5-yl)amino]-*N*-ethylpentanamide (L6):

58% overall yield; white solid; m.p. = 115.4 °C; $[\alpha]^{23}_{D}$ = –14.6 (c 0.71, $CHCl_3$); IR (film) 3302, 2903, 2864, 2359, 1646, 1541, 761 cm^{-1}; ^{1}H NMR (400 MHz, $CDCl_3$) δ 7.24 – 7.11 (m, 8H), 6.61

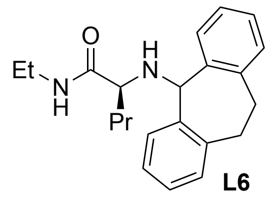
L6

(s, 1H), 4.64 (s, 1H), 3.67 (m, 2H), 3.15 (ddd, J = 21.6, 13.8, 7.2 Hz, 2H), 3.06 (dd, J = 7.7, 5.0 Hz, 1H), 2.96 (m, 2H), 1.95 (m, 1H), 1.69 – 1.62 (m, 1H), 1.53 – 1.50 (m, 1H), 1.40 – 1.24 (m, 2H), 1.03 (t, J = 7.3 Hz, 3H), 0.84 (t, J = 7.3 Hz, 3H); 13C NMR (125 MHz, $CDCl_3$) δ 174.3, 130.5, 130.4, 127.8, 127.7, 126.2, 126.1, 61.7, 35.9, 33.7, 32.9, 32.6, 19.1, 14.7, 13.8; HRMS (ESI+) *m/z* calcd. for $C_{22}H_{28}N_2NaO$: 359.2094, found: 359.2085.

(*S*)-2-[(10,11-Dihydro-5*H*-dibenzo[a,d][7]annulen-5-yl)amino]-*N*-propylpentanamide (L7):

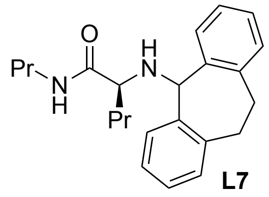
L7

61% overall yield; white solid; m.p. = 107.6 °C; $[\alpha]^{27}_{D}$ = –13.4 (*c* 0.71, $CHCl_3$); IR (film) 3298, 2958, 2929, 2871, 1638, 1557, 1444, 1156, 761 cm^{-1}; ^{1}H NMR (400 MHz, $CDCl_3$) δ 7.22 – 7.11 (m, 8H), 6.66 (s, 1H), 4.65 (s, 1H), 3.64 (brs, 2H), 3.14 (td, J = 13.4, 6.8 Hz, 1H), 3.06 – 2.98 (m, 4H), 1.98 (m, 1H), 1.68 – 1.29 (m, 6H), 0.86 (dt, J = 14.3, 7.3 Hz, 6H); 13C NMR (100 MHz, $CDCl_3$) δ 174.4, 130.4, 130.3, 127.7, 127.6, 126.1, 126.0, 61.4, 40.5, 35.8, 32.8, 32.4, 22.7, 19.0, 13.7, 11.3; HRMS (ESI+) *m/z* calcd. for $C_{23}H_{30}N_2NaO$: 373.2250, found: 373.2241.

(*S*)-2-[(10,11-Dihydro-5*H*-dibenzo[a,d][7]annulen-5-yl)amino]-*N*-neopentylpentanamide (L9):

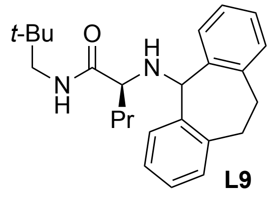
L9

56% overall yield; white solid; m.p. = 108.3 °C; $[\alpha]^{28}_{D}$ = –13.6 (*c* 0.71, $CHCl_3$); IR (film) 3313, 3061, 3016, 2957, 2869, 1651, 757 cm^{-1}; ^{1}H NMR (400 MHz, $CDCl_3$) δ 7.22 – 7.11 (m, 8H), 6.79 (s, 1H), 4.69 (s, 1H), 3.66 (m, 2H), 3.11 – 2.98 (m, 4H), 2.76 (m, 1H), 2.00 (m, 1H), 1.70 – 1.34 (m, 4H), 0.88 – 0.84 (m, 12H); 13C NMR (125 MHz, $CDCl_3$) δ 174.4, 130.5, 130.4, 127.8, 127.7, 126.2, 126.1, 61.7, 50.1, 36.0, 32.9, 32.6, 31.5, 27.1, 19.1, 13.8; HRMS (ESI+) *m/z* calcd. for $C_{25}H_{34}N_2NaO$: 401.2563, found: 401.2558.

(*S*)-2-[(10,11-Dihydro-5*H*-dibenzo[a,d][7]annulen-5-yl)amino]-*N*-isopropylpentanamide (L10):

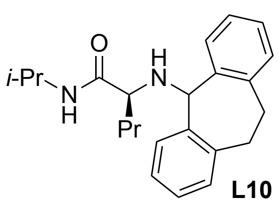
L10

60% overall yield; white solid; m.p. = 114.0 °C; $[\alpha]^{24}_{D}$ = –11.2 (*c* 0.71, $CHCl_3$); IR (film) 3301, 2964, 2873, 2360, 1639, 1543, 1456, 761 cm^{-1}; ^{1}H NMR (400 MHz, $CDCl_3$) δ 7.24 – 7.11 (m, 8H), 6.52 (d, J = 7.6 Hz, 1H), 4.63 (brs, 1H), 3.96 (ddt, J = 13.1, 8.0, 6.6 Hz, 1H), 3.68 (m, 2H), 3.03 – 2.97 (m, 3H), 1.94 (m, 1H), 1.69 – 1.63 (m, 1H), 1.51-1.49 (m, 1H), 1.36 – 1.29 (m, 2H), 1.10 (d, J = 6.6 Hz, 3H), 0.99 (d, J = 6.6 Hz, 3H), 0.84 (t, J = 7.3 Hz, 3H); ^{13}C NMR (125 MHz, $CDCl_3$) δ 173.5, 130.6, 130.4, 127.8, 127.7, 126.2, 126.0, 61.6, 40.6, 35.9, 32.9, 32.6, 22.8, 22.4, 19.1, 13.8; HRMS (ESI+) *m/z* calcd. for $C_{23}H_{30}N_2NaO$: 373.2250, found: 373.2251.

3. General Procedure for the Synthesis of Methyl 2-Allyloxy- and 2-Benzyloxy-1-naphthoates

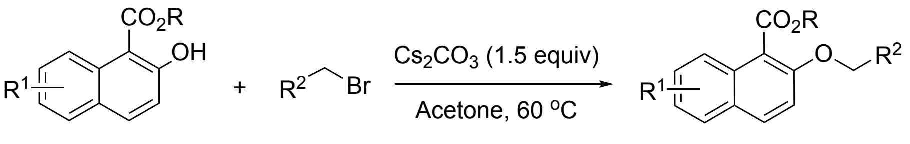

1

The starting naphthols[1] (6 mmol) and Cs_2CO_3 (9 mmol) were dissolved in acetone (40 mL). Cinnamyl bromide[2] (or benzyl bromide) (8 mmol) was added by syringe and the reaction was heated to 60 °C until TLC revealed complete conversion of naphthols. The mixture was diluted with ethyl acetate and water after cooling. The organic layer was washed with brine, dried with anhydrous Na_2SO_4, and concentrated under vacuum. The residue was purified by silica gel chromatography to afford corresponding product.

Methyl 2-(cinnamyloxy)-1-naphthoate (1a):

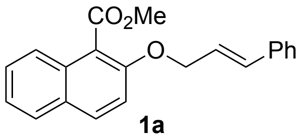

1a

1a was isolated by FC on silica gel using pentane/EtOAc 30:1; 86% yield; white solid; m.p. = 114.0 °C; IR (film) 2945, 1725, 1286, 1236, 756 cm^{-1}; ^{1}H NMR (500 MHz, $CDCl_3$) δ 7.88 (d, J = 9.1 Hz, 1H), 7.79 (d, J = 8.2 Hz, 1H), 7.76 (d, J = 8.5 Hz, 1H), 7.52 – 7.49 (m, 1H), 7.41 – 7.37 (m, 3H), 7.34 – 7.31 (m, 3H), 7.27 – 7.24 (m, 1H), 6.74 (d, J = 16.0 Hz, 1H), 6.41 (dt, J = 16.0, 5.6 Hz, 1H), 4.88 (dd, J = 5.5, 1.5 Hz, 2H), 4.04 (s, 3H); ^{13}C NMR (125 MHz, $CDCl_3$) δ 168.3, 153.5, 136.3, 132.6, 131.5, 130.9, 128.6, 128.5, 128.0, 127.8, 127.5, 126.4, 124.2, 124.1, 123.8, 118.4, 114.8, 70.2, 52.3; HRMS (ESI+) m/z calcd. for $C_{21}H_{18}NaO_3$: 341.1148; found: 341.1146.

Methyl (*E*)-2-{[3-(*o*-tolyl)allyl]oxy}-1-naphthoate (1b):

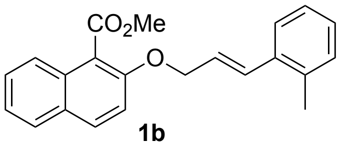

1b

1b was isolated by FC on silica gel using pentane/EtOAc 30:1; 78% yield; white solid; m.p. = 86.1 °C; IR (film) 3025, 2948, 1726, 1510, 1437, 1284, 1233, 809, 747 cm^{-1}; ^{1}H NMR (500 MHz, $CDCl_3$) δ 7.89 (d, J = 9.1 Hz, 1H), 7.79 (d, J = 8.2 Hz, 1H), 7.76 (d, J = 8.5 Hz, 1H), 7.50 (ddd, J = 8.4, 6.9, 1.2 Hz, 1H), 7.45 (dd, J = 5.3, 3.7 Hz, 1H), 7.38 (ddd, J = 8.0, 6.9, 1.1 Hz, 1H), 7.33 (d, J = 9.1 Hz, 1H), 7.18 – 7.13 (m, 3H), 6.97 (d, J = 15.8 Hz, 1H), 6.28 (dt, J = 15.8, 5.5 Hz, 1H), 4.90 (dd, J = 5.5, 1.6 Hz, 2H), 4.04 (s, 3H), 2.33 (s, 3H); ^{13}C NMR (125 MHz, $CDCl_3$) δ 168.4, 153.5, 135.5, 131.4, 130.9, 130.6, 130.2, 128.7, 128.0, 127.7, 127.5, 126.0, 125.7, 125.4, 124.2, 123.8, 118.4, 114.8, 70.3, 52.3, 19.6; HRMS (ESI+) m/z calcd. for $C_{22}H_{20}NaO_3$: 355.1305; found: 355.1298.

Methyl (*E*)-2-{[3-(*m*-tolyl)allyl]oxy}-1-naphthoate (1c):

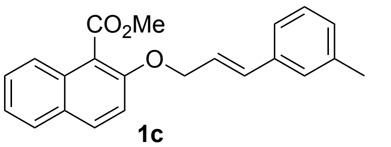

1c

1c was isolated by FC on silica gel using pentane/EtOAc 30:1; 67% yield; yellow liquid; IR (neat) 2941, 1727, 1510, 1283, 1235, 748 cm^{-1}; ^{1}H NMR (500 MHz, $CDCl_3$) δ 7.87 (d, J = 9.1 Hz, 1H), 7.79 (d,

J = 8.2 Hz, 1H), 7.76 (d, J = 8.6 Hz, 1H), 7.52 – 7.48 (m, 1H), 7.39 – 7.36 (m, 1H), 7.31 (d, J = 9.1 Hz, 1H), 7.22 – 7.20 (m, 3H), 7.08 – 7.06 (m, 1H), 6.71 (d, J = 16.0 Hz, 1H), 6.39 (dt, J = 16.0, 5.6 Hz, 1H), 4.87 (dd, J = 5.6, 1.5 Hz, 2H), 4.04 (s, 3H), 2.34 (s, 3H); ^{13}C NMR (125 MHz, $CDCl_3$) δ 168.4, 153.6, 138.1, 136.3, 132.9, 131.5, 131.0, 128.7, 128.4, 128.0, 127.5, 127.2, 124.3, 124.0, 123.8, 123.7, 118.4, 114.8, 70.4, 52.4 21.3; HRMS (ESI+) *m/z* calcd. for $C_{22}H_{20}NaO_3$: 355.1305; found: 355.1303.

Methyl (*E*)-2-{[3-(*p*-tolyl)allyl]oxy}-1-naphthoate (1d):

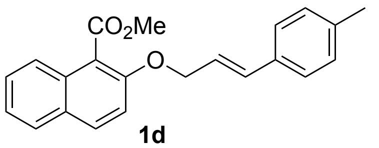

1d

1d was isolated by FC on silica gel using pentane/EtOAc 30:1; 83% yield; white solid; m.p. = 95.5 °C; IR (film) 2950, 1726, 1510, 1283, 1234, 754 cm^{-1}; ^{1}H NMR (500 MHz, $CDCl_3$) δ 7.87 (d, J = 9.1 Hz, 1H), 7.79 (d, J = 8.2 Hz, 1H), 7.76 (d, J = 8.5 Hz, 1H), 7.50 (t, J = 7.6 Hz, 1H), 7.38 (t, J = 7.5 Hz, 1H), 7.32 – 7.29 (m, 3H), 7.14 – 7.12 (m, 2H), 6.71 (d, J = 16.0 Hz, 1H), 6.35 (dt, J = 15.9, 5.7 Hz, 1H), 4.87 (dd, J = 5.7, 1.4 Hz, 2H), 4.04 (s, 3H), 2.34 (s, 3H); ^{13}C NMR (125 MHz, $CDCl_3$) δ 168.4, 153.6, 137.8, 133.6, 132.8, 131.5, 131.0, 129.3, 128.7, 128.0, 127.5, 126.4, 124.3, 123.8, 123.2, 118.5, 115.0, 70.6, 52.4, 21.2; HRMS (ESI+) *m/z* calcd. for $C_{22}H_{20}NaO_3$: 355.1305; found: 355.1297.

Methyl (*E*)-2-{[3-(4-bromophenyl)allyl]oxy}-1-naphthoate (1e):

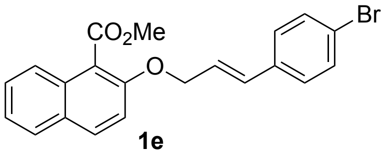

1e

1e was isolated by FC on silica gel using pentane/EtOAc 30:1; 83% yield; white solid; m.p. = 111.4 °C; IR (film) 2946, 1726, 1510, 1284, 1234, 748 cm^{-1}; ^{1}H NMR (500 MHz, $CDCl_3$) δ 7.88 (d, J = 9.1 Hz, 1H), 7.80 (d, J = 8.2 Hz, 1H), 7.76 (d, J = 8.5 Hz, 1H), 7.51 (t, J = 8.3 Hz, 1H), 7.45 – 7.43 (m, 2H), 7.39 (t, J = 8.0 Hz, 1H), 7.29 (d, J = 9.1 Hz, 1H), 7.27–7.25 (m, 2H), 6.68 (d, J = 16.0 Hz, 1H), 6.39 (dt, J = 16.0, 5.4 Hz, 1H), 4.87 (dd, J = 5.4, 1.5 Hz, 2H), 4.04 (s, 3H); ^{13}C NMR (125 MHz, $CDCl_3$) δ 168.3, 153.4, 135.3, 131.7, 131.6, 131.4, 131.0, 128.8, 128.0, 128.0, 127.6, 125.1, 124.4, 123.8, 121.7, 118.5, 114.8, 70.1, 52.4; HRMS (ESI+) *m/z* calcd. for $C_{21}H_{17}BrNaO_3$: 419.0253; found: 419.0244.

Methyl (*E*)-2-{[3-(4-chlorophenyl)allyl]oxy}-1-naphthoate (1f):

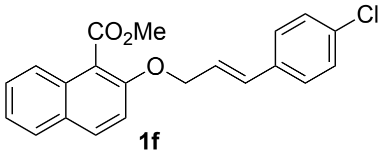

1f

1f was isolated by FC on silica gel using pentane/EtOAc 30:1; 73% yield; white solid; m.p. = 111.8 °C; IR (film) 2947, 1725, 1282, 1234 cm^{-1}; ^{1}H NMR (500 MHz, $CDCl_3$) δ 7.89 (d, J = 9.1 Hz, 1H), 7.80 (d, J = 8.2 Hz, 1H), 7.76 (d, J = 8.5 Hz, 1H), 7.51 (t, J = 8.3 Hz, 1H), 7.39 (t, J = 8.0 Hz, 1H), 7.33 – 7.28 (m, 5H), 6.70 (d, J = 16.0 Hz, 1H), 6.38 (dt, J = 16.0, 5.5 Hz, 1H), 4.88 (dd, J = 5.5, 1.6 Hz, 2H), 4.04 (s, 3H); ^{13}C NMR (125 MHz, $CDCl_3$) δ 168.3, 153.4, 134.8, 133.4, 131.5, 131.3, 130.9, 128.7, 128.6, 128.0, 127.7, 127.6, 124.9, 124.3, 123.8, 118.4, 114.7, 70.0, 52.3; HRMS (ESI+) *m/z* calcd. for $C_{21}H_{17}ClNaO_3$: 375.0758; found: 375.0749

Methyl (*E*)-2-{[3-(naphthalen-1-yl)allyl]oxy}-1-naphthoate (1g):

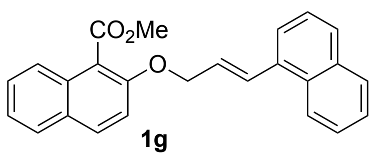

1g

1g was isolated by FC on silica gel using pentane/EtOAc 30:1; 48% yield; light yellow solid; m.p. = 136.3 °C; IR (film) 3095, 2951, 1726, 1510, 1284, 1234, 788 cm^{-1}; ^{1}H NMR (500 MHz, $CDCl_3$) δ 8.08 – 8.07 (m, 1H), 7.91 (d, *J* = 9.0 Hz, 1H), 7.86 – 7.77 (m, 4H), 7.61 (d, *J* = 7.0 Hz, 1H), 7.54 – 7.37 (m, 7H), 6.43 (dt, *J* = 15.7, 5.3 Hz, 1H), 5.00 (d, *J* = 4.4 Hz, 2H), 4.05 (s, 3H); ^{13}C NMR (125 MHz, $CDCl_3$) δ 168.4, 153.6, 134.2, 133.5, 131.6, 131.1, 131.0, 129.9, 128.8, 128.4, 128.2, 128.1, 127.6, 127.3, 126.1, 125.8, 125.5, 124.3, 123.9, 123.8, 123.7, 118.5, 114.8, 70.2, 52.4; HRMS (ESI+) *m*/*z* calcd. for $C_{25}H_{20}NaO_3$: 391.1305; found: 391.1296.

Methyl (*E*)-2-{[3-(naphthalen-2-yl)allyl]oxy}-1-naphthoate (1h):

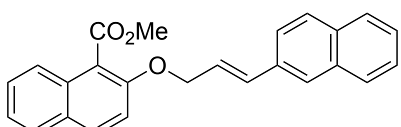

1h

1h was isolated by FC on silica gel using pentane/EtOAc 30:1; 53% yield; white solid; m.p. = 86.0 °C; IR (film) 3063, 2954, 1726, 1284, 1235, 787 cm^{-1}; ^{1}H NMR (500 MHz, $CDCl_3$) δ 7.90 (d, *J* = 9.1 Hz, 1H), 7.81 – 7.75 (m, 6H), 7.62 (d, *J* = 8.6 Hz, 1H), 7.51 (t, *J* = 8.0 Hz, 1H), 7.48 – 7.43 (m, 2H), 7.40 (t, *J* = 8.0 Hz, 1H), 7.35 (d, *J* = 9.1 Hz, 1H), 6.90 (d, *J* = 16.0 Hz, 1H), 6.54 (dt, *J* = 15.9, 5.5 Hz, 1H), 4.94 (dd, *J* = 5.5, 1.4 Hz, 2H), 4.06 (s, 3H); ^{13}C NMR (125 MHz, $CDCl_3$) δ 168.4, 153.6, 133.8, 133.5, 133.1, 132.8, 131.6, 131.0, 128.7, 128.2, 128.0, 127.9, 127.6, 127.5, 126.7, 126.3, 126.0, 124.6, 124.3, 123.8, 123.4, 118.4, 114.8, 70.4, 52.4; HRMS (ESI+) *m*/*z* calcd. for $C_{25}H_{20}NaO_3$: 391.1305; found: 391.1295.

Methyl (*E*)-2-{[3-(furan-3-yl)allyl]oxy}-1-naphthoate (1i):

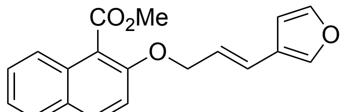

1i

1i was isolated by FC on silica gel using pentane/EtOAc 15:1; 46% yield; yellow viscous liquid; IR (neat) 2950, 1725, 1510, 1438, 1285, 1238, 751 cm^{-1}; ^{1}H NMR (500 MHz, $CDCl_3$) δ 7.88 (d, *J* = 9.1 Hz, 1H), 7.79 (d, *J* = 8.2 Hz, 1H), 7.75 (d, *J* = 8.2 Hz, 1H), 7.50 (t, *J* = 7.1 Hz, 1H), 7.43 (s, 1H), 7.40 – 7.37 (m, 2H), 7.29 (d, *J* = 9.1 Hz, 1H), 6.61.6.54 (m, 2H), 6.13 (dt, *J* = 15.8, 5.6 Hz, 1H), 4.82 (dd, *J* = 5.6, 1.4 Hz, 2H), 4.04 (s, 3H); ^{13}C NMR (125 MHz, $CDCl_3$) δ 168.4, 153.6, 143.6, 140.8, 131.5, 131.0, 128.7, 128.0, 127.6, 124.3, 123.8, 123.7, 123.4, 122.8, 118.4, 114.9, 107.5, 70.3, 52.4; HRMS (ESI+) *m*/*z* calcd. for $C_{19}H_{16}NaO_4$: 331.0941; found: 331.0941.

Methyl (*E*)-2-{[3-(thiophen-3-yl)allyl]oxy}-1-naphthoate (1j):

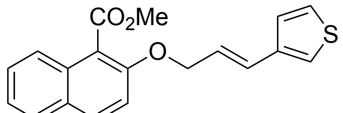

1j

1j was isolated by FC on silica gel using pentane/EtOAc 15:1; 47% yield; light brown solid; m.p. = 96.3 °C; IR (film) 2946, 1726, 1512, 1278, 1234 cm^{-1}; ^{1}H NMR (500 MHz, $CDCl_3$) δ 7.88 (d, *J* = 9.1 Hz, 1H), 7.79 (d, *J* = 8.2 Hz, 1H), 7.75 (d, *J* = 8.2 Hz, 1H), 7.50 (t, *J* = 7.0 Hz, 1H), 7.38 (t, *J* = 7.0 Hz, 1H), 7.30 (d, *J* = 9.1 Hz, 1H), 7.29 – 7.27 (m, 1H), 7.23 – 7.22 (m, 1H), 7.19 – 7.18 (m, 1H), 6.74 (d, *J* = 15.9 Hz, 1H), 6.26 (dt, *J* = 15.9, 5.6 Hz, 1H), 4.85 (dd, *J* = 5.6, 1.5 Hz, 2H), 4.04 (s, 3H); ^{13}C

NMR (125 MHz, $CDCl_3$) δ 168.4, 153.5, 138.9, 131.5, 130.9, 128.6, 128.0, 127.5, 127.0, 126.1, 124.9, 124.2, 124.0, 123.8, 122.7, 118.3, 114.8, 70.2, 52.3; HRMS (ESI+) *m*/*z* calcd. for $C_{19}H_{16}NaO_3S$: 347.0712; found: 347.0716.

Methyl (*E*)-2-[(3-phenylbut-2-en-1-yl)oxy]-1-naphthoate (1k):

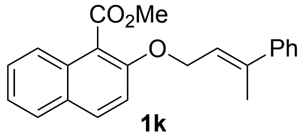

1k

1k was isolated by FC on silica gel using pentane/EtOAc 30:1; 85% yield; white solid; m.p. = 68.7 °C; IR (film) 2947, 1728, 1510, 1438, 1283, 1234, 756 cm^{-1}; ^1H NMR (500 MHz, $CDCl_3$) δ 7.88 (d, *J* = 9.1 Hz, 1H), 7.79 (d, *J* = 8.2 Hz, 1H), 7.74 (d, *J* = 8.2 Hz, 1H), 7.50 (t, *J* = 8.3 Hz, 1H), 7.44 – 7.26 (m, 7H), 6.05 (t, *J* = 6.2 Hz, 1H), 4.93 (d, *J* = 6.2 Hz, 2H), 4.03 (s, 3H), 2.14 (s, 3H); ^{13}C NMR (125 MHz, $CDCl_3$) δ 168.4, 153.6, 142.4, 139.0, 131.4, 130.9, 128.6, 128.2, 128.0, 127.5, 127.4, 125.7, 124.2, 123.7, 122.6, 118.4, 114.8, 67.2, 52.3, 16.3; HRMS (ESI+) *m*/*z* calcd. for $C_{22}H_{20}NaO_3$: 355.1310; found: 355.1298.

Methyl 2-{[3,3-bis(4-chlorophenyl)allyl]oxy}-1-naphthoate (1l):

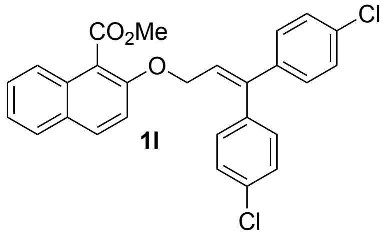

1l

1l was isolated by FC on silica gel using pentane/EtOAc 30:1; 88% yield; white solid; m.p. = 105.8 °C; IR (film) 2950, 1729, 1491, 1283, 1235, 1091, 1014, 751 cm^{-1}; ^1H NMR (500 MHz, $CDCl_3$) δ 7.82 (d, *J* = 9.1 Hz, 1H), 7.78 (d, *J* = 8.2 Hz, 1H), 7.74 (d, *J* = 8.2 Hz, 1H), 7.50 (t, *J* = 8.2 Hz, 1H), 7.40 – 7.37 (m, 3H), 7.27 – 7.24 (m, 2H), 7.16 – 7.13 (m, 4H), 7.07 (d, *J* = 9.1 Hz, 1H), 6.32 (t, *J* = 6.7 Hz, 1H), 4.73 (d, *J* = 6.7 Hz, 2H), 4.03 (s, 3H); ^{13}C NMR (125 MHz, $CDCl_3$) δ 168.4, 153.2, 143.9, 139.5, 136.7, 134.1, 134.0, 131.5, 131.0, 130.9, 128.9, 128.7, 128.5, 128.0, 127.6, 124.4, 124.4, 123.8, 118.7, 114.8, 67.5, 52.4; HRMS (ESI+) *m*/*z* calcd. for $C_{27}H_{20}Cl_2NaO_3$: 485.0682; found: 485.0687.

Methyl 2-[(3-methylbut-2-en-1-yl)oxy]-1-naphthoate (1m):

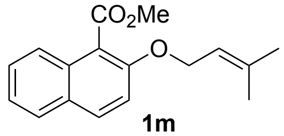

1m

1m was isolated by FC on silica gel using pentane/EtOAc 30:1; 78% yield; yellow liquid; IR (neat) 2949, 1731, 1510, 1473, 1283, 1234, 1053, 810, 748 cm^{-1}; ^1H NMR (500 MHz, $CDCl_3$) δ 7.87 (d, *J* = 9.1 Hz, 1H), 7.79 (d, *J* = 8.2 Hz, 1H), 7.73 (d, *J* = 8.2 Hz, 1H), 7.49 (t, *J* = 7.1 Hz, 1H), 7.37 (t, *J* = 7.1 Hz, 1H), 7.28 (s, 1H), 5.49 – 5.46 (m, 1H), 4.70 (d, *J* = 6.6 Hz, 2H), 4.02 (s, 3H), 1.77 (s, 3H), 1.74 (s, 3H); ^{13}C NMR (125 MHz, $CDCl_3$) δ 168.4, 153.7, 137.7, 131.2, 130.9, 128.4, 127.9, 127.3, 124.0, 123.6, 119.6, 118.3, 114.9, 66.7, 52.1, 25.6, 18.0; HRMS (ESI+) *m*/*z* calcd. for $C_{17}H_{18}NaO_3$: 293.1148; found: 293.1150.

Methyl 6-bromo-2-(cinnamyloxy)-1-naphthoate (1n):

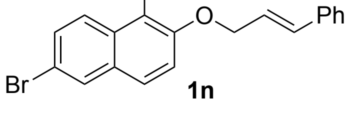

1n

1n was isolated by FC on silica gel using pentane/EtOAc 30:1; 87% yield; a light brown solid; m.p. = 86.5 °C; IR (film) 2947, 1730,1497, 1286, 1233 cm^{-1}; ^1H NMR (500 MHz, $CDCl_3$) δ 7.95 (d, *J* = 1.9 Hz,

1H), 7.79 (d, J = 9.1 Hz, 1H), 7.65 (d, J = 9.1 Hz, 1H), 7.57 – 7.55 (m, 1H), 7.41 – 7.26 (m, 6H), 6.74 (d, J = 16.0 Hz, 1H), 6.39 (dt, J = 16.0, 5.6 Hz, 1H), 4.88 (dd, J = 5.6, 1.4 Hz, 2H), 4.04 (s, 3H); ^{13}C NMR (125 MHz, $CDCl_3$) δ 167.9, 153.9, 136.2, 132.9, 130.8, 130.6, 129.9, 129.6, 129.5, 128.6, 127.9, 126.5, 125.6, 123.9, 118.4, 117.9, 115.8, 70.3, 52.5; HRMS (ESI+) *m/z* calcd. for $C_{21}H_{17}BrNaO_3$: 419.0253; found: 419.0252.

Methyl 3-bromo-2-(cinnamyloxy)-1-naphthoate (1o):

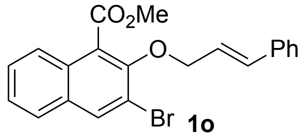

1o was isolated by FC on silica gel using pentane/EtOAc 30:1; 78% yield; colorless liquid; IR (neat) 2947, 1731, 1278, 1224, 748 cm^{-1}; ^{1}H NMR (500 MHz, $CDCl_3$) δ 8.18 (s, 1H), 7.76 (d, J = 9.4 Hz, 1H), 7.57 – 7.54 (m, 1H), 7.50 – 7.26 (m, 7H), 6.76 (d, J = 15.9 Hz, 1H), 6.50 (dt, J = 15.9, 6.2 Hz, 1H), 4.81 (dd, J = 6.2, 1.3 Hz, 2H), 4.02 (s, 3H); ^{13}C NMR (125 MHz, $CDCl_3$) δ 167.4, 150.7, 136.4, 134.4, 133.7, 131.3, 129.9, 128.6, 127.9, 127.7, 127.2, 126.7, 126.4, 125.7, 124.5, 124.2, 116.8, 75.9, 52.8; HRMS (ESI+) *m/z* calcd. for $C_{21}H_{17}BrNaO_3$: 419.0253; found: 419.0250.

Benzyl 2-(cinnamyloxy)-1-naphthoate (1p):

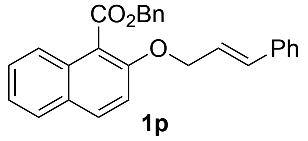

1p was isolated by FC on silica gel using pentane/EtOAc 30:1; 83% yield; white solid; m.p. = 62.3 °C; IR (film) 3025, 1727, 1510, 1281, 1226, 747 cm^{-1}; ^{1}H NMR (500 MHz, $CDCl_3$) δ 7.87 (d, J = 9.1 Hz, 1H), 7.79 – 7.42 (m, 2H), 7.49 – 7.46 (m, 3H), 7.38 – 7.25 (m, 10H), 6.70 (d, J = 16.0 Hz, 1H), 6.34 (dt, J = 15.9, 5.7 Hz, 1H), 5.51 (s, 2H), 4.85 (d, J = 5.7 Hz, 2H); ^{13}C NMR (125 MHz, $CDCl_3$) δ 167.9, 153.6, 136.3, 135.8, 133.0, 131.6, 131.0, 128.7, 128.6, 128.5, 128.4, 128.3, 128.2, 128.0, 127.9, 127.6, 126.6, 124.2, 124.1, 123.8, 118.2, 114.7, 70.3, 67.1; HRMS (ESI+) *m/z* calcd. for $C_{27}H_{22}NaO_3$: 417.1461; found: 417.1454.

Methyl 2-(cinnamyloxy)-6-(*p*-tolyl)-1-naphthoate (1q):

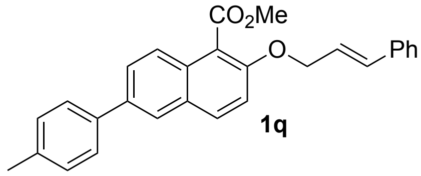

1q was isolated by FC on silica gel using pentane/EtOAc 30:1; 83% yield; white solid; IR (film) 1722, 1499, 1290, 1248, 813 cm^{-1}; ^{1}H NMR (500 MHz, $CDCl_3$) δ 7.97 (d, J = 1.6 Hz, 1H), 7.93 (d, J = 9.0 Hz, 1H), 7.83 (d, J = 8.8 Hz, 1H), 7.77 (dd, J = 8.8, 1.6 Hz, 1H), 7.60 – 7.59 (m, 2H), 7.42 – 7.40 (m, 2H), 7.37 – 7.31 (m, 3H), 7.30 – 7.26 (m, 3H), 6.76 (d, J = 16.0 Hz, 1H), 6.42 (dt, J = 16.0, 5.5 Hz, 1H), 4.90 (dd, J = 5.5, 1.4 Hz, 3H), 4.06 (s, 3H), 2.42 (s, 3H); ^{13}C NMR (125 MHz, $CDCl_3$) δ 168.4, 153.6, 137.6, 137.1, 136.9, 136.3, 132.7, 131.8, 130.0, 129.6, 129.0, 128.5, 127.9, 127.2, 127.0, 126.5, 125.4, 124.4, 124.2, 118.2, 115.2, 70.3, 52.4, 21.1; HRMS (ESI+) *m/z* calcd. for $C_{28}H_{24}NaO_3$: 431.1618; found: 431.1612.

Methyl 2-(cinnamyloxy)-6-(phenylethynyl)-1-naphthoate (1r):

1r was isolated by FC on silica gel using pentane/EtOAc 30:1; 83% yield; white solid; m.p. = 126.6 °C; IR (film) 2951, 1728, 1597, 1282, 1250, 756 cm^{-1}; ^{1}H NMR (500 MHz, $CDCl_3$) δ 8.00 (s,

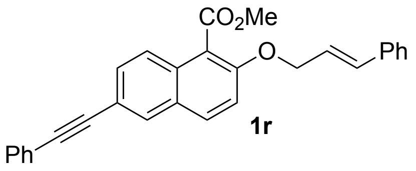
1r

1H), 7.86 (d, J = 9.1 Hz, 1H), 7.74 (d, J = 8.8 Hz, 1H), 7.61 – 7.56 (m, 3H), 7.41 – 7.26 (m, 9H), 6.75 (d, J = 16.0 Hz, 1H), 6.41 (dt, J = 16.0, 5.6 Hz, 1H), 4.90 (dd, J = 5.5, 1.2 Hz, 2H), 4.05 (s, 3H); ^{13}C NMR (125 MHz, $CDCl_3$) δ 168.1, 154.3, 136.2, 132.9, 131.6, 131.5, 130.4, 130.1, 128.6, 128.3, 127.9, 126.5, 124.0, 123.2, 119.0, 118.3, 115.3, 89.8, 89.4, 70.2, 52.5; HRMS (ESI+) *m/z* calcd. for $C_{29}H_{22}NaO_3$: 441.1461; found: 441.1458.

Methyl 2-[(3,4-dimethoxybenzyl)oxy]-1-naphthoate (1s):

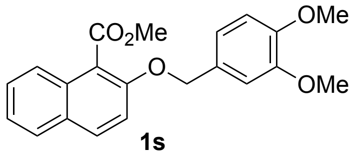
1s

1s was isolated by FC on silica gel using pentane/EtOAc 15:1; 78% yield; yellow viscous liquid; IR (neat) 2950, 2835, 1727, 1514, 1238, 1138, 1028, 811 cm^{-1}; ^{1}H NMR (500 MHz, $CDCl_3$) δ 7.83 (d, J = 9.1 Hz, 1H), 7.76 (t, J = 8.4 Hz, 2H), 7.49 (t, J = 8.1 Hz, 1H), 7.36 (t, J = 7.5 Hz, 1H), 7.27 (d, J = 9.1 Hz, 1H), 7.02 (d, J = 1.7 Hz, 1H), 6.95 (d, J = 8.1 Hz, 1H), 6.83 (d, J = 8.1 Hz, 1H), 5.19 (s, 2H), 4.00 (s, 3H), 3.89 (s, 3H), 3.86 (s, 3H); ^{13}C NMR (125 MHz, $CDCl_3$) δ 168.4, 153.5, 149.0, 148.7, 131.4, 130.9, 129.2, 128.7, 128.0, 127.5, 124.3, 123.7, 119.6, 118.5, 114.9, 110.8, 110.4, 71.4, 55.8, 55.7, 52.3; HRMS (ESI+) *m/z* calcd. for $C_{21}H_{20}NaO_5$: 375.1203; found: 375.1199.

Methyl 2-[(4-methoxybenzyl)oxy]-1-naphthoate (1t):

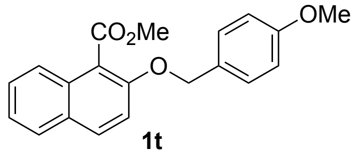
1t

1t was isolated by FC on silica gel using pentane/EtOAc 30:1; 81% yield; yellow solid; m.p. = 79.2 °C; IR (film) 2951, 1728, 1513, 1284, 1245, 812 cm^{-1}; ^{1}H NMR (500 MHz, $CDCl_3$) δ 7.80 (d, J = 9.1 Hz, 1H), 7.76 – 7.74 (m, 2H), 7.47 (t, J = 7.6 Hz, 1H), 7.36 – 7.32 (m, 3H), 7.24 (d, J = 9.1 Hz, 1H), 6.88 (d, J = 8.6 Hz, 2H), 5.15 (s, 2H), 3.98 (s, 3H), 3.76 (s, 3H); ^{13}C NMR (125 MHz, $CDCl_3$) δ 168.4, 159.3, 153.6, 131.4, 130.9, 128.7, 128.6, 128.0, 127.5, 124.2, 123.8, 118.5, 115.0, 113.8, 71.3, 55.2, 52.3; HRMS (ESI+) *m/z* calcd. for $C_{20}H_{18}NaO_4$: 345.1097; found: 345.1095.

Methyl 2-(benzo[d][1,3]dioxol-5-ylmethoxy)-1-naphthoate (1u):

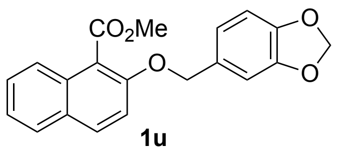
1u

1u was isolated by FC on silica gel using pentane/EtOAc 20:1; 85% yield; yellow viscous liquid; IR (neat) 2950, 1727, 1504, 1445, 1244, 1038, 810 cm^{-1}; ^{1}H NMR (500 MHz, $CDCl_3$) δ 7.82 (d, J = 9.0 Hz, 1H), 7.76 (d, J = 8.6 Hz, 2H), 7.48 (t, J = 7.6 Hz, 1H), 7.37 (d, J = 7.6 Hz, 1H), 7.24 (d, J = 9.0 Hz, 1H), 6.93 (s, 1H), 6.86 (d, J = 7.9 Hz, 1H), 6.77 (d, J = 7.9 Hz, 1H), 5.93 (s, 2H), 5.12 (s, 2H), 4.01 (s, 3H); ^{13}C NMR (125 MHz, $CDCl_3$) δ 168.4, 153.4, 147.8, 147.3, 131.5, 130.9, 130.5, 128.7, 128.0, 127.6, 124.3, 123.8, 120.8, 118.5, 114.9, 108.1, 107.9, 101.0, 71.5, 52.3; HRMS (ESI+) *m/z* calcd. for $C_{20}H_{16}NaO_5$: 359.0890; found: 359.0884.

Methyl (*E*)-2-[(2-methyl-3-phenylallyl)oxy]-1-naphthoate (1v):

1v was isolated by FC on silica gel using pentane/EtOAc 30:1; 83% yield; white solid; m.p. = 87.7 °C; IR (film) 2949, 1729, 1510, 1284, 1234, 1136, 748 cm^{-1}; ^{1}H NMR (500 MHz, $CDCl_3$)

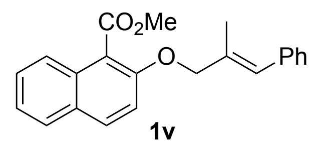
1v

δ7.89 (d, J = 9.0 Hz, 1H), 7.80 (d, J = 8.2 Hz, 1H), 7.77 (d, J = 8.5 Hz, 1H), 7.52 – 7.49 (m, 1H), 7.40 – 7.28 (m, 6H), 7.25 – 7.22 (m, 1H), 6.66 (s, 1H), 4.75 (s, 2H), 4.04 (s, 3H), 1.98 (s, 3H); ^{13}C NMR (125 MHz, $CDCl_3$) δ 168.4, 153.6, 137.1, 133.4, 131.5, 131.0, 128.9, 128.6, 128.1, 128.0, 127.5, 127.5, 126.6, 124.2, 123.8, 118.2, 114.6, 75.2, 52.3, 15.12; HRMS (ESI+) m/z calcd. for $C_{22}H_{20}NaO_3$: 335.1305; found: 335.1300.

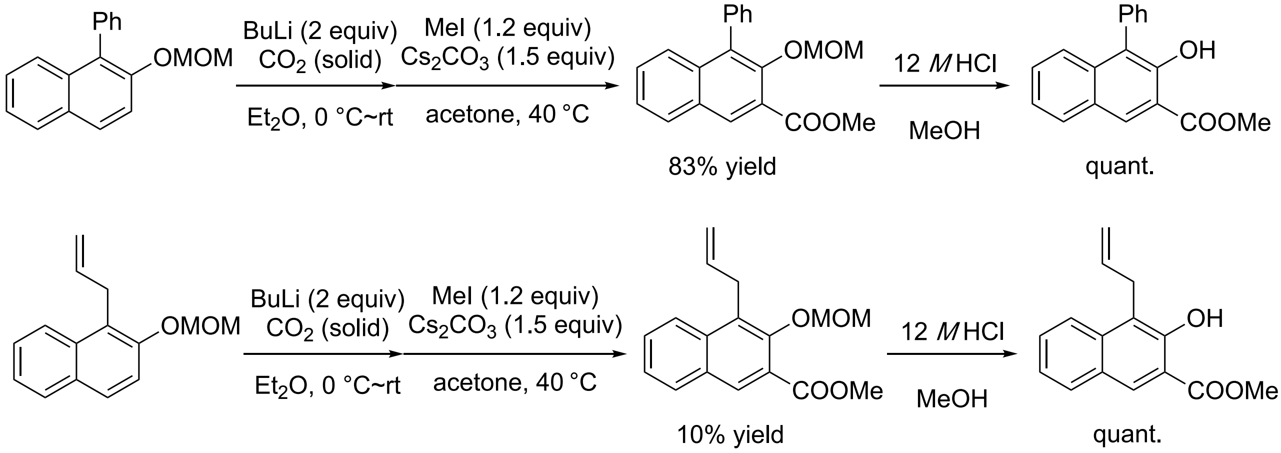

1-allylnaphthalen-2-ol[3] and 1-phenylnaphthalen-2-ol[4] were synthesized according to the literature. 3a and 3b were synthesized base on the literature[5] with a little modification, the modified procedure was shown as bellow.

To a solution of MOMO protected 1-allylnaphthalen-2-ol or 1-phenylnaphthalen-2-ol (5.00 mmol) in Et2O (20.0 mL) was added n-BuLi (6.25 mL, 10.0 mmol, 1.6M in hexane) at 0 oC. The reaction mixture was allowed to room temperature. After stirring for 1 h at room temperature, the reaction was quenched by dry ice (ca. 1 g) slowly, and then solvents were removed in vacuo. To the residue in Acetone (50.0mL) were added iodomethane (0.778 mL, 10.0 mmol) and Cs2CO3 (4.4 g, 12.5 mmol) at room temperature. After stirring for 2 h at 40 oC, the resulting mixture was poured into water and the aqueous layers were extracted with EtOAc (twice). The combined organic layers were washed with brine and dried over anhydrous MgSO4, then the solvents were removed in vacuo .The residue was purified by flash column chromatography on silica gel to give the corresponding ester.

To a solution of the corresponding ester intermidiate in MeOH (0.2 M) was added conc. HCl aq. (a few drops). The reaction mixture was warmed to 50 ºC. After stirring for 2 h, water was added to the resulting mixture, and the aqueous layers were extracted with EtOAc. The combined organic layers were washed with brine and dried over anhydrous MgSO4, then the solvents were removed

in vacuo. The residue was directly used in next step without further purification.

Then **3a** and **3b** could be synthesized by using the same procedure for the synthesis of methyl 2-allyloxy- and 2-Benzyloxy-1-naphthoates as we mentioned at the beginning of part 3.

Methyl (*E*)-4-phenyl-3-((3-(thiophen-3-yl)allyl)oxy)-2-naphthoate (3a):

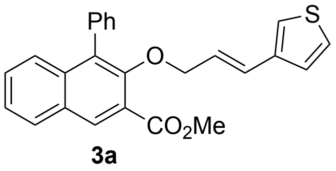

3a was isolated as yellow solid by FC on silica gel using pentane/EtOAc 30:1; 64% yield; yellow solid; m.p. = 75.3 °C; IR (neat) 2948, 1728, 1446, 1301, 1211, 965, 754 cm^{-1}; ^{1}H NMR (500 MHz, $CDCl_3$) δ 8.40 (s, 1H), 7.90 (dd, *J* = 6.5, 2.8 Hz, 1H), 7.58 – 7.43 (m, 8H), 7.21 (dd, *J* = 4.9, 3.0 Hz, 1H), 7.07 (d, *J* = 5.0 Hz, 1H), 7.03 (d, *J* = 2.6 Hz, 1H), 6.27 (d, *J* = 15.8 Hz, 1H), 5.79 (dt, *J* = 15.7, 6.4 Hz, 1H), 4.29 (d, *J* = 6.4 Hz, 2H), 3.97 (s, 3H); ^{13}C NMR (125 MHz, $CDCl_3$) δ 166.9, 151.8, 139.2, 135.6, 135.2, 133.1, 132.2, 131.1, 129.7, 128.9, 128.2, 128.1, 127.5, 127.2, 125.8, 125.5, 125.2, 125.1, 124.7, 122.4, 75.3, 52.4.; HRMS (ESI+) *m*/*z* calcd. for $C_{25}H_{20}NaO_3S$: 423.1025; found: 423.1017.

Methyl (*E*)-4-allyl-3-((3-(thiophen-3-yl)allyl)oxy)-2-naphthoate (3b):

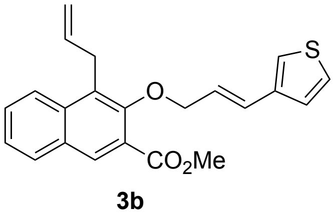

3b was isolated as yellow viscous liquid by FC on silica gel using pentane/EtOAc 30:1; 42% yield; yellow viscous liquid; IR (neat) 2948, 1726, 1446, 1219, 1159, 964, 752 cm^{-1}; ^{1}H NMR (500 MHz, $CDCl_3$) δ 8.32 (s, 1H), 7.96 (d, *J* = 8.5 Hz, 1H), 7.88 (d, *J* = 8.1 Hz, 1H), 7.57 (t, *J* = 7.6 Hz, 1H), 7.46 (t, *J* = 7.5 Hz, 1H), 7.29 – 7.24 (m, 2H), 7.20 (s, 1H), 6.74 (d, *J* = 15.8 Hz, 1H), 6.36 (dt, *J* = 15.8, 6.1 Hz, 1H), 6.10 (ddd, *J* = 22.6, 10.6, 5.6 Hz, 1H), 5.07 (d, *J* = 10.2 Hz, 1H), 4.97 (d, *J* = 17.2 Hz, 1H), 4.62 (d, *J* = 6.1 Hz, 2H), 3.96 (s, 3H); ^{13}C NMR (125 MHz, $CDCl_3$) δ 167.0, 152.4, 139.3, 136.7, 135.0, 131.9, 129.9, 129.5, 128.6, 128.2, 127.2, 126.0, 125.3, 125.1, 124.8, 124.6, 124.5, 122.7, 116.0, 75.9, 52.4, 29.8; HRMS (ESI+) *m*/*z* calcd. for $C_{22}H_{20}NaO_3S$: 387.1025; found: 387.1029.

4. General Procedure for Catalytic Asymmetric [1,3] Rearrangement of Methyl 2-Allyloxy- and 2-Benzyloxy-1-naphthoates

L8 (0.0165 mmol) and $Cu(OTf)_2$ (0.015 mmol) were stirred in 0.4 mL of DCM at room tempertaure for 0.5 h, then the reaction system was cooled to specific temperature. Naphthyl ethers **1** (0.15 mmol) was dissolved in 0.35 mL DCM and added to the system. The mixture was stirred at the same temperature until reaction completed. Then the solvent was removed, and the residue was purified by flash chromatography on silica gel (products **2s**, **2t**, **2u, 4a** and **4b** were purified using diol silica gel) to give the product **2**, which was then directly analyzed by HPLC to determine the enantiomeric excess.

5. Prospect for Appropriate Substrate and Optimization of the Reaction Conditions

As shown in Scheme S1, compounds **1** are appropriate substrates for the [1,3] rearrangement. 1-Methoxycarbonyl moiety of **1** was important for chelation with $Cu(OTf)_2$. 3-Aryl substituent on the allyl moiety of **1** was also important for [1,3] rearrangement because of the stability of the tight ion-pair transition state. Secondary allylic ether **1F** was decomposed to 2-hydroxy-1-naphthoate without giving **2F** under the same conditions.

Scheme S1: Prospect for appropriate substrastes[a]

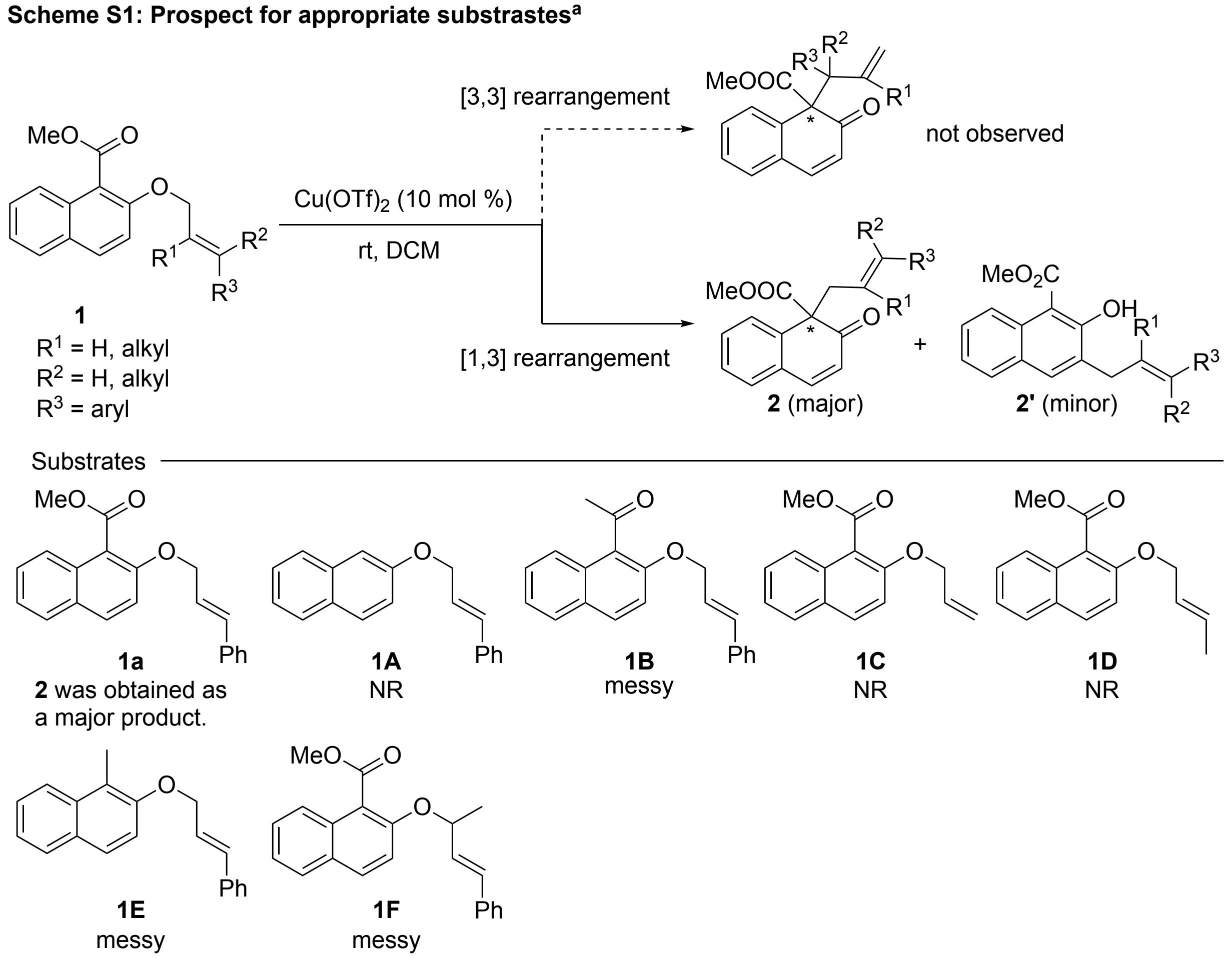

[a]All reactions were carried out with 0.15 mmol of **1** in 0.75 mL of DCM.

Table S1: Ligand screening[a]

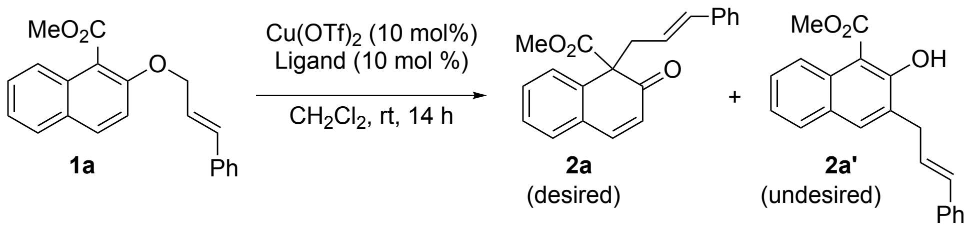

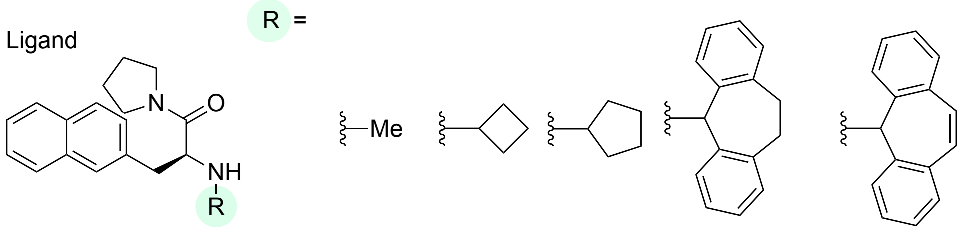

Conv.[b]	99%	99%	68%	38%	100%[d]
Ee of **2a**[c]	5% ee	2% ee	rac	45% ee	50% ee
**2a** : **2a'**[b]	67:33	72:28	65:35	68:22	21:79

[a]All reactions were carried out with 0.15 mmol of **1a** in 0.75 mL of DCM. [b]Based on NMR. [c]Determined by HPLC analysis. [d]The reaction was carried out at 30 °C.

As shown in Table S2, dichloromethane was the best solvent. 1,2-Dichloroethane was also available. However, Et_2O reduced the reactivity and the ee value. More polar solvents like ethers, esters and methanol were less effective because Lewis acidity of the catalyst was seriously decreased in polar solvents. In addition, the rearrangement did not occur in less polar toluene even at 30 °C. Judging from these experimental results, polarity of halogenated solvents may be effective to stabilize the tight ion pair transition state. Moreover, available solvents are also limited due to the solubility of ligands and substrates.

It was noted that this rearrangement more cleanly occurred in the presence of $Cu(OTf)_2$•**L11** or $Cu(OTf)_2$•**L8**, although $Cu(OTf)_2$ was active for this rearrangement (cf. Scheme S1). Moreover, **L11** was inferior to **L8** with regard to the enantioselectivity, regioselectivity (**2a**:**2a'**) and catalytic activity, probably due to some undesired steric hindrance of $Cu(OTf)_2$•**L11** (cf. Table 1).

Table S2: Solvent effect[a]

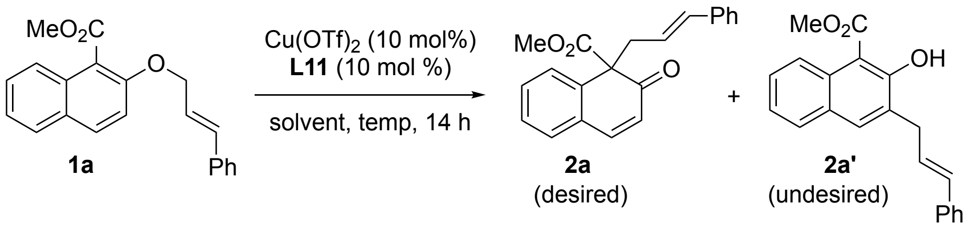

solvent	temp [°C]	conv. [%][b]	**2a** : **2a'**[b]	Ee [%][c]	Comments
toluene	30	0	–	–	
CH_2Cl_2	30	100	66 : 34	58	
CH_2Cl_2	–20	100	77 : 23	83	
$ClCH_2CH_2Cl$	–20	93	77 : 23	83	
Et_2O	30	34	59 : 41	35	**L11** was not dissolved.
THF	30	0	–	–	
EtOAc	30	0	–	–	
MeCN	30	0	–	–	
MeOH	30	0	–	–	**1a** was not dissolved.

[a]All reactions were carried out with 0.10 mmol of **1a** in 0.5 mL of solvent. [b]Based on NMR. [c]Determined by HPLC analysis.

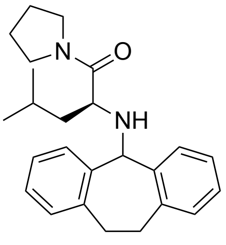

L11

6. Spectral Characterization Data for the Products

Methyl (*S*)-1-cinnamyl-2-oxo-1,2-dihydronaphthalene-1-carboxylate (2a):

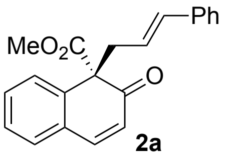

Yield (90%); colorless viscous liquid; IR (neat) 3026, 2950, 1742, 1663, 1222, 746 cm^{-1}; $[\alpha]^{26}_{D}$ = +111.9 (*c* 1.50, $CHCl_3$); ^{1}H NMR (500 MHz, $CDCl_3$) δ 7.46 – 7.43 (m, 2H), 7.38 – 7.34 (m, 3H), 7.20 – 7.17 (m, 2H), 7.16 – 7.14 (m, 1H), 7.09 – 7.07 (m, 2H), 6.20 – 6.17 (m, 2H), 5.68 – 5 .62 (m, 1H), 3.65 (s, 3H), 3.28 (ddd, *J* = 13.6, 8.1, 1.0 Hz, 1H), 3.14 (ddd, *J* = 13.6, 7.0, 1.3 Hz, 1H); ^{13}C NMR (125 MHz, $CDCl_3$) δ 197.4, 170.9, 146.0, 139.2, 136.9, 134.0, 130.4, 129.7, 129.5, 128.2, 128.1, 127.2, 126.9, 126.0, 125.2, 122.1, 62.6, 52.9, 43.9; HRMS (ESI+) *m*/*z* calcd. for $C_{21}H_{18}NaO_3$: 341.1148; found: 341.1154; The product was analyzed by HPLC to determine the enantiomeric excess: 91% ee (Chiralpak IA-3, *i*-propanol/hexane = 20/80, flow rate 1.0 mL/min, λ = 254 nm); t_r = 6.83 and 8.65 min.

Methyl (*S*,*E*)-2-oxo-1-[3-(*o*-tolyl)allyl]-1,2-dihydronaphthalene-1-carboxylate (2b):

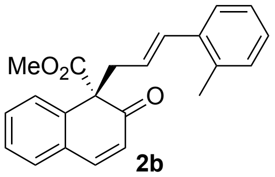

Yield (90%); colorless viscous liquid; $[\alpha]^{28}_{D}$ = +76.2 (*c* 0.84, $CHCl_3$); IR (neat) 3023, 2952, 1742, 1663, 1222, 967, 750 cm^{-1}; ^{1}H NMR (500 MHz, $CDCl_3$) δ 7.46-7.43 (m, 2H), 7.39 – 7.32 (m, 3H), 7.05 – 7.00 (d, *J* = 25.3 Hz, 4H), 6.34 (d, *J* = 15.6 Hz, 1H), 6.19 (d, *J* = 9.9 Hz, 1H), 5.51 (dt, *J* = 15.4, 7.6 Hz, 1H), 3.64 (s, 3H), 3.32 (dd, *J* = 13.5, 7.8 Hz, 1H), 3.17 (dd, *J* = 13.5, 7.4 Hz, 1H), 2.06 (s, 3H); ^{13}C NMR (125 MHz, $CDCl_3$) δ 197.4, 170.9, 146.1, 139.3, 136.3, 135.1, 132.5, 130.4, 129.9, 129.7, 129.6, 128.1, 127.2, 127.0, 125.8, 125.6, 125.3, 123.7, 62.8, 52.9, 44.2, 19.5; HRMS

(ESI+) *m/z* calcd. for $C_{22}H_{20}NaO_3$: 355.1305; found: 355.1306; The product was analyzed by HPLC to determine the enantiomeric excess: 95% ee (Chiralpak IA-3, *i*-propanol/hexane = 20/80, flow rate 1.0 mL/min, λ = 272 nm); t_r = 6.24 and 6.74 min.

Methyl (*S*,*E*)-2-oxo-1-[3-(*m*-tolyl)allyl]-1,2-dihydronaphthalene-1-carboxylate (2c):

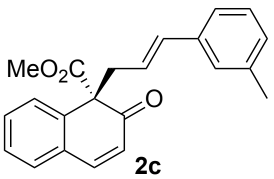

2c

Yield (91%); colorless viscous liquid; $[\alpha]^{28}_D$ = +93.8 (*c* 0.83, $CHCl_3$); IR (neat) 3024, 2951, 1743, 1663, 1223, 764 cm^{-1}; ^1H NMR (500 MHz, $CDCl_3$) δ 7.46 – 7.42 (m, 2H), 7.38 – 7.33 (m, 3H), 7.09 – 7.06 (m, 1H), 6.96 (d, *J* = 7.5 Hz, 1H), 6.90 – 6.98 (m, 2H), 6.19 (d, *J* = 9.9 Hz, 1H), 6.14 (d, *J* = 15.7 Hz, 1H), 5.64 (dt, *J* = 15.4, 7.6 Hz, 1H), 3.64 (s, 3H), 3.28 (dd, *J* = 13.6, 8.1 Hz, 1H), 3.13 (dd, *J* = 13.6, 7.1 Hz, 1H), 2.25 (s, 3H); ^{13}C NMR (125 MHz, $CDCl_3$) δ 197.4, 171.0, 146.0, 139.3, 137.8, 136.9, 134.2, 130.4, 129.7, 129.6, 128.2, 128.1, 128.0, 127.0, 126.9, 125.3, 123.1, 122.0, 62.8, 52.9, 44.0, 21.2; HRMS (ESI+) *m/z* calcd. for $C_{22}H_{20}NaO_3$: 355.1305; found: 355.1301. The product was analyzed by HPLC to determine the enantiomeric excess: 92% ee (Chiralpak IA-3, *i*-propanol/hexane = 20/80, flow rate 1.0 mL/min, λ = 254 nm); t_r = 6.56 and 8.02 min.

Methyl (*S*,*E*)-2-oxo-1-[3-(*p*-tolyl)allyl]-1,2-dihydronaphthalene-1-carboxylate (2d):

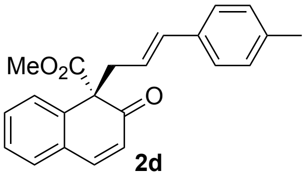

2d

Yield (87%); colorless viscous liquid; $[\alpha]^{28}_D$ = +93.1 (*c* 0.85, $CHCl_3$); IR (neat) 3034, 2951, 1742, 1664, 1223, 764 cm^{-1}; ^1H NMR (500 MHz, $CDCl_3$) δ 7.45 – 7.41 (m, 2H), 7.36 – 7.31 (m, 3H), 7.00 – 6.96 (m, 4H), 6.17 (d, *J* = 9.9 Hz, 1H), 6.14 (d, *J* = 15.8 Hz, 1H), 5.60 (dt, *J* = 15.5, 7.6 Hz, 1H), 3.63 (s, 3H), 3.27 (dd, *J* = 13.5, 8.1 Hz, 1H), 3.13 (dd, *J* = 13.5, 7.0 Hz, 1H), 2.25 (s, 3H); ^{13}C NMR (125 MHz, $CDCl_3$) δ 197.5, 171.0, 146.0, 139.3, 137.0, 134.2, 133.9, 130.3, 129.7, 129.6 129.0, 128.0, 126.9, 125.9, 125.3, 121.1, 62.8, 52.9, 44.0, 21.0; HRMS (ESI+) *m/z* calcd. for $C_{22}H_{20}NaO_3$: 355.1305; found: 355.1306. The product was analyzed by HPLC to determine the enantiomeric excess: 90% ee (Chiralpak IA-3, *i*-propanol/hexane = 20/80, flow rate 1.0 mL/min, λ = 254 nm); t_r = 6.67 and 10.29 min.

Methyl (*S*,*E*)-1-[3-(4-bromophenyl)allyl]-2-oxo-1,2-dihydronaphthalene-1-carboxylate (2e):

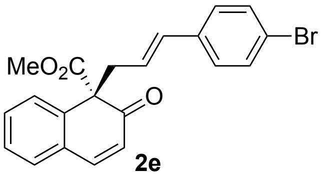

2e

Yield (76%); yellow viscous liquid; $[\alpha]^{29}_D$ = +98.1 (*c* 0.84, $CHCl_3$); IR (neat) 3026, 2951, 1742, 1663, 1487, 1223, 761 cm^{-1}; ^1H NMR (500 MHz, $CDCl_3$) δ 7.46 – 7.43 (m, 2H), 7.38 – 7.33 (m, 3H), 7.30 – 7.29 (m, 2H), 6.94 – 6.93 (m, 2H), 6.18 (d, *J* = 9.9 Hz, 1H), 6.11 (d, *J* = 15.7 Hz, 1H), 5.69 – 5.62 (m, 1H), 3.64 (s, 3H), ^1H 3.27 (ddd, *J* = 13.6, 8.1, 0.9 Hz, 1H), 3.11 (ddd, *J* = 13.6, 7.0, 1.2 Hz, 1H)); ^{13}C NMR (125 MHz, $CDCl_3$) δ 197.2, 170.8, 146.0, 139.1, 135.8, 132.9, 131.4, 130.4, 129.8, 129.5, 128.2, 127.6, 126.9, 125.2, 123.2, 121.0, 62.6, 52.9, 43.8; HRMS (ESI+) *m/z* calcd. for $C_{21}H_{17}BrNaO_3$: 419.0253; found: 419.0251. The product was analyzed by HPLC to

determine the enantiomeric excess: 91% ee (Chiralpak IA-3, *i*-propanol/hexane = 20/80, flow rate 1.0 mL/min, λ = 254 nm); t_r = 8.05 and 11.36 min.

Methyl (*S*,*E*)-1-[3-(4-chlorophenyl)allyl]-2-oxo-1,2-dihydronaphthalene-1-carboxylate (2f):

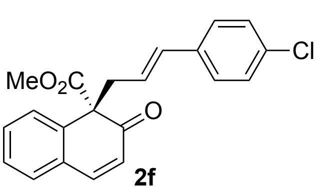

2f

Yield (78%); colorless viscous liquid; $[\alpha]^{29}_D$ = +113.7 (*c* 0.83, $CHCl_3$); IR (neat) 3038, 2952, 1742, 1663, 1224, 761 cm^{-1}; ^1H NMR (500 MHz, $CDCl_3$) δ 7.46 – 7.43 (m, 2H), 7.38 – 7.34 (m, 3H), 7.15 – 7.13 (m, 2H), 7.01 – 6.99 (m, 2H), 6.18 (d, *J* = 9.9 Hz, 1H), 6.13 (d, *J* = 15.8 Hz, 1H), 5.67-5.61 (m, 1H), 3.64 (s, 3H), 3.27 (ddd, *J* = 13.6, 8.1, 1.0 Hz, 1H), 3.11 (ddd, *J* = 13.6, 7.0, 1.3 Hz, 1H); ^{13}C NMR (125 MHz, $CDCl_3$) δ 197.3, 170.8, 146.0, 139.2, 135.4, 132.9, 130.4, 129.8, 129.5, 128.5, 128.2, 127.2, 126.9, 125.3, 123.1, 62.7, 52.9, 43.8; HRMS (ESI+) *m*/*z* calcd. for $C_{21}H_{17}ClNaO_3$: 375.0758; found: 375.0760. The product was analyzed by HPLC to determine the enantiomeric excess: 92% ee (Chiralpak IA-3, *i*-propanol/hexane = 20/80, flow rate 1.0 mL/min, λ = 254 nm); t_r = 7.78 and 10.73 min.

Methyl (*S*,*E*)-1-[3-(naphthalen-1-yl)allyl]-2-oxo-1,2-dihydronaphthalene-1-carboxylate (2g):

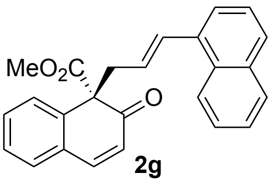

2g

Yield (95%); colorless viscous liquid; $[\alpha]^{30}_D$ = +40.5 (*c* 0.84, $CHCl_3$); IR (neat) 3038, 2951, 1742, 1662, 1224, 761 cm^{-1}; ^1H NMR (500 MHz, $CDCl_3$) δ 7.77 – 7.75 (m, 1H), 7.67 (d, *J* = 8.2 Hz, 1H), 7.63 (d, *J* = 8.2 Hz, 1H), 7.50 – 7.47 (m, 1H), 7.44 – 7.29 (m, 7H), 7.20 (d, *J* = 7.1 Hz, 1H), 6.85 (d, *J* = 15.5 Hz, 1H), 6.21 (d, *J* = 9.9 Hz, 1H), 5.66 (dt, *J* = 15.3, 7.6 Hz, 1H), 3.67 (s, 3H), 3.42 (dd, *J* = 13.4, 7.8 Hz, 1H), 3.26 (dd, *J* = 13.4, 7.5 Hz, 1H); ^{13}C NMR (125 MHz, $CDCl_3$) δ 197.5, 170.9, 146.1, 139.4, 135.0, 133.3, 132.1, 130.9, 130.5, 129.8, 129.7, 128.2, 128.1, 127.6, 127.0, 125.8, 125.6, 125.5, 125.4, 125.3, 123.9, 123.7, 62.8, 53.0, 44.3; HRMS (ESI+) *m*/*z* calcd. for $C_{25}H_{20}NaO_3$: 391.1305; found: 391.1310. The product was analyzed by HPLC to determine the enantiomeric excess: 92% ee (Chiralpak IA-3, *i*-propanol/hexane = 20/80, flow rate 1.0 mL/min, λ = 254 nm); t_r = 7.60 and 8.35 min.

Methyl (*S*,*E*)-1-[3-(naphthalen-2-yl)allyl]-2-oxo-1,2-dihydronaphthalene-1-carboxylate (2h):

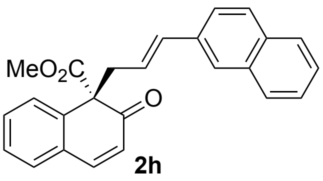

2h

Yield (86%); colorless viscous liquid; $[\alpha]^{20}_D$ = +120.0 (*c* 0.85, $CHCl_3$); IR (neat) 3022, 2951, 1742, 1662, 1224, 757 cm^{-1}; ^1H NMR (500 MHz, $CDCl_3$) δ 7.71 – 7.68 (m, 2H), 7.63 (d, *J* = 8.6 Hz, 1H), 7.46 – 7.31 (m, 8H), 7.26 (dd, *J* = 8.6, 1.5 Hz, 1H), 6.33 (d, *J* = 15.7 Hz, 1H), 6.19 (d, *J* = 9.9 Hz, 1H), 5.79 (dt, *J* = 15.4, 7.6 Hz, 1H), 3.64 (s, 3H), 3.34 (dd, *J* = 13.6, 8.1 Hz, 1H), 3.18 (dd, *J* = 13.6, 7.1 Hz, 1H); ^{13}C NMR (125 MHz, $CDCl_3$) δ 197.4, 171.0, 146.0, 139.3, 134.4, 134.2, 133.4, 132.7, 130.4, 129.8, 129.6, 128.1, 127.9, 127.8, 127.5, 127.0, 126.1, 125.8, 125.7, 125.3, 123.4, 122.7, 62.8, 52.9, 44.11; HRMS (ESI+) *m*/*z* calcd. for $C_{25}H_{20}NaO_3$: 391.1305; found:

391.1303. The product was analyzed by HPLC to determine the enantiomeric excess: 92% ee (Chiralpak IA-3, *i*-propanol/hexane = 20/80, flow rate 1.0 mL/min, λ = 254 nm); t_r = 8.77 and 13.43 min.

Methyl (*S*,*E*)-1-[3-(furan-3-yl)allyl]-2-oxo-1,2-dihydronaphthalene-1-carboxylate (2i):

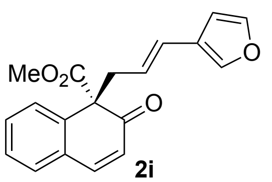

2i

Yield (63%); colorless viscous liquid; $[\alpha]^{27}_{D}$ = +59.4 (*c* 0.95, $CHCl_3$); IR (neat) 3026, 1742, 1661, 1225, 759 cm^{-1}; ^1H NMR (500 MHz, $CDCl_3$) δ 7.46 – 7.42 (m, 2H), 7.38 – 7.34 (m, 3H), 7.23 – 7.19 (m, 2H), 6.22 (d, *J* = 1.6 Hz, 1H), 6.19 (d, *J* = 9.9 Hz, 1H), 6.03 (d, *J* = 15.7 Hz, 1H), 5.40 – 5.34 (m, 1H), 3.64 (s, 3H), 3.23 (ddd, *J* = 13.6, 8.2, 0.9 Hz, 1H), 3.08 (ddd, *J* = 13.6, 7.0, 1.2 Hz, 1H); ^{13}C NMR (125 MHz, $CDCl_3$) δ 197.4, 171.0, 146.0, 143.1, 140.0, 139.3, 130.4, 129.7, 129.6, 128.1, 127.0, 125.4, 123.8, 121.8, 107.4, 62.8, 52.9, 43.8; HRMS (ESI+) *m/z* calcd. for $C_{19}H_{16}NaO_4$: 331.0941; found: 331.0950. The product was analyzed by HPLC to determine the enantiomeric excess: 96% ee (Chiralpak IA-3, *i*-propanol/hexane = 20/80, flow rate 1.0 mL/min, λ = 254 nm); t_r = 7.69 and 10.62 min.

Methyl (*S*,*E*)-2-oxo-1-[3-(thiophen-3-yl)allyl]-1,2-dihydronaphthalene-1-carboxylate (2j):

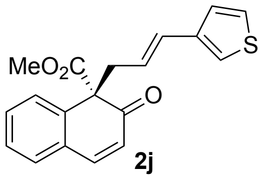

2j

Yield (85%); colorless liquid; $[\alpha]^{27}_{D}$ = +107.1 (*c* 1.14, $CHCl_3$); IR (neat) 2959, 1741, 1661, 1224, 765 cm^{-1}; ^1H NMR (500 MHz, $CDCl_3$) δ 7.45 – 7.42 (m, 2H), 7.38 – 7.34 (m, 3H), 7.13 – 7.11 (m, 1H), 6.90 – 6.89 (m, 2H), 6.20 – 6.17 (m, 2H), 5.53–5.47 (m, 1H), 3.63 (s, 3H), 3.25 (dd, *J* = 13.6, 8.2 Hz, 1H), 3.10 (ddd, *J* = 13.6, 7.0, 1.1 Hz, 1H); ^{13}C NMR (125 MHz, $CDCl_3$) δ 197.4, 170.9, 146.0, 139.6, 139.3, 130.4, 129.7, 129.6, 128.3, 128.1, 126.9, 125.6, 125.3, 124.9, 122.1, 121.5, 62.7, 52.9, 43.8; HRMS (ESI+) *m/z* calcd. for $C_{19}H_{16}NaO_3S$: 347.0712; found: 347.0720. The product was analyzed by HPLC to determine the enantiomeric excess: 94% ee (Chiralpak IA-3, *i*-propanol/hexane = 20/80, flow rate 1.0 mL/min, λ = 254 nm); t_r = 8.48 and 13.16 min.

Methyl (*S*,*E*)-2-oxo-1-[3-phenylbut-2-en-1-yl]-1,2-dihydronaphthalene-1-carboxylate (2k):

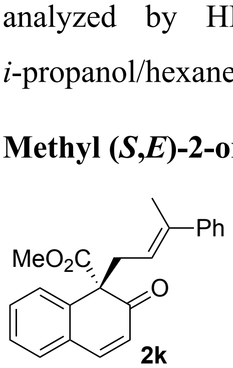

2k

Yield (92%); colorless viscous liquid; $[\alpha]^{28}_{D}$ = +81.0 (*c* 0.84, $CHCl_3$); IR (neat) 3022, 2951, 1743, 1663, 1224, 760 cm^{-1}; ^1H NMR (500 MHz, $CDCl_3$) δ 7.48 (d, *J* = 9.9 Hz, 1H), 7.42 – 7.39 (m, 1H), 7.36 – 7.34 (m, 3H), 7.21 – 7.13 (m, 3H), 7.08 – 7.06 (m, 2H), 6.22 (d, *J* = 9.9 Hz, 1H), 5.25 – 5.20 (m, 1H), 3.65 (s, 3H), 3.34 (dd, *J* = 14.2, 8.2 Hz, 1H), 3.14 (dd, *J* = 14.2, 7.2 Hz, 1H), 1.77 (s, 3H); ^{13}C NMR (125 MHz, $CDCl_3$) δ 197.6, 171.2, 146.0, 143.6, 139.5, 138.9, 130.3, 129.7, 128.1, 128.0, 127.7, 127.1, 126.7, 125.7, 125.3, 119.9, 62.6, 52.9, 39.7, 16.0; HRMS (ESI+) *m/z* calcd. for $C_{22}H_{20}NaO_3$: 355.1310; found: 355.12306. The product was analyzed by HPLC to determine the enantiomeric excess: 94% ee (Chiralpak AD-3, *i*-propanol/hexane = 20/80, flow rate 1.0 mL/min, λ = 254 nm); t_r = 6.67 and

9.22 min.

Methyl (*S*)-1-[3,3-bis(4-chlorophenyl)allyl]-2-oxo-1,2-dihydronaphthalene-1-carboxylate (2l):

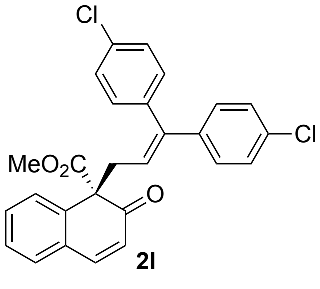

Yield (89%); white solid; m.p. = 99.5 °C; $[\alpha]^{24}_{D}$ = +109.16 (*c* 0.85, $CHCl_3$); IR (neat) 3027, 2952, 1743, 1664, 1490, 1224, 1092, 830, 759 cm^{-1}; ^{1}H NMR (500 MHz, $CDCl_3$) δ 7.55 (d, *J* = 9.9 Hz, 1H), 7.40–7.30 (m, 3H), 7.27 – 7.25 (m, 2H), 7.13 – 7.12 (m, 2H), 7.06 (d, *J* = 7.5 Hz, 1H), 6.82 (d, *J* = 8.4 Hz, 2H), 6.73 (d, *J* = 8.2 Hz, 2H), 6.27 (d, *J* = 9.9 Hz, 1H), 5.65 (t, *J* = 7.6 Hz, 1H), 3.60 (s, 3H), 3.20 (dd, *J* = 14.3, 7.0 Hz, 1H), 3.03 (dd, *J* = 14.3, 8.3 Hz, 1H); ^{13}C NMR (125 MHz, $CDCl_3$) δ 197.1, 170.8, 146.2, 143.0, 140.2, 139.1, 137.0, 133.2, 133.2, 131.0, 130.6, 129.7, 129.4, 128.5, 128.4, 128.2, 128.2, 127.1, 125.2, 122.5, 62.5, 53.0, 40.2; HRMS (ESI+) *m*/*z* calcd. for $C_{27}H_{20}Cl_2NaO_3$: 485.0682; found: 485.0678. The product was analyzed by HPLC to determine the enantiomeric excess: 90% ee (Chiralpak AD-3, *i*-propanol/hexane = 20/80, flow rate 1.0 mL/min, λ = 254 nm); t_r = 8.02 and 10.86 min.

Methyl (*S*)-1-(3-methylbut-2-en-1-yl)-2-oxo-1,2-dihydronaphthalene-1-carboxylate (2m):

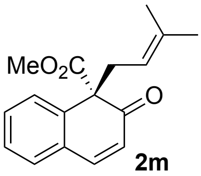

Yield (58%); colorless liquid; $[\alpha]^{29}_{D}$ = –3.8 (*c* 0.83, $CHCl_3$); IR (neat) 2952, 2913, 1742, 1664, 1227, 828, 762 cm^{-1}; ^{1}H NMR (500 MHz, $CDCl_3$) δ 7.47 (d, *J* = 9.9 Hz, 1H), 7.41 – 7.38 (m, 1H), 7.35 –7 .34 (m, 2H), 7.30 (d, *J* = 7.6 Hz, 1H), 6.19 (d, *J* = 9.9 Hz, 1H), 4.64 (tdd, *J* = 7.0, 2.8, 1.4 Hz, 1H), 3.62 (s, 3H), 3.14 (dd, *J* = 14.1, 8.1 Hz, 1H), 2.91 (dd, *J* = 14.1, 7.0 Hz, 1H), 1.47 (s, 3H), 1.35 (s, 3H); ^{13}C NMR (125 MHz, $CDCl_3$) δ 197.8, 171.3, 145.8, 139.7, 135.9, 130.2, 129.7, 129.5, 127.8, 127.0, 125.4, 116.2, 62.6, 52.8, 39.2, 25.6, 17.7; HRMS (ESI+) *m*/*z* calcd. for $C_{17}H_{18}NaO_3$: 293.1148; found: 293.1152. The product was analyzed by HPLC to determine the enantiomeric excess: 68% ee (Chiralpak AS-3, *i*-propanol/hexane = 20/80, flow rate 1.0 mL/min, λ = 290 nm); t_r = 5.63 and 6.08 min.

Methyl (*S*)-6-bromo-1-cinnamyl-2-oxo-1,2-dihydronaphthalene-1-carboxylate (2n):

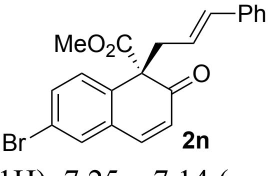

Yield (95%); yellow viscous liquid; $[\alpha]^{26}_{D}$ = +115.7 (*c* 0.84, $CHCl_3$); IR (neat) 3027, 2950, 1743, 1667, 1224, 748 cm^{-1}; ^{1}H NMR (500 MHz, $CDCl_3$) δ 7.56 (dd, *J* = 8.3, 1.8 Hz, 1H), 7.49 (d, *J* = 1.6 Hz, 1H), 7.36 (d, *J* = 9.9 Hz, 1H), 7.25 – 7.14 (m, 4H), 7.11 – 7.09 (m, 2H), 6.23 – 6.19 (m, 2H), 5.64 (dt, *J* = 15.4, 7.6 Hz, 1H), 3.64 (s, 3H), 3.28 (dd, *J* = 13.6, 8.2 Hz, 1H), 3.11 (dd, *J* = 13.6, 7.0 Hz, 1H); ^{13}C NMR (125 MHz, $CDCl_3$) δ 196.7, 170.4, 144.3, 137.8, 136.7, 134.4, 133.1, 132.2, 131.4, 128.6, 128.3, 127.4, 126.4, 126.1, 121.9, 121.7, 62.4, 53.1, 43.6; HRMS (ESI+) *m*/*z* calcd. for $C_{21}H_{17}BrNaO_3$: 419.0253; found: 419.0251. The product was analyzed by HPLC to determine the enantiomeric excess: 93% ee (Chiralpak IA-3, *i*-propanol/hexane = 20/80, flow rate 1.0 mL/min, λ = 254 nm); t_r = 7.32 and 9.04

min.

Methyl (*S*)-3-bromo-1-cinnamyl-2-oxo-1,2-dihydronaphthalene-1-carboxylate (2o):

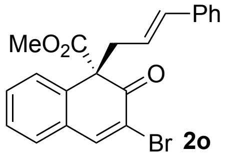

2o

Yield (95%); yellow viscous liquid; $[\alpha]^{27}_{D}$ = +0.9 (*c* 0.84, $CHCl_3$); IR (neat) 3027, 2952, 1745, 1675, 1229, 748 cm^{-1}; ^1H NMR (500 MHz, $CDCl_3$) δ 7.87 (s, 1H), 7.48 – 7.45 (m, 1H), 7.38 – 7.35 (m, 2H), 7.29 (d, *J* = 7.4 Hz, 1H), 7.20 – 7.12 (m, 3H), 7.09–7.07 (m, 2H), 6.19 (d, *J* = 15.7 Hz, 1H), 5.60 (dt, *J* = 15.5, 7.6 Hz, 1H), 3.63 (s, 3H), 3.31 (dd, *J* = 13.6, 8.1 Hz, 1H), 3.15 (ddd, *J* = 13.5, 7.1, 0.7 Hz, 1H); ^{13}C NMR (125 MHz, $CDCl_3$) δ 190.4, 170.2, 147.3, 138.6, 136.8, 134.7, 130.7, 130.0, 129.3, 128.4, 128.3, 127.4, 126.9, 126.1, 121.5, 121.4, 64.1, 53.1, 44.3; HRMS (ESI+) *m*/*z* calcd. for $C_{21}H_{17}BrNaO_3$: 419.0253; found: 419.0253. The product was analyzed by HPLC to determine the enantiomeric excess: 81% ee (Chiralpak IA-3, *i*-propanol/hexane = 20/80, flow rate 1.0 mL/min, λ = 254 nm); t_r = 7.91 and 16.81 min.

Benzyl (*S*)-1-cinnamyl-2-oxo-1,2-dihydronaphthalene-1-carboxylate (2p):

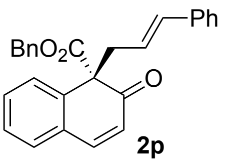

2p

Yield (82%); colorless viscous liquid; $[\alpha]^{28}_{D}$ = +32.5 (*c* 0.85, $CHCl_3$); IR (neat) 3029, 1742, 1663, 1214, 744 cm^{-1}; ^1H NMR (500 MHz, $CDCl_3$) δ 7.43 (d, *J* = 9.9 Hz, 1H), 7.40 – 7.32 (m, 3H), 7.29 – 7.24 (m, 4H), 7.20 – 7.12 (m, 3H), 7.08 – 7.07 (m, 4H), 6.20 – 6.17 (m, 2H), 5.69 – 5.63 (m, 1H), 5.14 – 5.08 (m, 2H), 3.31 (dd, *J* = 13.6, 8.1 Hz, 1H), 3.14 (dd, *J* = 13.7, 7.0 Hz, 1H); ^{13}C NMR (125 MHz, $CDCl_3$) δ 197.3, 170.1, 146.0, 139.1, 137.0, 135.4, 134.1, 130.3, 129.7, 129.6, 128.3, 128.1, 127.9, 127.4, 127.2, 127.0, 126.1, 125.3, 122.3, 67.1, 62.9, 43.7; HRMS (ESI+) *m*/*z* calcd. for $C_{27}H_{22}NaO_3$: 417.1461; found: 417.1462. The product was analyzed by HPLC to determine the enantiomeric excess: 89% ee (Chiralpak AD-3, *i*-propanol/hexane = 20/80, flow rate 1.0 mL/min, λ = 254 nm); t_r = 13.4 and 15.15 min.

Methyl (*S*)-1-cinnamyl-2-oxo-6-(*p*-tolyl)-1,2-dihydronaphthalene-1-carboxylate (2q):

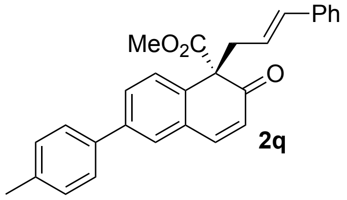

2q

Yield (95%); colorless viscous liquid; $[\alpha]^{31}_{D}$ = +120.4 (*c* 0.84, $CHCl_3$); IR (neat) 3034, 2951, 1742, 1665, 1234, 815, 751 cm^{-1};^1H NMR (500 MHz, $CDCl_3$) δ 7.65 (dd, *J* = 8.1, 1.8 Hz, 1H), 7.54 – 7.49 (m, 4H), 7.42 (d, *J* = 8.1 Hz, 1H), 7.28 – 7.27 (m, 2H), 7.19 – 7.09 (m, 5H), 6.25 – 6.21 (m, 2H), 5.70 (dt, *J* = 15.3, 7.5 Hz, 1H), 3.66 (s, 3H), 3.31 (dd, *J* = 13.6, 8.2 Hz, 1H), 3.18 (dd, *J* = 13.6, 6.9 Hz, 1H), 2.41 (s, 3H); ^{13}C NMR (125 MHz, $CDCl_3$) δ 197.4, 170.9, 146.0, 141.0, 137.8, 137.6, 137.0, 136.6, 134.1, 130.0, 129.6, 128.7, 128.3, 128.0, 127.4, 127.2, 126.8, 126.1, 125.6, 122.3, 62.5, 52.9, 43.8, 21.1; HRMS (ESI+) *m*/*z* calcd. for $C_{28}H_{24}NaO_3$: 431.1618; found: 431.1611. The product was analyzed by HPLC to determine the enantiomeric excess: 93% ee (Chiralpak AD-3, *i*-propanol/hexane = 20/80, flow rate 1.0 mL/min, λ = 254 nm); t_r = 8.93 and

9.76 min.

Methyl (*S*)-1-cinnamyl-2-oxo-6-(phenylethynyl)-1,2-dihydronaphthalene-1-carboxylate (2r):

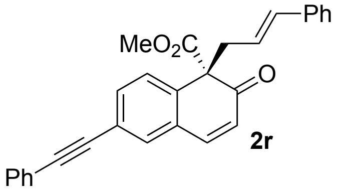

2r

Yield (94%); colorless viscous liquid; $[\alpha]^{30}_{D}$ = +226.6 (*c* 0.84, $CHCl_3$); IR (neat) 3027, 2947, 1743, 1666, 1222, 757 cm^{-1}; ^1H NMR (500 MHz, $CDCl_3$) δ 7.58 (dd, *J* = 8.0, 1.6 Hz, 1H), 7.54 – 7.50 (m, 3H), 7.41 (d, *J* = 9.9 Hz, 1H), 7.36 – 7.34 (m, 4H), 7.20 – 7.15 (m, 2H), 7.15 – 7.09 (m, 3H), 6.23 – 6.20 (m, 2H), 5.66 (dt, *J* = 15.5, 7.6 Hz, 1H), 3.64 (s, 3H), 3.30 (dd, *J* = 13.6, 8.2 Hz, 1H), 3.15 (dd, *J* = 13.6, 7.0 Hz, 1H); ^{13}C NMR (125 MHz, $CDCl_3$) δ 196.9, 170.6, 145.1, 138.9, 136.8, 134.3, 133.1, 132.5, 131.6, 129.8, 128.6, 128.4, 128.3, 127.3, 127.1, 126.1, 126.0, 123.4, 122.6, 121.9, 90.8, 87.9, 62.7, 53.0, 43.7; HRMS (ESI+) *m/z* calcd. for $C_{29}H_{22}NaO_3$: 441.1461; found: 441.1454. The product was analyzed by HPLC to determine the enantiomeric excess: 93% ee (Chiralpak IA-3, *i*-propanol/hexane = 20/80, flow rate 1.0 mL/min, λ = 254 nm); t_r = 8.71 and 9.98 min.

Methyl (*S*)-1-(3,4-dimethoxybenzyl)-2-oxo-1,2-dihydronaphthalene-1-carboxylate (2s):

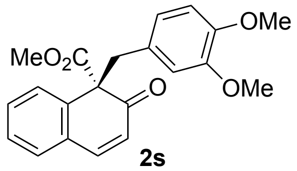

2s

Yield (96%); light yellow solid; m.p. = 140.1 °C; $[\alpha]^{25}_{D}$ = +96.1 (*c* 0.61, $CHCl_3$); IR (neat) 2953, 2841, 1742, 1661, 1516, 1236, 1028, 763 cm^{-1}; ^1H NMR (500 MHz, $CDCl_3$) δ 7.50–7.44 (m, 2H), 7.35 (t, *J* = 7.3 Hz, 1H), 7.20 (d, *J* = 7.5 Hz, 1H), 7.11 (d, *J* = 9.9 Hz, 1H), 6.50 (d, *J* = 8.2 Hz, 1H), 6.25 (dd, *J* = 8.2, 1.9 Hz, 1H), 5.93–5.91 (m, 2H), 3.74 (s, 3H), 3.68 (d, *J* = 13.5 Hz, 1H), 3.66 (s, 3H), 3.47 (s, 3H), 3.46 (d, *J* = 13.5 Hz, 1H); ^{13}C NMR (125 MHz, $CDCl_3$) δ 197.8, 171.1, 147.6, 145.6, 139.6, 130.1, 130.0, 129.6, 128.0, 127.2, 126.6, 125.5, 122.1, 112.5, 110.2, 63.6, 55.5, 55.3, 52.9, 46.3; HRMS (ESI+) *m/z* calcd. for $C_{21}H_{20}NaO_5$: 375.1203; found: 375.1201. The product was analyzed by HPLC to determine the enantiomeric excess: 94% ee (Chiralpak AS-3, *i*-propanol/hexane = 20/80, flow rate 1.0 mL/min, λ = 300 nm); t_r = 10.79 and 18.18 min.

Methyl (*S*)-1-(4-methoxybenzyl)-2-oxo-1,2-dihydronaphthalene-1-carboxylate (2t):

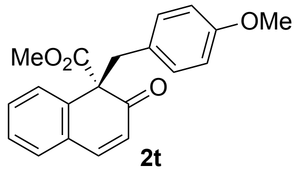

2t

Yield (92%); yellow solid; m.p. = 88.0 °C; $[\alpha]^{25}_{D}$ = +87.8 (*c* 0.61, $CHCl_3$); IR (film) 2952, 2837, 1742, 1661, 1513, 1246, 1034, 754 cm^{-1}; ^1H NMR (500 MHz, $CDCl_3$) δ 7.48 – 7.42 (m, 2H), 7.34 (t, *J* = 8.0 Hz, 1H), 7.18 (d, *J* = 7.5 Hz, 1H), 7.11 (d, *J* = 10.0 Hz, 1H), 6.50 – 6.46 (m, 4H), 5.93 (d, *J* = 10.0 Hz, 1H), 3.68 (d, *J* = 13.5 Hz, 1H), 3.66 (s, 3H), 3.65 (s, 3H), 3.46 (d, *J* = 13.5 Hz, 1H); ^{13}C NMR (125 MHz, $CDCl_3$) δ 197.9, 171.2, 158.3, 145.7, 139.5, 130.7, 130.1, 130.0, 129.5, 128.0, 127.2, 126.2, 125.4, 112.9, 63.6, 55.0, 52.9, 45.8; HRMS (ESI+) *m/z* calcd. for $C_{20}H_{18}NaO_4$: 345.1097; found: 345.1094. The product was analyzed by HPLC to determine the enantiomeric excess: 88% ee (Chiralpak AD-3, *i*-propanol/hexane = 20/80, flow rate 1.0 mL/min, λ = 254 nm); t_r

= 8.06 and 8.99 min.

Methyl (*S*)-1-(benzo[d][1,3]dioxol-5-ylmethyl)-2-oxo-1,2-dihydronaphthalene-1-carboxylate (2u):

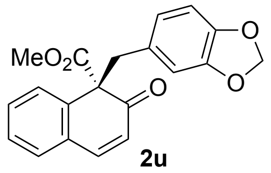

Yield (95%); yellow solid; m.p. = 131.3 °C; $[\alpha]^{23}_{D}$ = +110.4 (*c* 0.61, $CHCl_3$); IR (film) 2938, 2890, 1742, 1658, 1487, 1443, 1224, 1038, 930 cm^{-1}; ^1H NMR (500 MHz, $CDCl_3$) δ 7.47 (t, *J* = 7.5 Hz, 1H), 7.41 (d, *J* = 7.2 Hz, 1H), 7.35 (t, *J* = 7.2 Hz, 1H), 7.22 (d, *J* = 7.5 Hz, 1H), 7.17 (d, *J* = 10.0 Hz, 1H), 6.42 (d, *J* = 8.0 Hz, 1H), 6.07 (dd, *J* = 8.0, 1.7 Hz, 1H), 6.03 (d, *J* = 1.7 Hz, 1H), 5.97 (d, *J* = 10.0 Hz, 1H), 5.79 (s, 2H), 3.66 (d, *J* = 13.5 Hz, 1H), 3.65 (s, 3H), 3.43 (d, *J* = 13.5 Hz, 1H); ^{13}C NMR (125 MHz, $CDCl_3$) δ 197.7, 171.1, 146.7, 146.2, 145.7, 139.3, 130.2, 129.9, 129.6, 128.2, 127.9, 127.1, 125.4, 123.0, 110.0, 107.5, 100.6, 63.5, 52.9, 46.2; HRMS (ESI+) *m*/*z* calcd. for $C_{20}H_{16}NaO_5$: 359.0890; found: 359.0883. The product was analyzed by HPLC to determine the enantiomeric excess: 93% ee (Chiralpak AD-3, *i*-propanol/hexane = 20/80, flow rate 1.0 mL/min, λ = 300 nm); t_r = 9.42 and 12.12 min.

Methyl (*S*,*E*)-1-(2-methyl-3-phenylallyl)-2-oxo-1,2-dihydronaphthalene-1-carboxylate (2v):

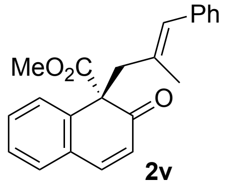

Yield (71%); colorless viscous liquid; $[\alpha]^{25}_{D}$ = +40.5 (*c* 0.84, $CHCl_3$); IR (neat) 3022, 2951, 1742, 1663, 1223, 762 cm^{-1}; ^1H NMR (500 MHz, $CDCl_3$) δ 7.47 – 7.42 (m, 2H), 7.40 – 7.35 (m, 3H), 7.22 – 7.18 (m, 2H), 7.13 – 7.10 (m, 1H), 6.90 – 6.88 (m, 2H), 6.22 (d, *J* = 9.9 Hz, 1H), 5.89 (s, 1H), 3.63 (s, 3H), 3.39 (d, *J* = 13.3 Hz, 1H), 3.20 (d, *J* = 13.3 Hz, 1H), 1.36 (s, 3H); ^{13}C NMR (125 MHz, $CDCl_3$) δ 197.8, 171.2, 145.9, 139.6, 137.7, 132.2, 130.2, 129.74, 129.72, 128.6, 128.1, 127.8, 127.4, 126.1, 125.7, 62.8, 53.0, 50.5, 19.3; HRMS (ESI+) *m*/*z* calcd. for $C_{22}H_{20}NaO_3$: 335.1305; found: 335.1308. The product was analyzed by HPLC to determine the enantiomeric excess: 88% ee (Chiralpak AS-3, *i*-propanol/hexane = 20/80, flow rate 1.0 mL/min, λ = 254 nm); t_r = 6.81 and 8.00 min.

Methyl (+)-(*R*,*E*)-3-oxo-4-phenyl-4-(3-(thiophen-3-yl)allyl)-3,4-dihydronaphthalene-2-carboxylate (4a):

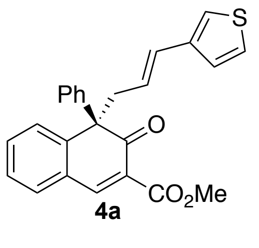

Yield (52%); light yellow viscous liquid; $[\alpha]^{23}_{D}$ = +61.7 (*c* 1.20, $CHCl_3$); IR (neat) 3020, 2949, 1734, 1216, 756 cm^{-1}; ^1H NMR (500 MHz, $CDCl_3$) δ 8.18 (s, 1H), 7.50 (t, *J* = 6.8 Hz, 2H), 7.39 (t, *J* = 7.5 Hz, 1H), 7.30 (d, *J* = 7.9 Hz, 1H), 7.26 – 7.21 (m, 3H), 7.12 (dd, *J* = 4.9, 3.0 Hz, 1H), 7.07 (d, *J* = 7.0 Hz, 2H), 6.91 (dd, *J* = 7.7, 3.7 Hz, 2H), 6.34 (d, *J* = 15.8 Hz, 1H), 5.74 – 5.52 (m, 1H), 3.81 (s, 3H), 3.77 (dd, *J* = 13.9, 7.3 Hz, 1H), 3.06 (dd, *J* = 13.6, 7.3 Hz, 1H); ^{13}C NMR (125 MHz, $CDCl_3$) δ 196.3, 165.0, 150.5, 145.8, 141.9, 139.7, 131.9, 131.5, 129.6, 129.3, 128.6, 128.1, 127.6, 127.4, 125.7, 125.6, 124.9, 124.3, 121.3, 61.6, 52.3, 42.4; HRMS (ESI+) *m*/*z* calcd. for

$C_{25}H_{20}NaO_3S$: 423.1025; found: 423.1026; The product was analyzed by HPLC to determine the enantiomeric excess: 92% ee (Chiralpak AS-3, *i*-propanol/hexane = 20/80, flow rate 1.0 mL/min, λ = 254 nm); t_r = 11.08 and 14.26 min. The absolute configuration of **4a** was speculated based on the proposed transition state shown in Figure 1b.

Methyl (–)-(*S*,*E*)-4-allyl-3-oxo-4-(3-(thiophen-3-yl)allyl)-3,4-dihydronaphthalene-2-carboxylate (4b):

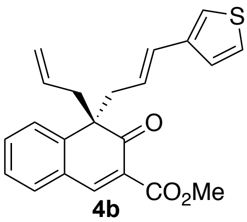

4b

Yield (56%); light yellow viscous liquid; $[\alpha]^{22}_D$ = -14.3 (*c* 1.20, $CHCl_3$); IR (neat) 3006, 2950, 1734, 1676, 1217, 759 cm^{-1}; ^1H NMR (500 MHz, $CDCl_3$) δ 8.12 (s, 1H), 7.55 (t, *J* = 7.5 Hz, 1H), 7.48 (d, *J* = 7.8 Hz, 1H), 7.44 (d, *J* = 7.5 Hz, 1H), 7.35 (t, *J* = 7.4 Hz, 1H), 7.13 – 7.12 (m, 1H), 6.91 (d, *J* = 4.0 Hz, 2H), 6.22 (d, *J* = 15.7 Hz, 1H), 5.57 – 5.51 (m, 1H), 5.35 – 5.27 (m, 1H), 4.87 (d, *J* = 17.0 Hz, 1H), 4.82 (d, *J* = 10.2 Hz, 1H), 3.86 (s, 3H), 3.04 (dd, *J* = 13.6, 7.6 Hz, 1H), 2.98 (dd, *J* = 13.6, 7.2 Hz, 1H), 2.68 (dd, *J* = 13.6, 7.6 Hz, 1H), 2.61 (dd, *J* = 13.6, 7.2 Hz, 1H); ^{13}C NMR (126 MHz, $CDCl_3$) δ 198.0, 165.3, 150.5, 145.2, 139.6, 132.2, 131.8, 131.6, 129.2, 127.8, 127.3, 126.3, 125.6, 124.9, 123.7, 121.3, 118.6, 57.8, 52.3, 45.5, 44.7; HRMS (ESI+) *m*/*z* calcd. for $C_{22}H_{20}NaO_3$: 355.1310; found: 355.12306; The product was analyzed by HPLC to determine the enantiomeric excess: 90% ee (Chiralpak OD-3, *i*-propanol/hexane = 20/80, flow rate 1.0 mL/min, λ = 254 nm); t_r = 9.09 and 11.97 min. The absolute configuration of **4b** was speculated based on the proposed transition state shown in Figure 1b.

7. Synthetic Application of 2a

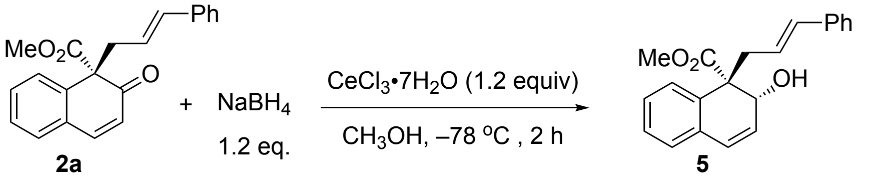

2a
92% ee
5
88% yield, 92% ee, 96:4 dr

To 1 mL of MeOH were added **2a** (63.6 mg, 0.2 mmol, 92% ee) and $CeCl_3•7H_2O$ (1.1 mg, 0.22 mmol), then the reaction system was cooled to –78 °C. After which $NaBH_4$ (8.4 mg, 0.22 mmol) was added slowly to the solution. After stirring at –78 °C temperature for 2 h, the solvent was evaporated and the residue was purified by column chromatography to give **5** in 88% yield, which was then directly analyzed by HPLC to determine the enantiomeric excess.

Methyl (1*S*,2*R*)-1-cinnamyl-2-hydroxy-1,2-dihydronaphthalene-1-carboxylate (5):

Yield (88%); colorless viscous liquid; $[\alpha]^{23}_D$ = +69.0 (*c* 0.73, $CHCl_3$); IR (neat) 3447, 3024, 2950, 1730, 1224, 967, 744 cm^{-1}; ^1H NMR (500 MHz, $CDCl_3$) δ 7.28 – 7.18 (m, 8H), 7.11 (d, *J* = 6.8 Hz,

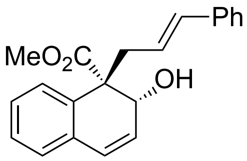

5

1H), 6.46 (d, J = 9.7 Hz, 1H), 6.34 – 6.26 (m, 2H), 6.08 (dd, J = 9.6, 3.5 Hz, 1H), 5.10 (d, J = 6.2 Hz, 1H), 3.75 (s, 3H), 3.03 (dd, J = 13.5, 5.6 Hz, 1H), 2.96 (dd, J = 13.5, 5.6 Hz, 1H), 1.86 (d, J = 8.1 Hz, 1H); ^{13}C NMR (125 MHz, $CDCl_3$) δ 174.4, 137.4, 134.9, 132.8, 132.1, 130.7, 128.4, 127.8, 127.4, 127.1, 127.1, 126.8, 126.1, 70.4, 57.2, 52.4, 35.0; HRMS (ESI+) *m*/*z* calcd. for $C_{21}H_{20}NaO_3$: 343.1305; found: 343.299. The product was analyzed by HPLC to determine the enantiomeric excess: 92% ee (Chiralpak AD-3, *i*-propanol/hexane = 20/80, flow rate 1.0 mL/min, λ = 254 nm); t_r = 6.78 and 8.15 min.

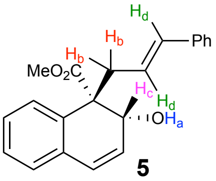

5

H-H COSY

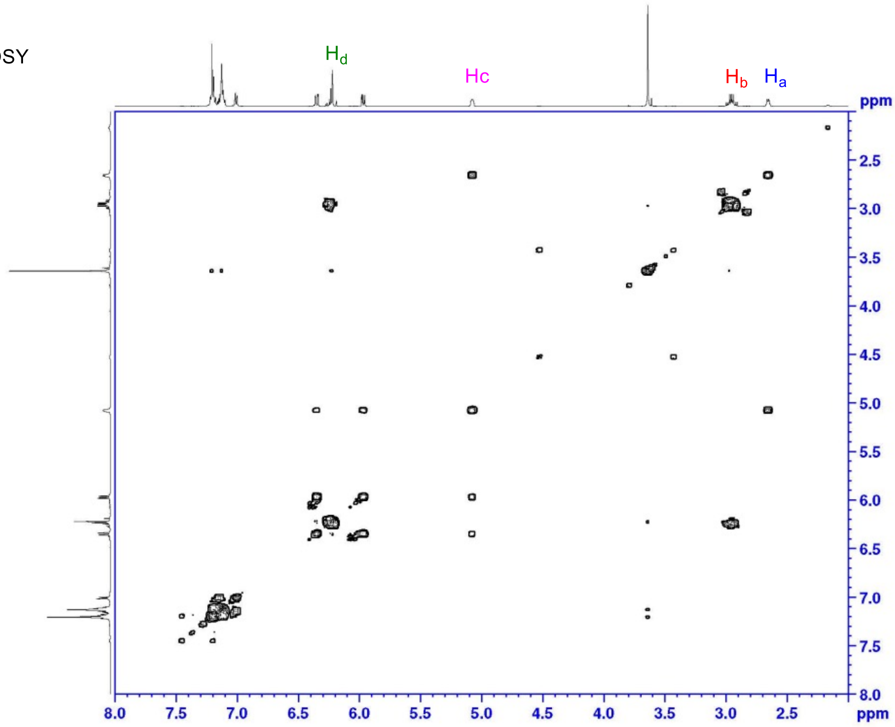

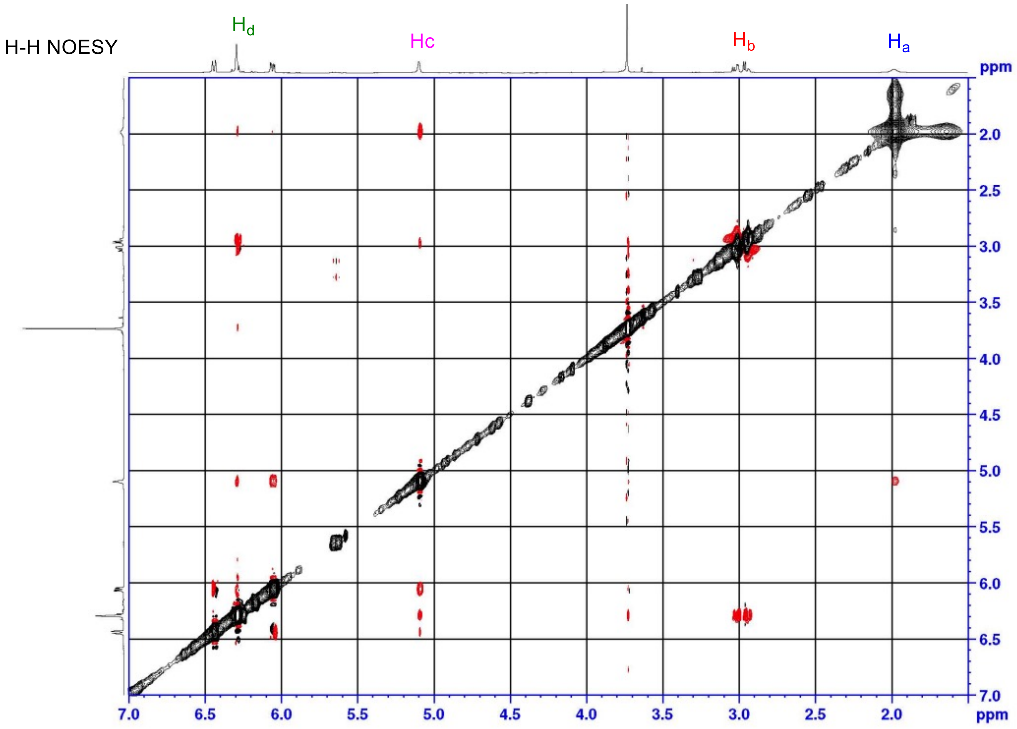

8. SX-ray Structure of Product (*S*)-2l and Cu(II) Complex

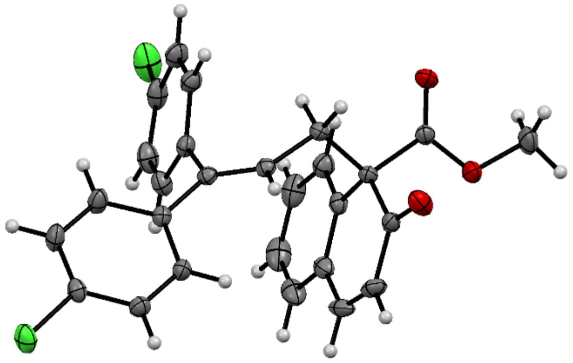

Figure S1. X-ray structure of **(*S*)-2l**

Crystal data for (*S*)-**2l**: $C_{27}H_{20}Cl_2O_3$, T = 123 K, Orthorhombic, space group *P212121*(#19), a = 7.6102(8), b = 10.7051(11), c = 27.326(3) Å, V = 2226.2(4) Å^3, Z = 4, λ(MoKα)= 0.71073 Å, 4973 reflections collected, and 294 parameters were used for the solution of the structure, final R_1 = 0.0256 and wR_2 = 0.0671, GOF = –0.005(9), CCDC 1865509 contains the supplementary crystallographic data for this paper.

a) bottom-up view

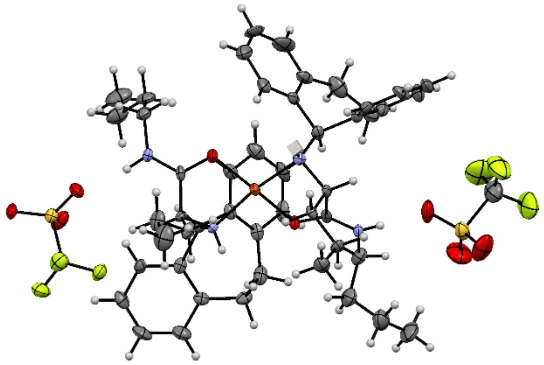

b) top view

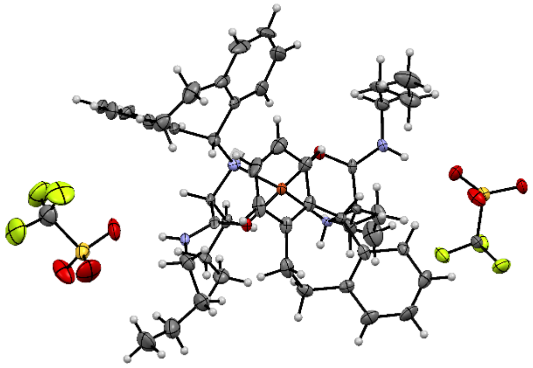

c) side view-1

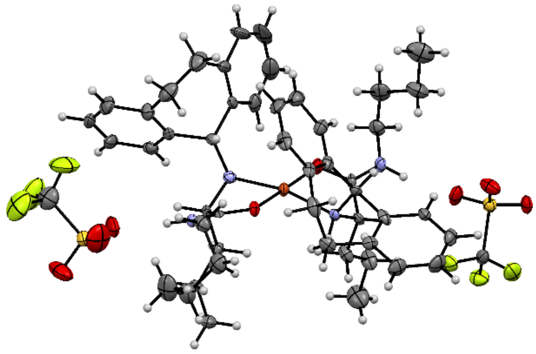

d) side view-2

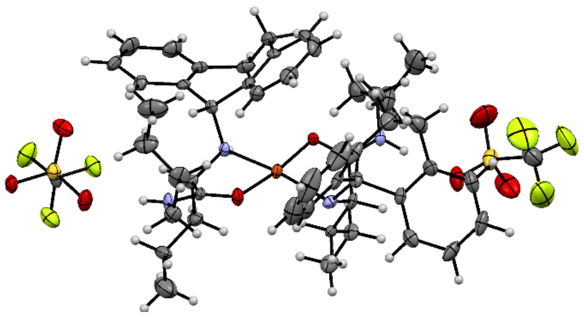

Figure S2. X-ray structure of Cu(II) complex

Crystal data for Cu(II) complex: $C_{50} H_{64} Cu F_6 N_4 O_8 S_2$, T = 123 K, orthorhombic, space group

P212121, *a* =14.293(3), *b* = 16.823(3), *c* = 21.700(5) Å, *V* = 5217.8(19) Å^3, *Z* = 4, λ(MoKα)= 0.71075 Å, 11938 reflections collected, and 648 parameters were used for the solution of the structure, final R_1 = 0.0834 and wR_2 = 0.1397, GOF = 1.107, CCDC 1865510 contains the supplementary crystallographic data for this paper.

9. References

1. (a) Aoyama, T.; Okutome, T.; Nakayama, T.; Yaegashi, T.; Matsui, R.; Nunomura, S.; Kurumi, M.; Sakurai, Y.; Fujii, S. *Chem. Pharm. Bull.* **1985,** *33*, 1458–1471. (b) Yin, Q.; Wang, S.-G.; Liang, X.-W.; Gao, D.-W.; Zheng, J.; You, S.-L., *Chem. Sci.* **2015,** *6*, 4179–4183.
2. Tayama, E.; Naganuma, N.; Iwamoto, H.; Hasegawa, E. *Chem. Comm.* **2014,** *50*, 6860–6862.
3. M. Amézquita-Valencia, H. Alper, *Org. Lett.* **2014**, *16*, 5827-5829.
4. P. Wawrzyniak, M. K. Kindermann, G. Thede, R. Thede, P. G. Jones, S. Enthaler, K. Junge, M. Beller, J. W. Heinicke, *Eur. J. Inorg. Chem.* **2017**, *2017*, 2762-2773.
5. M. Uyanik, N. Sahara, K. Ishihara, *Eur. J. Org. Chem.*, DOI: 10.1002/ejoc.201801063

10. ^{1}H NMR and ^{13}C NMR Spectra

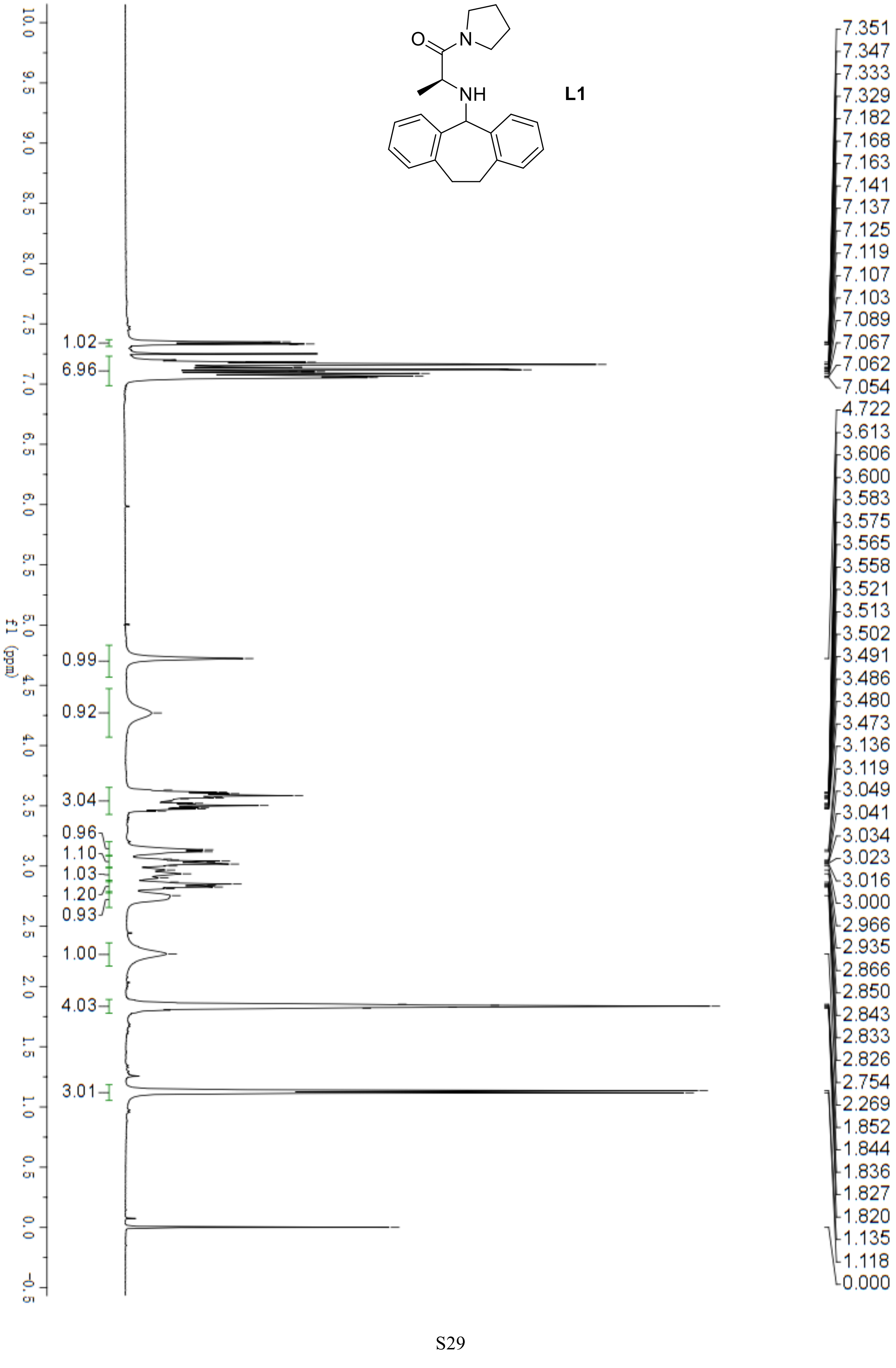

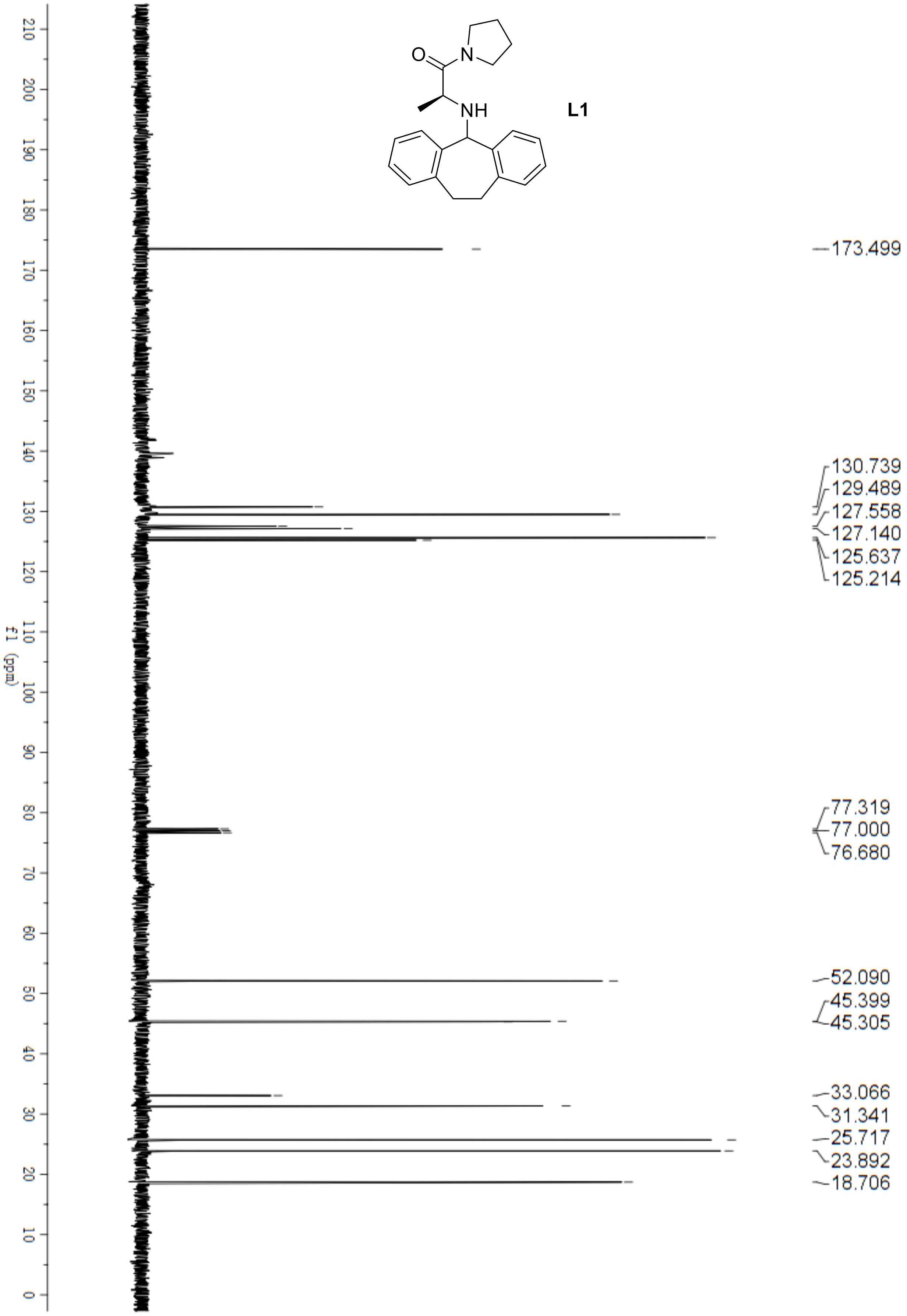
L1
173.499
130.739
129.489
127.558
127.140
125.637
125.214
77.319
77.000
76.680
52.090
45.399
45.305
33.066
31.341
25.717
23.892
18.706
f1 (ppm)

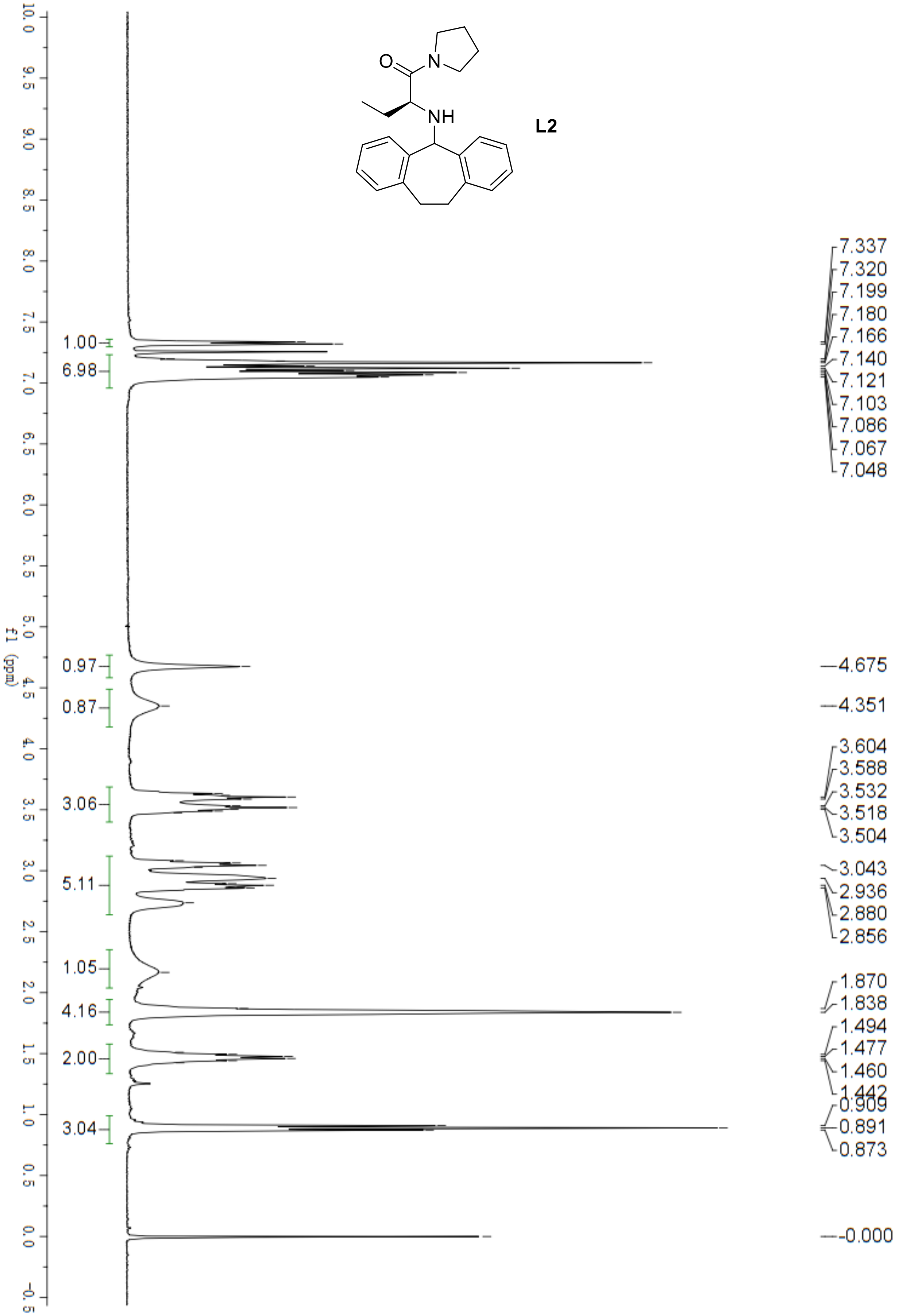

O
N
NH
L2
1.00
6.98
0.97
0.87
3.06
5.11
1.05
4.16
2.00
3.04
7.337
7.320
7.199
7.180
7.166
7.140
7.121
7.103
7.086
7.067
7.048
4.675
4.351
3.604
3.588
3.532
3.518
3.504
3.043
2.936
2.880
2.856
1.870
1.838
1.494
1.477
1.460
1.442
0.909
0.891
0.873
-0.000

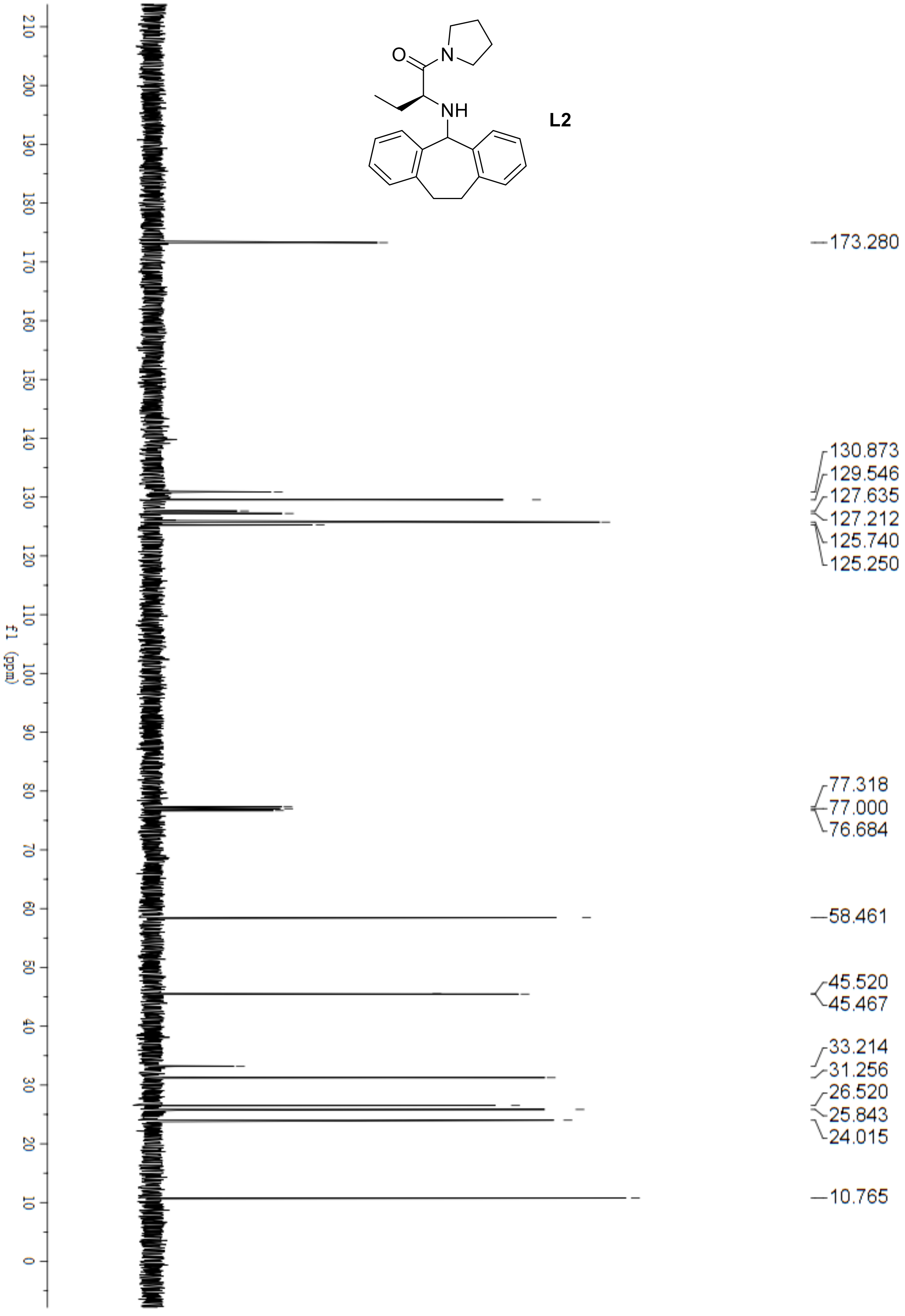
O
N
NH
L2
173.280
130.873
129.546
127.635
127.212
125.740
125.250
77.318
77.000
76.684
58.461
45.520
45.467
33.214
31.256
26.520
25.843
24.015
10.765
f1 (ppm)

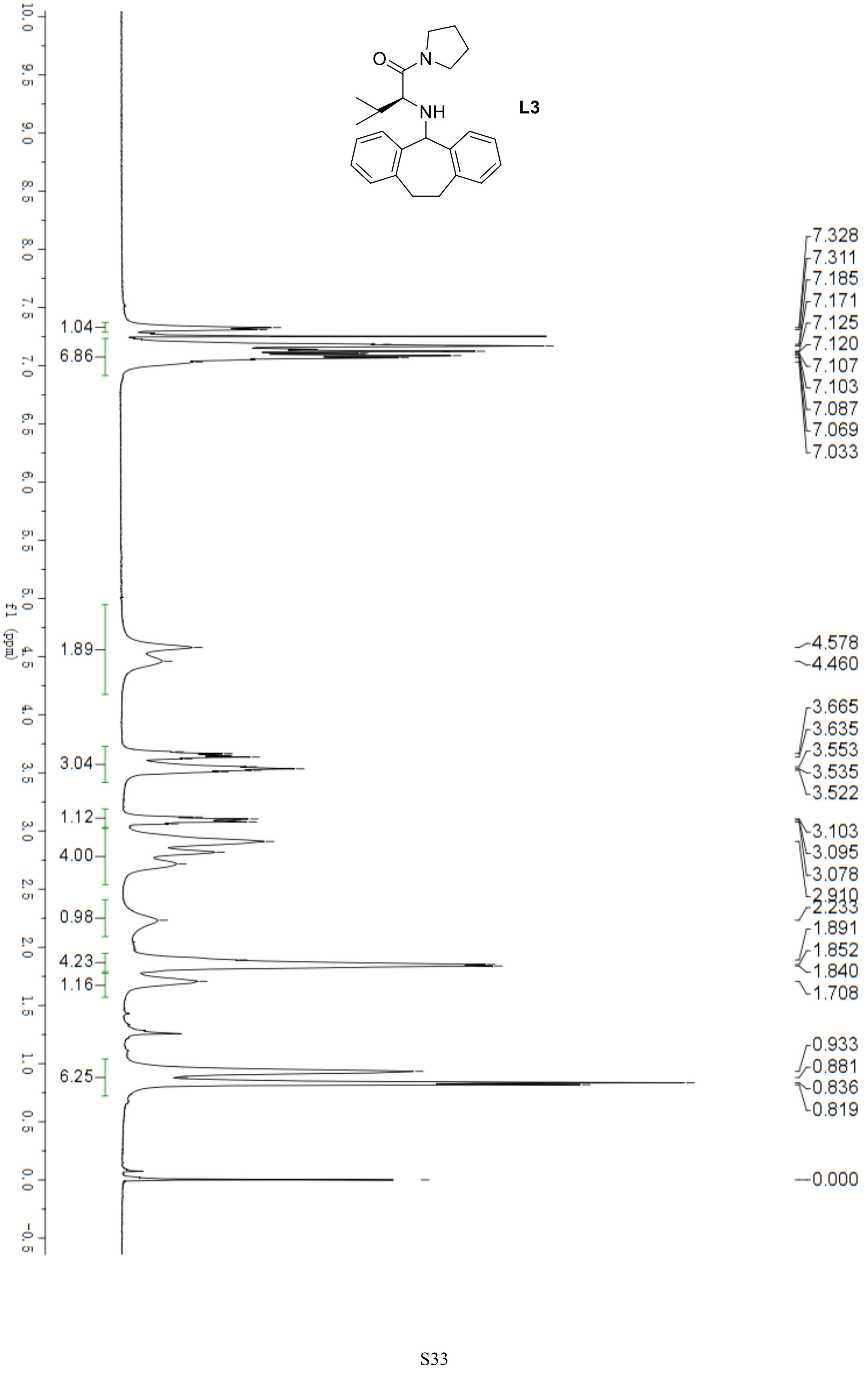

L3
NH
O
N
1.04
6.86
1.89
3.04
1.12
4.00
0.98
4.23
1.16
6.25
7.328
7.311
7.185
7.171
7.125
7.120
7.107
7.103
7.087
7.069
7.033
4.578
4.460
3.665
3.635
3.553
3.535
3.522
3.103
3.095
3.078
2.910
2.233
1.891
1.852
1.840
1.708
0.933
0.881
0.836
0.819
0.000

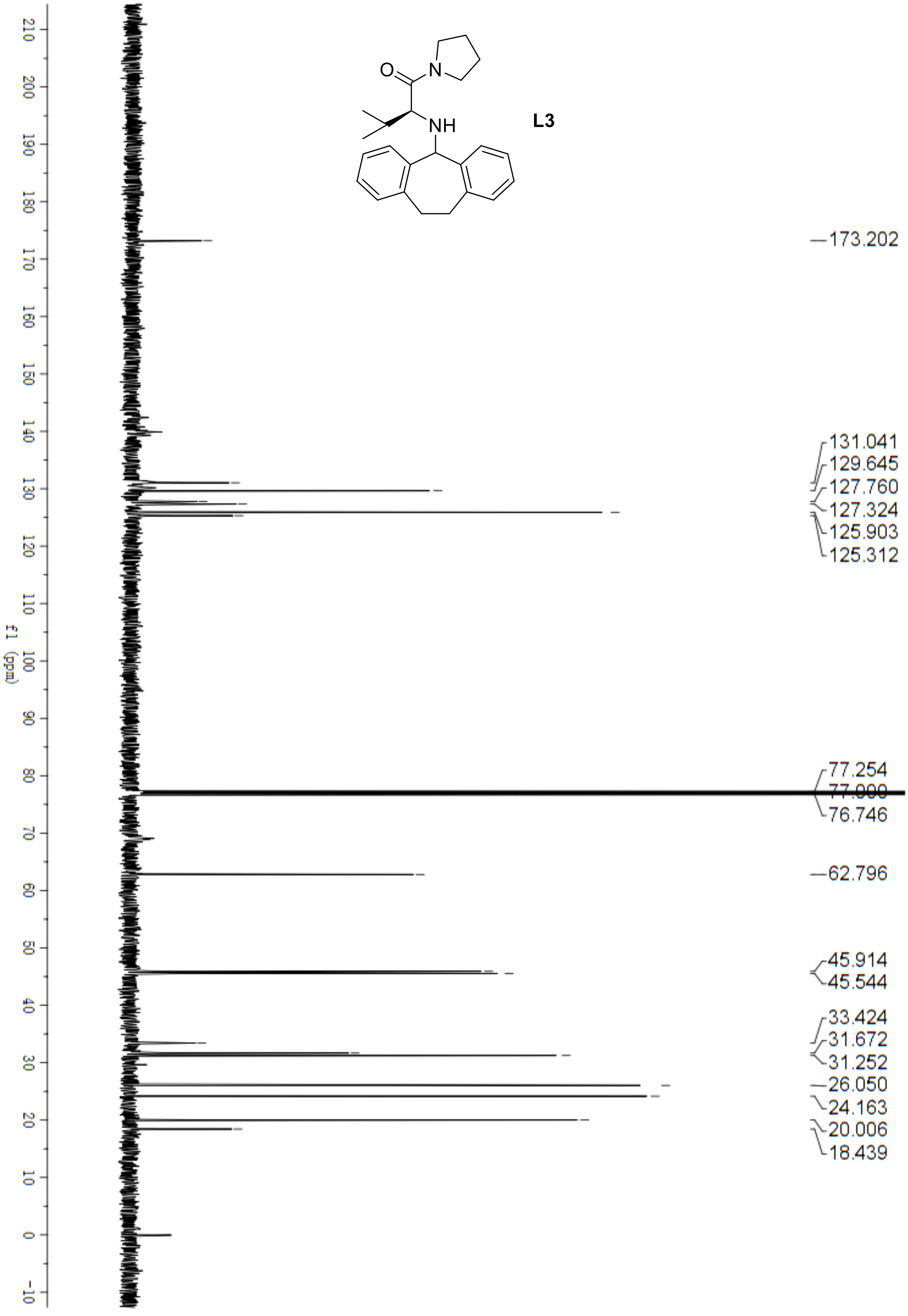
L3
O
N
NH
173.202
131.041
129.645
127.760
127.324
125.903
125.312
77.254
77.000
76.746
62.796
45.914
45.544
33.424
31.672
31.252
26.050
24.163
20.006
18.439
f1 (ppm)

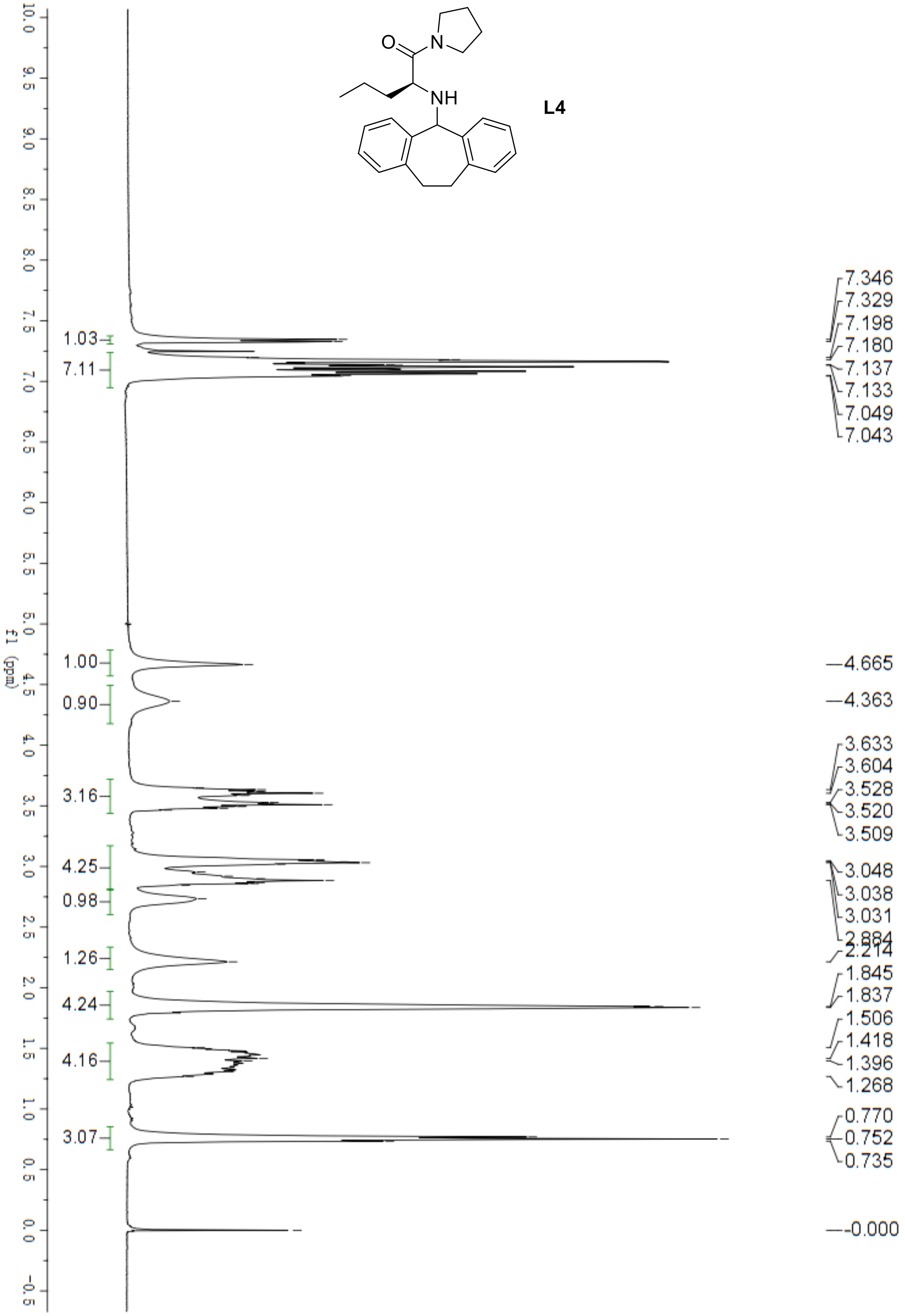
O
N
NH
L4
7.346
7.329
7.198
7.180
7.137
7.133
7.049
7.043
4.665
4.363
3.633
3.604
3.528
3.520
3.509
3.048
3.038
3.031
2.884
2.214
1.845
1.837
1.506
1.418
1.396
1.268
0.770
0.752
0.735
-0.000
1.03
7.11
1.00
0.90
3.16
4.25
0.98
1.26
4.24
4.16
3.07
f1 (ppm)

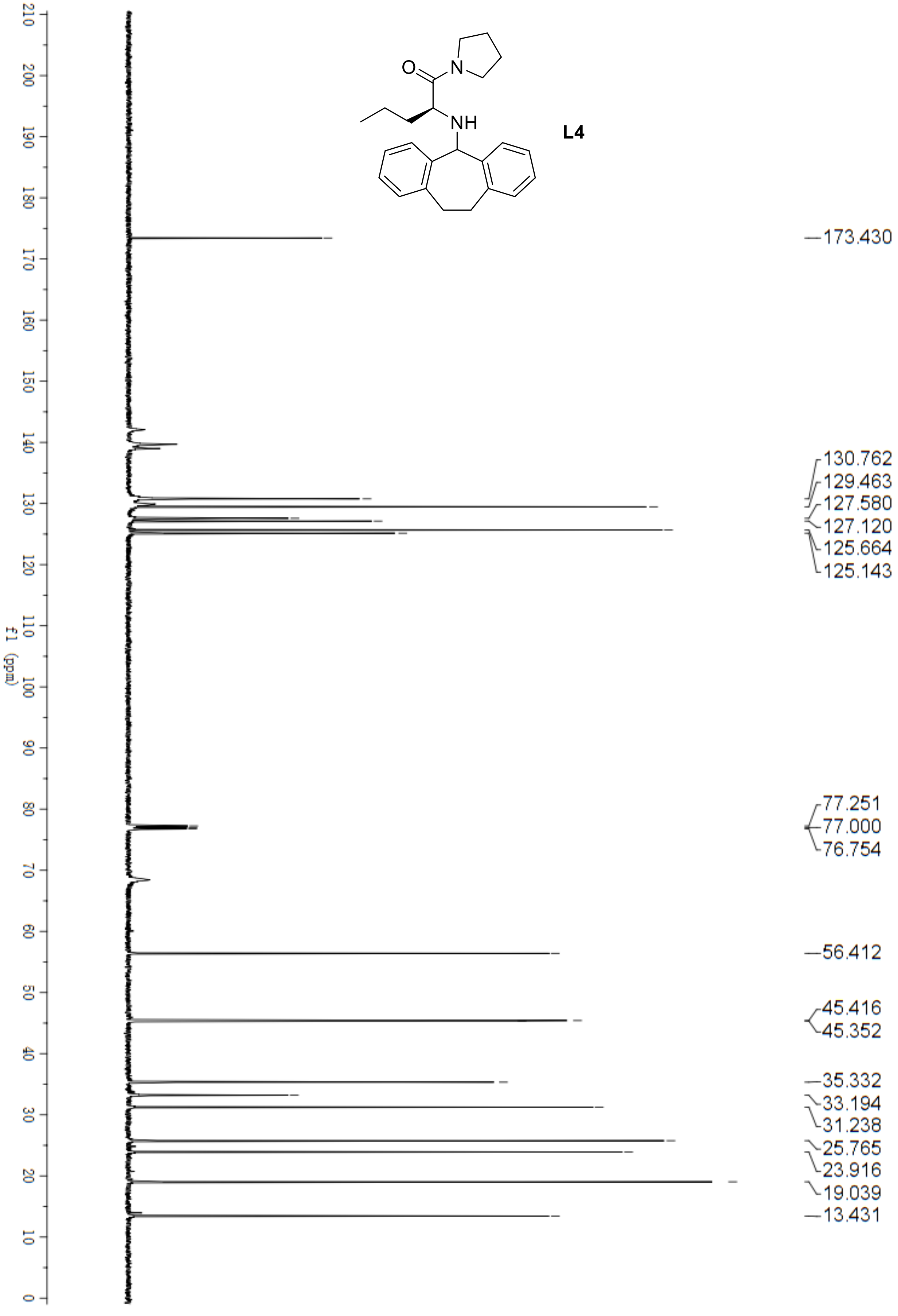
L4
173.430
130.762
129.463
127.580
127.120
125.664
125.143
77.251
77.000
76.754
56.412
45.416
45.352
35.332
33.194
31.238
25.765
23.916
19.039
13.431
f1 (ppm)

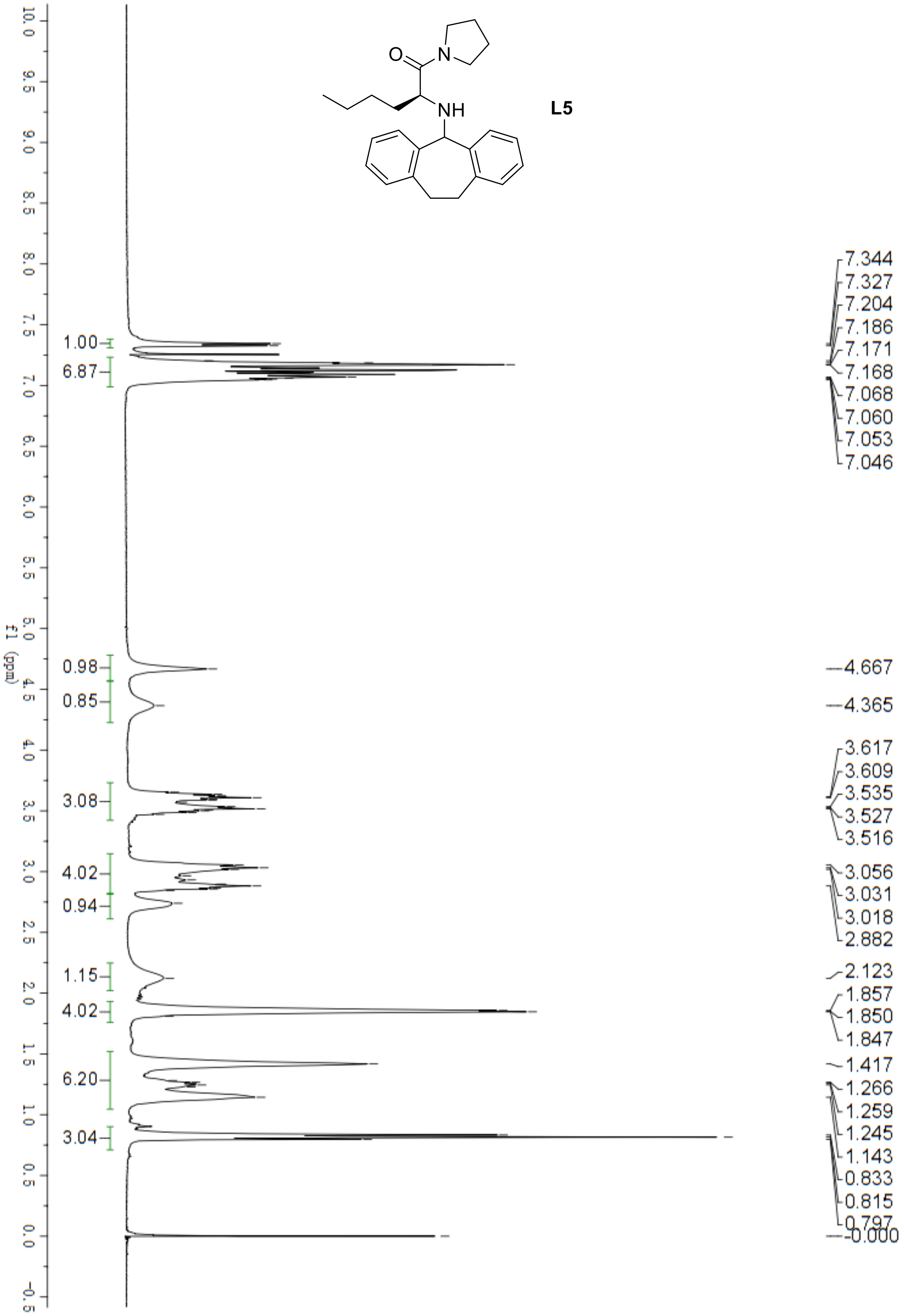
L5
7.344
7.327
7.204
7.186
7.171
7.168
7.068
7.060
7.053
7.046
4.667
4.365
3.617
3.609
3.535
3.527
3.516
3.056
3.031
3.018
2.882
2.123
1.857
1.850
1.847
1.417
1.266
1.259
1.245
1.143
0.833
0.815
0.797
-0.000
1.00
6.87
0.98
0.85
3.08
4.02
0.94
1.15
4.02
6.20
3.04
f1 (ppm)

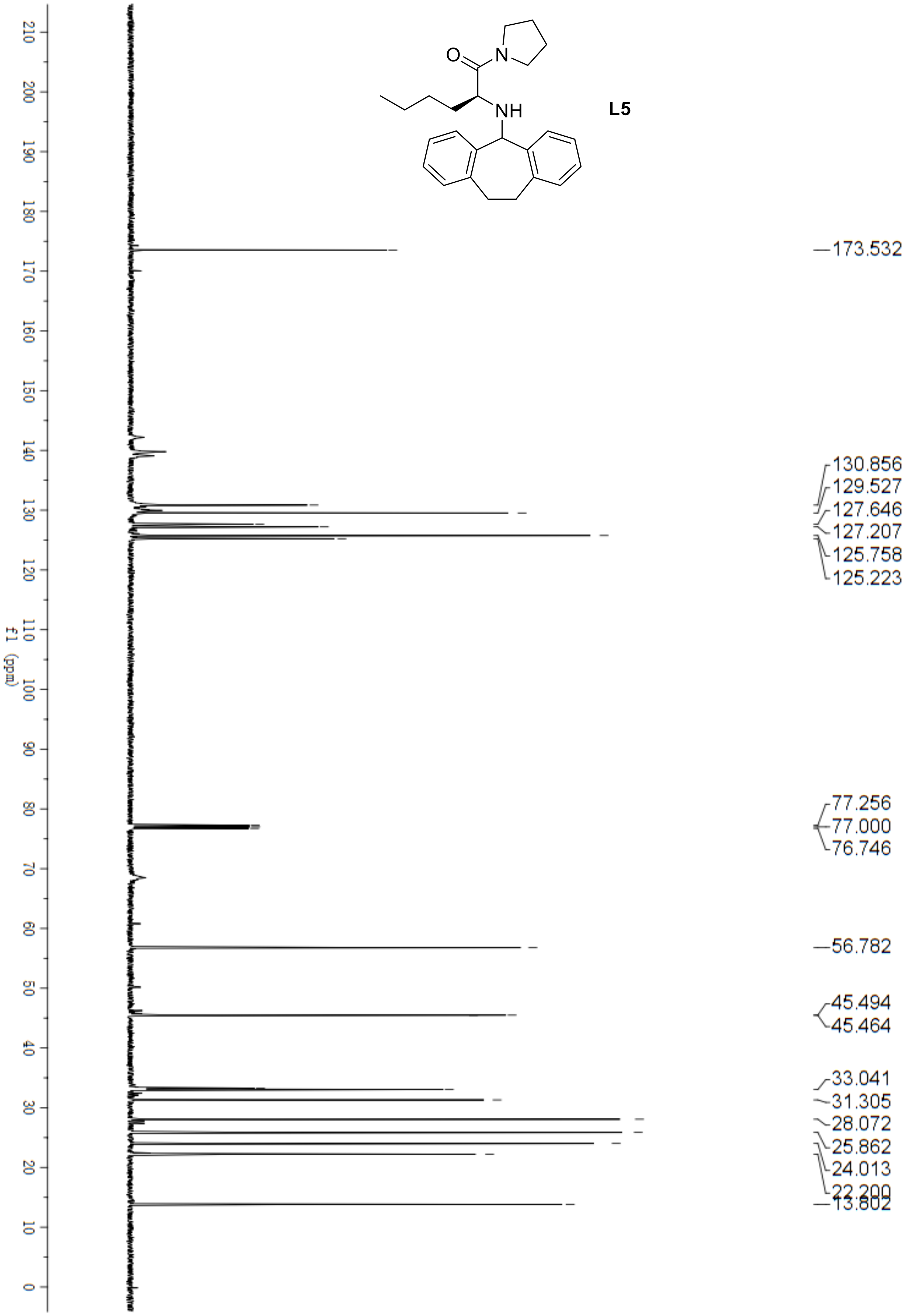
L5
O
N
NH
173.532
130.856
129.527
127.646
127.207
125.758
125.223
77.256
77.000
76.746
56.782
45.494
45.464
33.041
31.305
28.072
25.862
24.013
22.200
13.802
f1 (ppm)
210
200
190
180
170
160
150
140
130
120
110
100
90
80
70
60
50
40
30
20
10
0

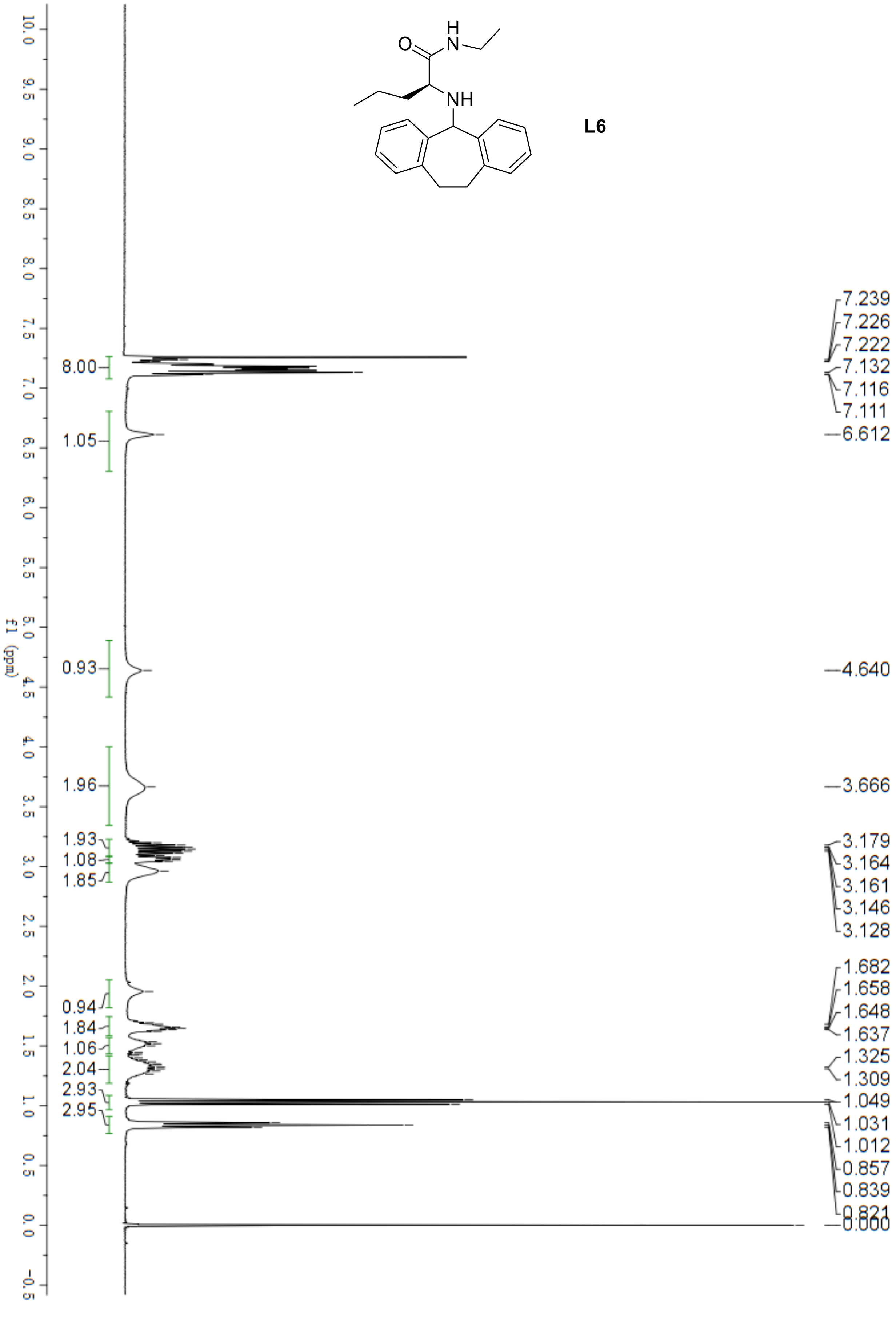
L6
f1 (ppm)
7.239
7.226
7.222
7.132
7.116
7.111
6.612
4.640
3.666
3.179
3.164
3.161
3.146
3.128
1.682
1.658
1.648
1.637
1.325
1.309
1.049
1.031
1.012
0.857
0.839
0.821
0.000
8.00
1.05
0.93
1.96
1.93
1.08
1.85
0.94
1.84
1.06
2.04
2.93
2.95

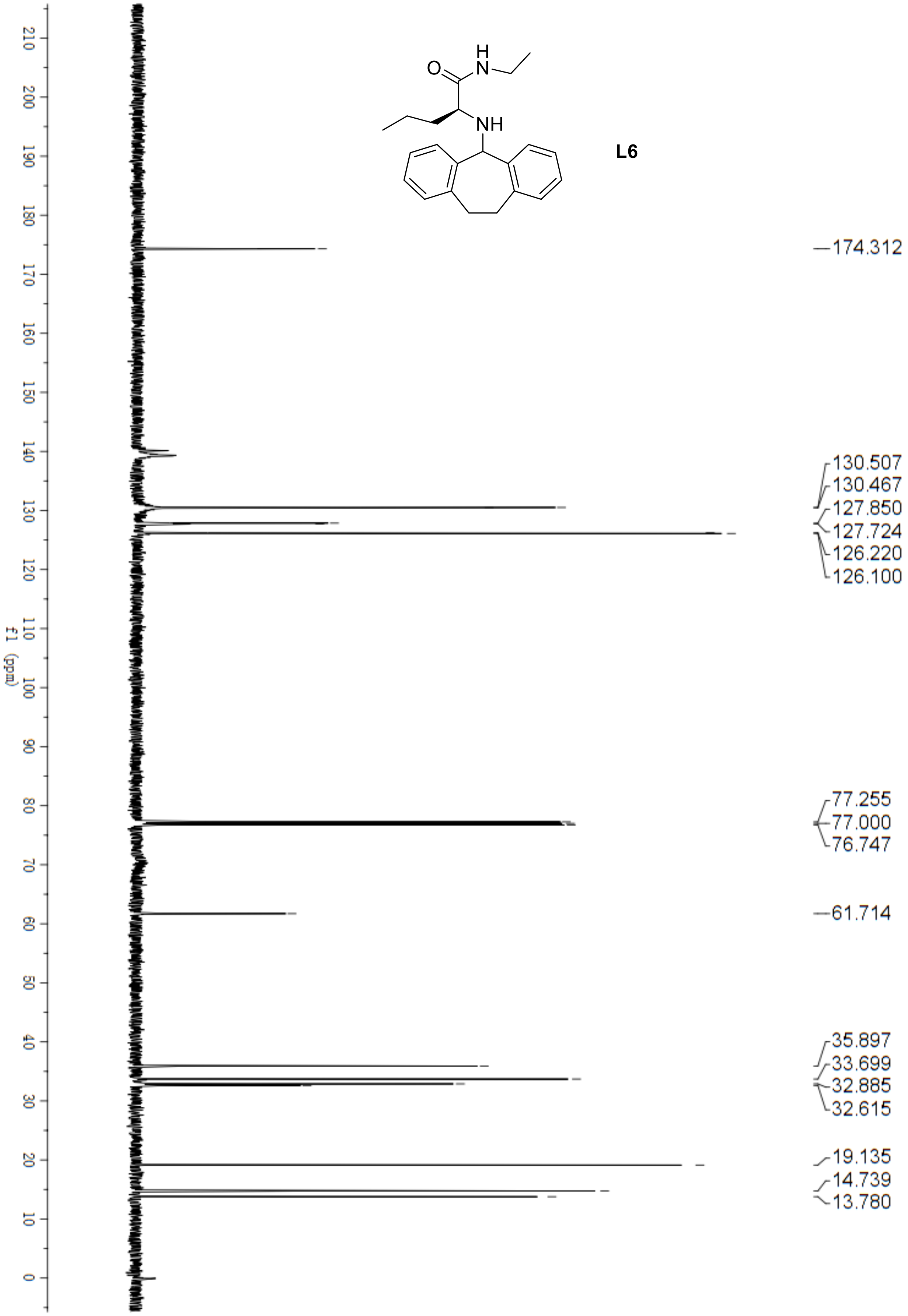

L6
f1 (ppm)
174.312
130.507
130.467
127.850
127.724
126.220
126.100
77.255
77.000
76.747
61.714
35.897
33.699
32.885
32.615
19.135
14.739
13.780

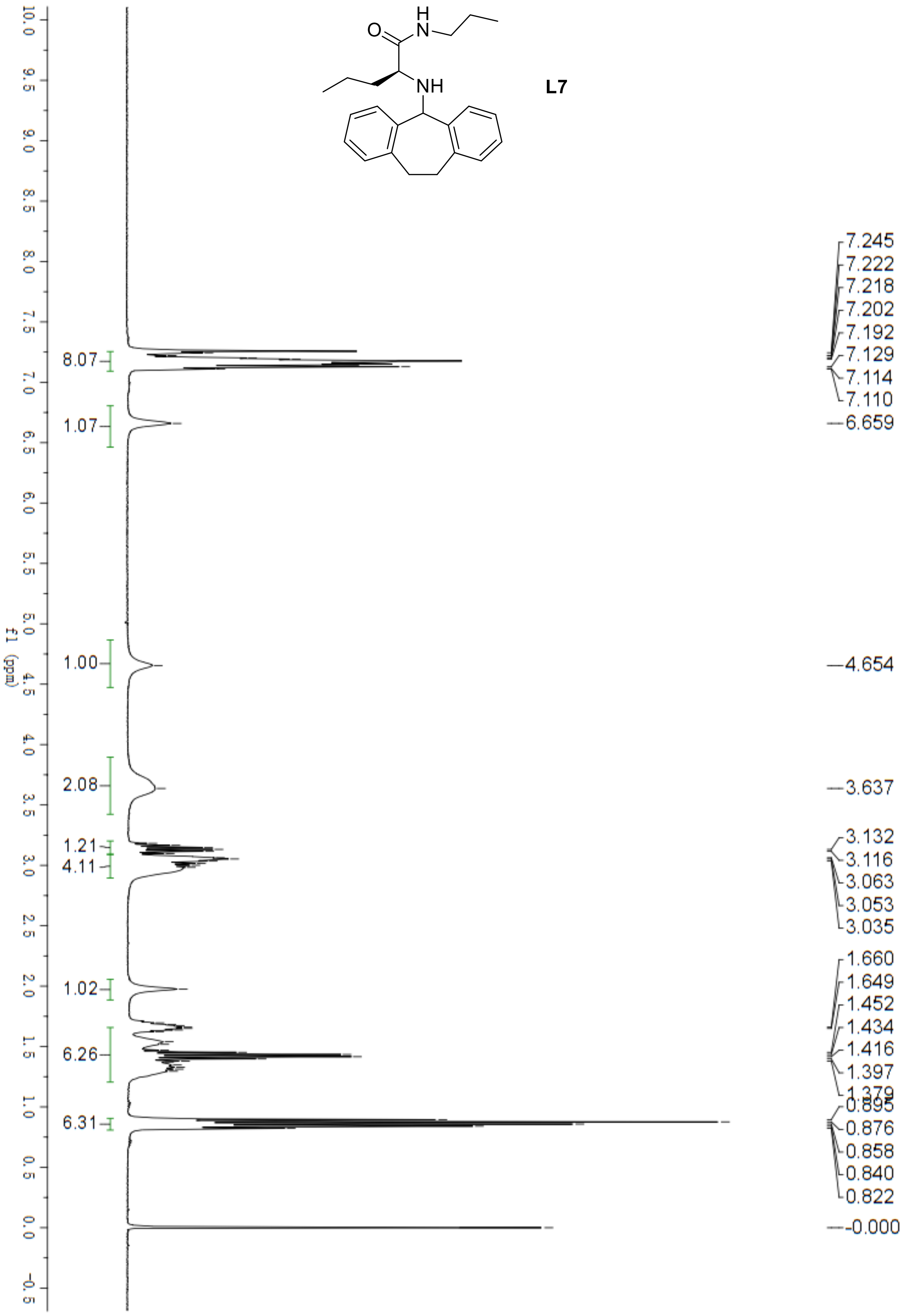
L7
7.245
7.222
7.218
7.202
7.192
7.129
7.114
7.110
6.659
4.654
3.637
3.132
3.116
3.063
3.053
3.035
1.660
1.649
1.452
1.434
1.416
1.397
1.379
0.895
0.876
0.858
0.840
0.822
-0.000
8.07
1.07
1.00
2.08
1.21
4.11
1.02
6.26
6.31
f1 (ppm)

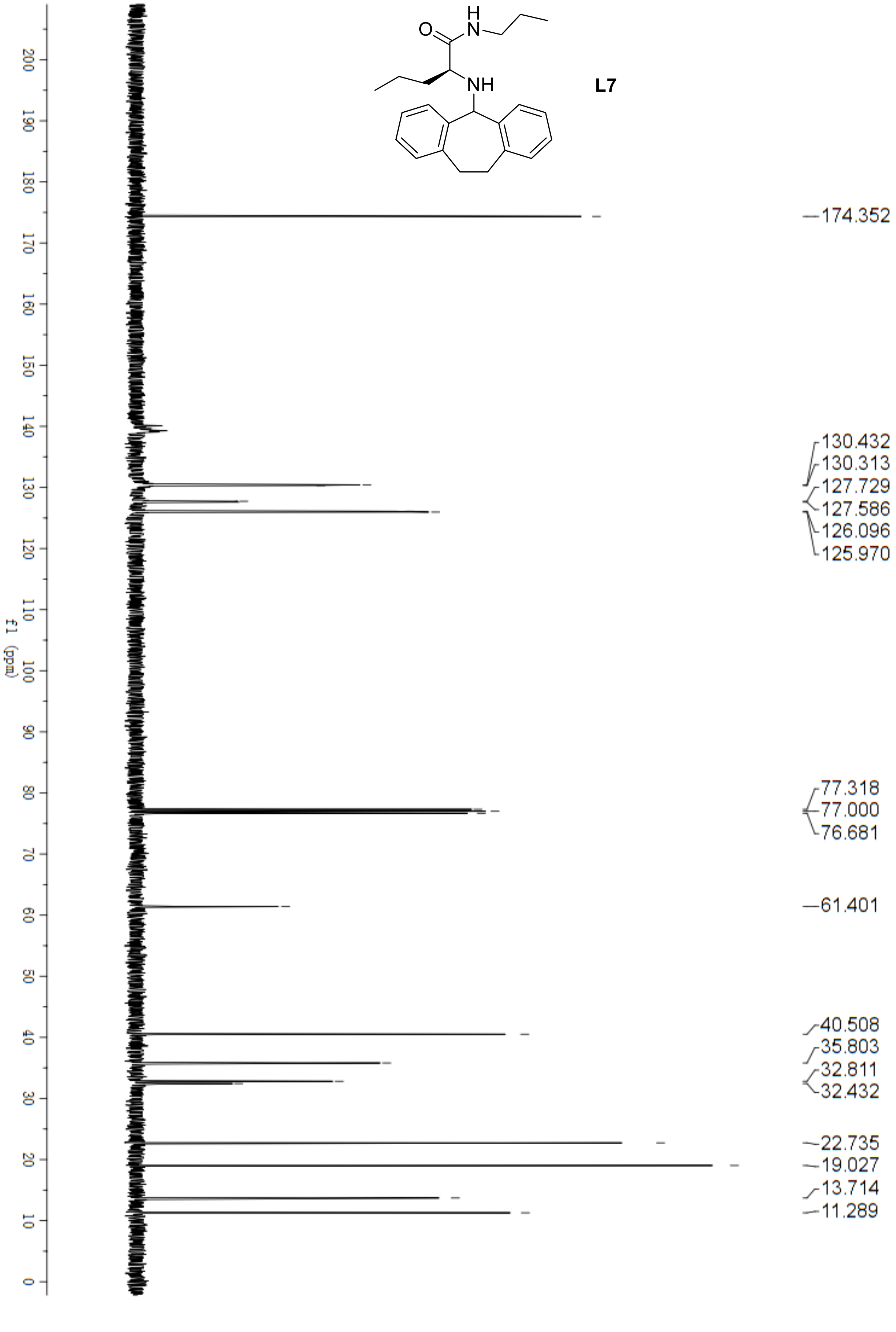
L7
174.352
130.432
130.313
127.729
127.586
126.096
125.970
77.318
77.000
76.681
61.401
40.508
35.803
32.811
32.432
22.735
19.027
13.714
11.289
f1 (ppm)

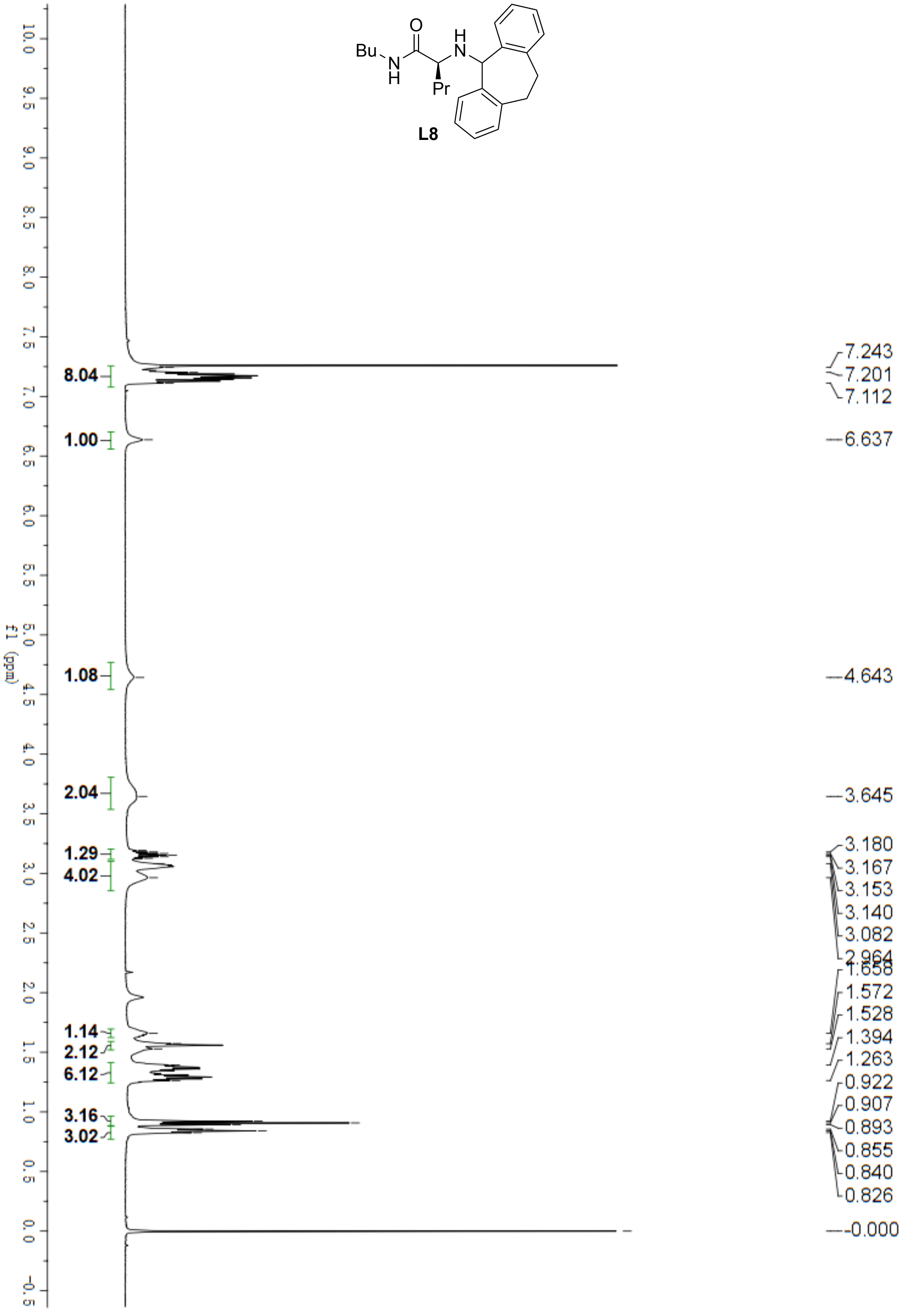
Bu
N
H
O
H
N
Pr
L8
7.243
7.201
7.112
6.637
4.643
3.645
3.180
3.167
3.153
3.140
3.082
2.964
1.658
1.572
1.528
1.394
1.263
0.922
0.907
0.893
0.855
0.840
0.826
-0.000
8.04
1.00
1.08
2.04
1.29
4.02
1.14
2.12
6.12
3.16
3.02
f1 (ppm)

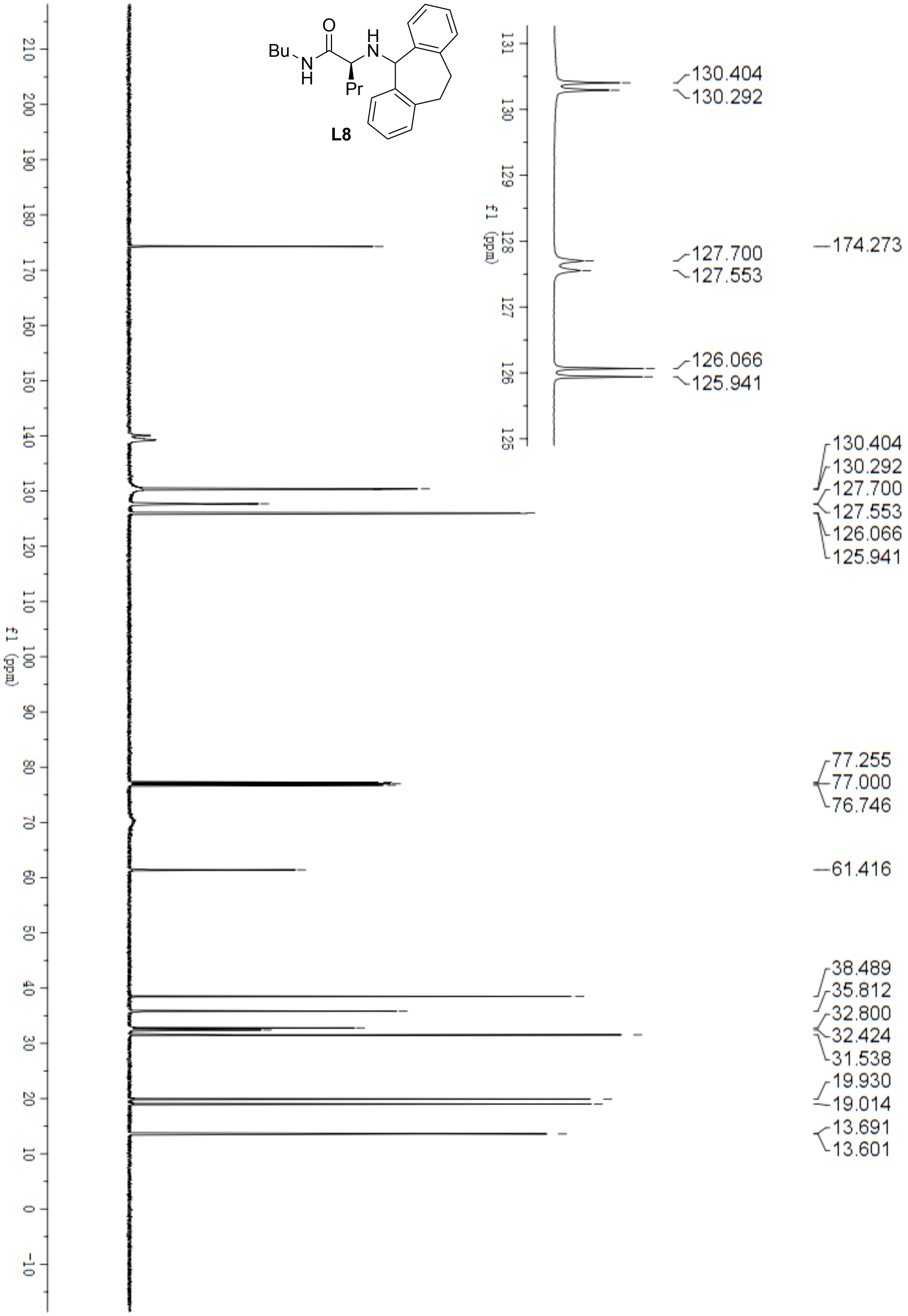
Bu
N
H
O
N
H
Pr
L8
174.273
130.404
130.292
127.700
127.553
126.066
125.941
77.255
77.000
76.746
61.416
38.489
35.812
32.800
32.424
31.538
19.930
19.014
13.691
13.601
f1 (ppm)

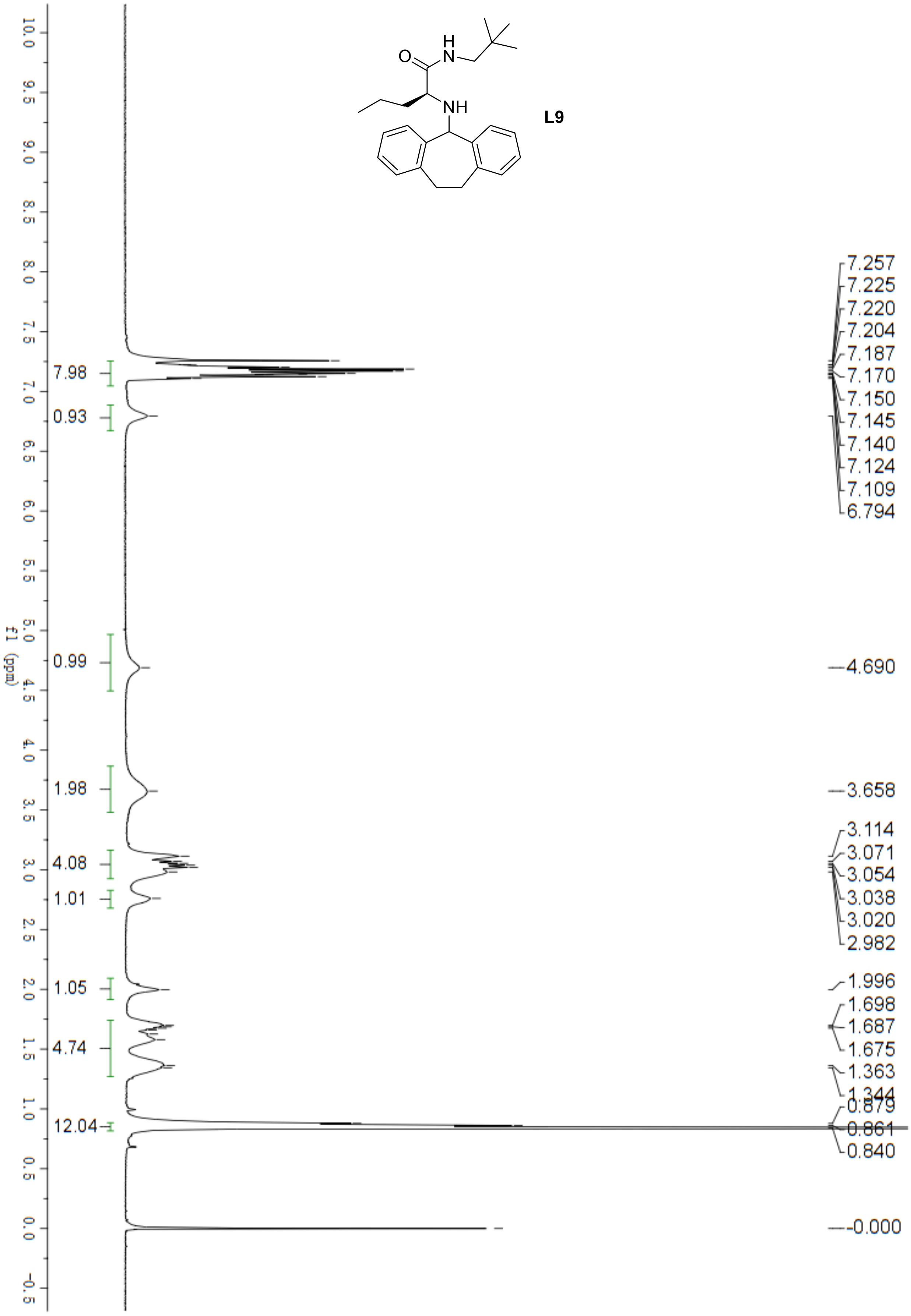
L9
7.257
7.225
7.220
7.204
7.187
7.170
7.150
7.145
7.140
7.124
7.109
6.794
4.690
3.658
3.114
3.071
3.054
3.038
3.020
2.982
1.996
1.698
1.687
1.675
1.363
1.344
0.879
0.861
0.840
-0.000
7.98
0.93
0.99
1.98
4.08
1.01
1.05
4.74
12.04
f1 (ppm)

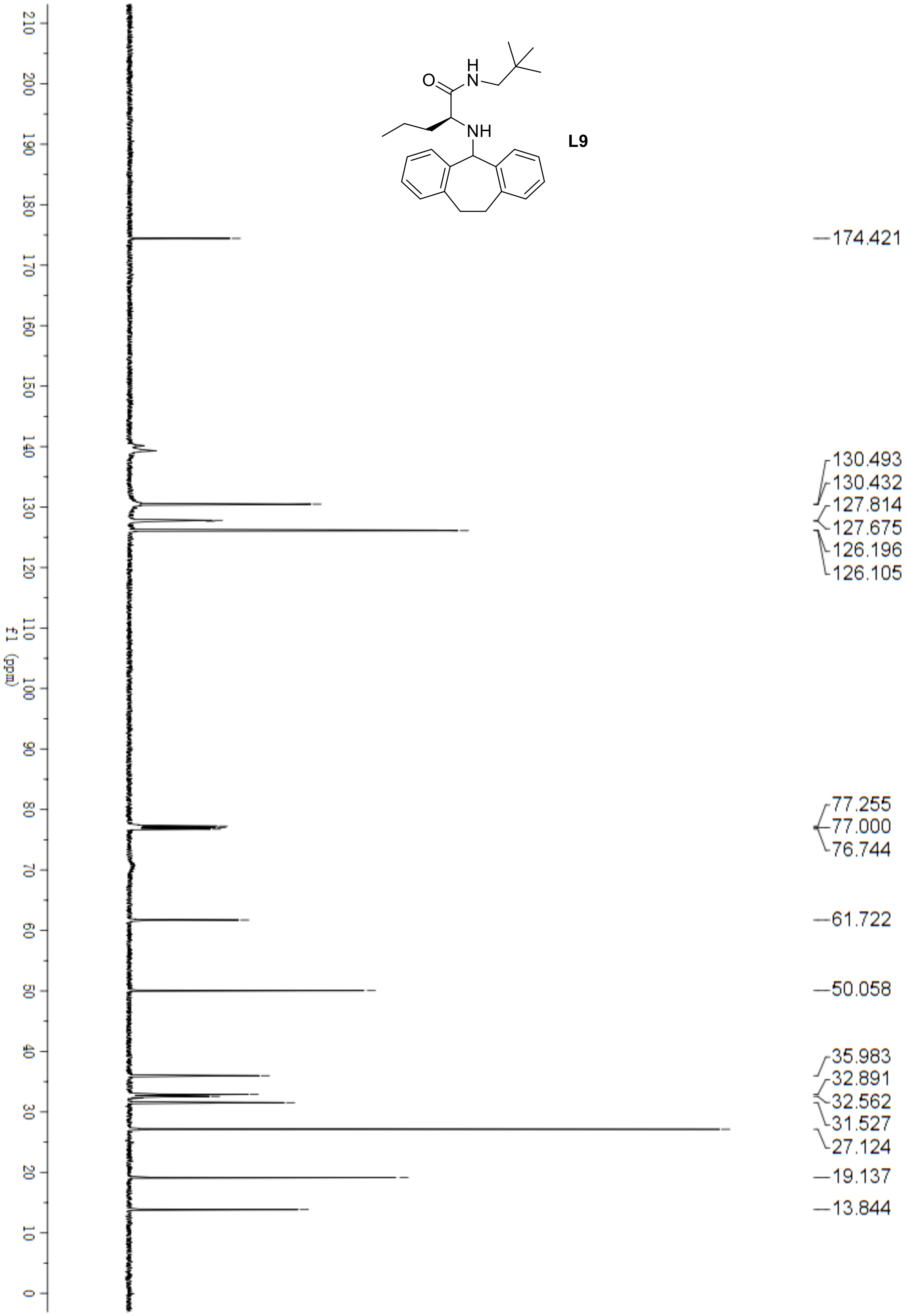
L9
174.421
130.493
130.432
127.814
127.675
126.196
126.105
77.255
77.000
76.744
61.722
50.058
35.983
32.891
32.562
31.527
27.124
19.137
13.844
f1 (ppm)

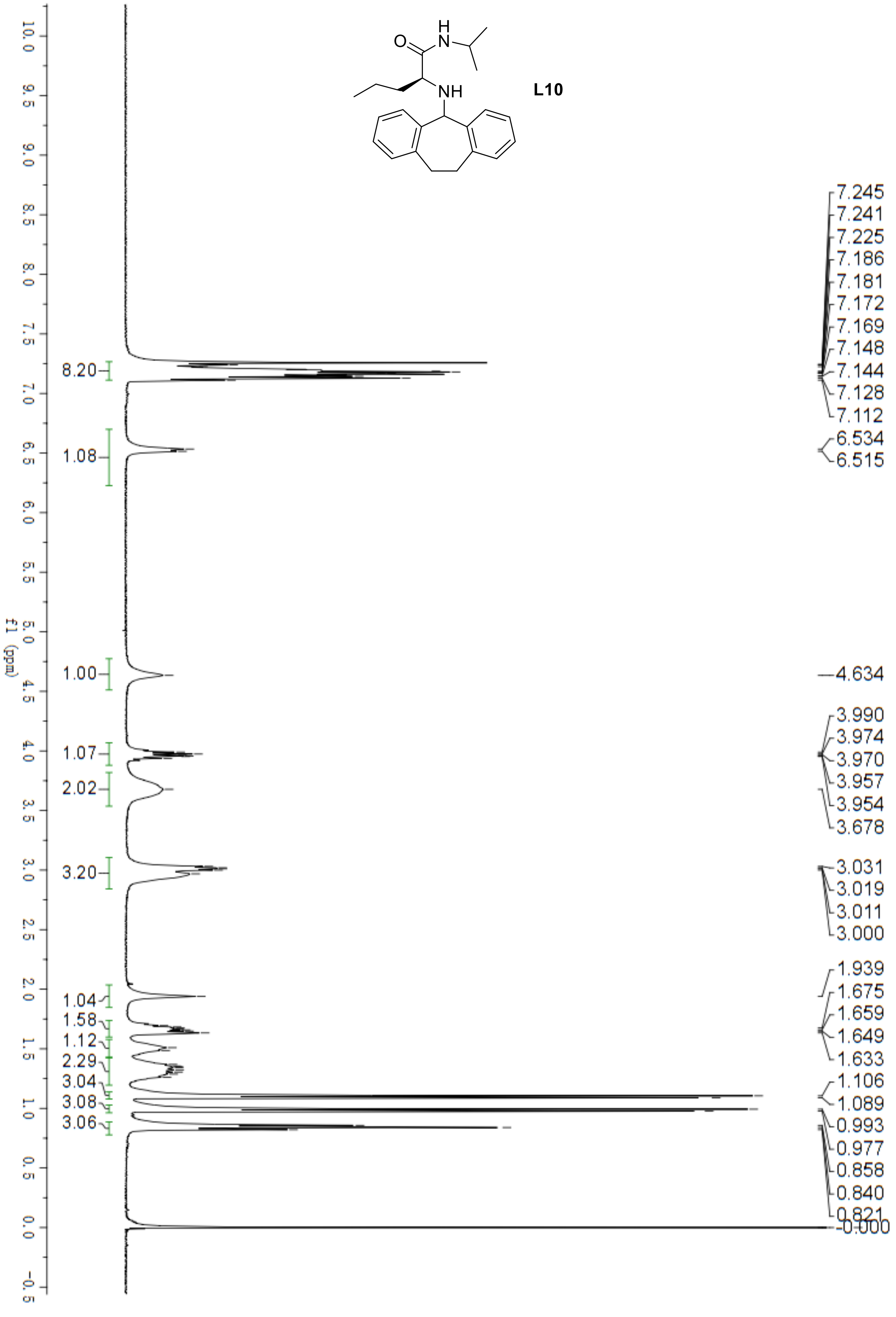
L10
7.245
7.241
7.225
7.186
7.181
7.172
7.169
7.148
7.144
7.128
7.112
6.534
6.515
4.634
3.990
3.974
3.970
3.957
3.954
3.678
3.031
3.019
3.011
3.000
1.939
1.675
1.659
1.649
1.633
1.106
1.089
0.993
0.977
0.858
0.840
0.821
0.000
8.20
1.08
1.00
1.07
2.02
3.20
1.04
1.58
1.12
2.29
3.04
3.08
3.06
f1 (ppm)

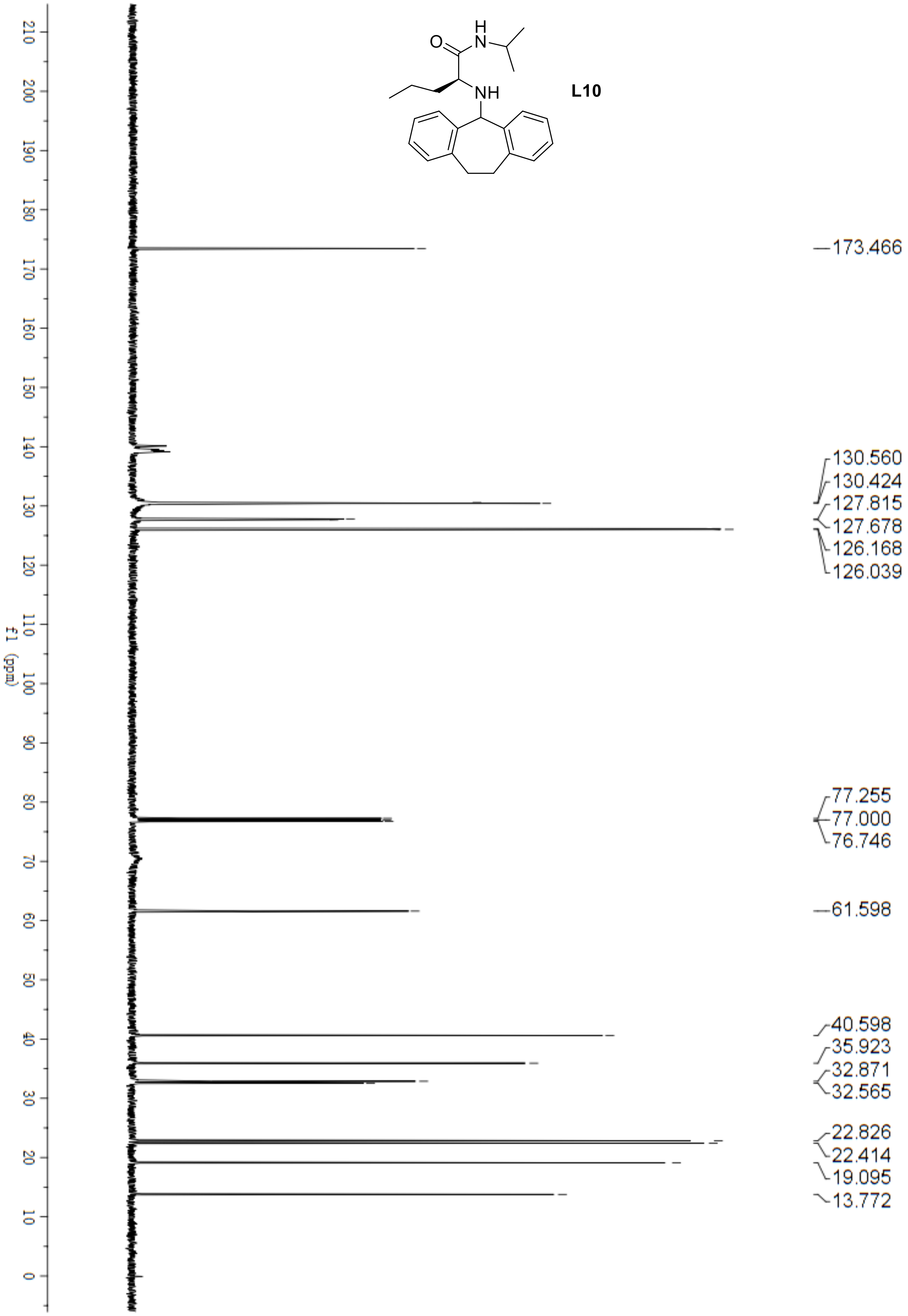
173.466
130.560
130.424
127.815
127.678
126.168
126.039
77.255
77.000
76.746
61.598
40.598
35.923
32.871
32.565
22.826
22.414
19.095
13.772
f1 (ppm)

L10

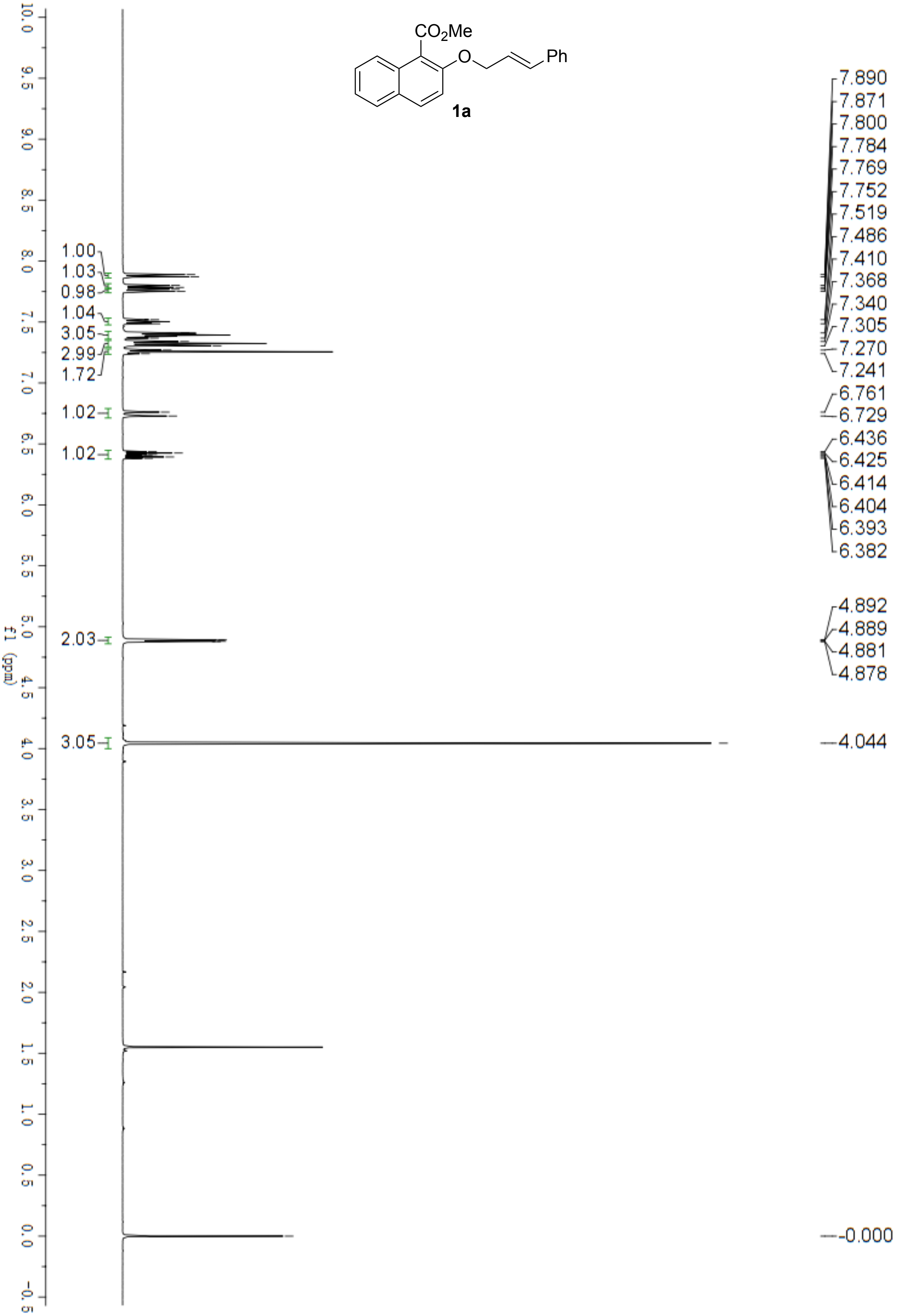
CO2Me
O
Ph
1a
7.890
7.871
7.800
7.784
7.769
7.752
7.519
7.486
7.410
7.368
7.340
7.305
7.270
7.241
6.761
6.729
6.436
6.425
6.414
6.404
6.393
6.382
4.892
4.889
4.881
4.878
4.044
-0.000
1.00
1.03
0.98
1.04
3.05
2.99
1.72
1.02
1.02
2.03
3.05
f1 (ppm)
10.0
9.5
9.0
8.5
8.0
7.5
7.0
6.5
6.0
5.5
5.0
4.5
4.0
3.5
3.0
2.5
2.0
1.5
1.0
0.5
0.0
-0.5

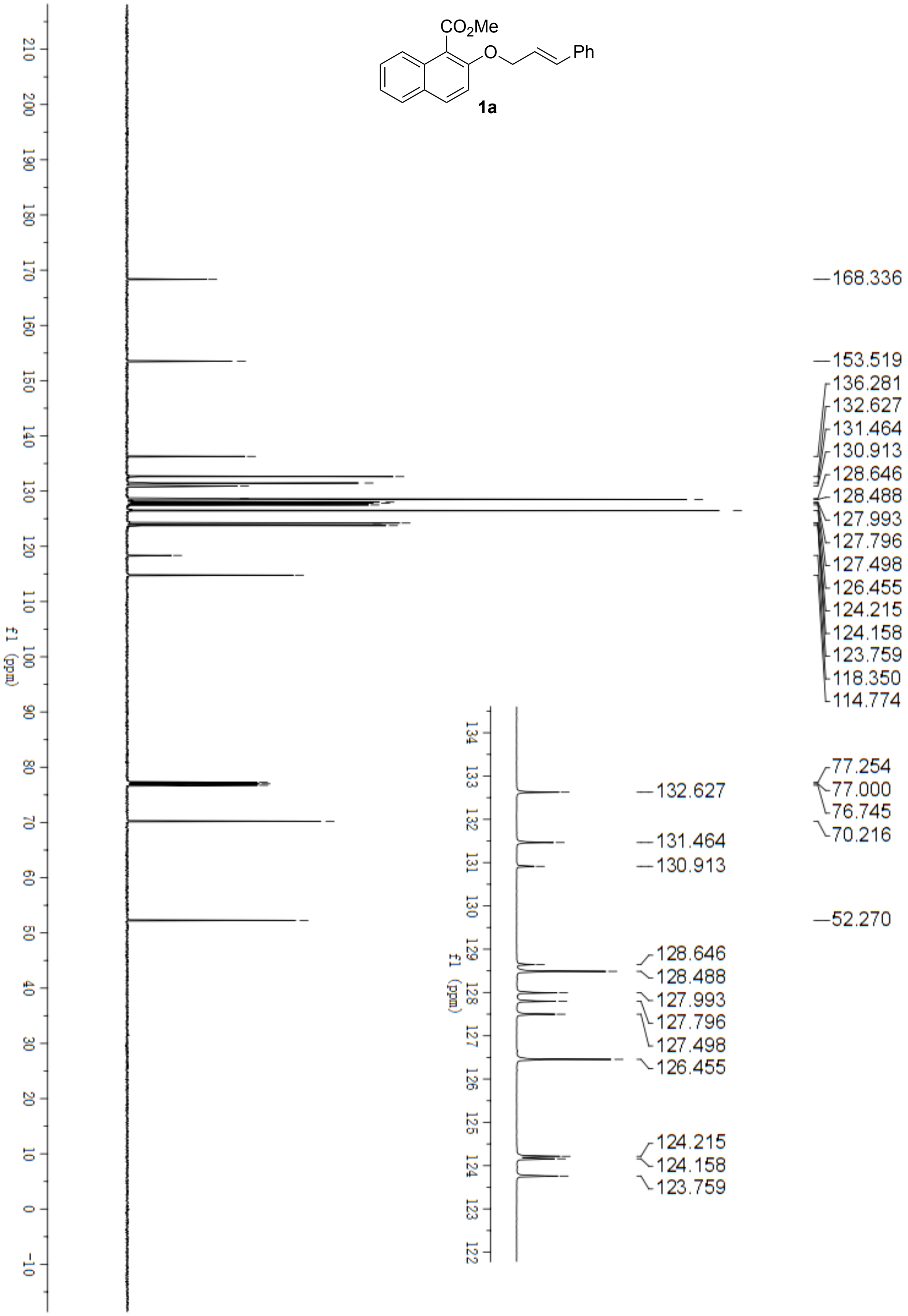
CO2Me
O
Ph
1a
168.336
153.519
136.281
132.627
131.464
130.913
128.646
128.488
127.993
127.796
127.498
126.455
124.215
124.158
123.759
118.350
114.774
77.254
77.000
76.745
70.216
52.270
f1 (ppm)

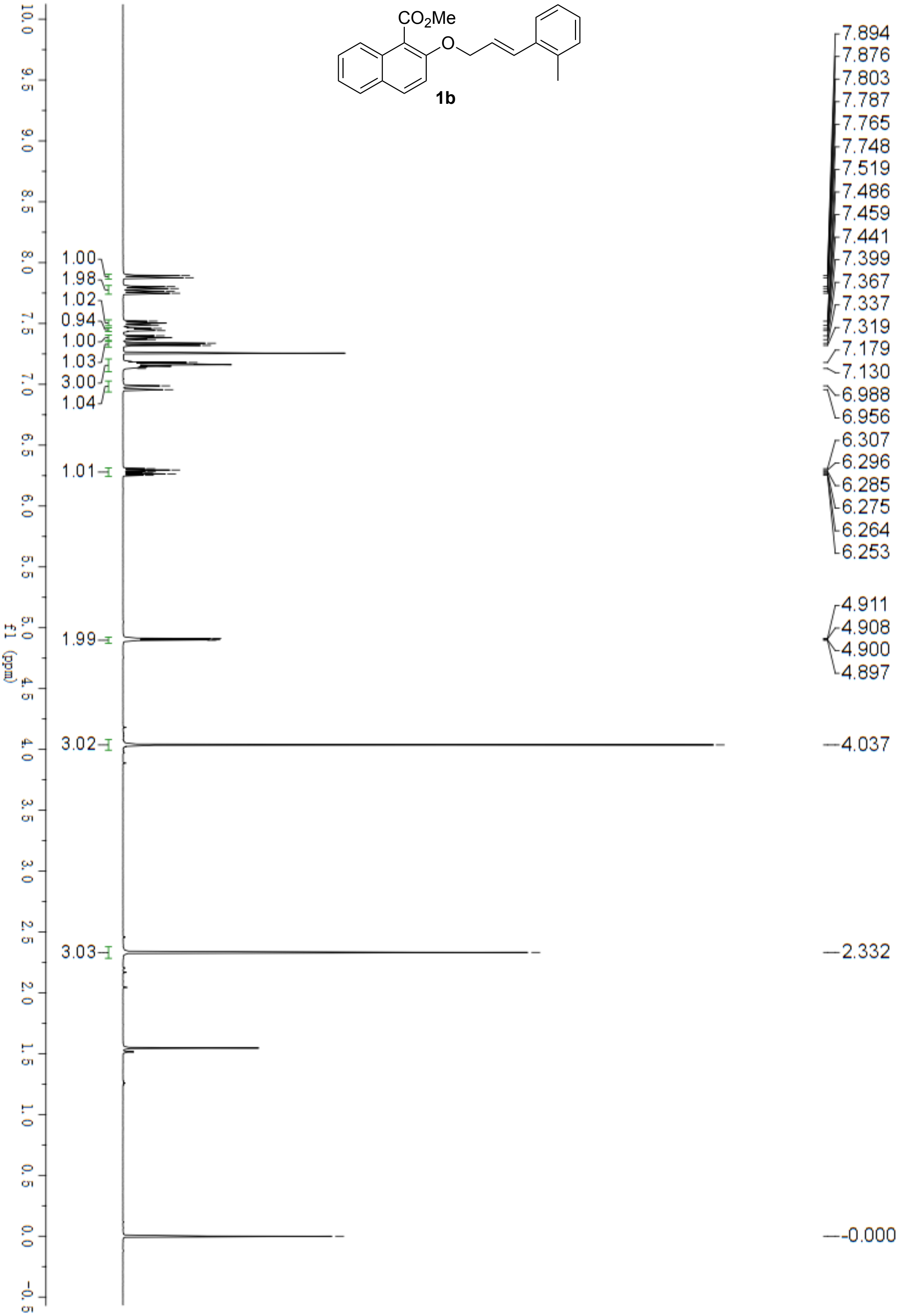
CO2Me
O
1b
7.894
7.876
7.803
7.787
7.765
7.748
7.519
7.486
7.459
7.441
7.399
7.367
7.337
7.319
7.179
7.130
6.988
6.956
6.307
6.296
6.285
6.275
6.264
6.253
4.911
4.908
4.900
4.897
4.037
2.332
-0.000
1.00
1.98
1.02
0.94
1.00
1.03
3.00
1.04
1.01
1.99
3.02
3.03
f1 (ppm)

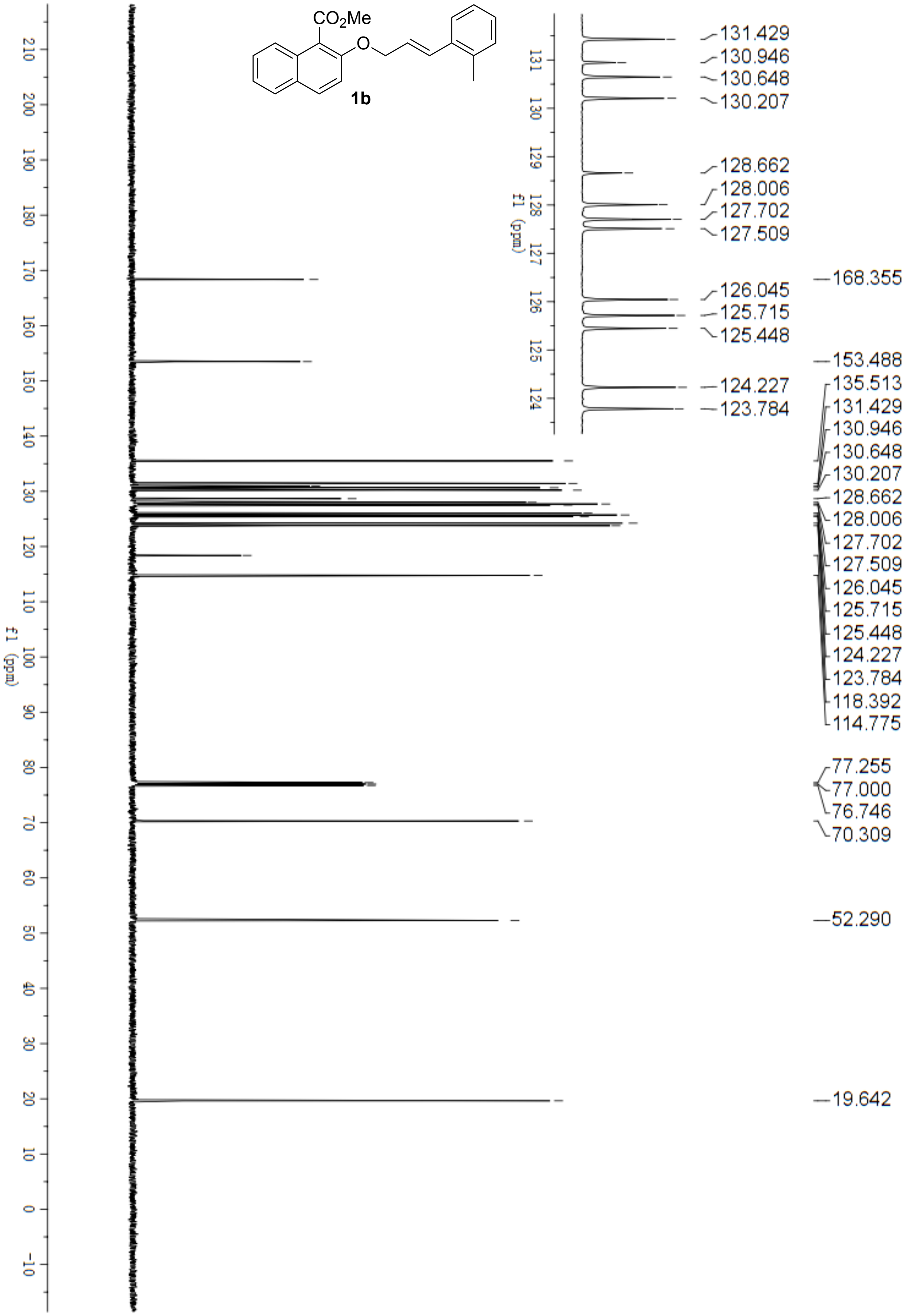

CO2Me
O
1b
131.429
130.946
130.648
130.207
128.662
128.006
127.702
127.509
126.045
125.715
125.448
124.227
123.784
168.355
153.488
135.513
131.429
130.946
130.648
130.207
128.662
128.006
127.702
127.509
126.045
125.715
125.448
124.227
123.784
118.392
114.775
77.255
77.000
76.746
70.309
52.290
19.642
f1 (ppm)

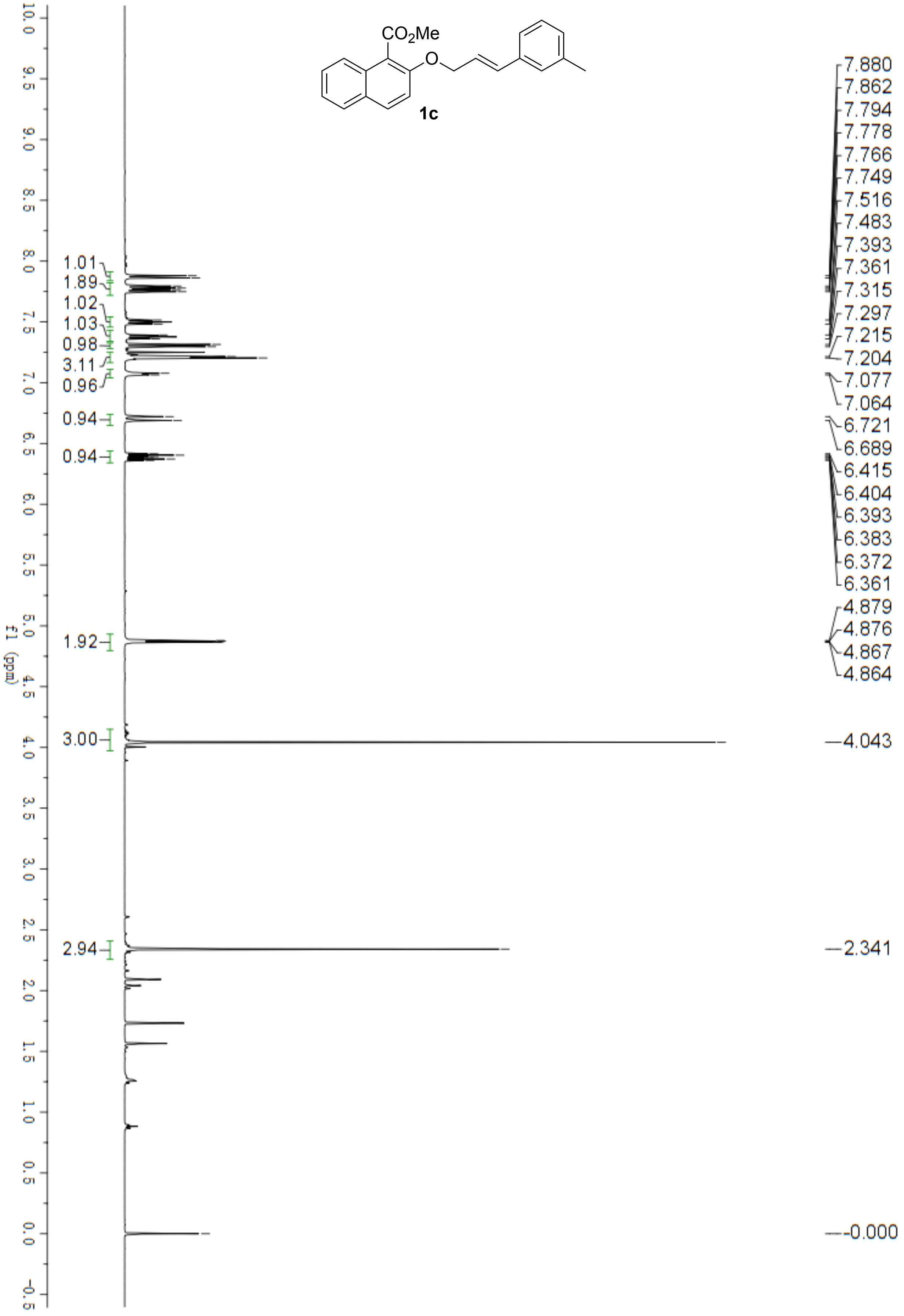
CO2Me
O
1c
7.880
7.862
7.794
7.778
7.766
7.749
7.516
7.483
7.393
7.361
7.315
7.297
7.215
7.204
7.077
7.064
6.721
6.689
6.415
6.404
6.393
6.383
6.372
6.361
4.879
4.876
4.867
4.864
4.043
2.341
-0.000
1.01
1.89
1.02
1.03
0.98
3.11
0.96
0.94
0.94
1.92
3.00
2.94
f1 (ppm)

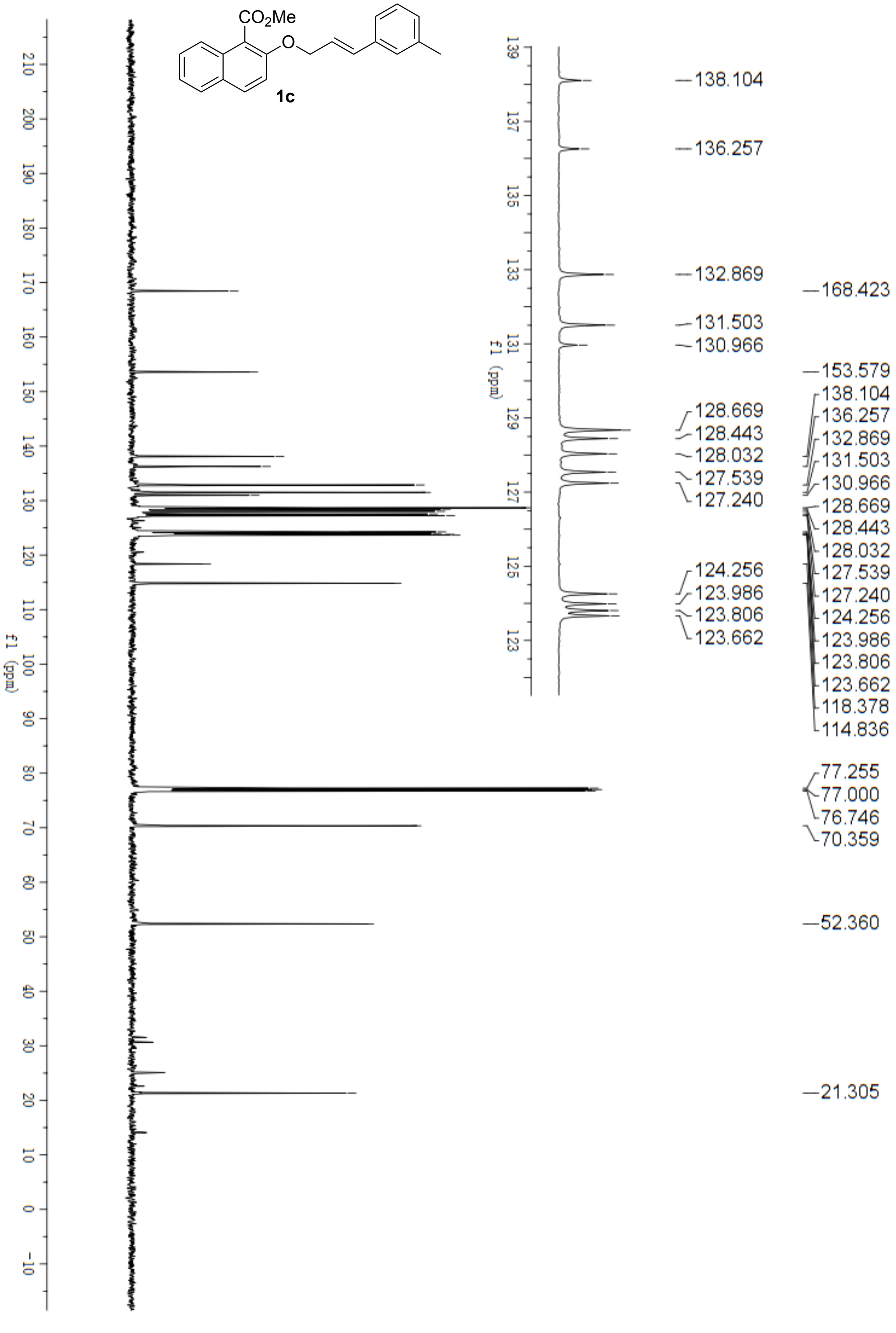

CO2Me
O
1c
138.104
136.257
132.869
131.503
130.966
128.669
128.443
128.032
127.539
127.240
124.256
123.986
123.806
123.662
168.423
153.579
138.104
136.257
132.869
131.503
130.966
128.669
128.443
128.032
127.539
127.240
124.256
123.986
123.806
123.662
118.378
114.836
77.255
77.000
76.746
70.359
52.360
21.305
f1 (ppm)

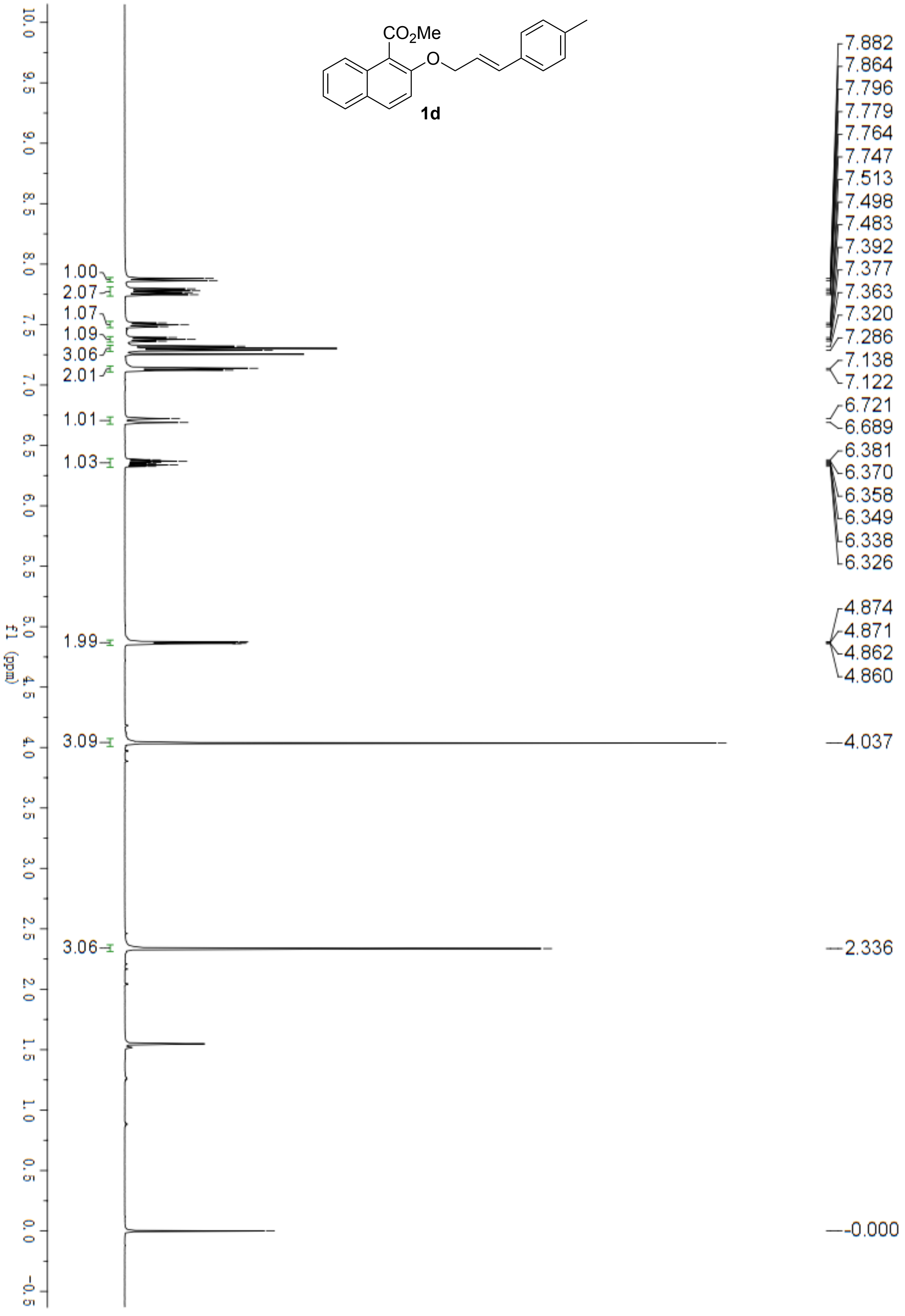

CO2Me
O
1d
7.882
7.864
7.796
7.779
7.764
7.747
7.513
7.498
7.483
7.392
7.377
7.363
7.320
7.286
7.138
7.122
6.721
6.689
6.381
6.370
6.358
6.349
6.338
6.326
4.874
4.871
4.862
4.860
4.037
2.336
-0.000
1.00
2.07
1.07
1.09
3.06
2.01
1.01
1.03
1.99
3.09
3.06
f1 (ppm)
10.0 9.5 9.0 8.5 8.0 7.5 7.0 6.5 6.0 5.5 5.0 4.5 4.0 3.5 3.0 2.5 2.0 1.5 1.0 0.5 0.0 -0.5

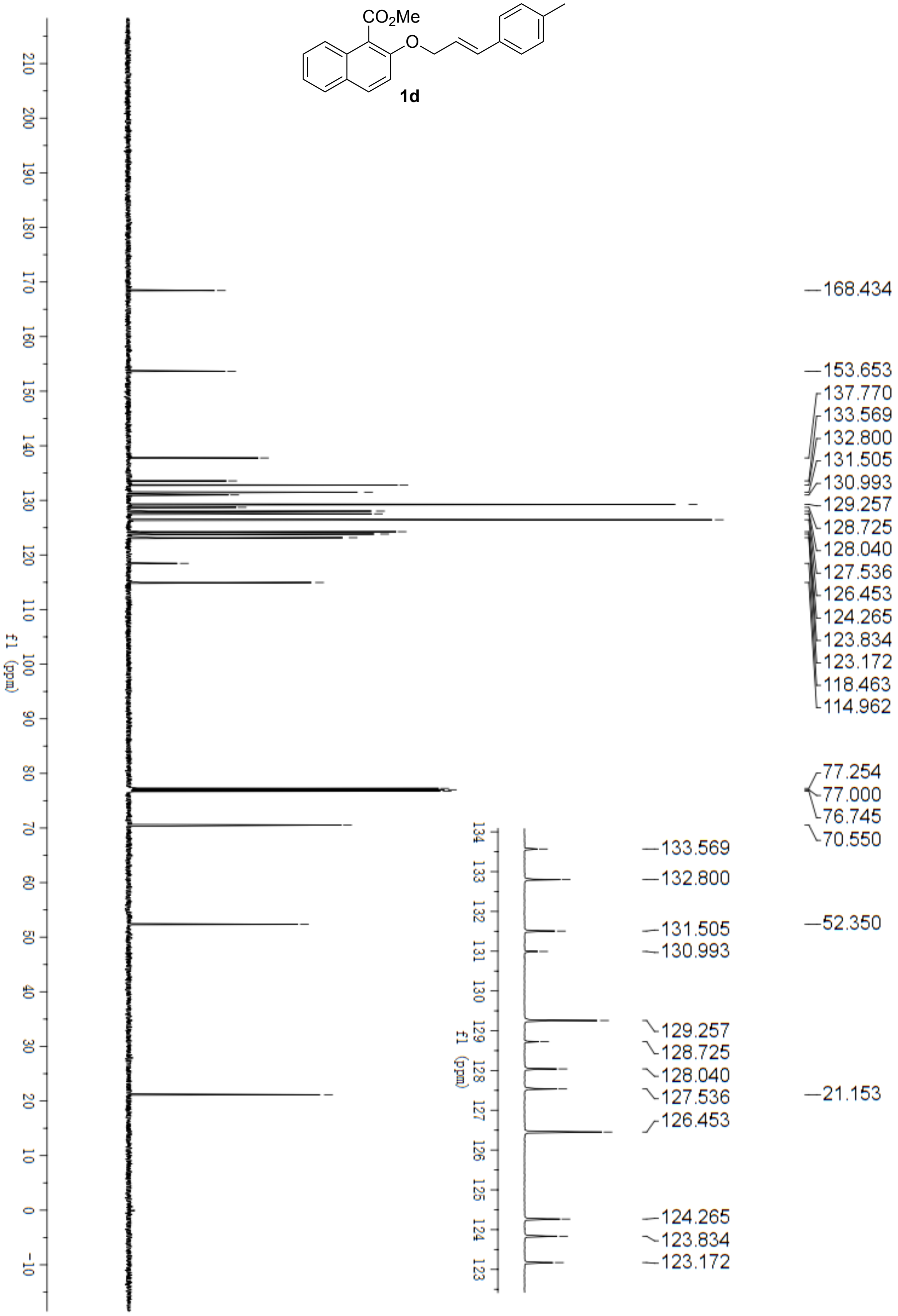
CO2Me
O
1d
f1 (ppm)
168.434
153.653
137.770
133.569
132.800
131.505
130.993
129.257
128.725
128.040
127.536
126.453
124.265
123.834
123.172
118.463
114.962
77.254
77.000
76.745
70.550
52.350
21.153
133.569
132.800
131.505
130.993
129.257
128.725
128.040
127.536
126.453
124.265
123.834
123.172

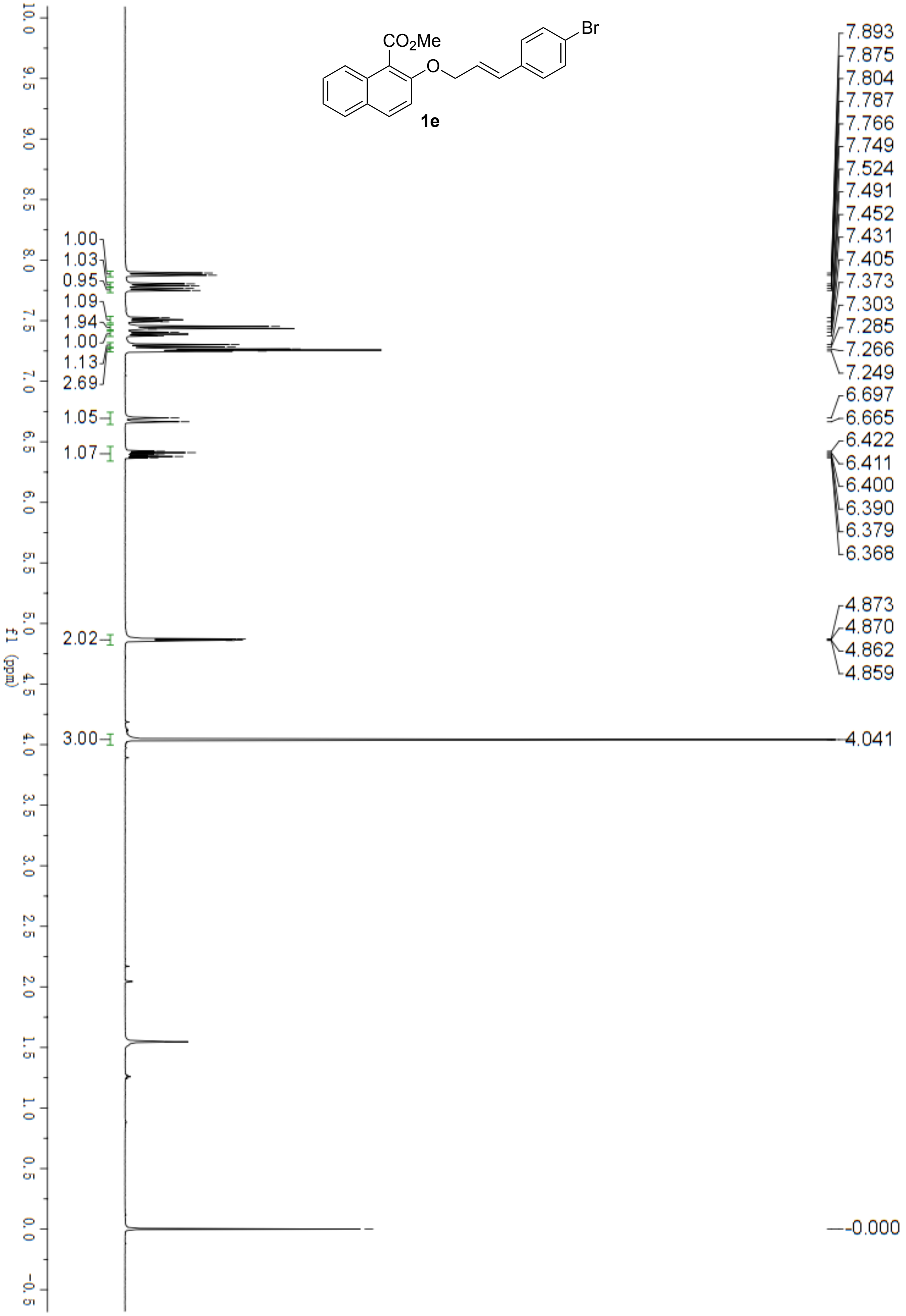
CO2Me
O
Br
1e
7.893
7.875
7.804
7.787
7.766
7.749
7.524
7.491
7.452
7.431
7.405
7.373
7.303
7.285
7.266
7.249
6.697
6.665
6.422
6.411
6.400
6.390
6.379
6.368
4.873
4.870
4.862
4.859
4.041
-0.000
1.00
1.03
0.95
1.09
1.94
1.00
1.13
2.69
1.05
1.07
2.02
3.00
f1 (ppm)

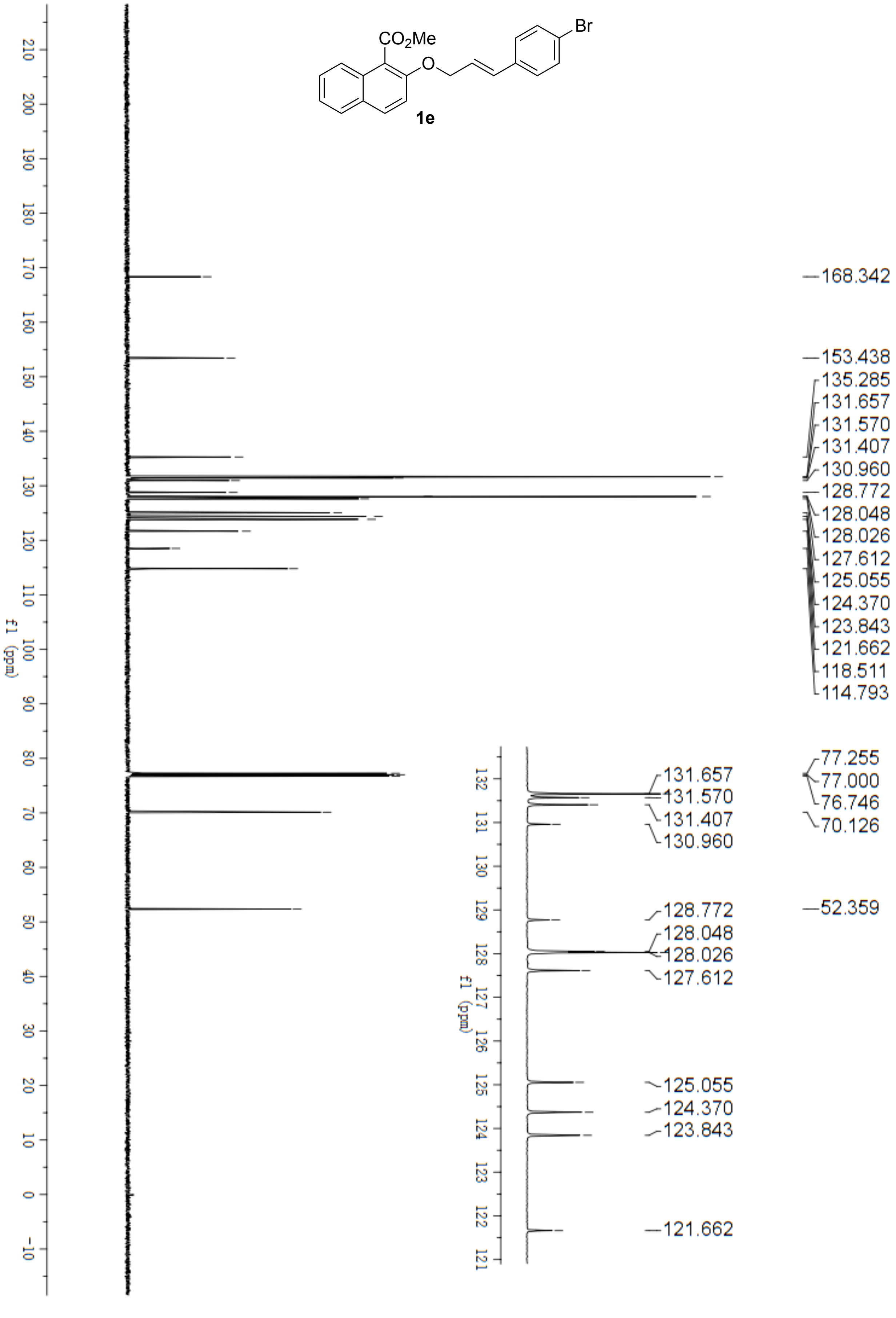
CO2Me
Br
O
1e
168.342
153.438
135.285
131.657
131.570
131.407
130.960
128.772
128.048
128.026
127.612
125.055
124.370
123.843
121.662
118.511
114.793
77.255
77.000
76.746
70.126
52.359
f1 (ppm)

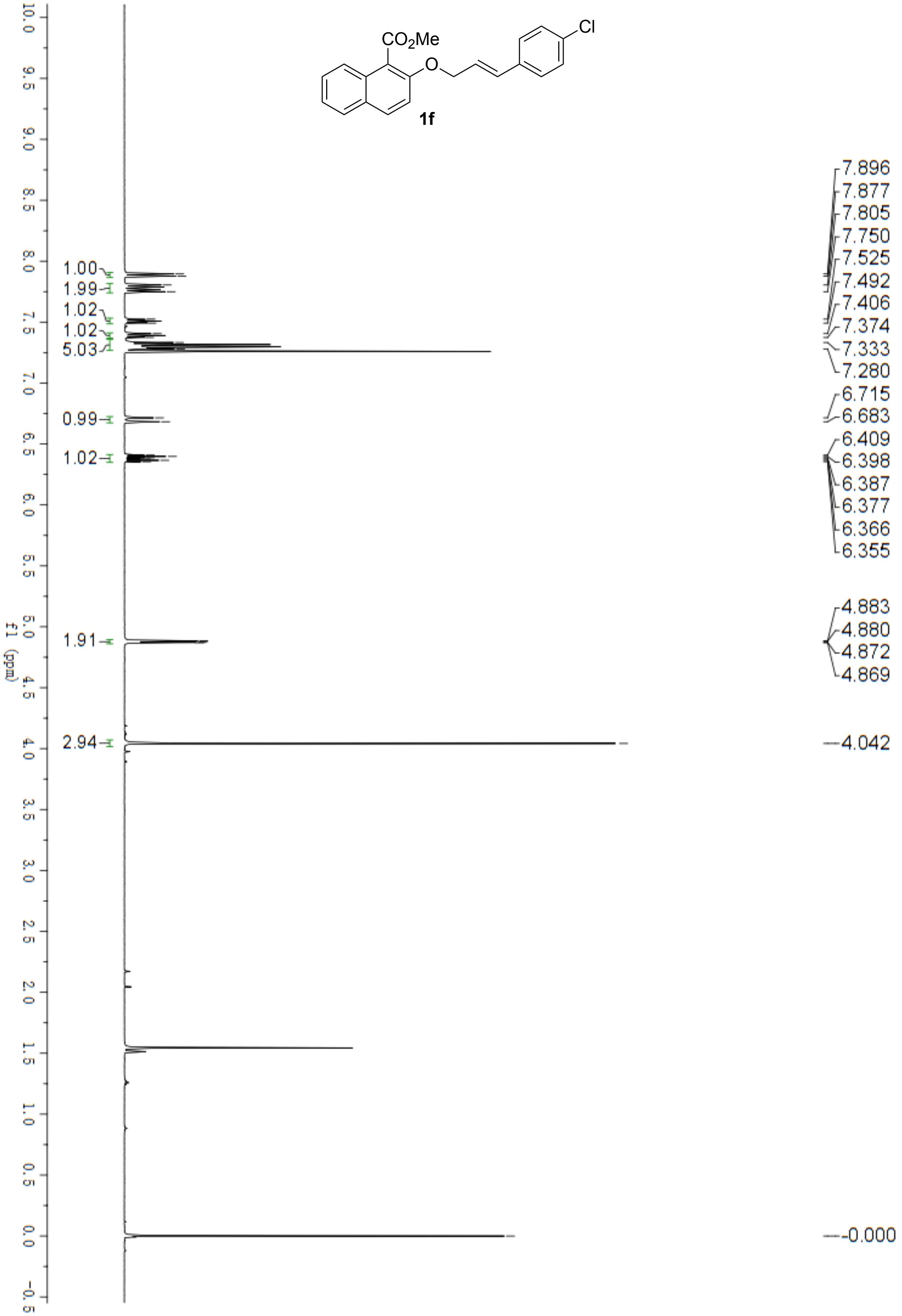
CO2Me
Cl
O
1f
7.896
7.877
7.805
7.750
7.525
7.492
7.406
7.374
7.333
7.280
6.715
6.683
6.409
6.398
6.387
6.377
6.366
6.355
4.883
4.880
4.872
4.869
4.042
-0.000
1.00
1.99
1.02
1.02
5.03
0.99
1.02
1.91
2.94
f1 (ppm)

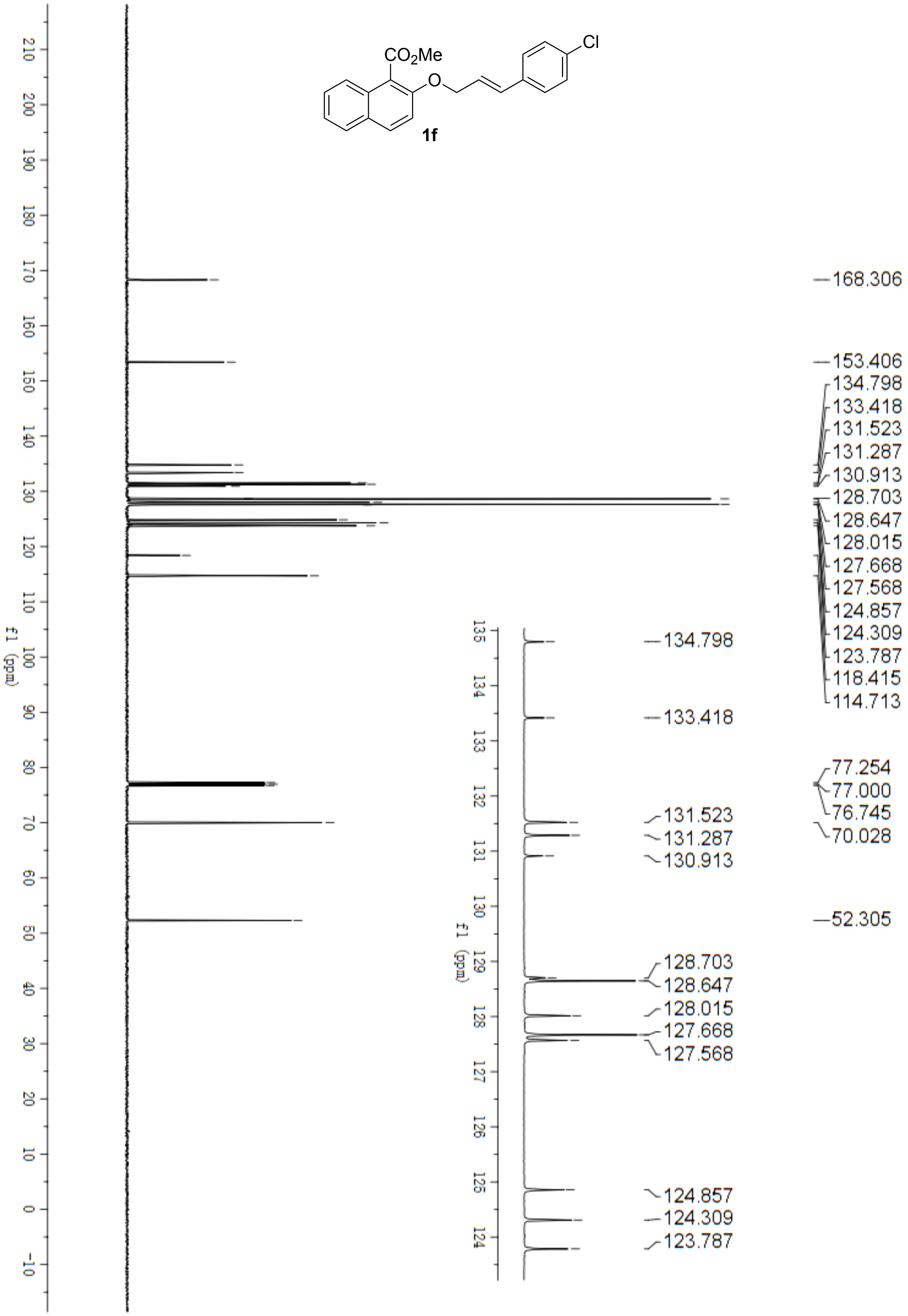
CO2Me
O
Cl
1f
168.306
153.406
134.798
133.418
131.523
131.287
130.913
128.703
128.647
128.015
127.668
127.568
124.857
124.309
123.787
118.415
114.713
77.254
77.000
76.745
70.028
52.305
f1 (ppm)

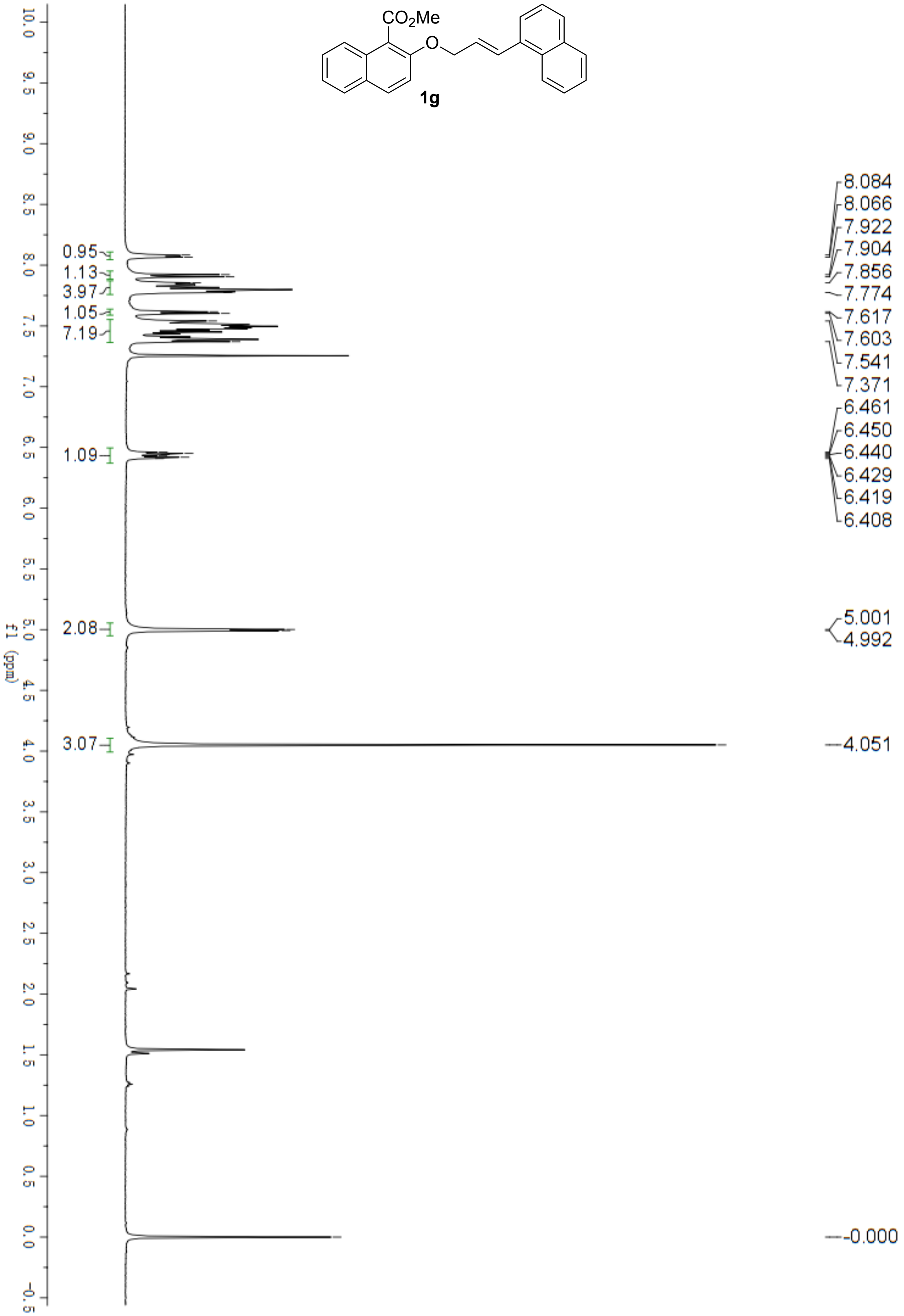
CO2Me
O
1g
8.084
8.066
7.922
7.904
7.856
7.774
7.617
7.603
7.541
7.371
6.461
6.450
6.440
6.429
6.419
6.408
5.001
4.992
4.051
-0.000
0.95
1.13
3.97
1.05
7.19
1.09
2.08
3.07
f1 (ppm)

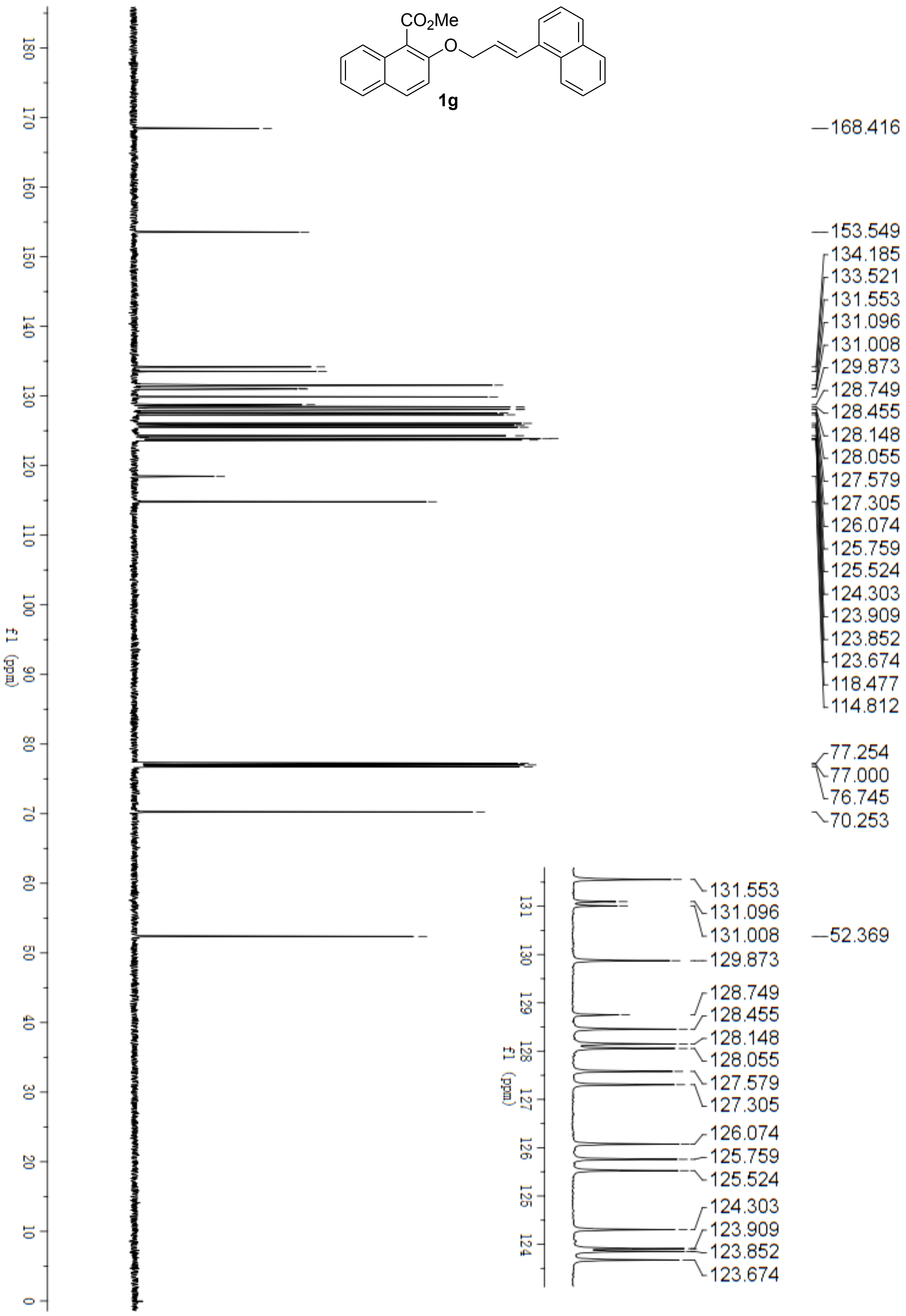
CO2Me
O
1g
168.416
153.549
134.185
133.521
131.553
131.096
131.008
129.873
128.749
128.455
128.148
128.055
127.579
127.305
126.074
125.759
125.524
124.303
123.909
123.852
123.674
118.477
114.812
77.254
77.000
76.745
70.253
52.369
f1 (ppm)

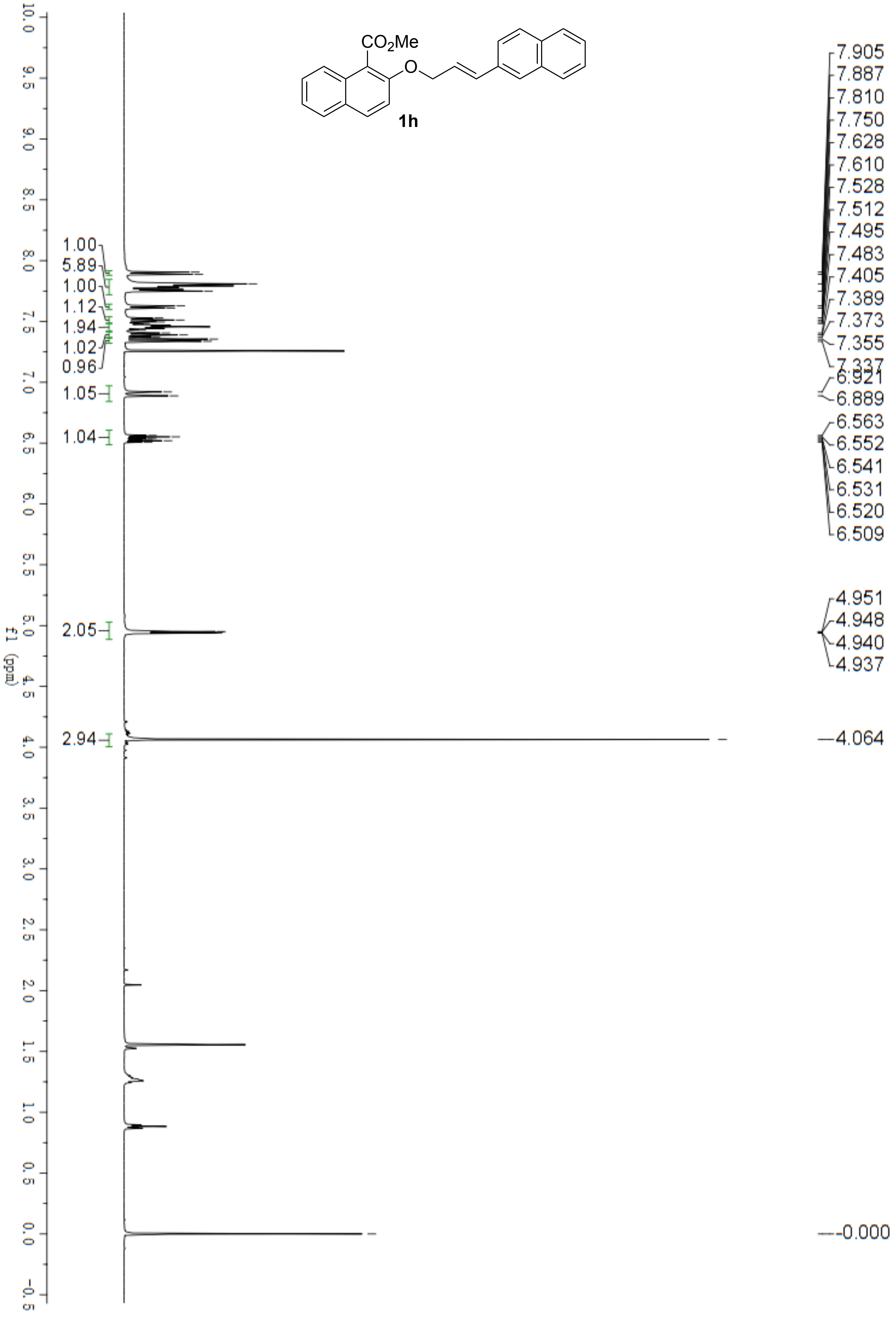
CO2Me
O
1h
7.905
7.887
7.810
7.750
7.628
7.610
7.528
7.512
7.495
7.483
7.405
7.389
7.373
7.355
7.337
6.921
6.889
6.563
6.552
6.541
6.531
6.520
6.509
4.951
4.948
4.940
4.937
4.064
-0.000
1.00
5.89
1.00
1.12
1.94
1.02
0.96
1.05
1.04
2.05
2.94
f1 (ppm)

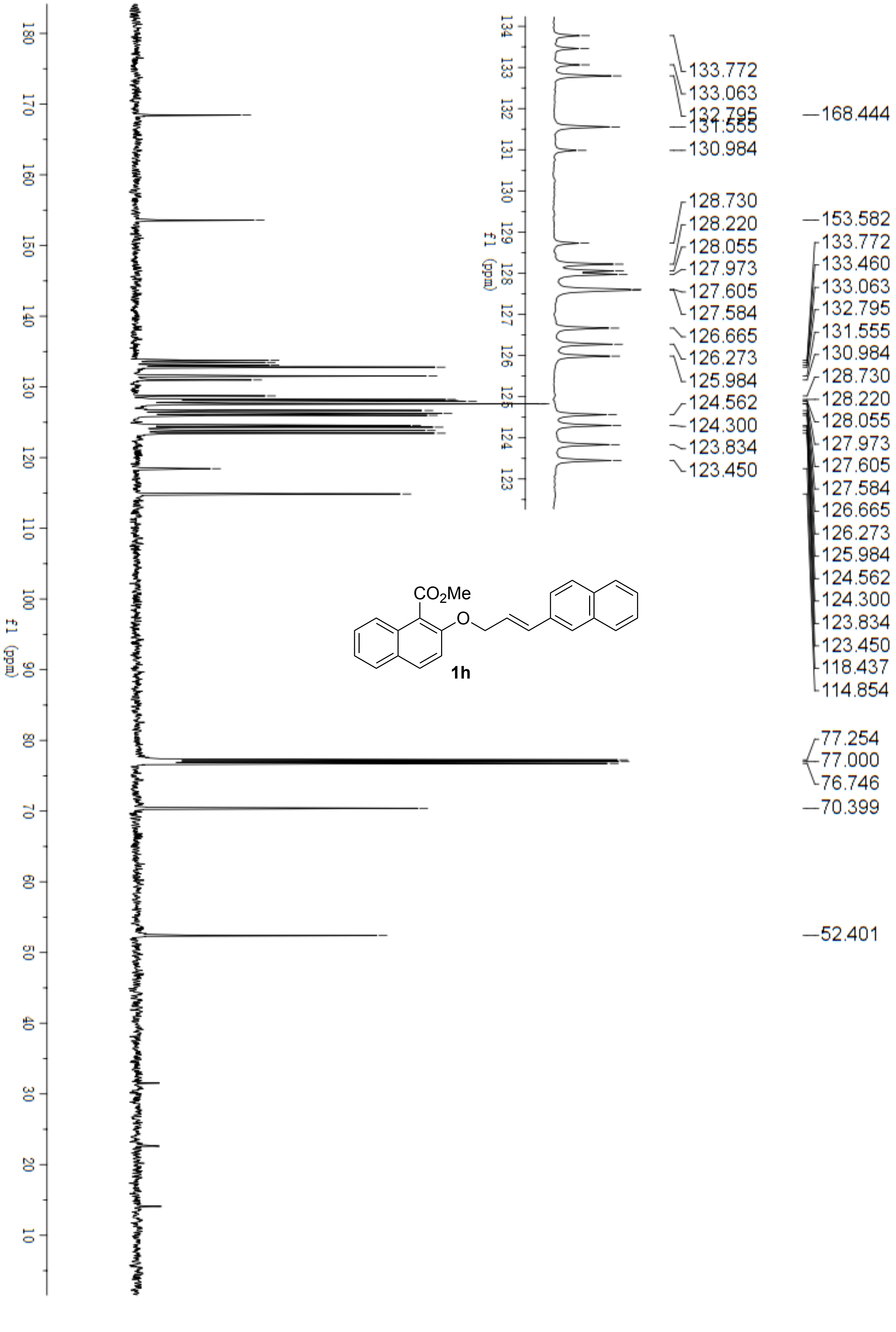
133.772
133.063
132.795
131.555
130.984
128.730
128.220
128.055
127.973
127.605
127.584
126.665
126.273
125.984
124.562
124.300
123.834
123.450
f1 (ppm)
168.444
153.582
133.772
133.460
133.063
132.795
131.555
130.984
128.730
128.220
128.055
127.973
127.605
127.584
126.665
126.273
125.984
124.562
124.300
123.834
123.450
118.437
114.854
77.254
77.000
76.746
70.399
52.401
CO2Me
O
1h
f1 (ppm)

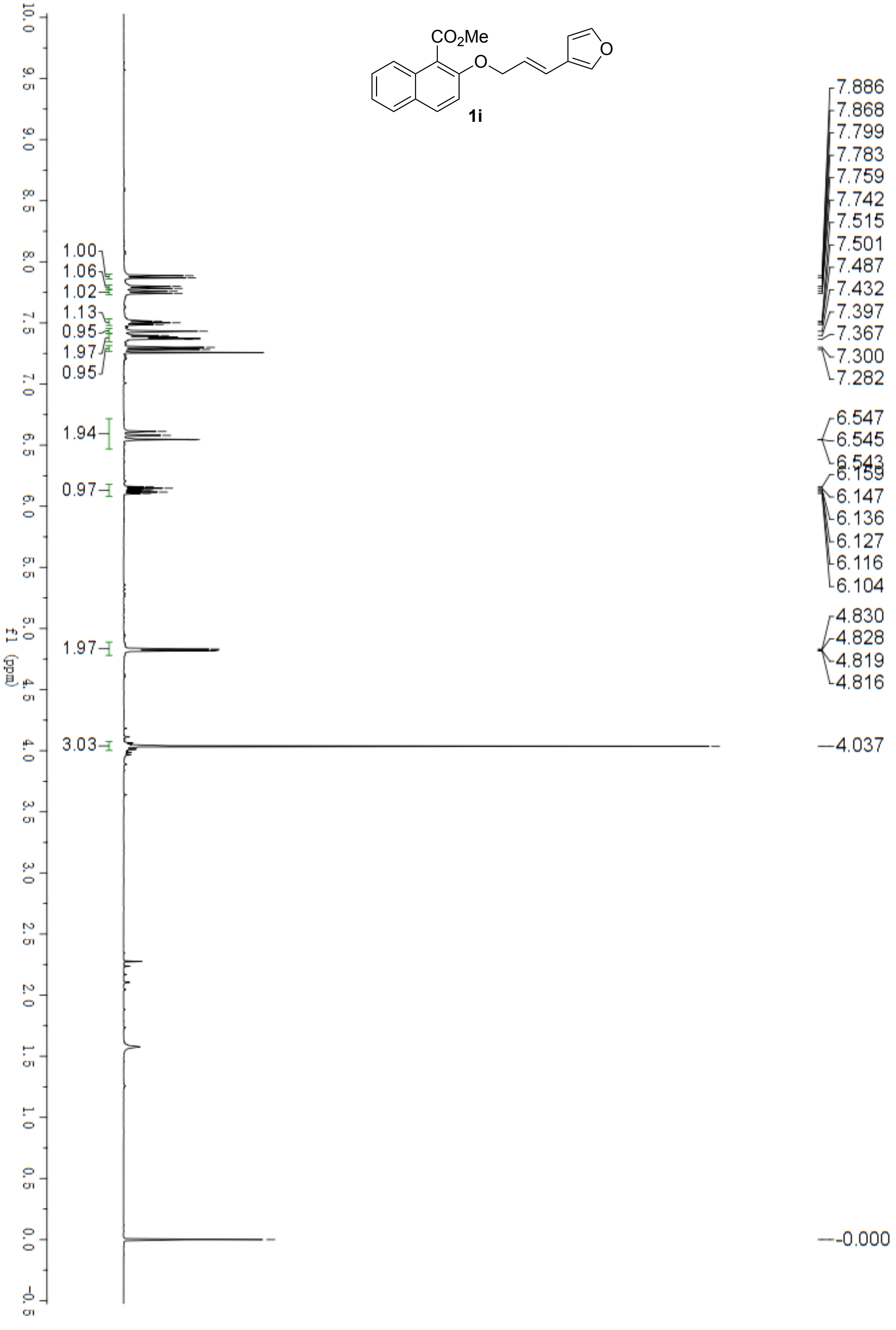

CO2Me
O
O
1i
7.886
7.868
7.799
7.783
7.759
7.742
7.515
7.501
7.487
7.432
7.397
7.367
7.300
7.282
6.547
6.545
6.543
6.159
6.147
6.136
6.127
6.116
6.104
4.830
4.828
4.819
4.816
4.037
-0.000
1.00
1.06
1.02
1.13
0.95
1.97
0.95
1.94
0.97
1.97
3.03
f1 (ppm)
10.0
9.5
9.0
8.5
8.0
7.5
7.0
6.5
6.0
5.5
5.0
4.5
4.0
3.5
3.0
2.5
2.0
1.5
1.0
0.5
0.0
-0.5

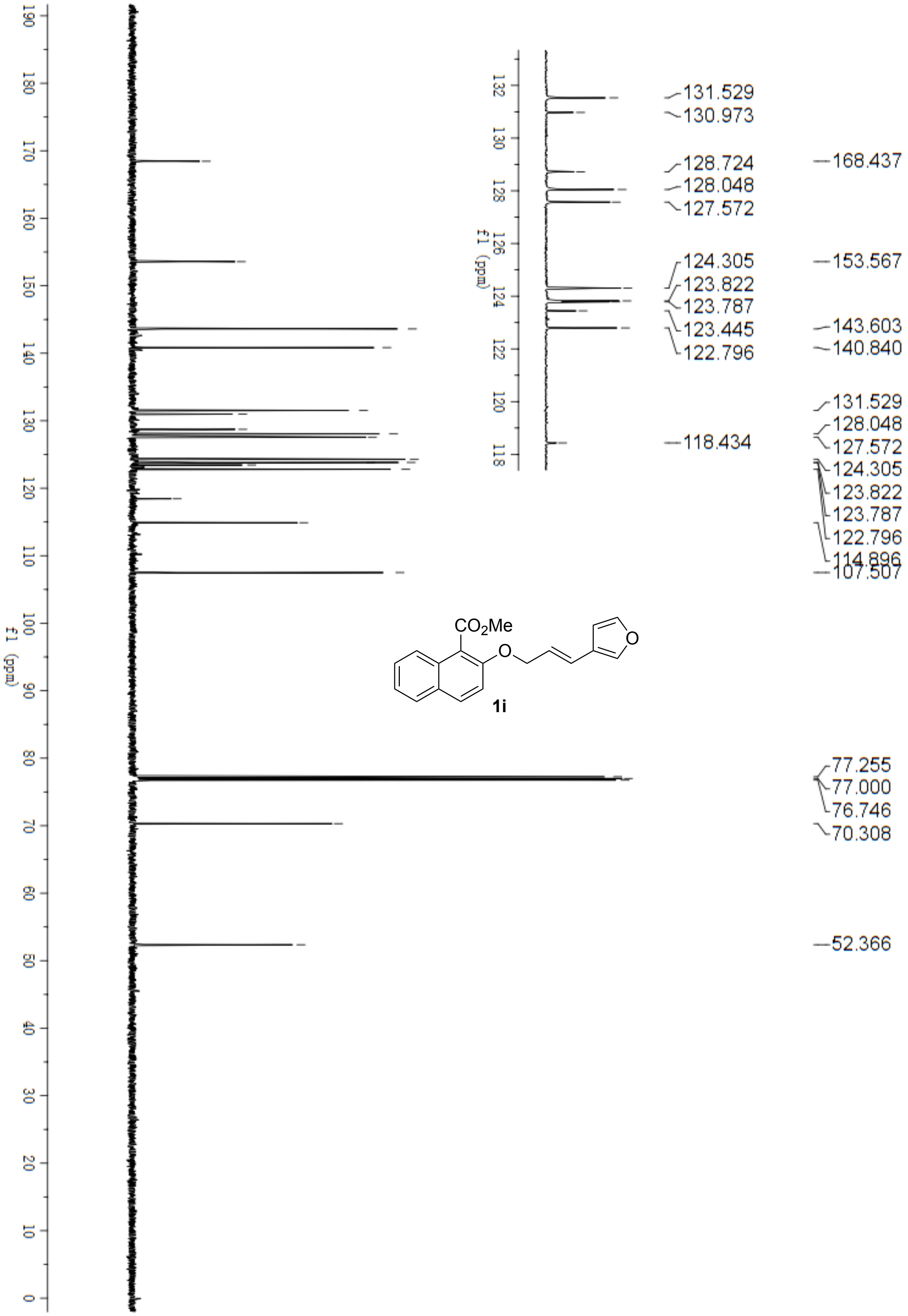
CO2Me
O
O
1i
131.529
130.973
128.724
128.048
127.572
124.305
123.822
123.787
123.445
122.796
118.434
168.437
153.567
143.603
140.840
131.529
128.048
127.572
124.305
123.822
123.787
122.796
114.896
107.507
77.255
77.000
76.746
70.308
52.366
f1 (ppm)

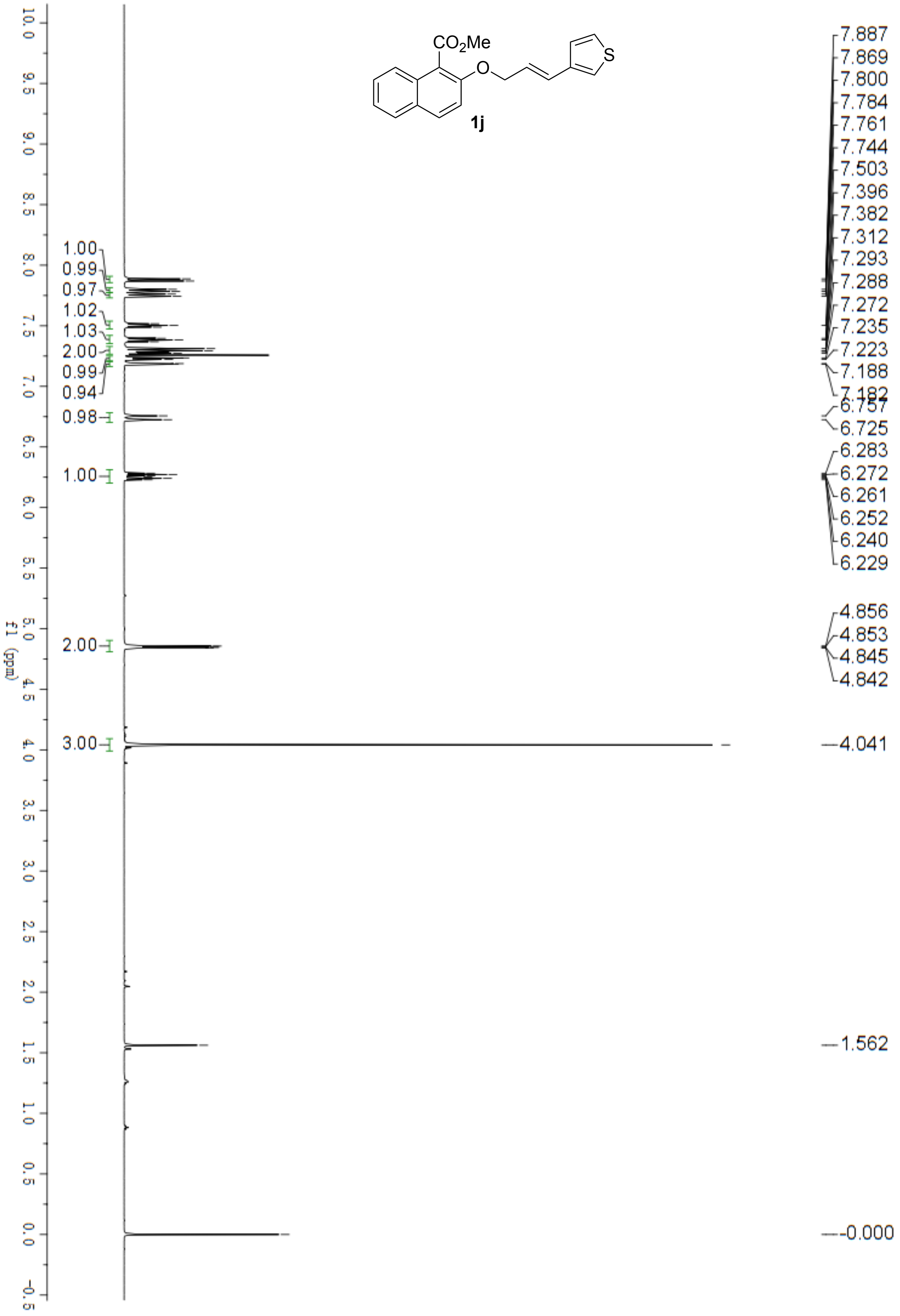
CO2Me
O
S
1j
7.887
7.869
7.800
7.784
7.761
7.744
7.503
7.396
7.382
7.312
7.293
7.288
7.272
7.235
7.223
7.188
7.182
6.757
6.725
6.283
6.272
6.261
6.252
6.240
6.229
4.856
4.853
4.845
4.842
4.041
1.562
-0.000
1.00
0.99
0.97
1.02
1.03
2.00
0.99
0.94
0.98
1.00
2.00
3.00
f1 (ppm)

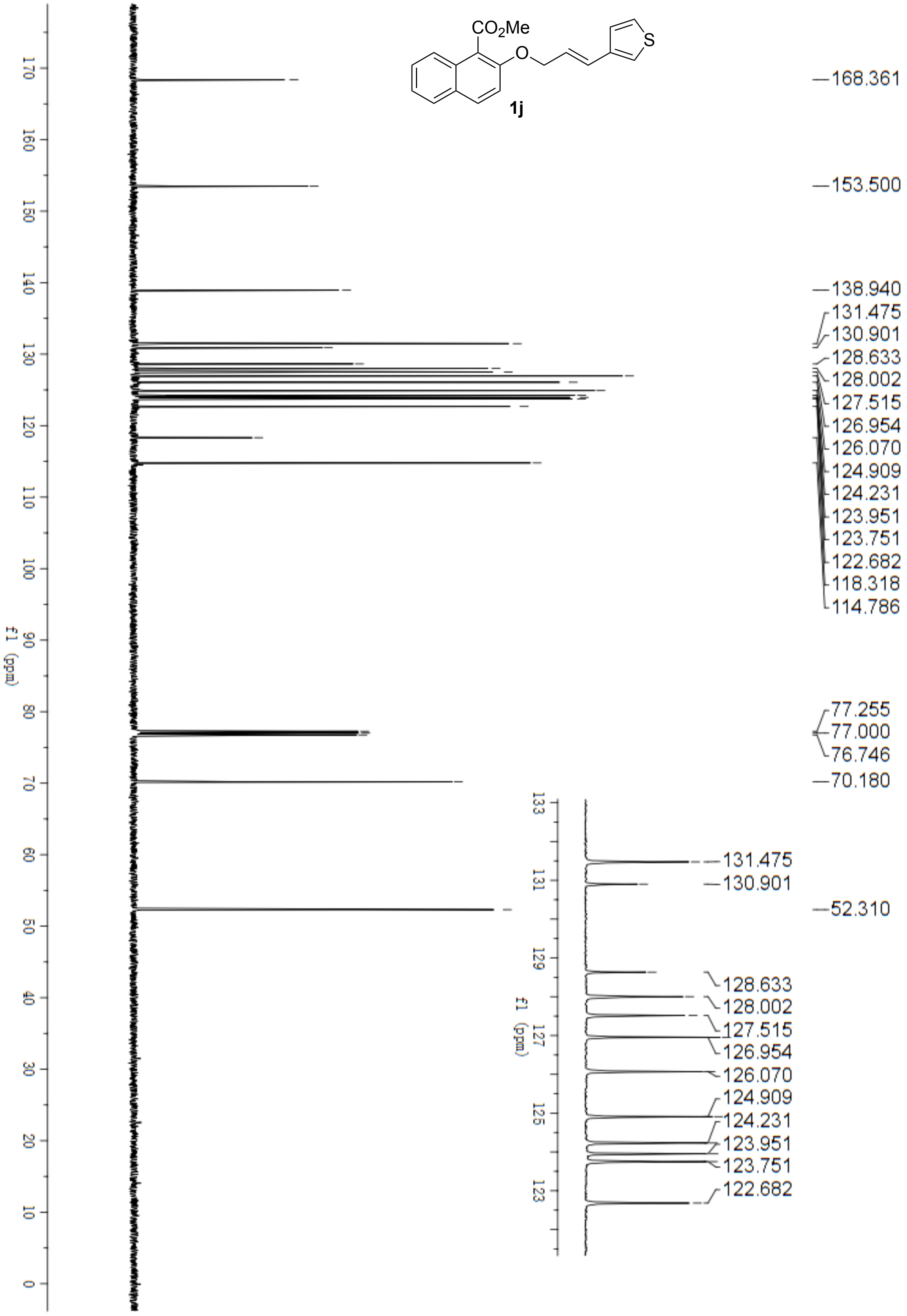
CO2Me
O
S
1j
168.361
153.500
138.940
131.475
130.901
128.633
128.002
127.515
126.954
126.070
124.909
124.231
123.951
123.751
122.682
118.318
114.786
77.255
77.000
76.746
70.180
52.310
f1 (ppm)

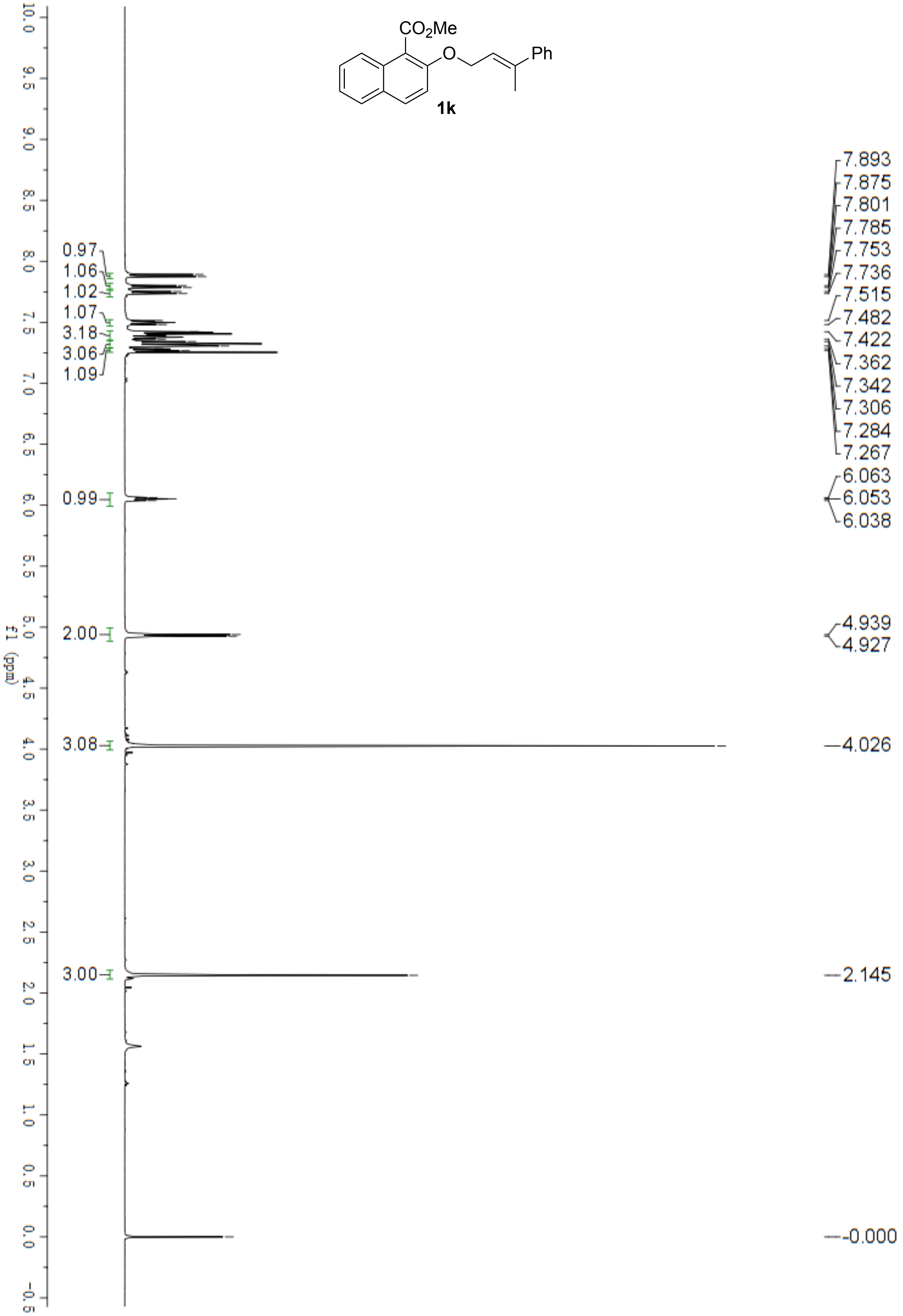
CO2Me
O
Ph
1k
0.97
1.06
1.02
1.07
3.18
3.06
1.09
0.99
2.00
3.08
3.00
7.893
7.875
7.801
7.785
7.753
7.736
7.515
7.482
7.422
7.362
7.342
7.306
7.284
7.267
6.063
6.053
6.038
4.939
4.927
4.026
2.145
-0.000
f1 (ppm)
10.0
9.5
9.0
8.5
8.0
7.5
7.0
6.5
6.0
5.5
5.0
4.5
4.0
3.5
3.0
2.5
2.0
1.5
1.0
0.5
0.0
-0.5

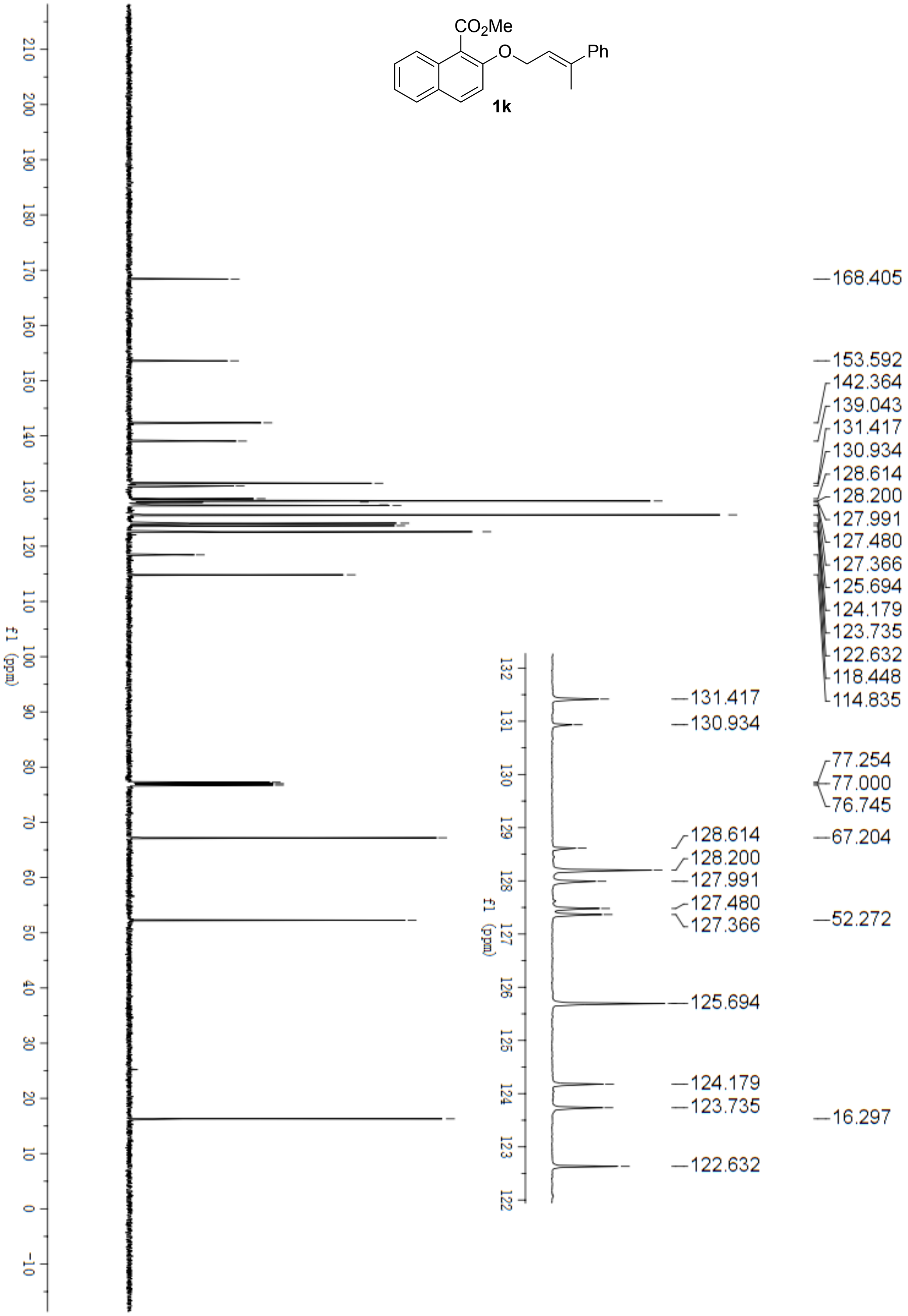
CO2Me
O
Ph
1k
168.405
153.592
142.364
139.043
131.417
130.934
128.614
128.200
127.991
127.480
127.366
125.694
124.179
123.735
122.632
118.448
114.835
77.254
77.000
76.745
67.204
52.272
16.297
f1 (ppm)

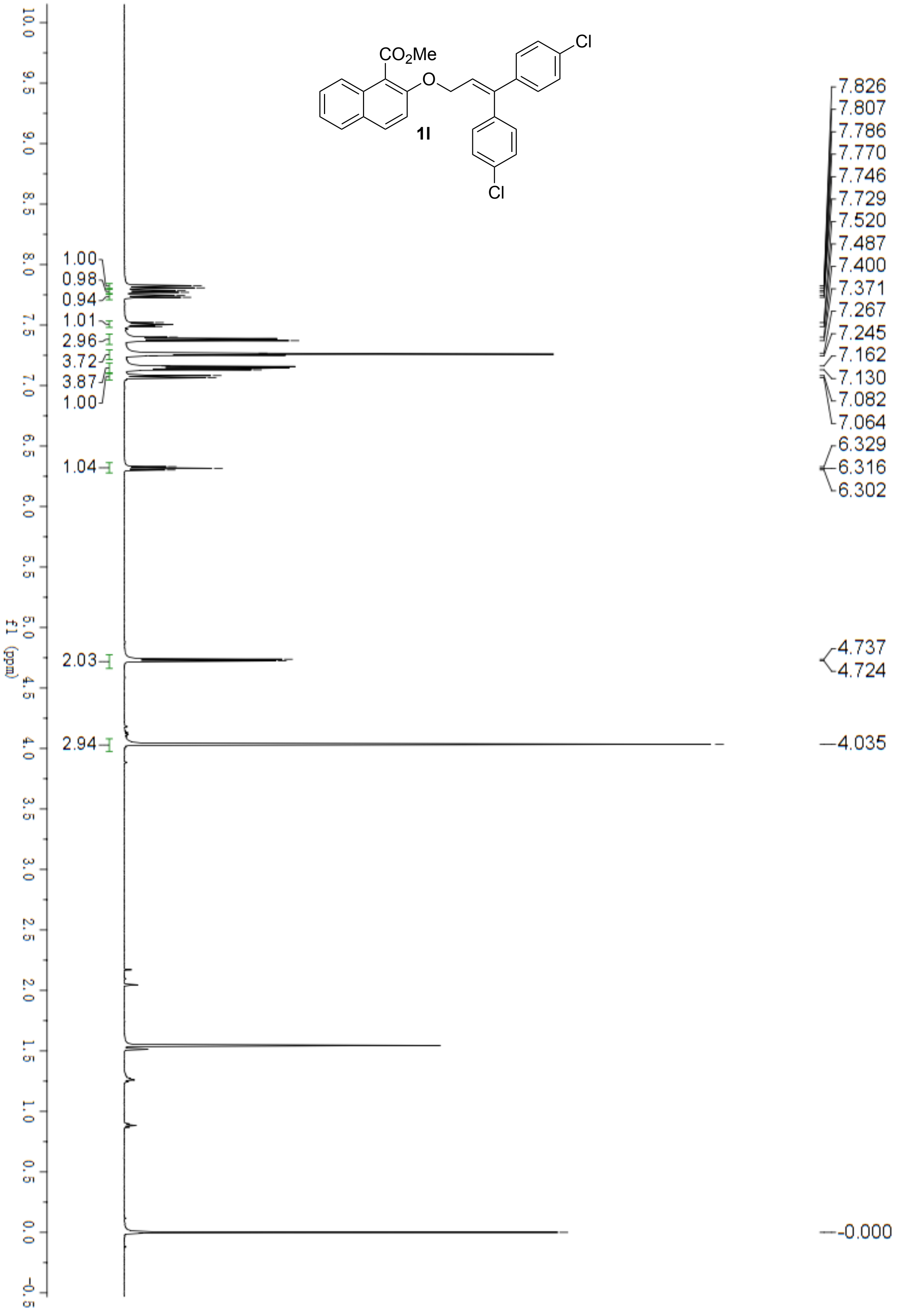
CO2Me
O
Cl
Cl
1l
7.826
7.807
7.786
7.770
7.746
7.729
7.520
7.487
7.400
7.371
7.267
7.245
7.162
7.130
7.082
7.064
6.329
6.316
6.302
4.737
4.724
4.035
-0.000
1.00
0.98
0.94
1.01
2.96
3.72
3.87
1.00
1.04
2.03
2.94
10.0
9.5
9.0
8.5
8.0
7.5
7.0
6.5
6.0
5.5
5.0
4.5
4.0
3.5
3.0
2.5
2.0
1.5
1.0
0.5
0.0
-0.5
f1 (ppm)

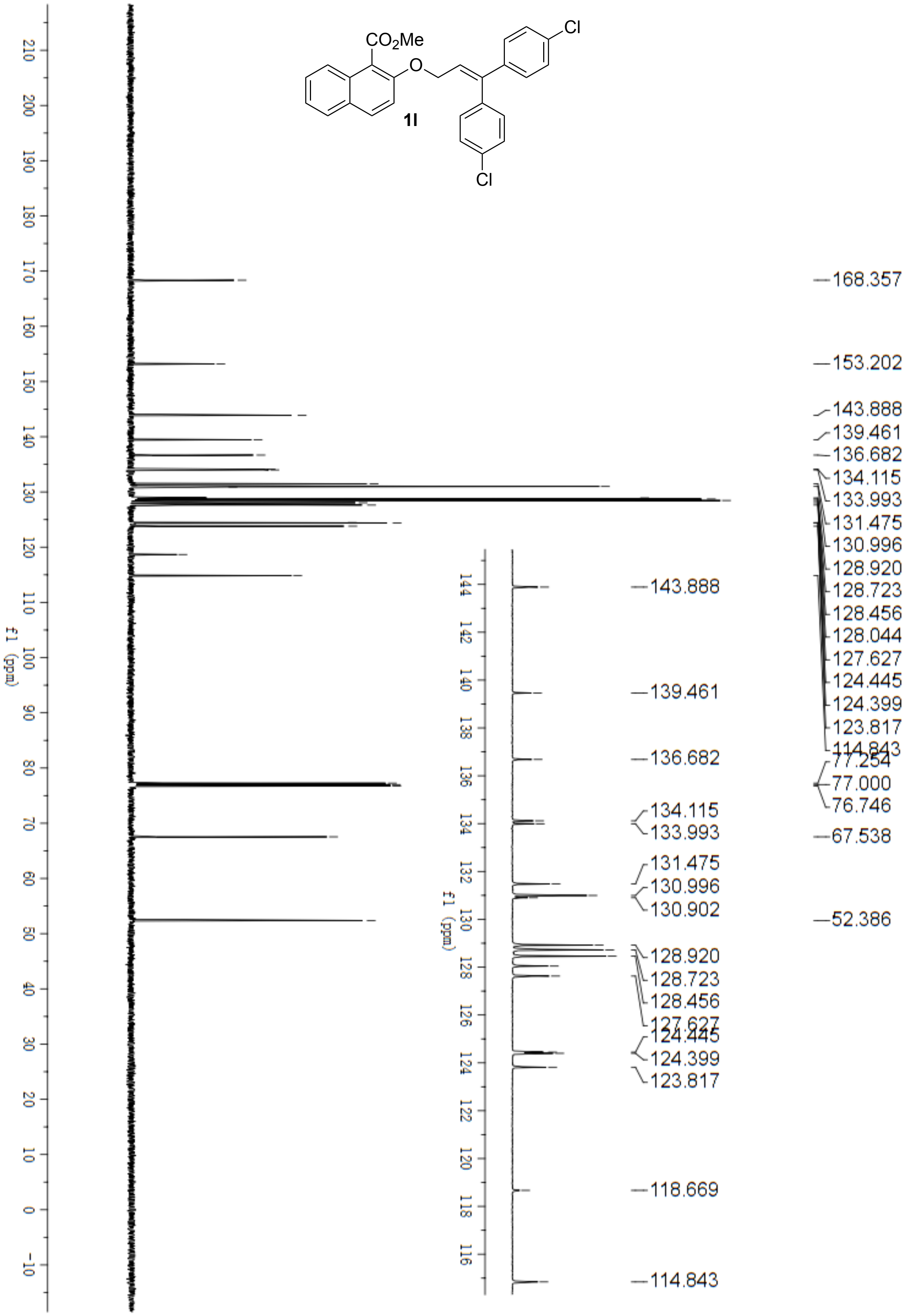
CO2Me
O
Cl
Cl
1l
168.357
153.202
143.888
139.461
136.682
134.115
133.993
131.475
130.996
128.920
128.723
128.456
128.044
127.627
124.445
124.399
123.817
114.843
77.254
77.000
76.746
67.538
52.386
143.888
139.461
136.682
134.115
133.993
131.475
130.996
130.902
128.920
128.723
128.456
127.627
124.445
124.399
123.817
118.669
114.843
f1 (ppm)
f1 (ppm)

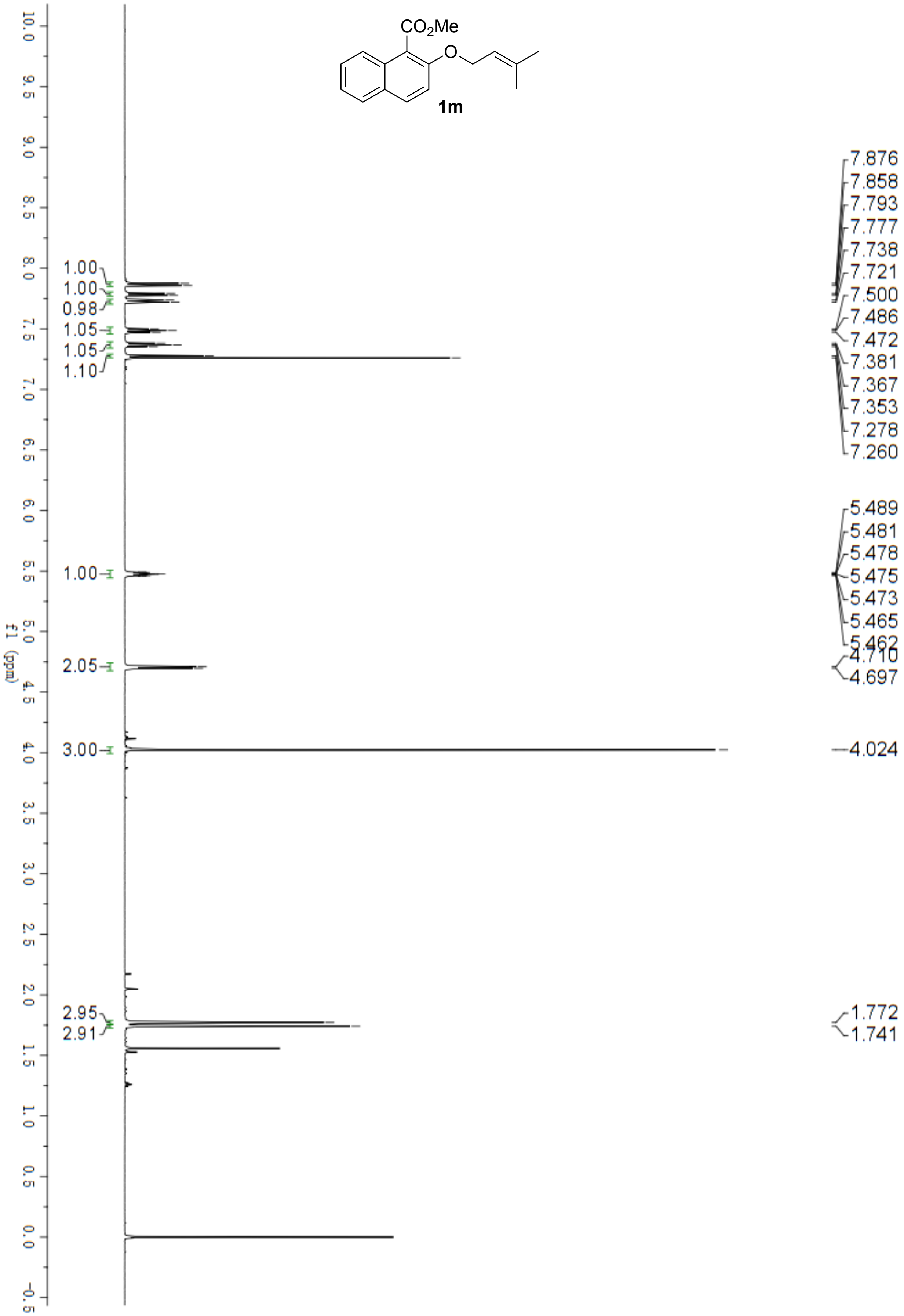
CO2Me
O
1m
7.876
7.858
7.793
7.777
7.738
7.721
7.500
7.486
7.472
7.381
7.367
7.353
7.278
7.260
5.489
5.481
5.478
5.475
5.473
5.465
5.462
4.710
4.697
4.024
1.772
1.741
1.00
1.00
0.98
1.05
1.05
1.10
1.00
2.05
3.00
2.95
2.91
f1 (ppm)

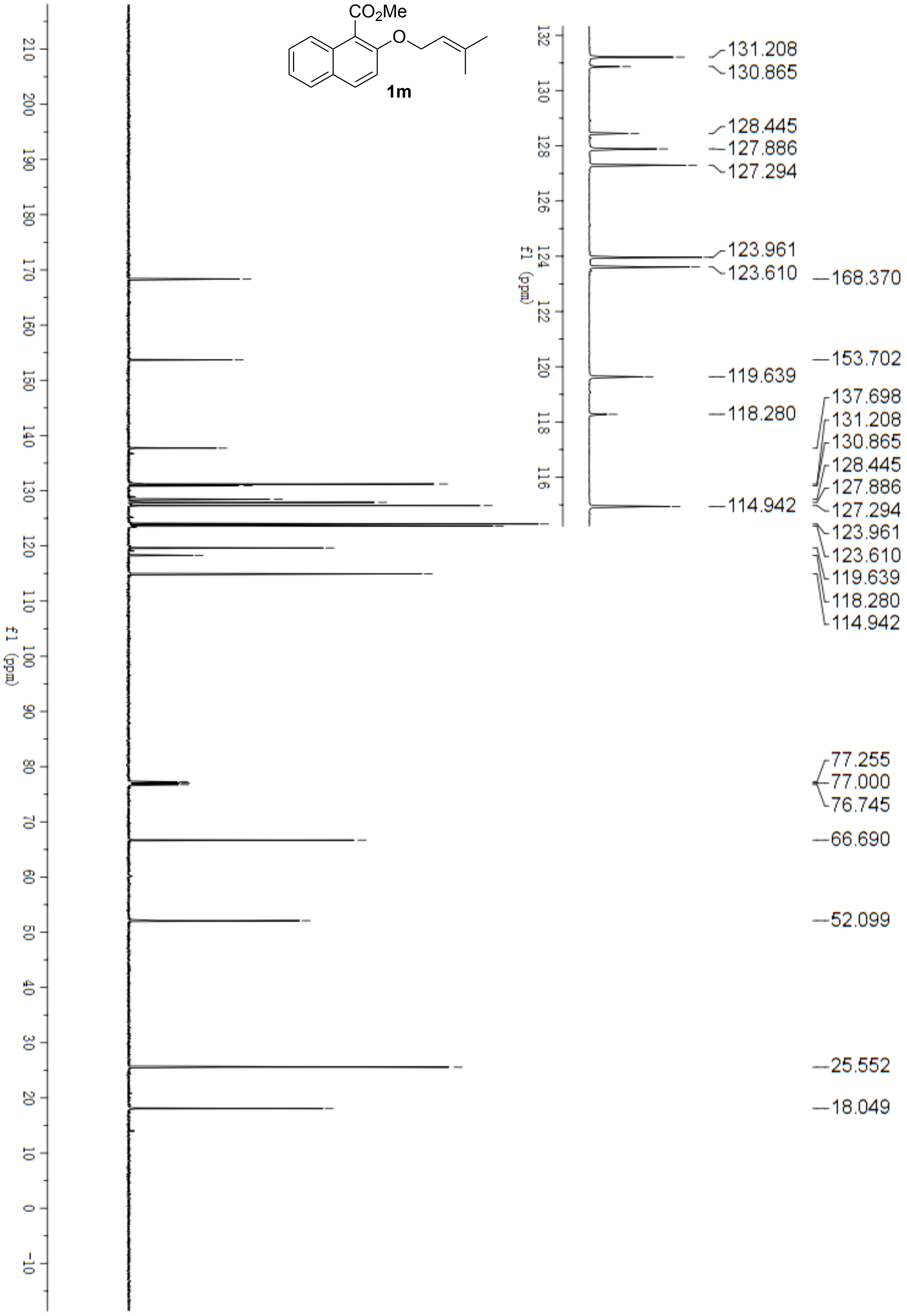
CO2Me
O
1m
131.208
130.865
128.445
127.886
127.294
123.961
123.610
119.639
118.280
114.942
168.370
153.702
137.698
131.208
130.865
128.445
127.886
127.294
123.961
123.610
119.639
118.280
114.942
77.255
77.000
76.745
66.690
52.099
25.552
18.049
f1 (ppm)

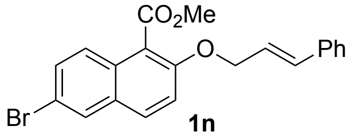
CO2Me
O
Ph
Br

1n

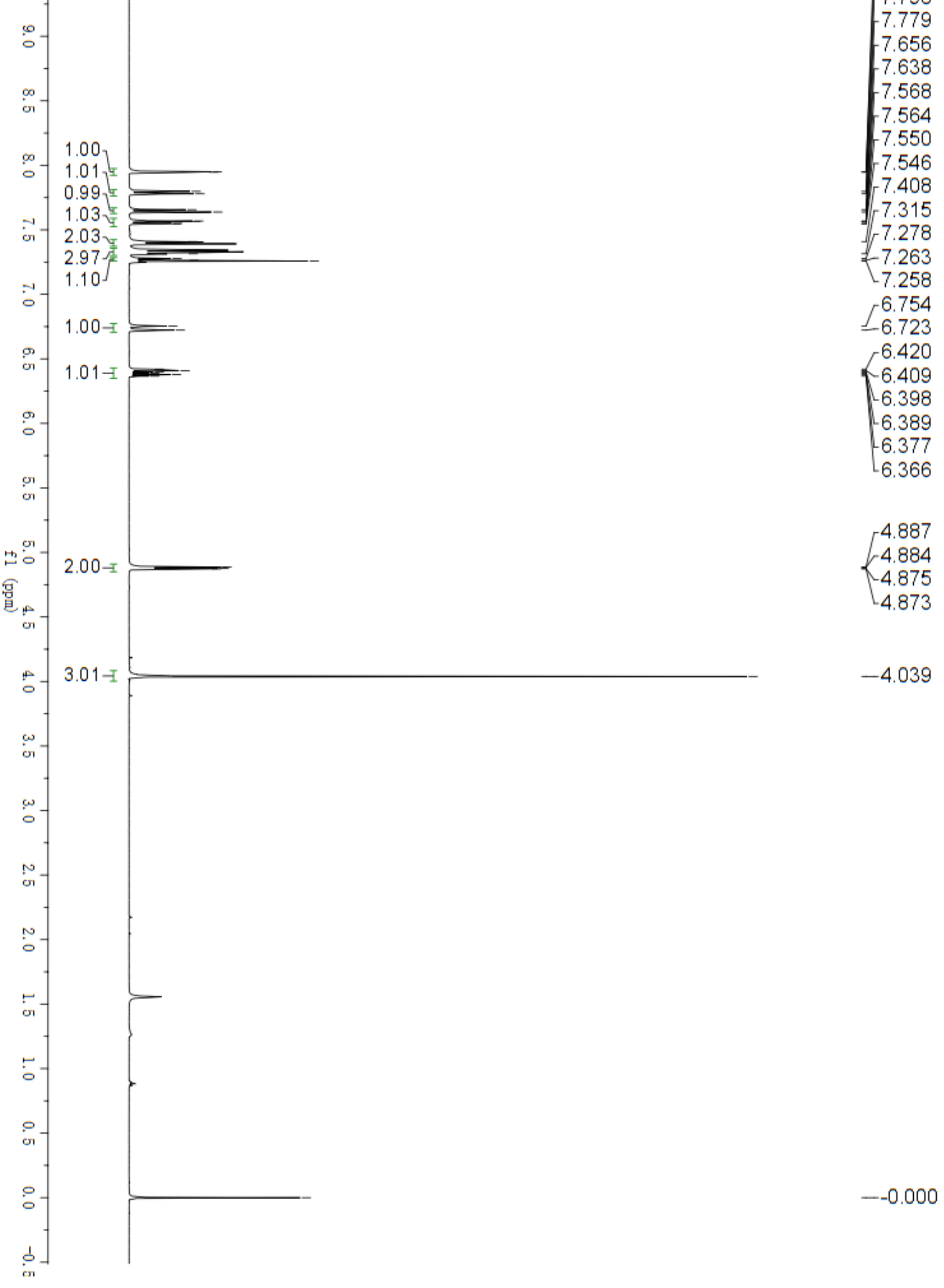
7.950
7.947
7.798
7.779
7.656
7.638
7.568
7.564
7.550
7.546
7.408
7.315
7.278
7.263
7.258
6.754
6.723
6.420
6.409
6.398
6.389
6.377
6.366
4.887
4.884
4.875
4.873
4.039
-0.000
1.00
1.01
0.99
1.03
2.03
2.97
1.10
1.00
1.01
2.00
3.01
f1 (ppm)

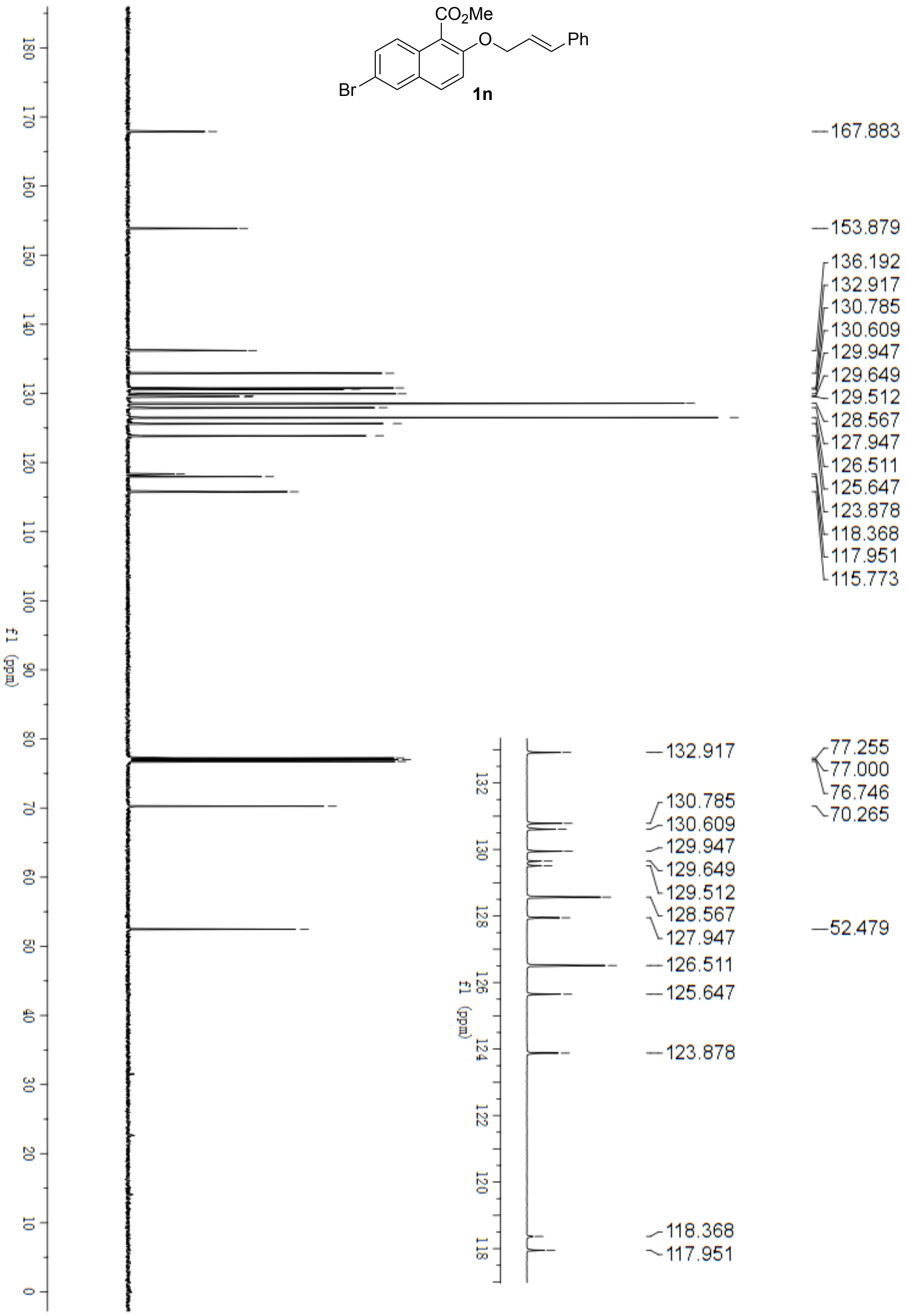
CO2Me
O
Ph
Br
1n
167.883
153.879
136.192
132.917
130.785
130.609
129.947
129.649
129.512
128.567
127.947
126.511
125.647
123.878
118.368
117.951
115.773
77.255
77.000
76.746
70.265
52.479
f1 (ppm)

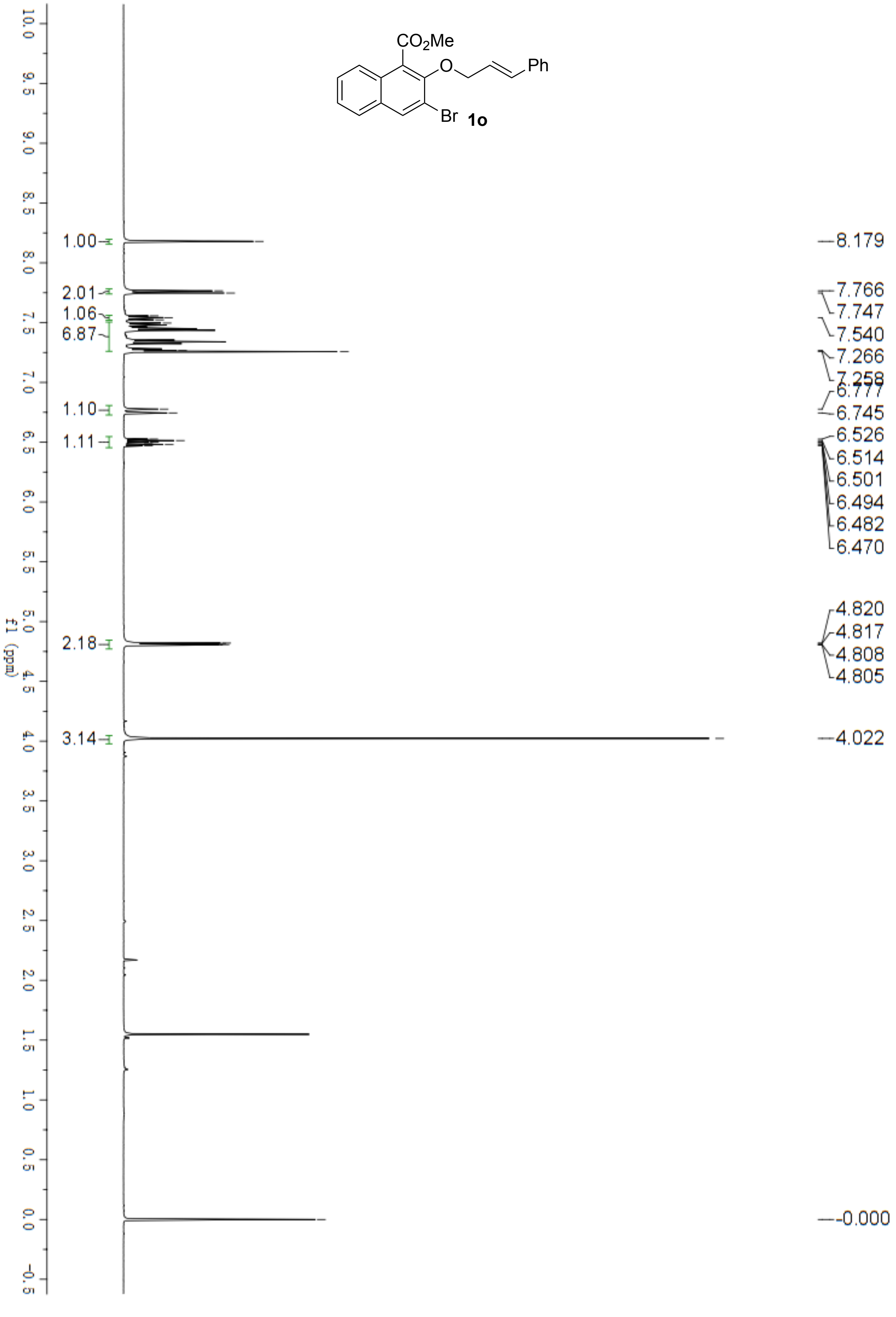
CO2Me
O
Ph
Br
1o
8.179
7.766
7.747
7.540
7.266
7.258
6.777
6.745
6.526
6.514
6.501
6.494
6.482
6.470
4.820
4.817
4.808
4.805
4.022
-0.000
1.00
2.01
1.06
6.87
1.10
1.11
2.18
3.14
f1 (ppm)

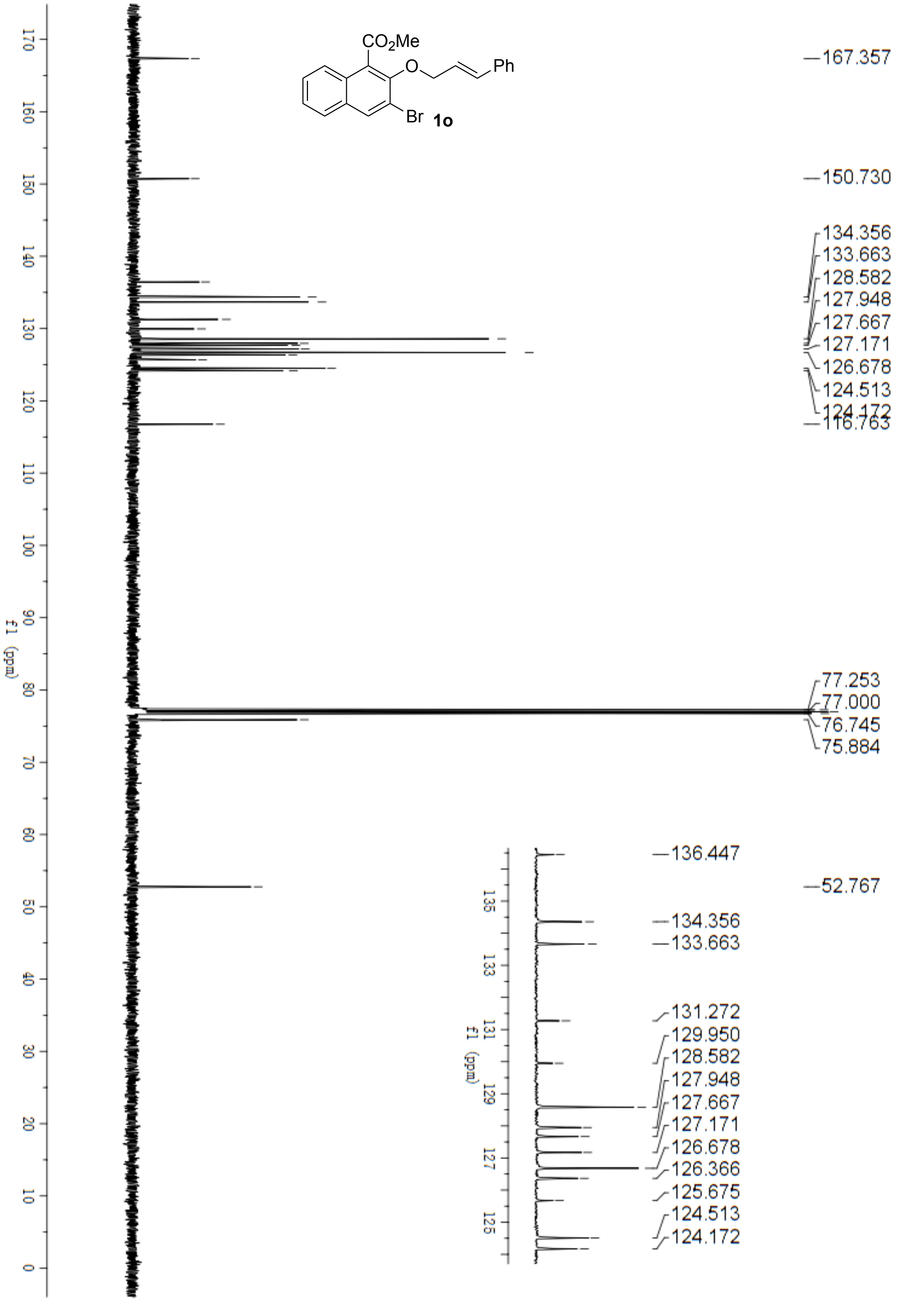
CO2Me
O
Ph
Br
1o
167.357
150.730
134.356
133.663
128.582
127.948
127.667
127.171
126.678
124.513
124.172
116.763
77.253
77.000
76.745
75.884
52.767
136.447
134.356
133.663
131.272
129.950
128.582
127.948
127.667
127.171
126.678
126.366
125.675
124.513
124.172
f1 (ppm)

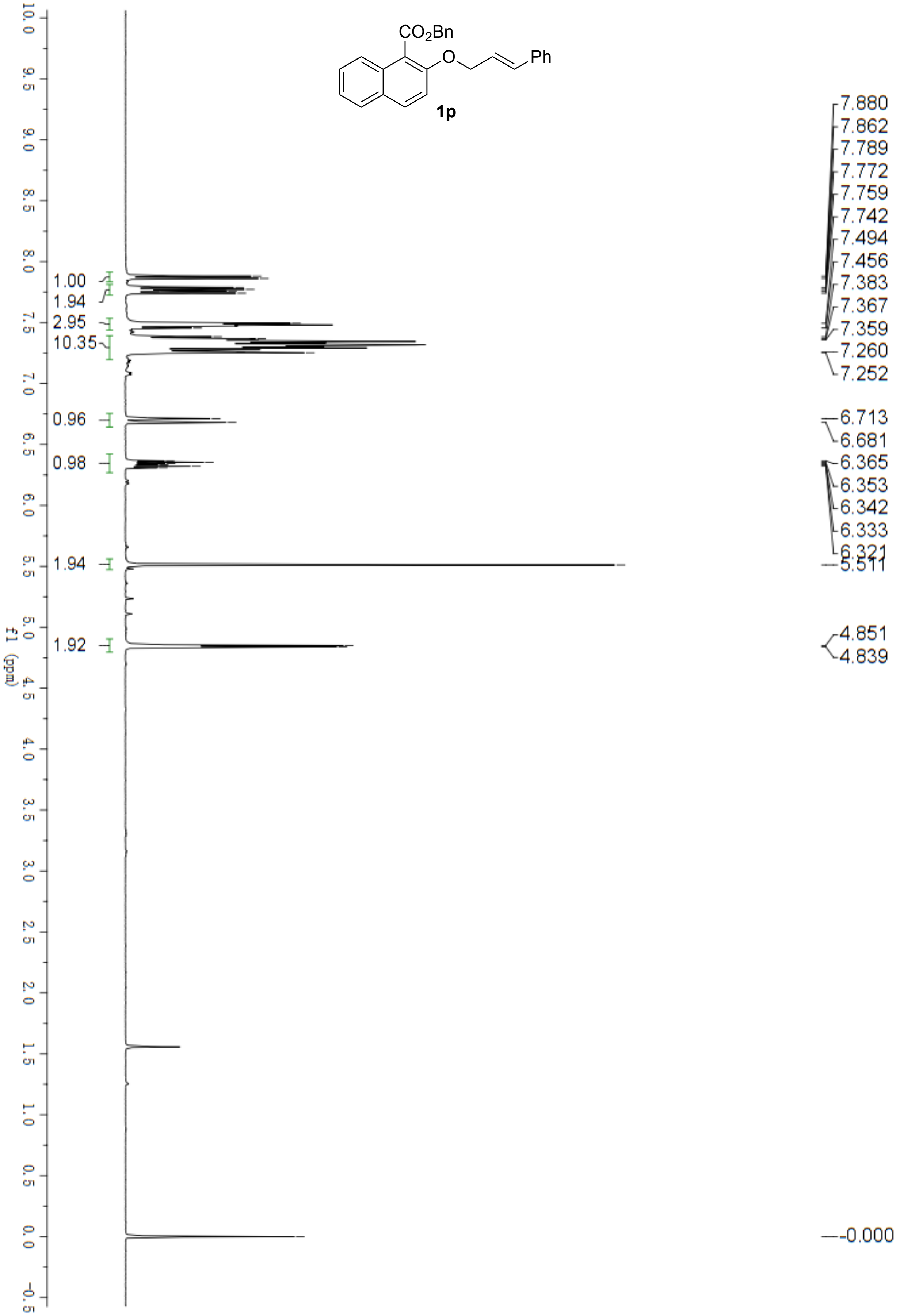
CO2Bn
O
Ph
1p
7.880
7.862
7.789
7.772
7.759
7.742
7.494
7.456
7.383
7.367
7.359
7.260
7.252
6.713
6.681
6.365
6.353
6.342
6.333
6.321
5.511
4.851
4.839
-0.000
1.00
1.94
2.95
10.35
0.96
0.98
1.94
1.92
f1 (ppm)

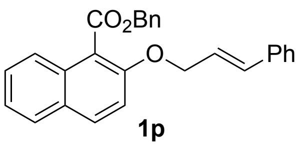
CO2Bn
O
Ph

1p

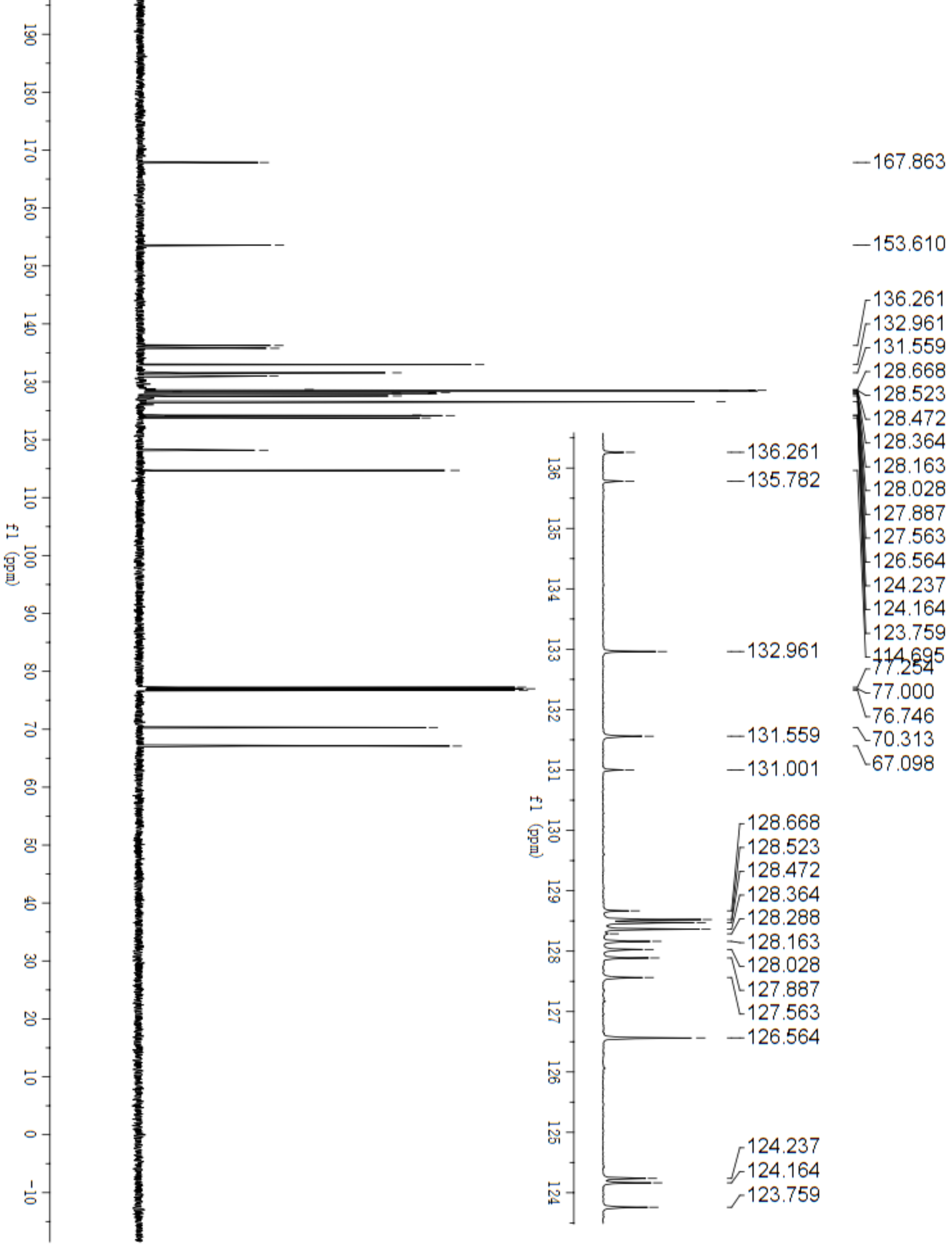
167.863
153.610
136.261
132.961
131.559
128.668
128.523
128.472
128.364
128.163
128.028
127.887
127.563
126.564
124.237
124.164
123.759
114.695
77.254
77.000
76.746
70.313
67.098
136.261
135.782
132.961
131.559
131.001
128.668
128.523
128.472
128.364
128.288
128.163
128.028
127.887
127.563
126.564
124.237
124.164
123.759
f1 (ppm)

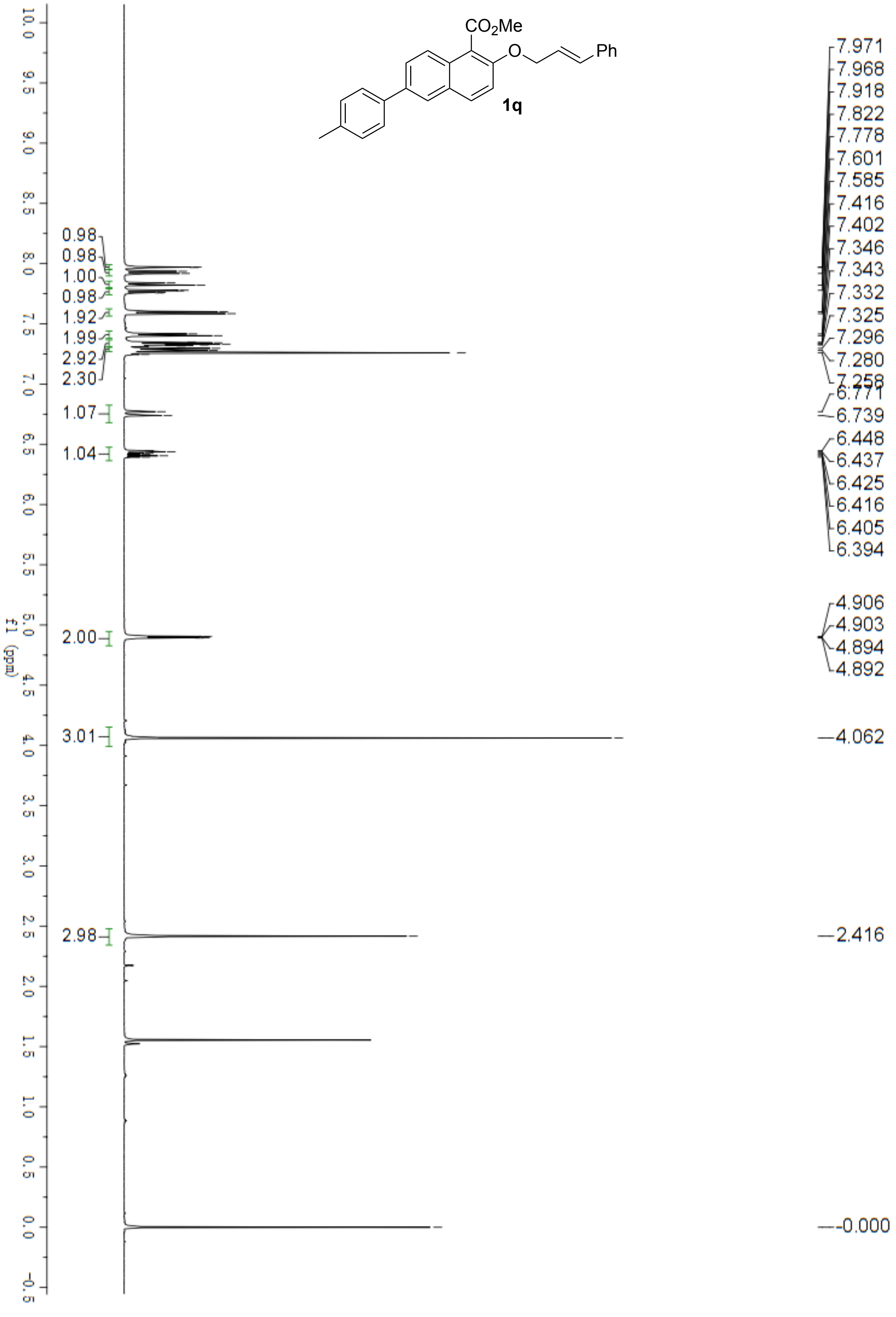
CO2Me
O
Ph
1q
7.971
7.968
7.918
7.822
7.778
7.601
7.585
7.416
7.402
7.346
7.343
7.332
7.325
7.296
7.280
7.258
6.771
6.739
6.448
6.437
6.425
6.416
6.405
6.394
4.906
4.903
4.894
4.892
4.062
2.416
-0.000
0.98
0.98
1.00
0.98
1.92
1.99
2.92
2.30
1.07
1.04
2.00
3.01
2.98
f1 (ppm)

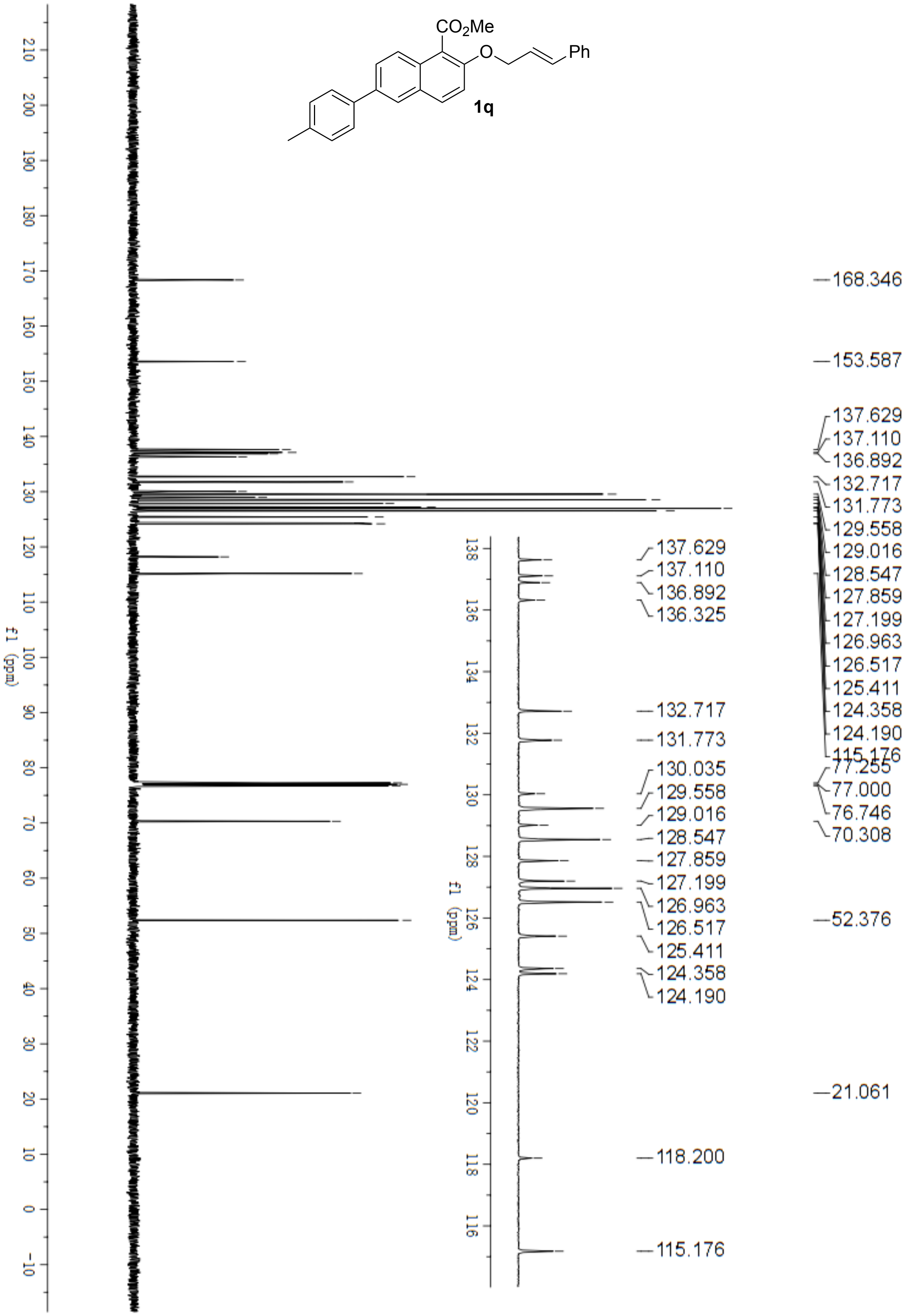
CO2Me
O
Ph
1q
168.346
153.587
137.629
137.110
136.892
132.717
131.773
129.558
129.016
128.547
127.859
127.199
126.963
126.517
125.411
124.358
124.190
115.176
77.255
77.000
76.746
70.308
52.376
21.061
130.035
136.325
118.200
f1 (ppm)

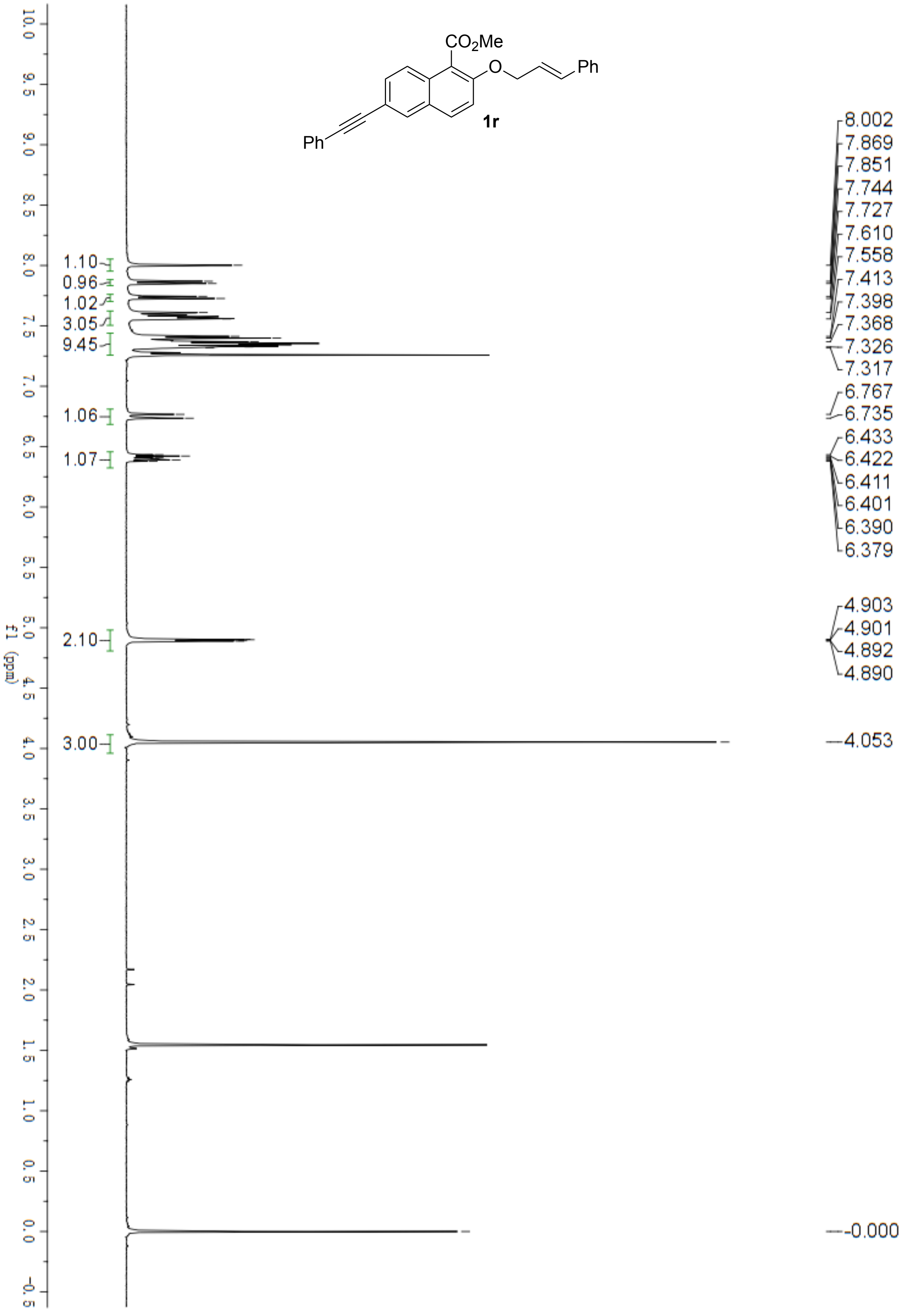
CO2Me
O
Ph
Ph
1r
8.002
7.869
7.851
7.744
7.727
7.610
7.558
7.413
7.398
7.368
7.326
7.317
6.767
6.735
6.433
6.422
6.411
6.401
6.390
6.379
4.903
4.901
4.892
4.890
4.053
-0.000
1.10
0.96
1.02
3.05
9.45
1.06
1.07
2.10
3.00
f1 (ppm)

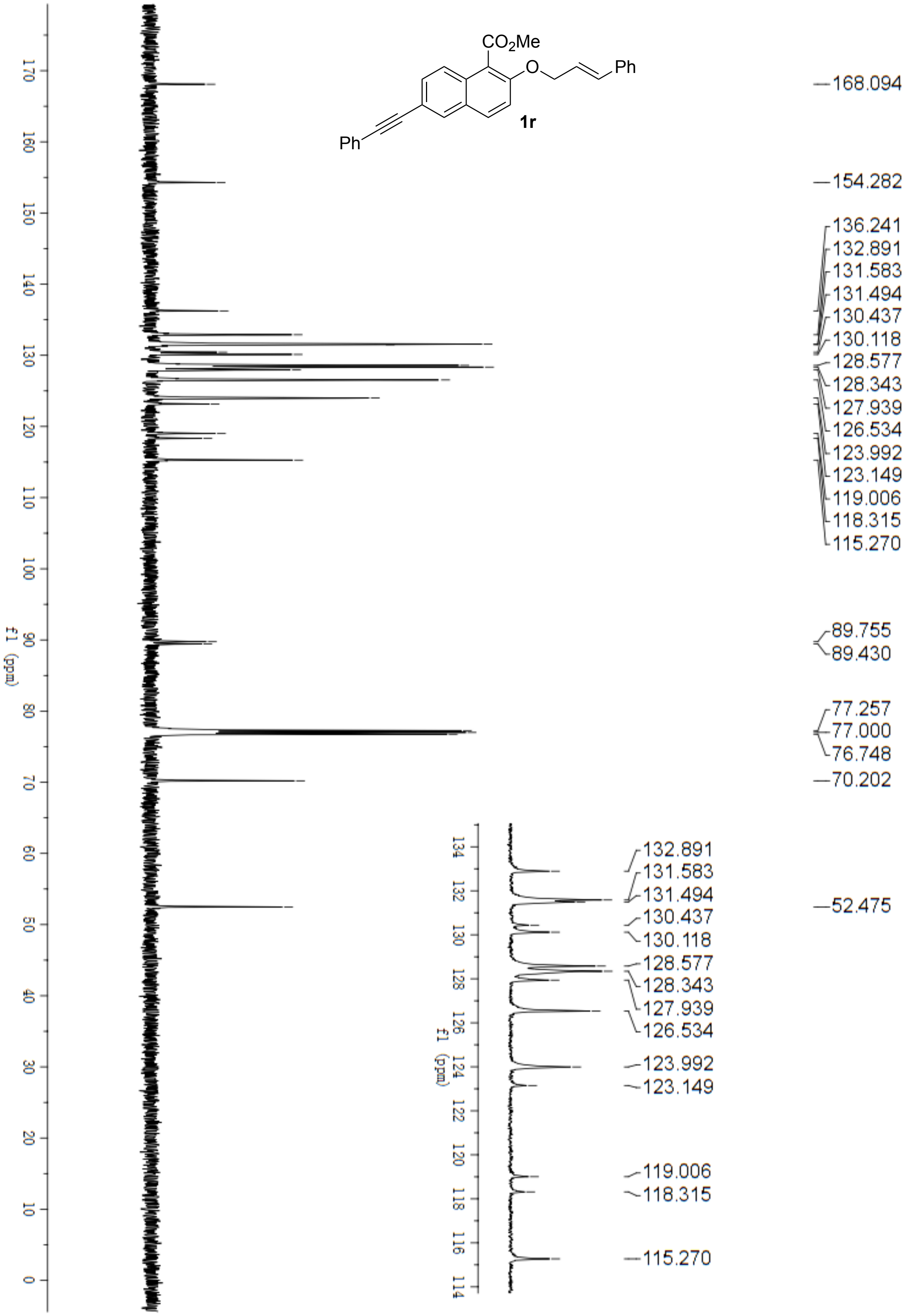
CO2Me
O
Ph
Ph
1r
168.094
154.282
136.241
132.891
131.583
131.494
130.437
130.118
128.577
128.343
127.939
126.534
123.992
123.149
119.006
118.315
115.270
89.755
89.430
77.257
77.000
76.748
70.202
52.475
f1 (ppm)

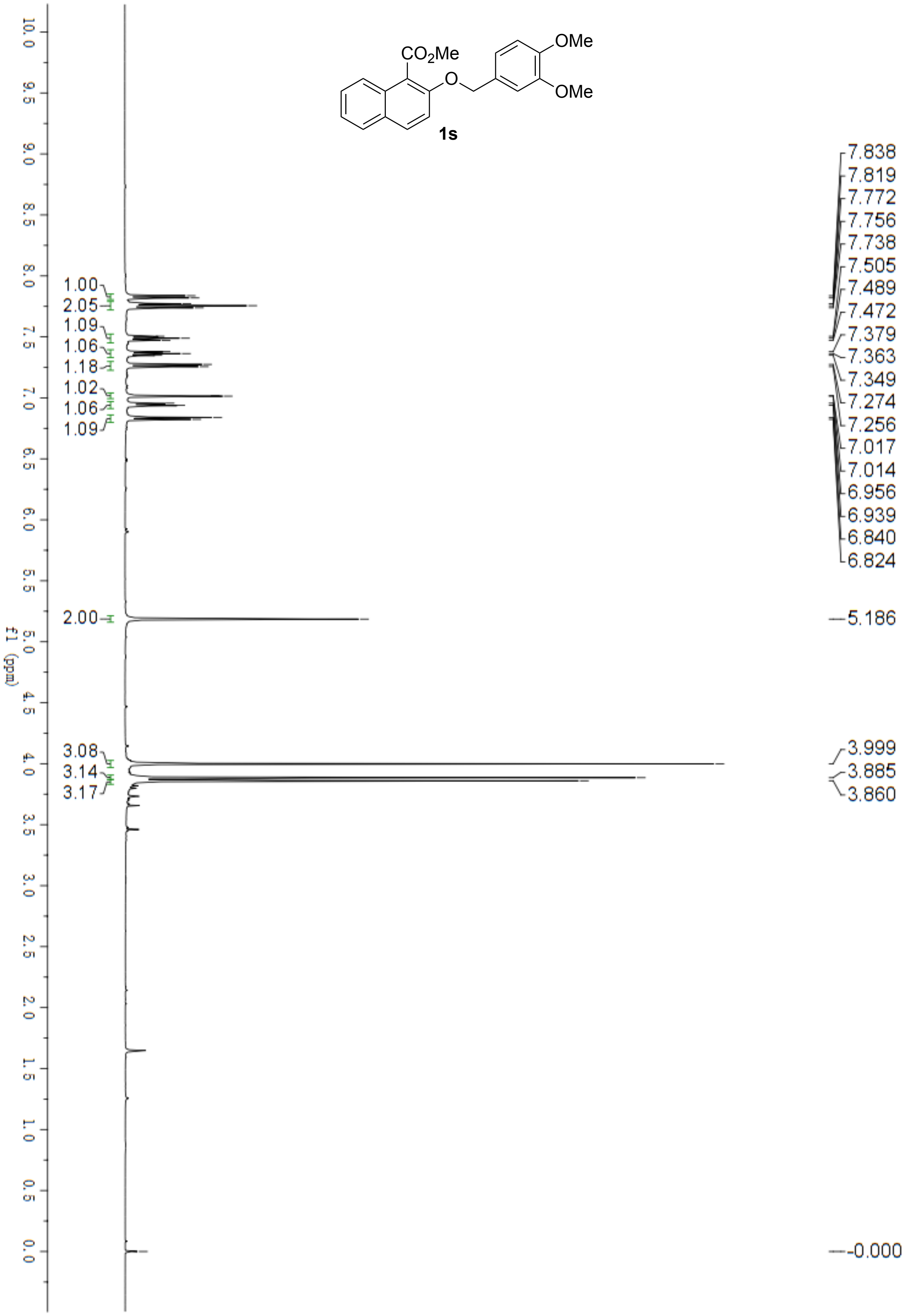
CO2Me
OMe
O
OMe
1s
7.838
7.819
7.772
7.756
7.738
7.505
7.489
7.472
7.379
7.363
7.349
7.274
7.256
7.017
7.014
6.956
6.939
6.840
6.824
5.186
3.999
3.885
3.860
-0.000
1.00
2.05
1.09
1.06
1.18
1.02
1.06
1.09
2.00
3.08
3.14
3.17
f1 (ppm)
10.0
9.5
9.0
8.5
8.0
7.5
7.0
6.5
6.0
5.5
5.0
4.5
4.0
3.5
3.0
2.5
2.0
1.5
1.0
0.5
0.0

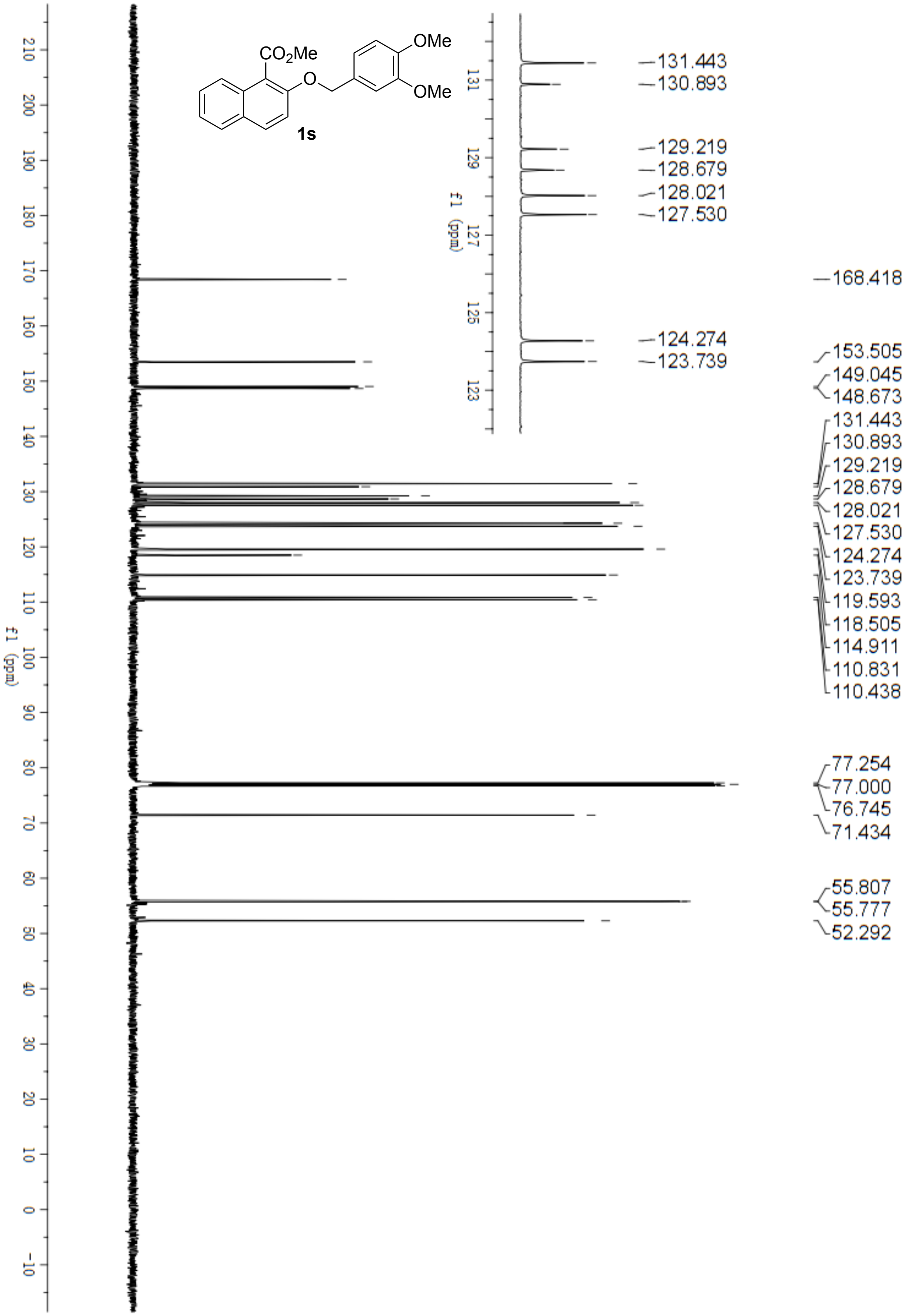
CO2Me
OMe
OMe
1s
131.443
130.893
129.219
128.679
128.021
127.530
124.274
123.739
168.418
153.505
149.045
148.673
131.443
130.893
129.219
128.679
128.021
127.530
124.274
123.739
119.593
118.505
114.911
110.831
110.438
77.254
77.000
76.745
71.434
55.807
55.777
52.292
f1 (ppm)

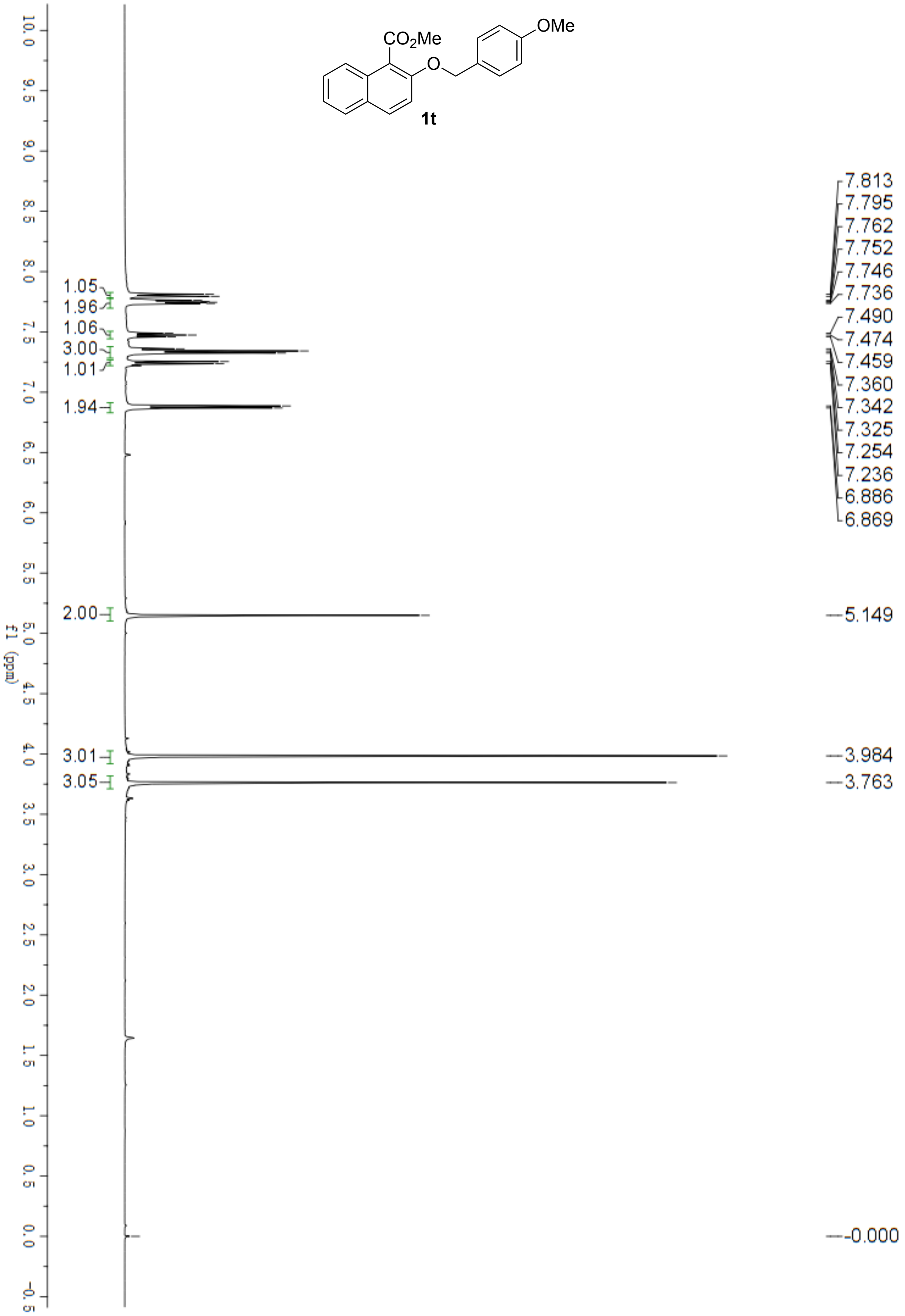
CO2Me
OMe
O
1t
7.813
7.795
7.762
7.752
7.746
7.736
7.490
7.474
7.459
7.360
7.342
7.325
7.254
7.236
6.886
6.869
5.149
3.984
3.763
-0.000
1.05
1.96
1.06
3.00
1.01
1.94
2.00
3.01
3.05
f1 (ppm)

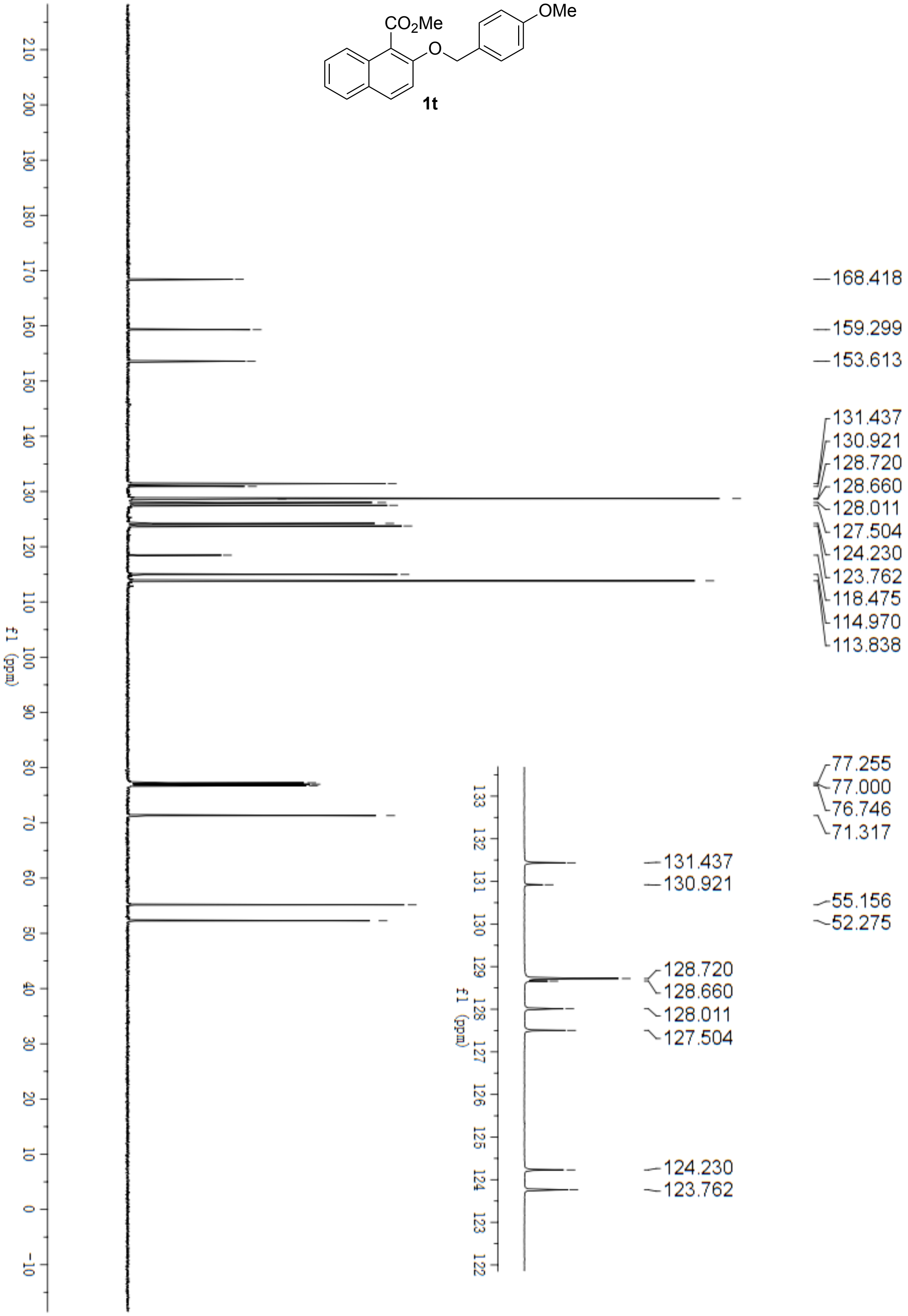

OMe
CO2Me
O
1t
168.418
159.299
153.613
131.437
130.921
128.720
128.660
128.011
127.504
124.230
123.762
118.475
114.970
113.838
77.255
77.000
76.746
71.317
55.156
52.275
f1 (ppm)
131.437
130.921
128.720
128.660
128.011
127.504
124.230
123.762
f1 (ppm)

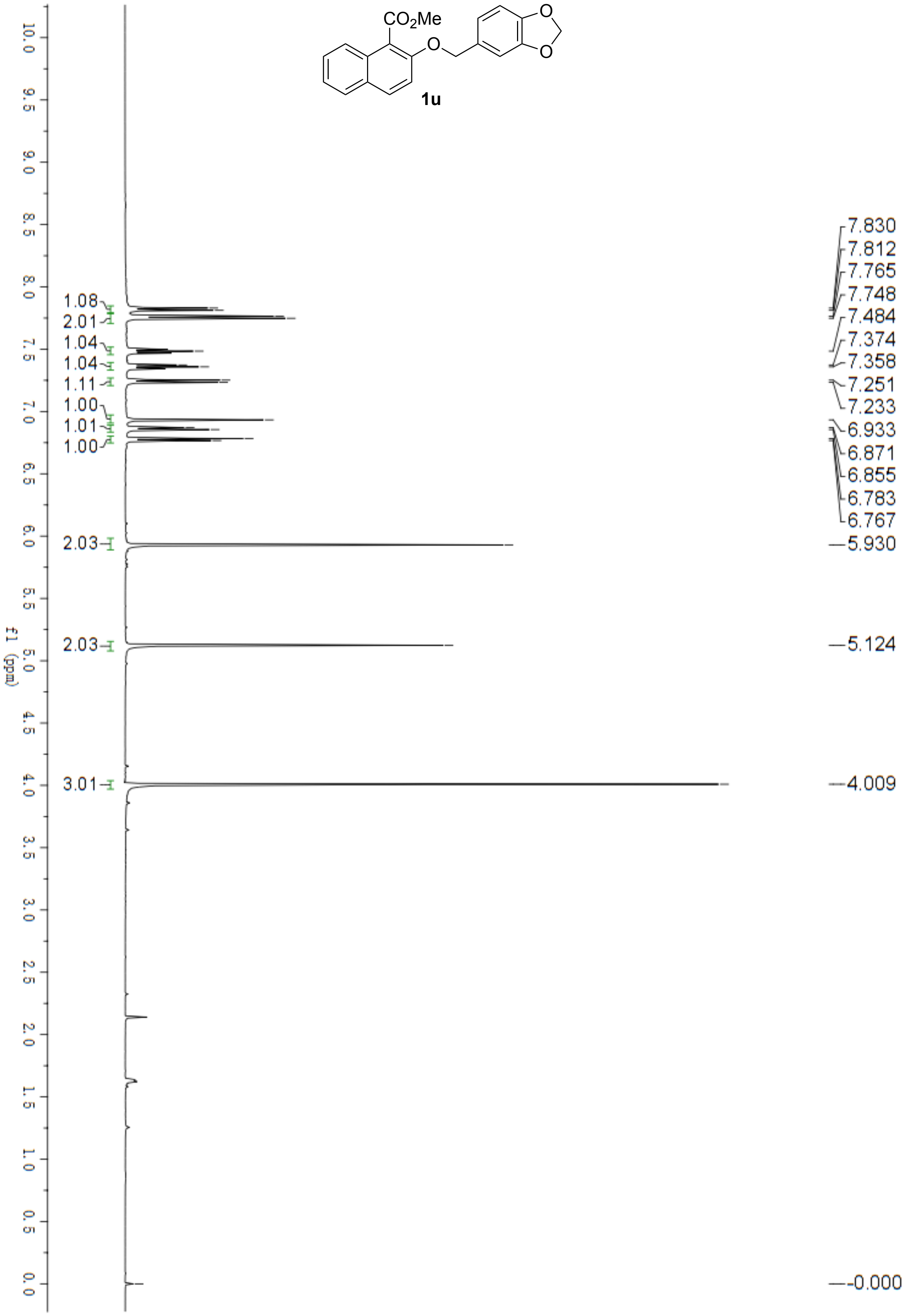
CO2Me
O
O
O
1u
7.830
7.812
7.765
7.748
7.484
7.374
7.358
7.251
7.233
6.933
6.871
6.855
6.783
6.767
5.930
5.124
4.009
-0.000
1.08
2.01
1.04
1.04
1.11
1.00
1.01
1.00
2.03
2.03
3.01
f1 (ppm)

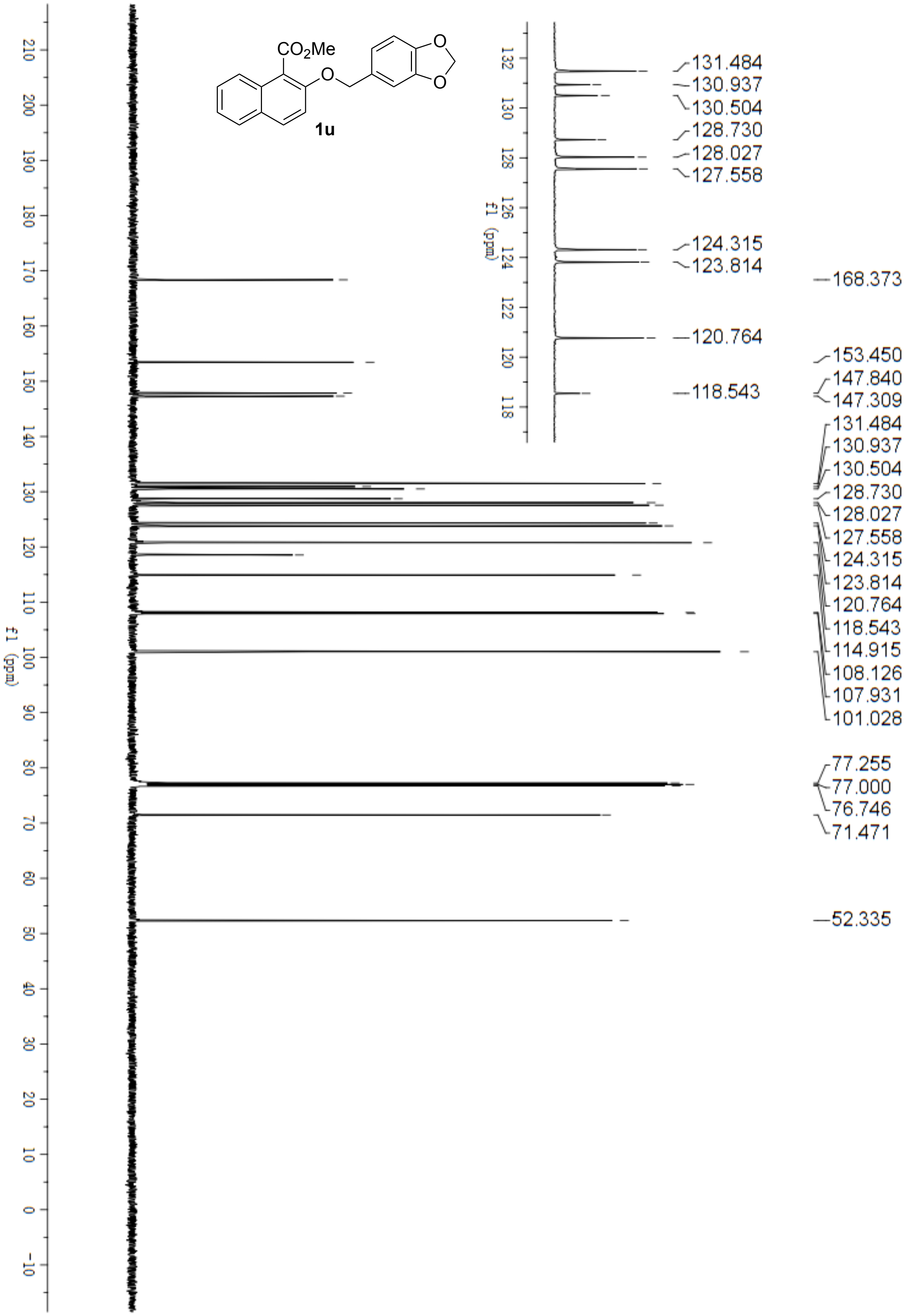
CO2Me
O
O
O
1u
131.484
130.937
130.504
128.730
128.027
127.558
124.315
123.814
120.764
118.543
168.373
153.450
147.840
147.309
131.484
130.937
130.504
128.730
128.027
127.558
124.315
123.814
120.764
118.543
114.915
108.126
107.931
101.028
77.255
77.000
76.746
71.471
52.335
f1 (ppm)

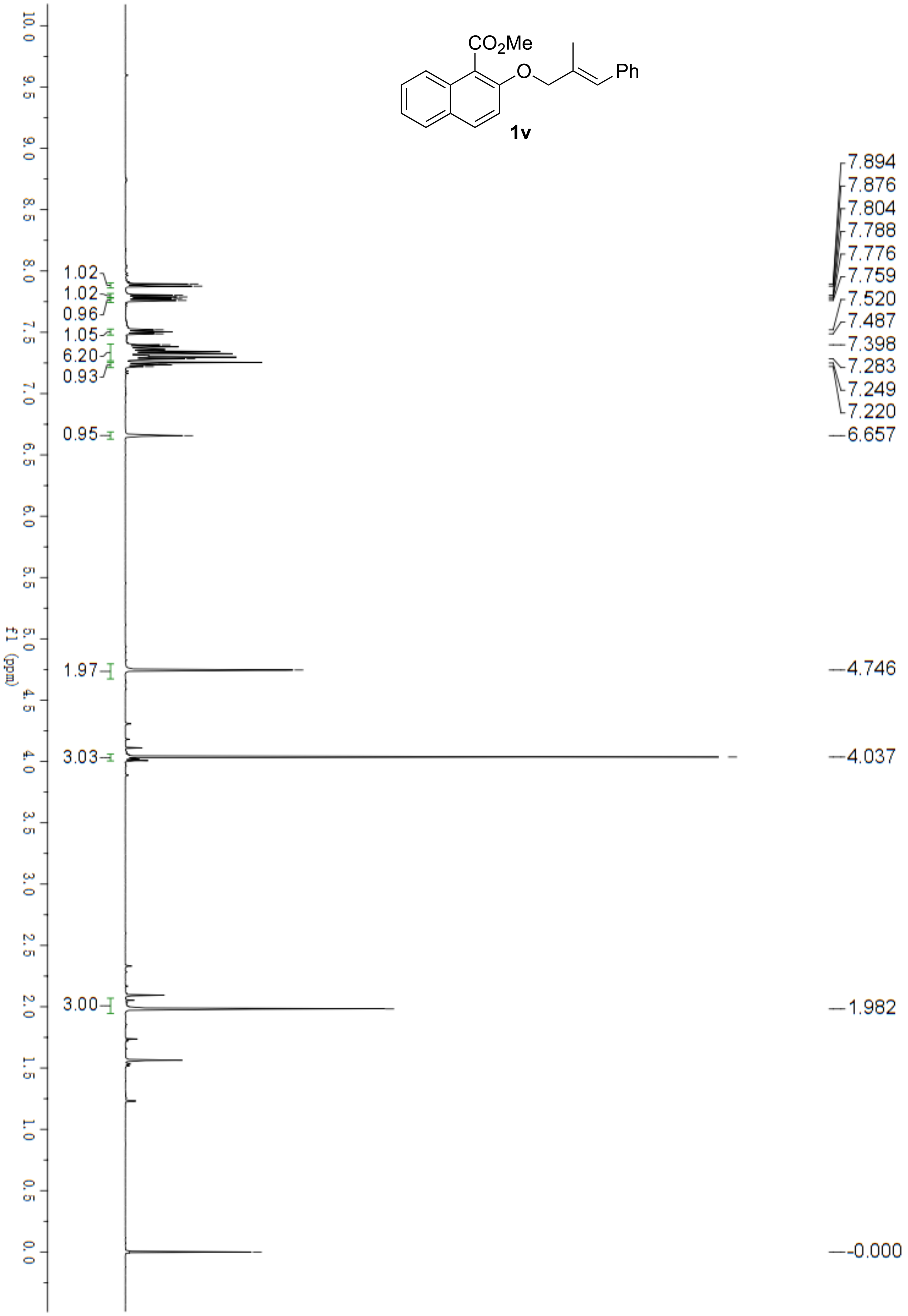
CO2Me
O
Ph
1v
7.894
7.876
7.804
7.788
7.776
7.759
7.520
7.487
7.398
7.283
7.249
7.220
6.657
4.746
4.037
1.982
-0.000
1.02
1.02
0.96
1.05
6.20
0.93
0.95
1.97
3.03
3.00
f1 (ppm)

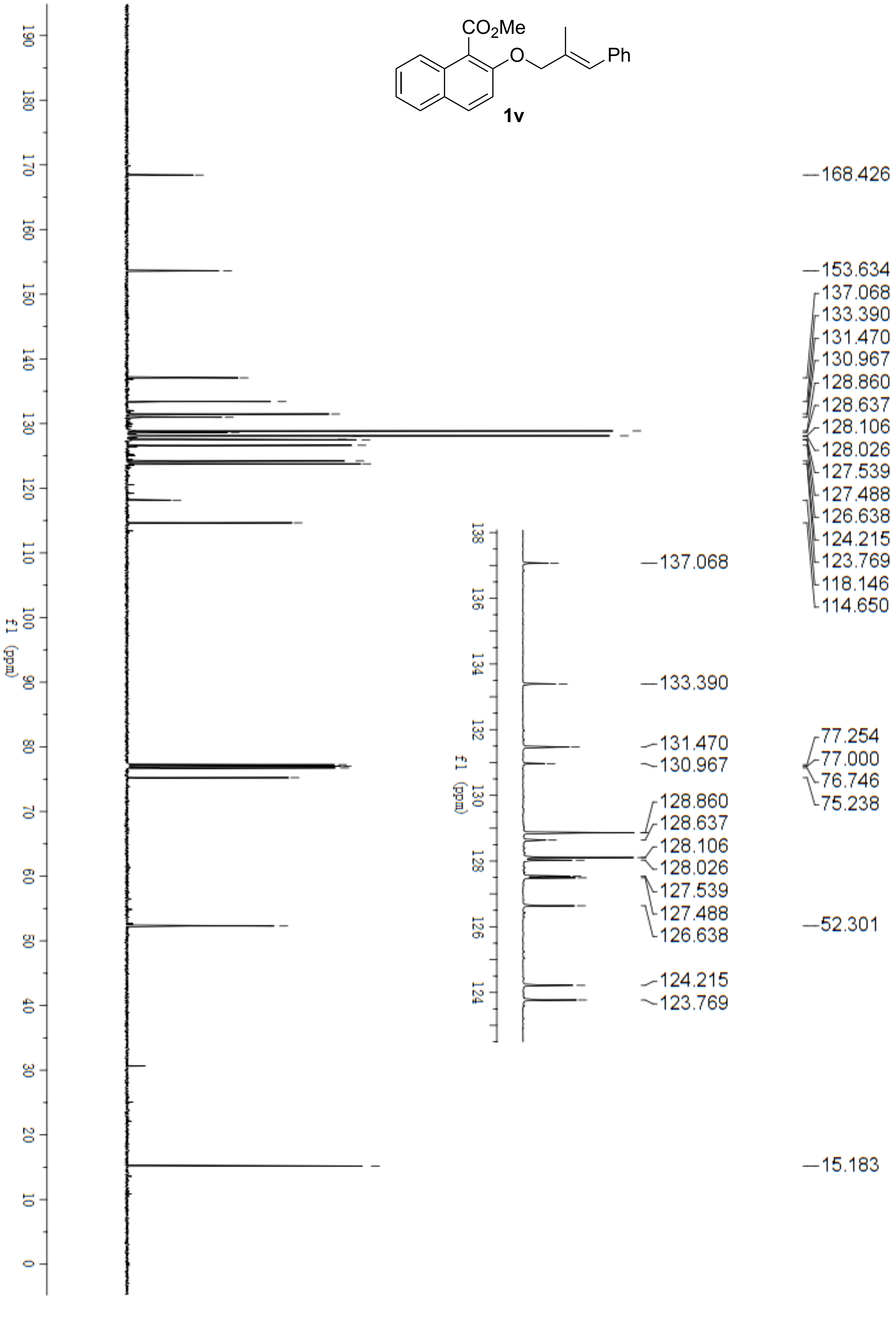
CO2Me
O
Ph
1v
168.426
153.634
137.068
133.390
131.470
130.967
128.860
128.637
128.106
128.026
127.539
127.488
126.638
124.215
123.769
118.146
114.650
77.254
77.000
76.746
75.238
52.301
15.183
f1 (ppm)

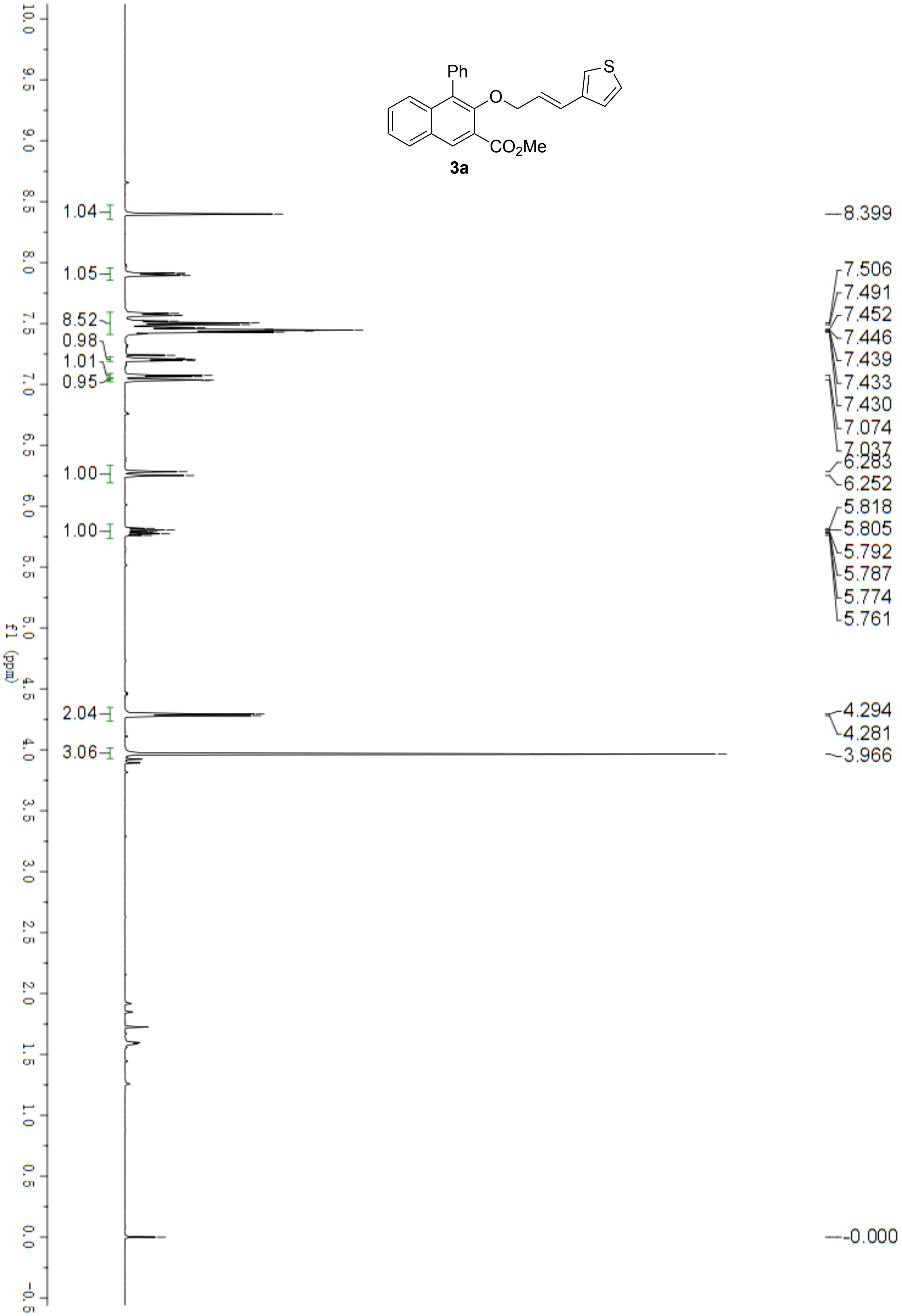
Ph
O
S
CO2Me
3a
8.399
7.506
7.491
7.452
7.446
7.439
7.433
7.430
7.074
7.037
6.283
6.252
5.818
5.805
5.792
5.787
5.774
5.761
4.294
4.281
3.966
-0.000
1.04
1.05
8.52
0.98
1.01
0.95
1.00
1.00
2.04
3.06
f1 (ppm)

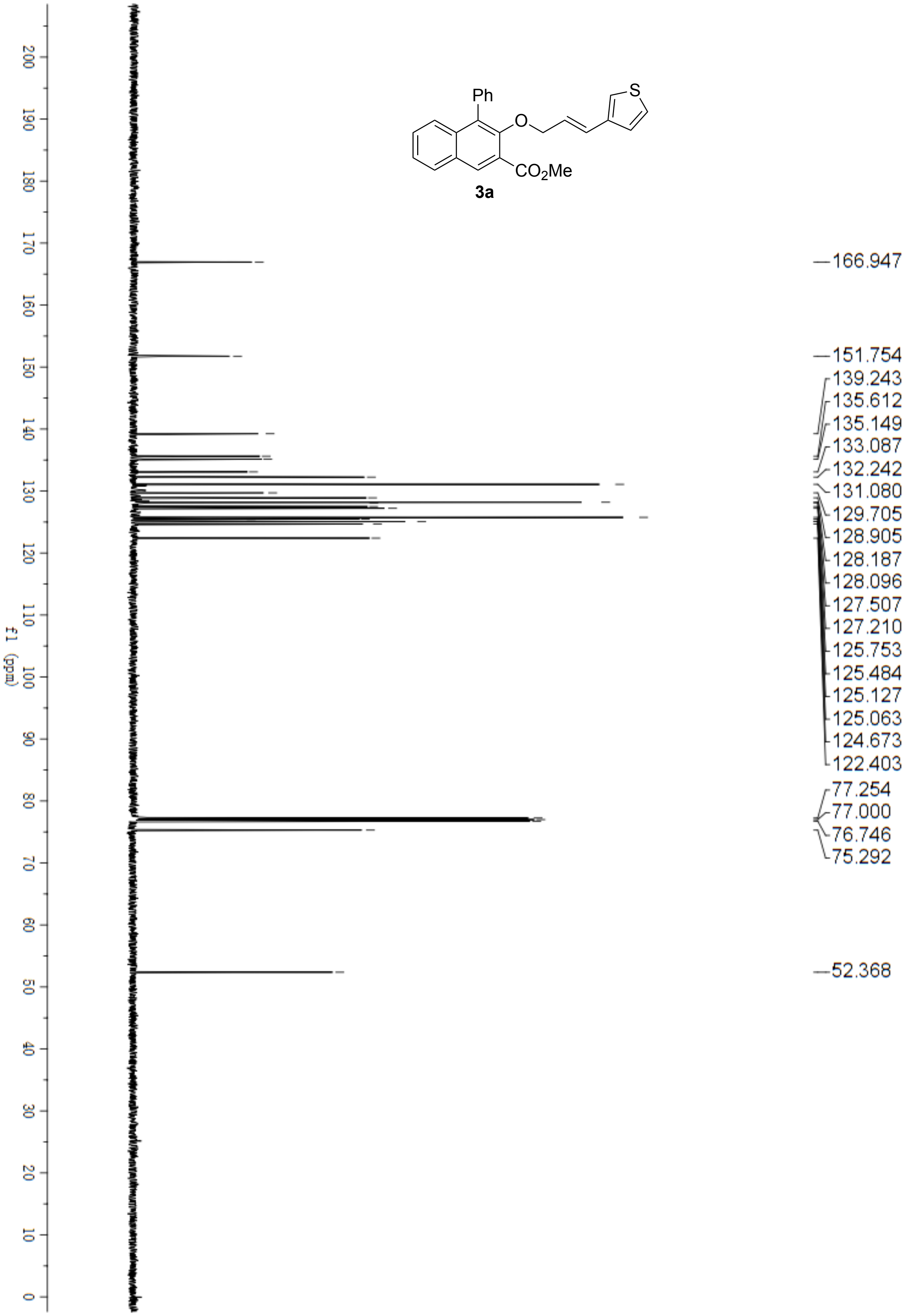
Ph
O
S
CO2Me
3a
166.947
151.754
139.243
135.612
135.149
133.087
132.242
131.080
129.705
128.905
128.187
128.096
127.507
127.210
125.753
125.484
125.127
125.063
124.673
122.403
77.254
77.000
76.746
75.292
52.368
f1 (ppm)

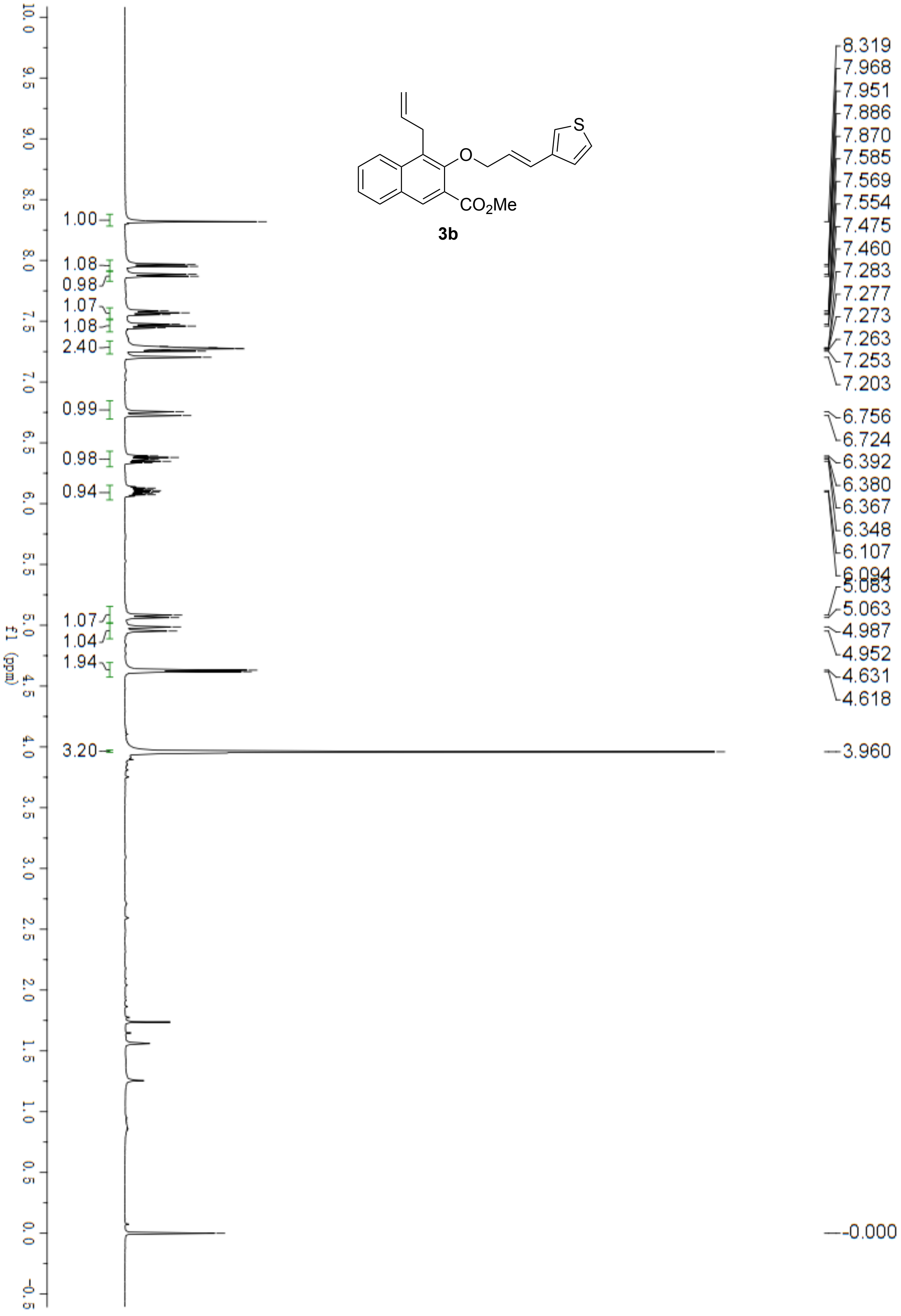
CO2Me
3b
S
O
8.319
7.968
7.951
7.886
7.870
7.585
7.569
7.554
7.475
7.460
7.283
7.277
7.273
7.263
7.253
7.203
6.756
6.724
6.392
6.380
6.367
6.348
6.107
6.094
5.083
5.063
4.987
4.952
4.631
4.618
3.960
-0.000
1.00
1.08
0.98
1.07
1.08
2.40
0.99
0.98
0.94
1.07
1.04
1.94
3.20
f1 (ppm)

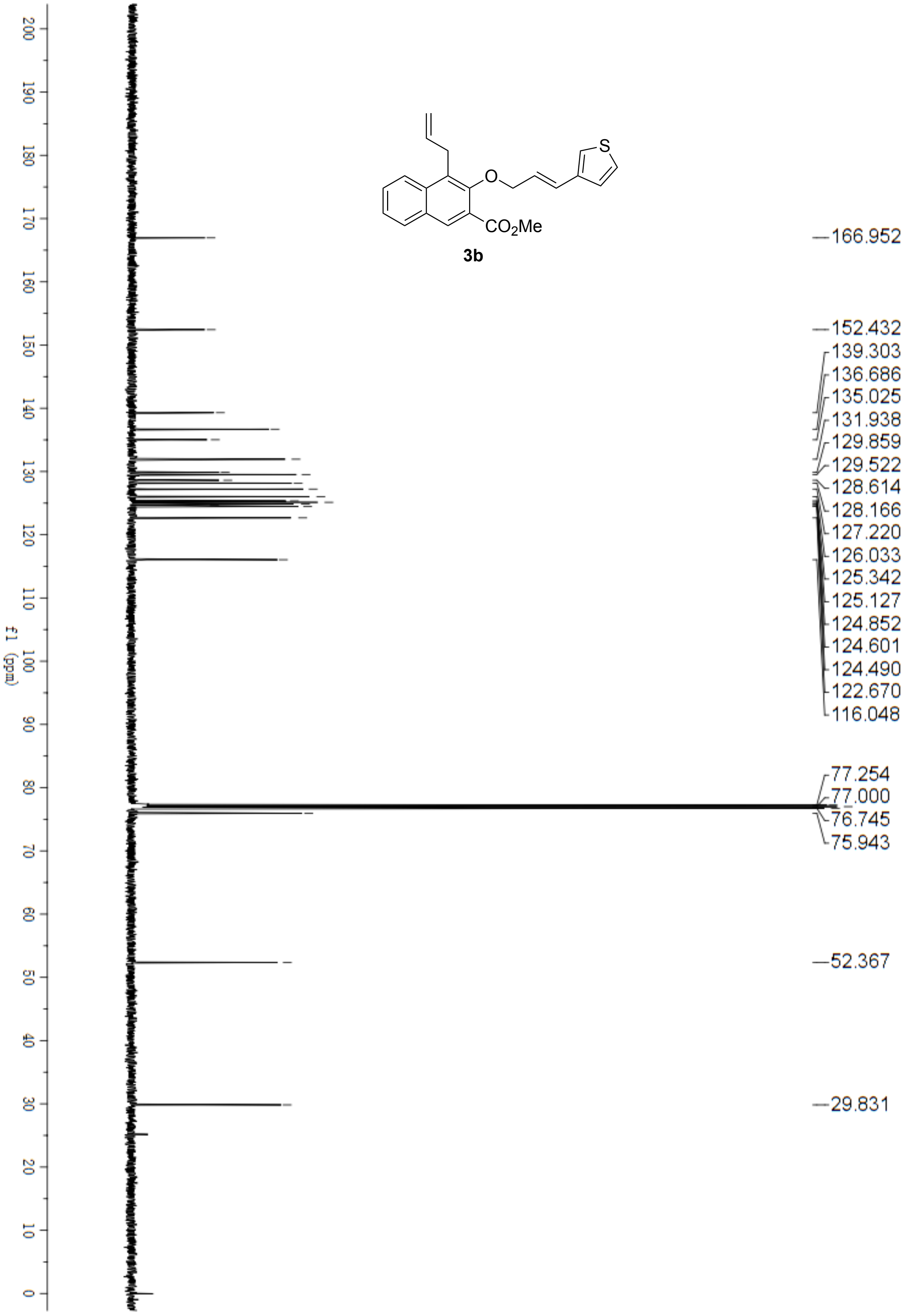
S
O
CO2Me
3b
166.952
152.432
139.303
136.686
135.025
131.938
129.859
129.522
128.614
128.166
127.220
126.033
125.342
125.127
124.852
124.601
124.490
122.670
116.048
77.254
77.000
76.745
75.943
52.367
29.831
f1 (ppm)

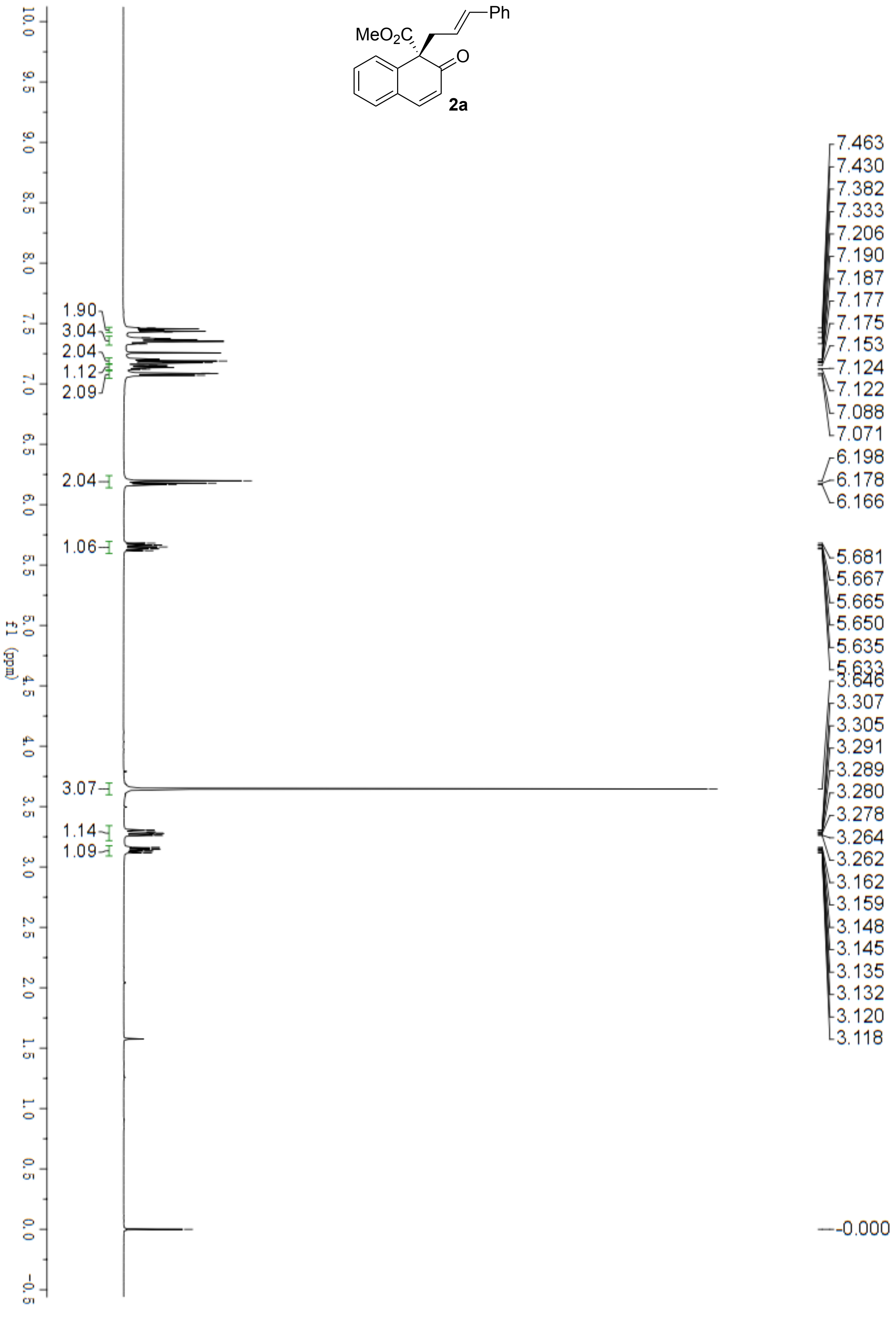
MeO2C
Ph
O
2a
7.463
7.430
7.382
7.333
7.206
7.190
7.187
7.177
7.175
7.153
7.124
7.122
7.088
7.071
6.198
6.178
6.166
5.681
5.667
5.665
5.650
5.635
5.633
3.646
3.307
3.305
3.291
3.289
3.280
3.278
3.264
3.262
3.162
3.159
3.148
3.145
3.135
3.132
3.120
3.118
-0.000
1.90
3.04
2.04
1.12
2.09
2.04
1.06
3.07
1.14
1.09
f1 (ppm)

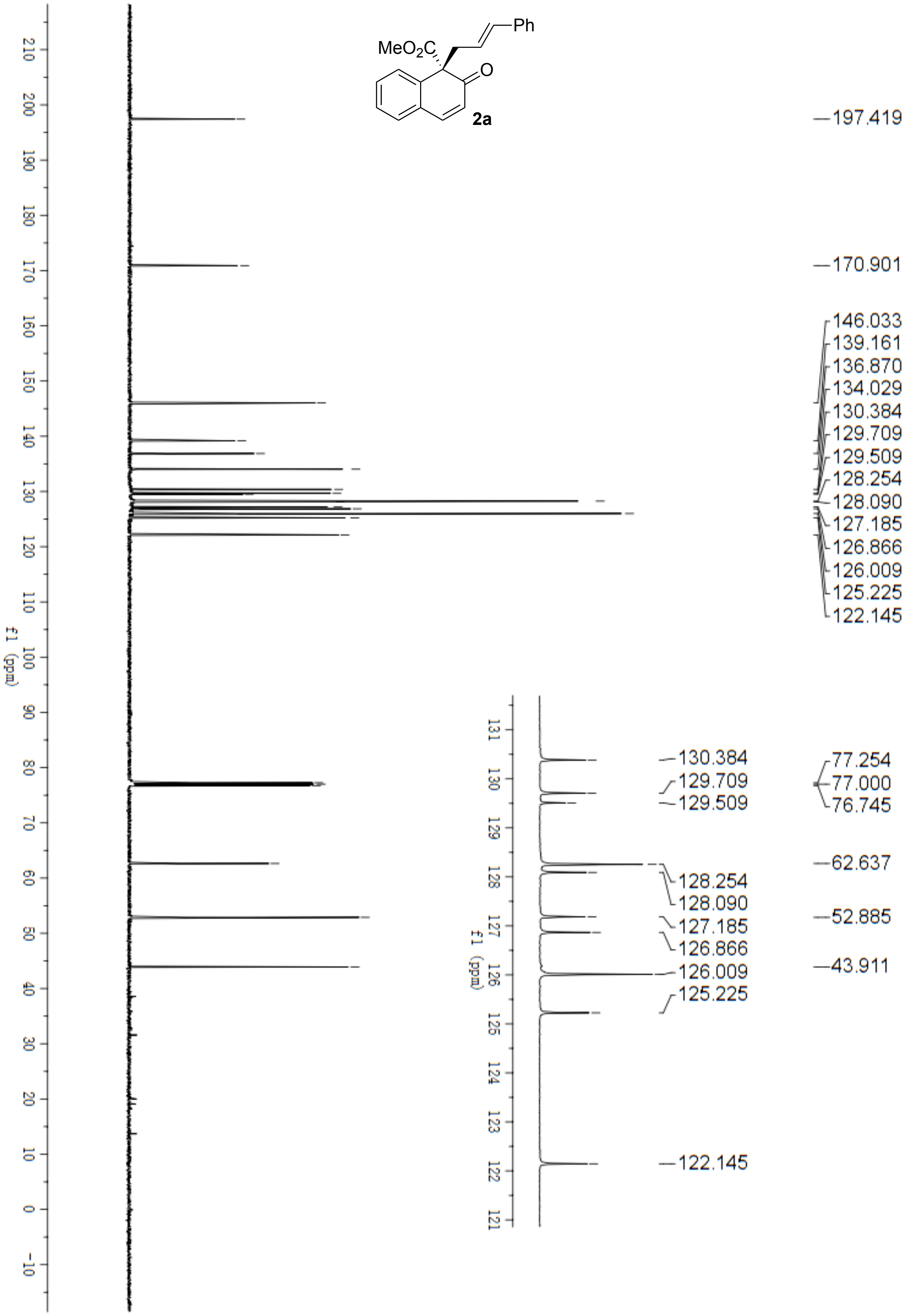
MeO2C
Ph
O
2a
197.419
170.901
146.033
139.161
136.870
134.029
130.384
129.709
129.509
128.254
128.090
127.185
126.866
126.009
125.225
122.145
77.254
77.000
76.745
62.637
52.885
43.911
f1 (ppm)

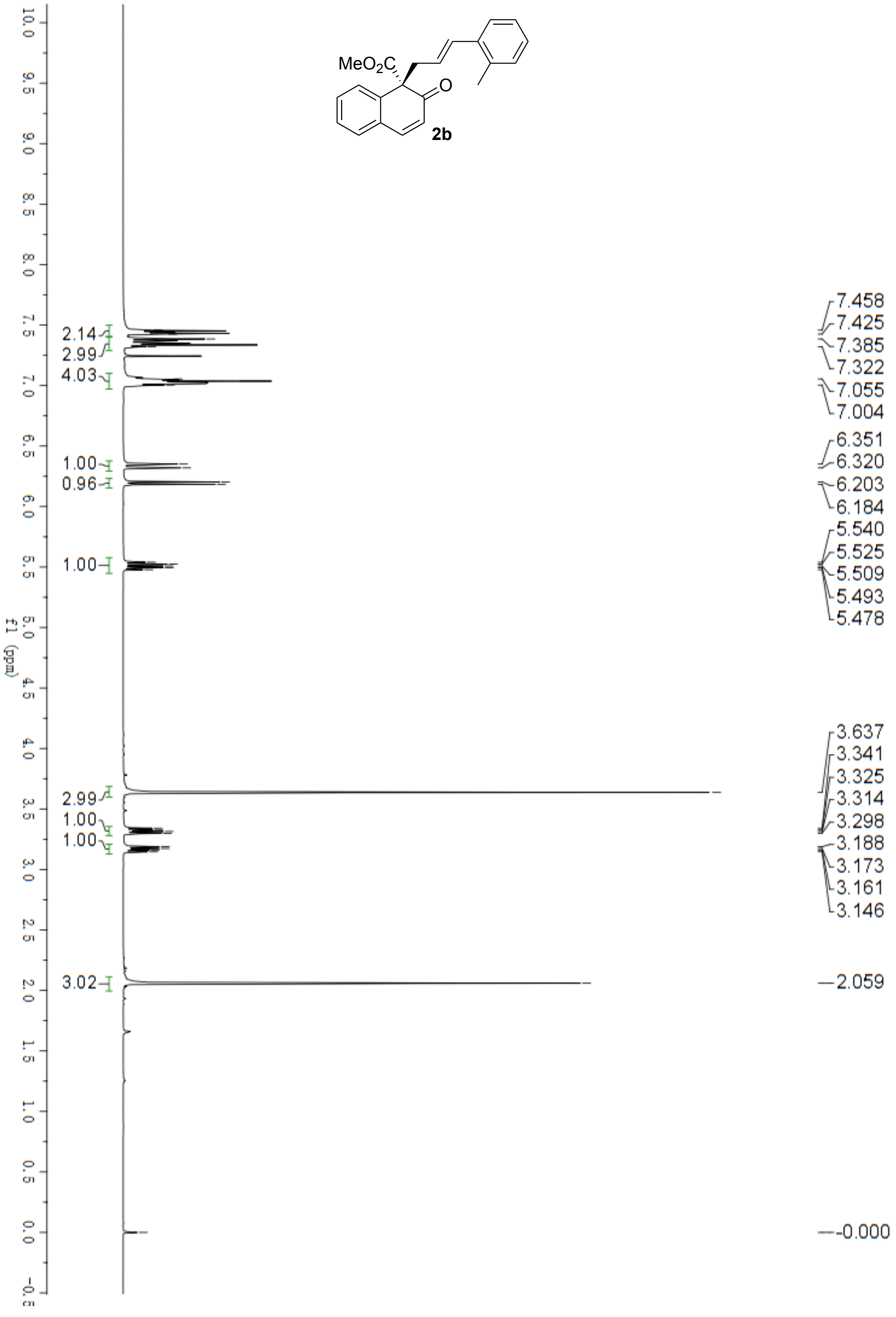
MeO2C
O
2b
7.458
7.425
7.385
7.322
7.055
7.004
6.351
6.320
6.203
6.184
5.540
5.525
5.509
5.493
5.478
3.637
3.341
3.325
3.314
3.298
3.188
3.173
3.161
3.146
2.059
-0.000
2.14
2.99
4.03
1.00
0.96
1.00
2.99
1.00
1.00
3.02
f1 (ppm)

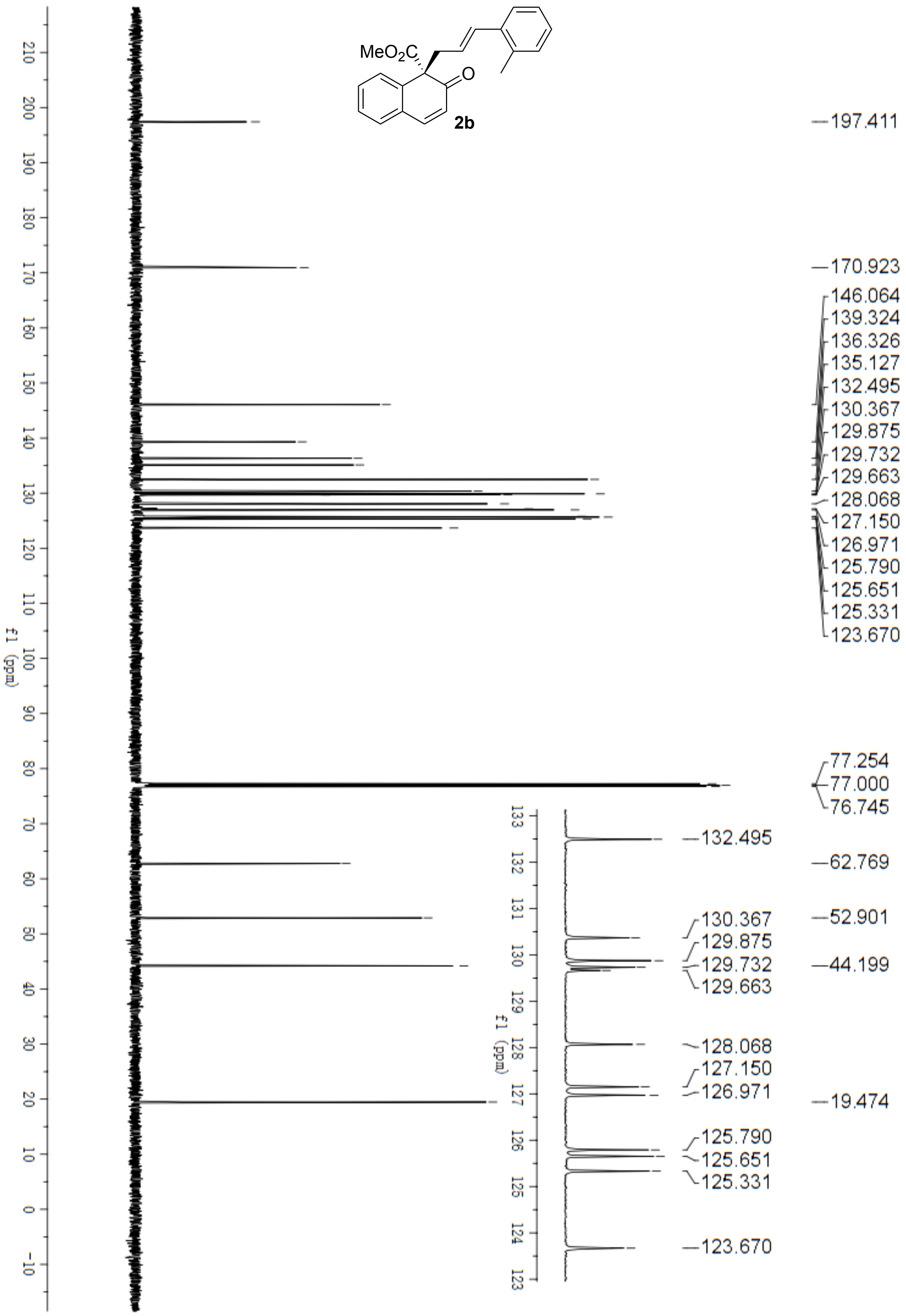
MeO2C
O
2b
197.411
170.923
146.064
139.324
136.326
135.127
132.495
130.367
129.875
129.732
129.663
128.068
127.150
126.971
125.790
125.651
125.331
123.670
77.254
77.000
76.745
62.769
52.901
44.199
19.474
f1 (ppm)

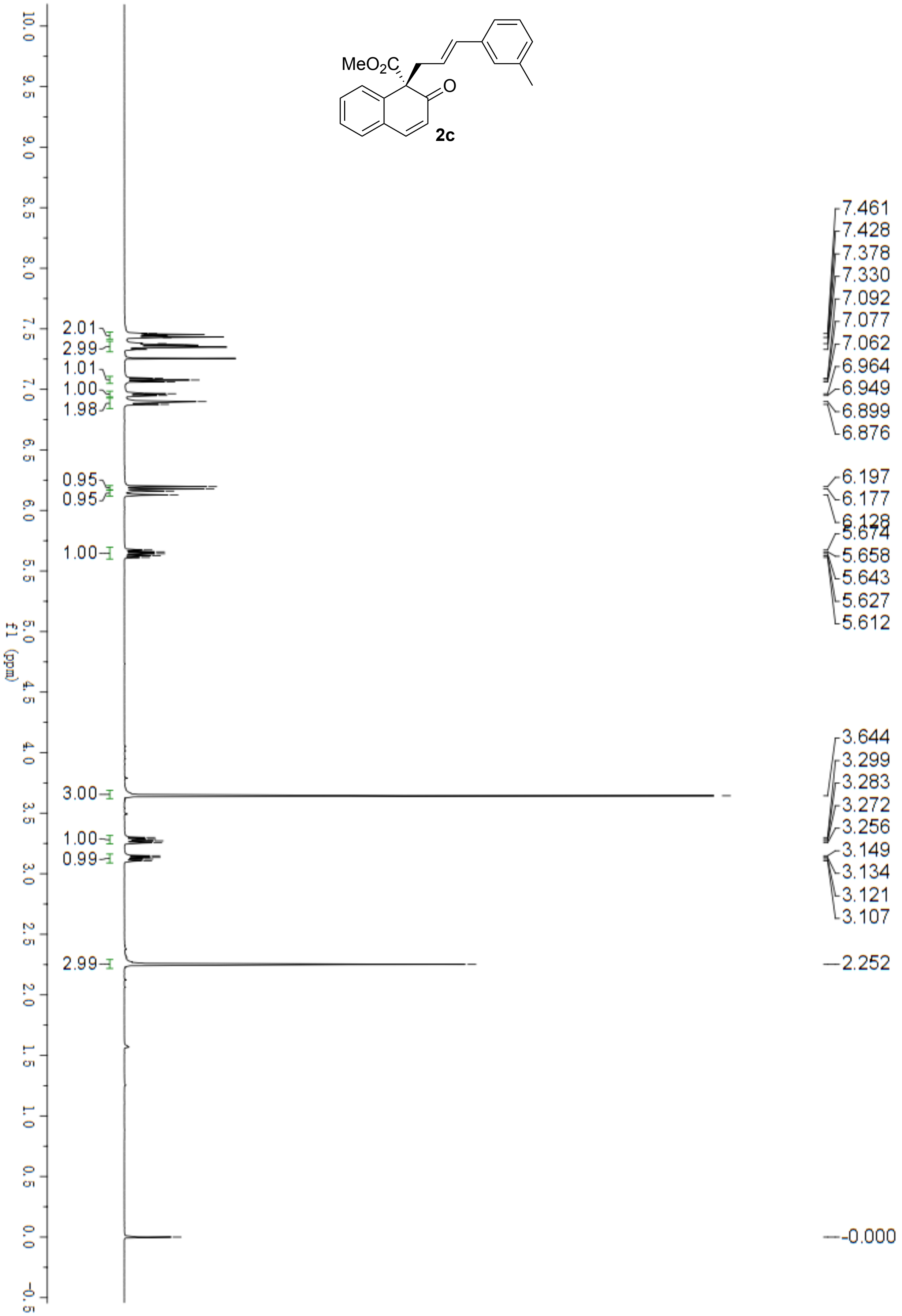
MeO2C
O
2c
7.461
7.428
7.378
7.330
7.092
7.077
7.062
6.964
6.949
6.899
6.876
6.197
6.177
6.128
5.674
5.658
5.643
5.627
5.612
3.644
3.299
3.283
3.272
3.256
3.149
3.134
3.121
3.107
2.252
-0.000
2.01
2.99
1.01
1.00
1.98
0.95
0.95
1.00
3.00
1.00
0.99
2.99
f1 (ppm)

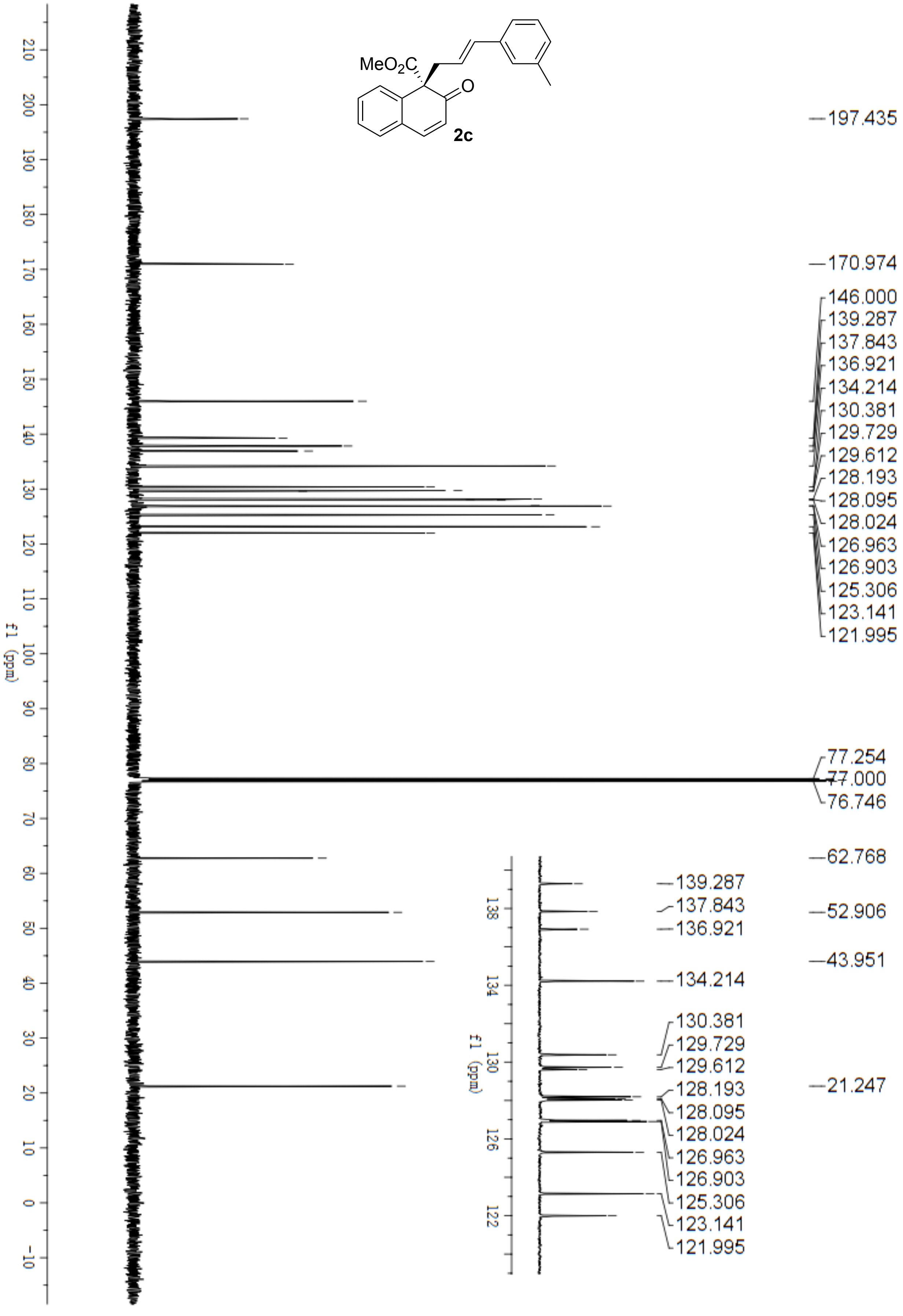
MeO2C
O
2c
197.435
170.974
146.000
139.287
137.843
136.921
134.214
130.381
129.729
129.612
128.193
128.095
128.024
126.963
126.903
125.306
123.141
121.995
77.254
77.000
76.746
62.768
52.906
43.951
21.247
f1 (ppm)

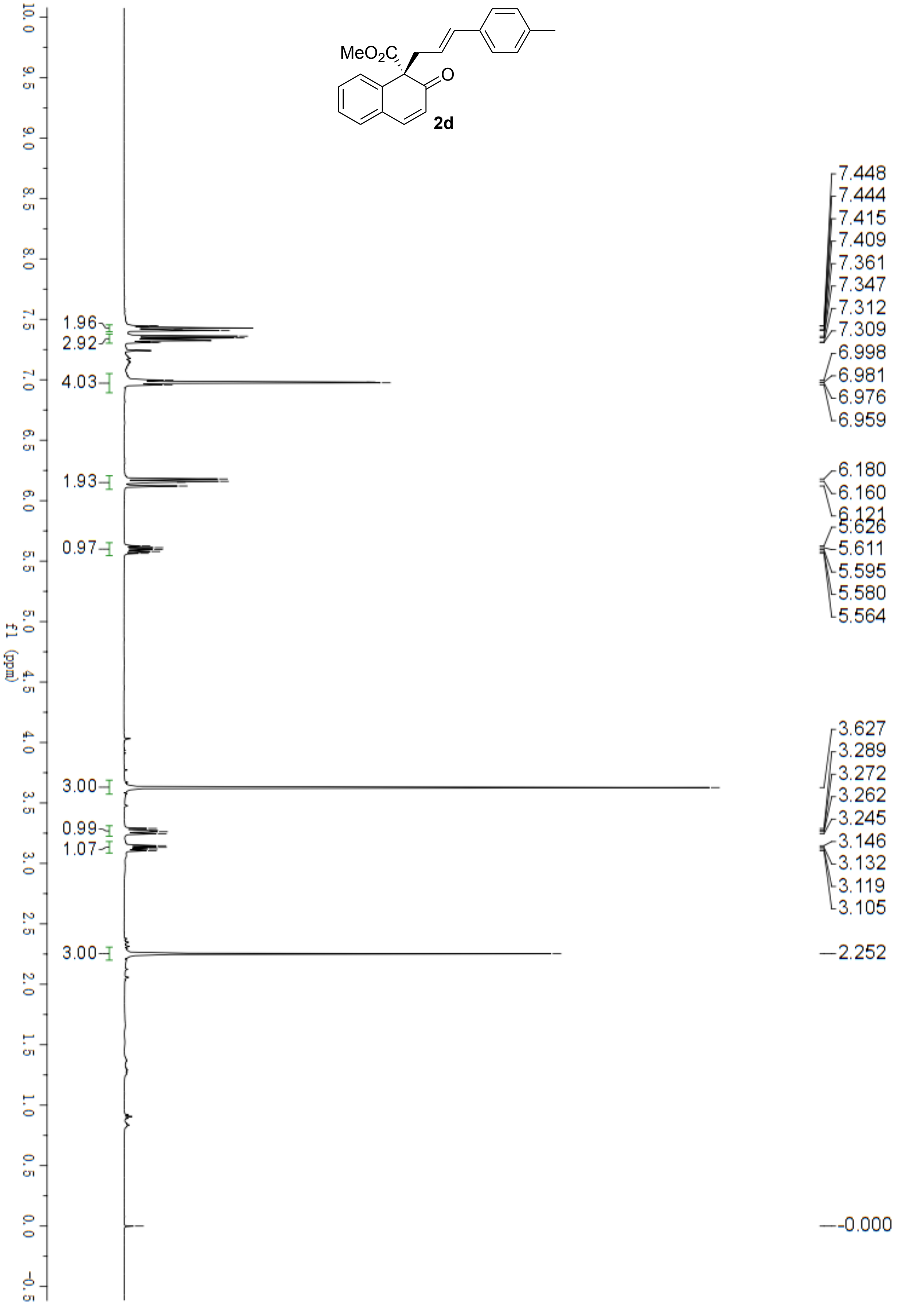
MeO2C
O
2d
7.448
7.444
7.415
7.409
7.361
7.347
7.312
7.309
6.998
6.981
6.976
6.959
6.180
6.160
6.121
5.626
5.611
5.595
5.580
5.564
3.627
3.289
3.272
3.262
3.245
3.146
3.132
3.119
3.105
2.252
-0.000
1.96
2.92
4.03
1.93
0.97
3.00
0.99
1.07
3.00
f1 (ppm)

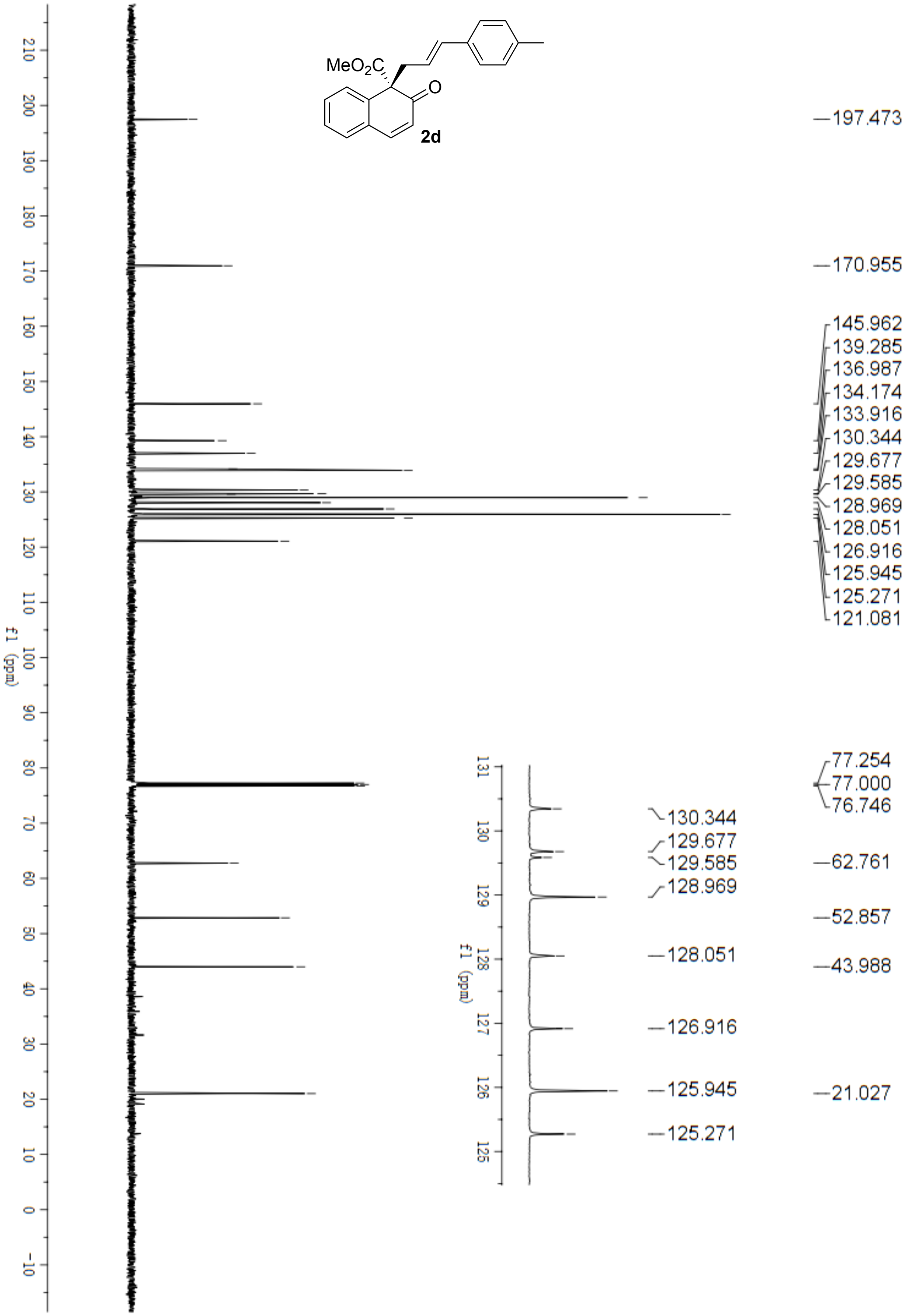
MeO2C
O
2d
197.473
170.955
145.962
139.285
136.987
134.174
133.916
130.344
129.677
129.585
128.969
128.051
126.916
125.945
125.271
121.081
77.254
77.000
76.746
62.761
52.857
43.988
21.027
130.344
129.677
129.585
128.969
128.051
126.916
125.945
125.271
f1 (ppm)
f1 (ppm)

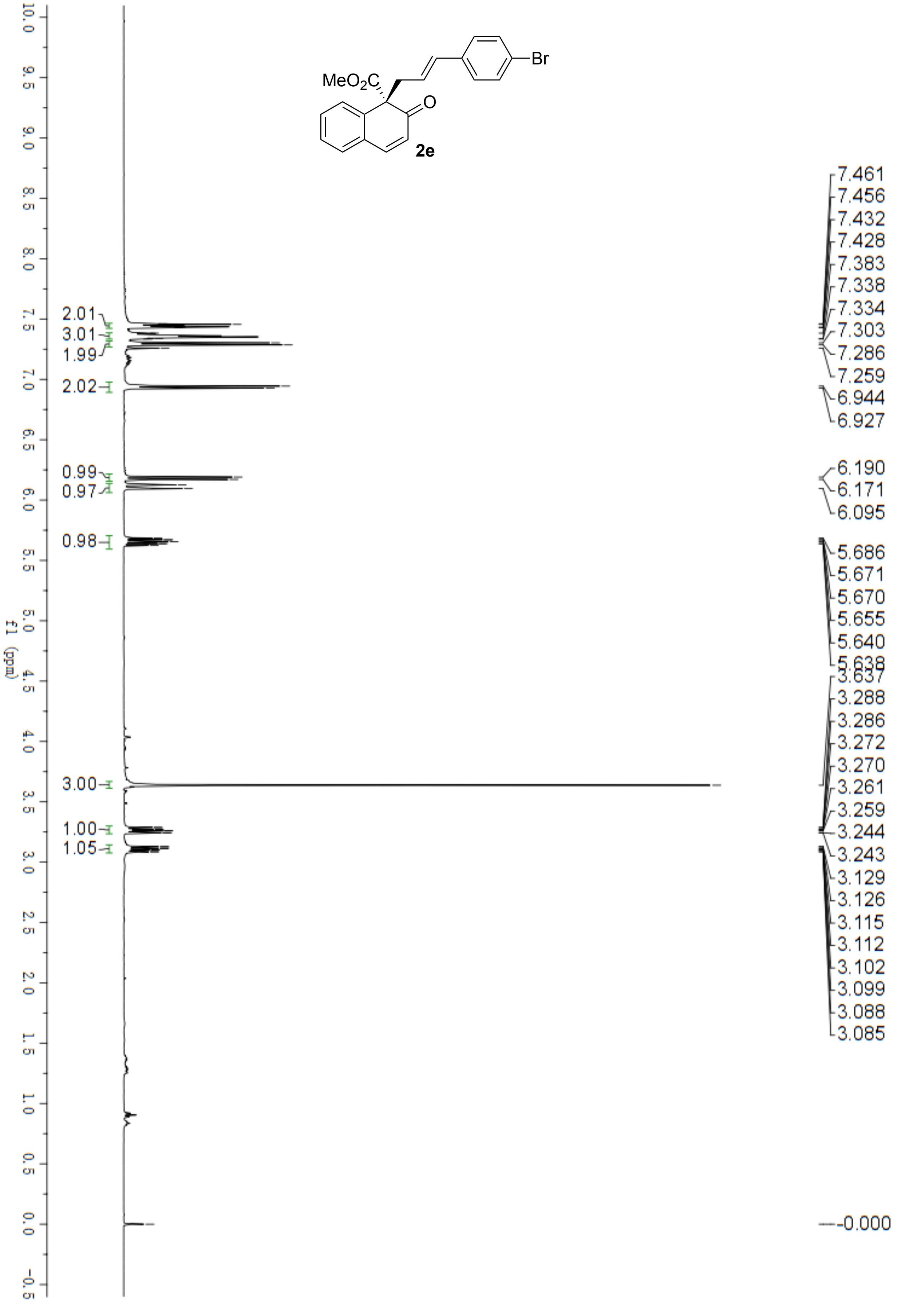
MeO2C
Br
O
2e
7.461
7.456
7.432
7.428
7.383
7.338
7.334
7.303
7.286
7.259
6.944
6.927
6.190
6.171
6.095
5.686
5.671
5.670
5.655
5.640
5.638
3.637
3.288
3.286
3.272
3.270
3.261
3.259
3.244
3.243
3.129
3.126
3.115
3.112
3.102
3.099
3.088
3.085
-0.000
2.01
3.01
1.99
2.02
0.99
0.97
0.98
3.00
1.00
1.05
f1 (ppm)

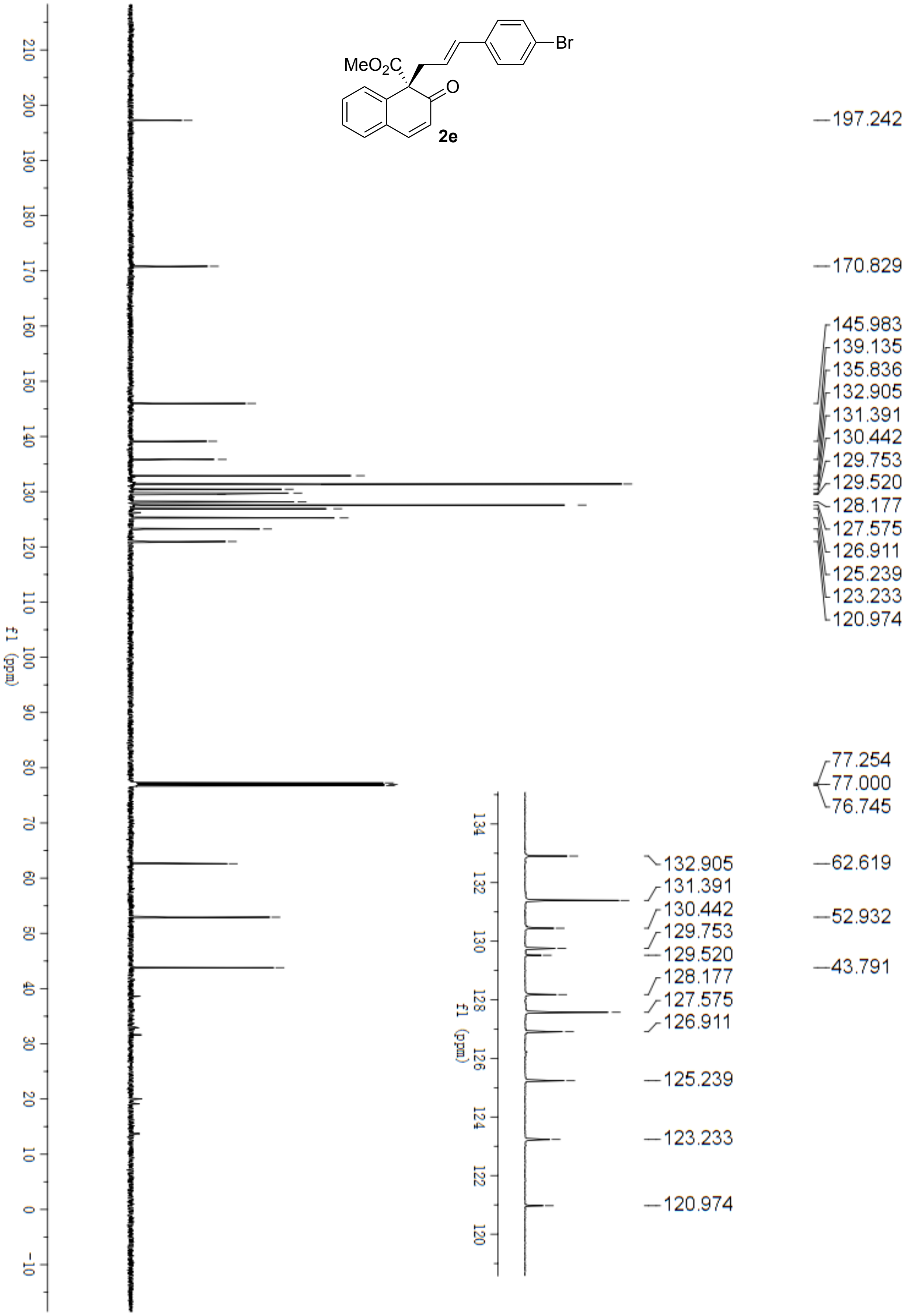
MeO2C
Br
O
2e
197.242
170.829
145.983
139.135
135.836
132.905
131.391
130.442
129.753
129.520
128.177
127.575
126.911
125.239
123.233
120.974
77.254
77.000
76.745
62.619
52.932
43.791
f1 (ppm)

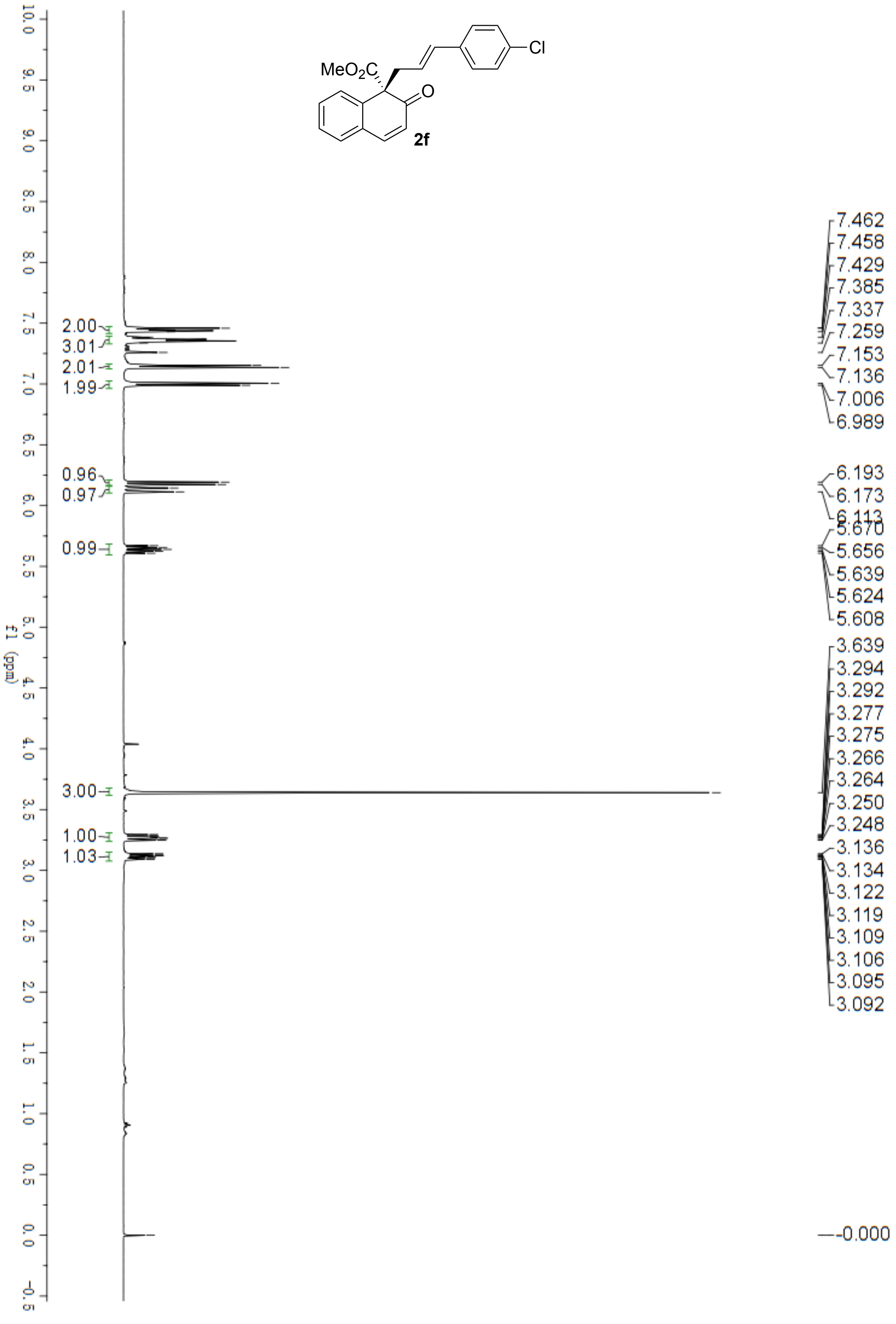
MeO2C
O
Cl
2f
7.462
7.458
7.429
7.385
7.337
7.259
7.153
7.136
7.006
6.989
6.193
6.173
6.113
5.670
5.656
5.639
5.624
5.608
3.639
3.294
3.292
3.277
3.275
3.266
3.264
3.250
3.248
3.136
3.134
3.122
3.119
3.109
3.106
3.095
3.092
-0.000
2.00
3.01
2.01
1.99
0.96
0.97
0.99
3.00
1.00
1.03
f1 (ppm)

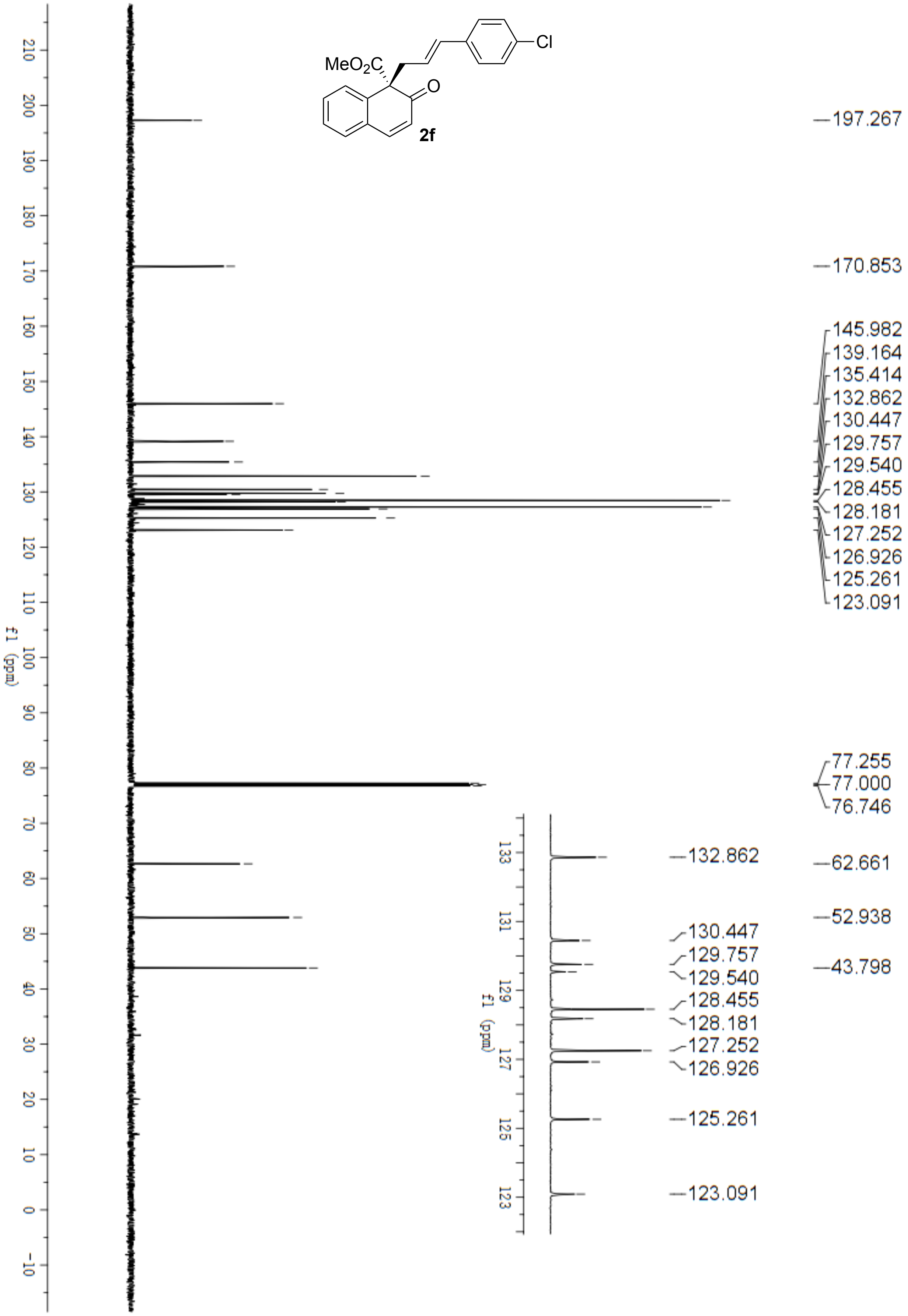
MeO2C
O
Cl
2f
197.267
170.853
145.982
139.164
135.414
132.862
130.447
129.757
129.540
128.455
128.181
127.252
126.926
125.261
123.091
77.255
77.000
76.746
62.661
52.938
43.798
f1 (ppm)
132.862
130.447
129.757
129.540
128.455
128.181
127.252
126.926
125.261
123.091
f1 (ppm)

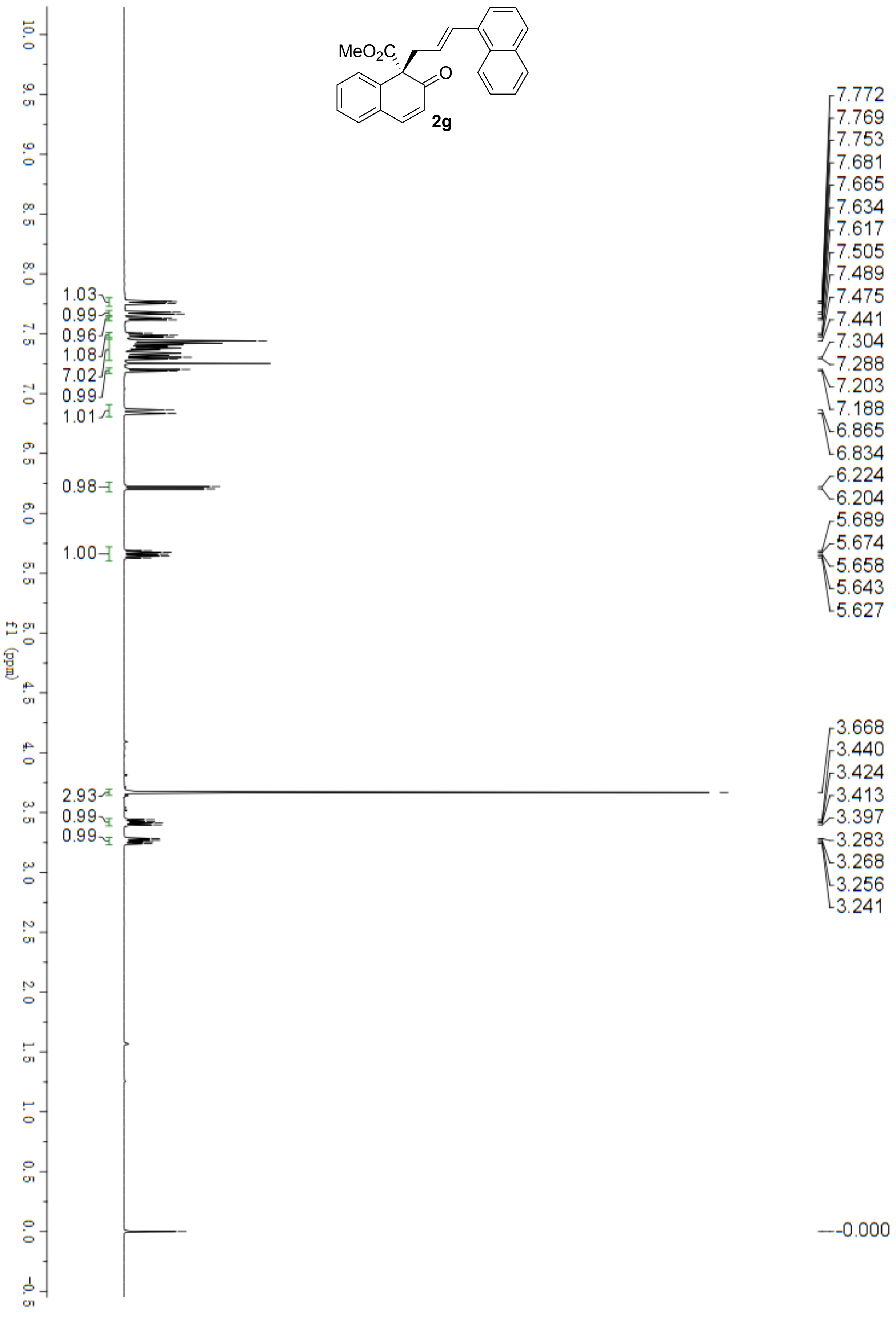
MeO2C
O
2g
7.772
7.769
7.753
7.681
7.665
7.634
7.617
7.505
7.489
7.475
7.441
7.304
7.288
7.203
7.188
6.865
6.834
6.224
6.204
5.689
5.674
5.658
5.643
5.627
3.668
3.440
3.424
3.413
3.397
3.283
3.268
3.256
3.241
-0.000
1.03
0.99
0.96
1.08
7.02
0.99
1.01
0.98
1.00
2.93
0.99
0.99
f1 (ppm)

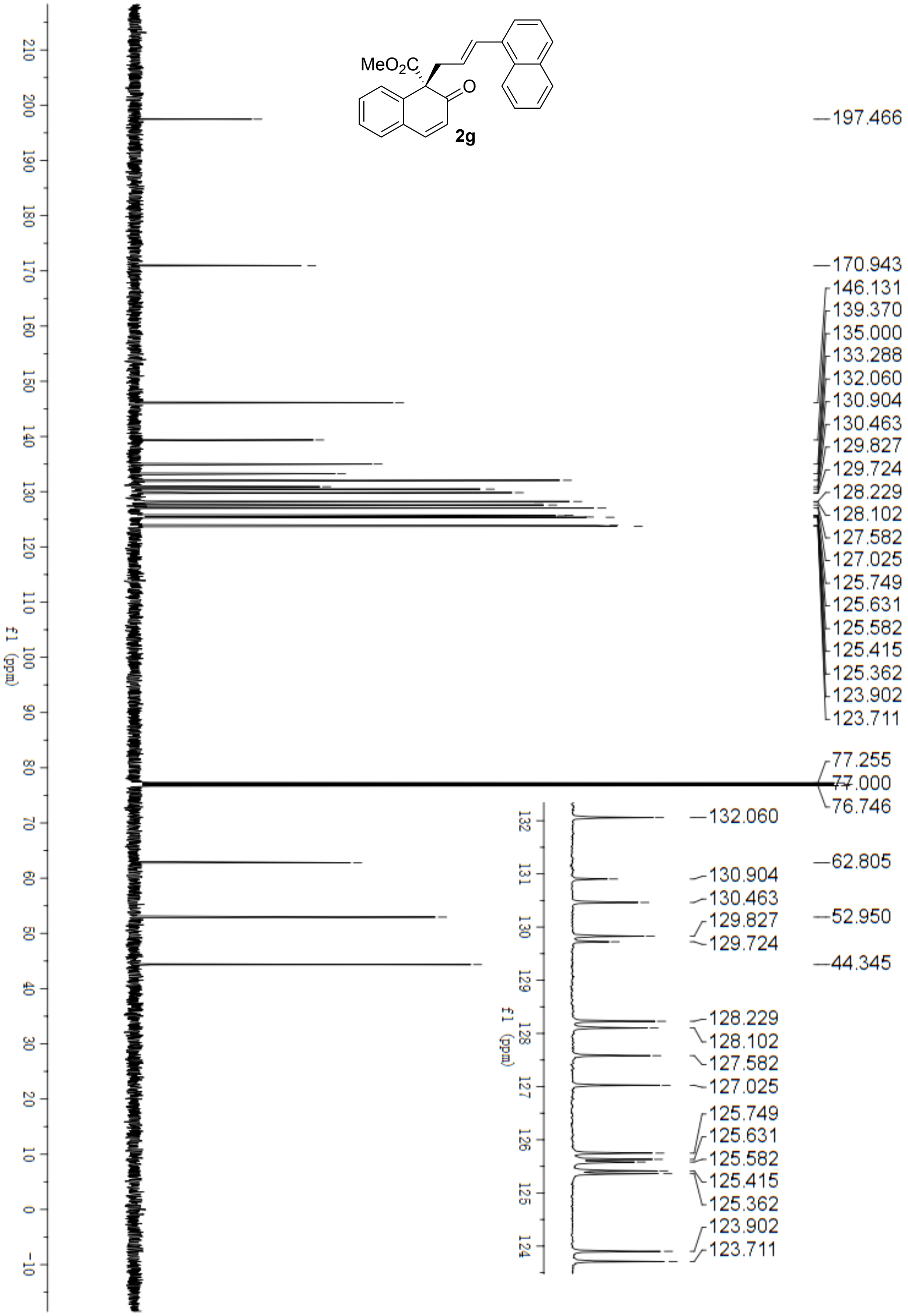
MeO2C
O
2g
197.466
170.943
146.131
139.370
135.000
133.288
132.060
130.904
130.463
129.827
129.724
128.229
128.102
127.582
127.025
125.749
125.631
125.582
125.415
125.362
123.902
123.711
77.255
77.000
76.746
62.805
52.950
44.345
f1 (ppm)

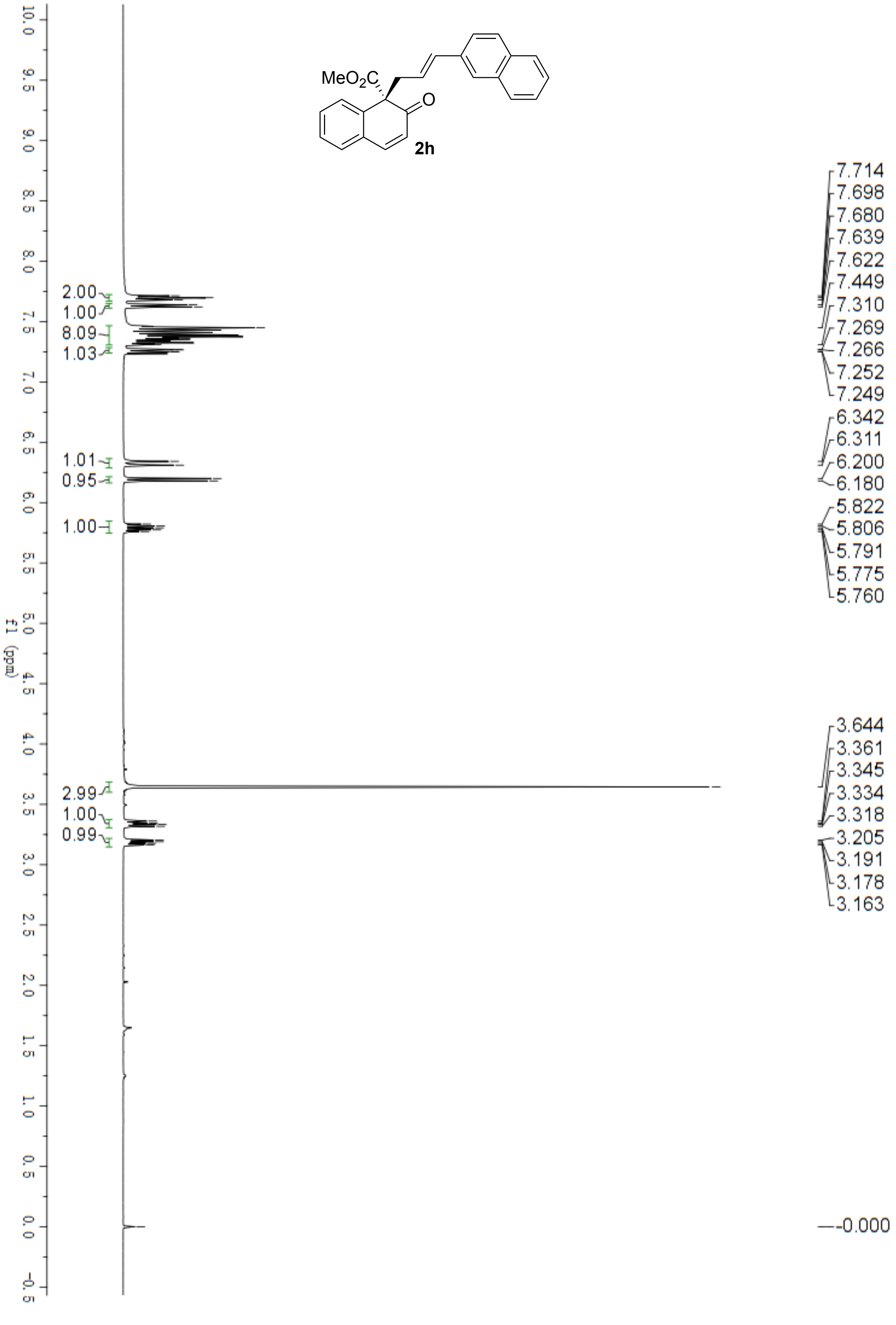
MeO2C
O
2h
7.714
7.698
7.680
7.639
7.622
7.449
7.310
7.269
7.266
7.252
7.249
6.342
6.311
6.200
6.180
5.822
5.806
5.791
5.775
5.760
3.644
3.361
3.345
3.334
3.318
3.205
3.191
3.178
3.163
-0.000
2.00
1.00
8.09
1.03
1.01
0.95
1.00
2.99
1.00
0.99
f1 (ppm)

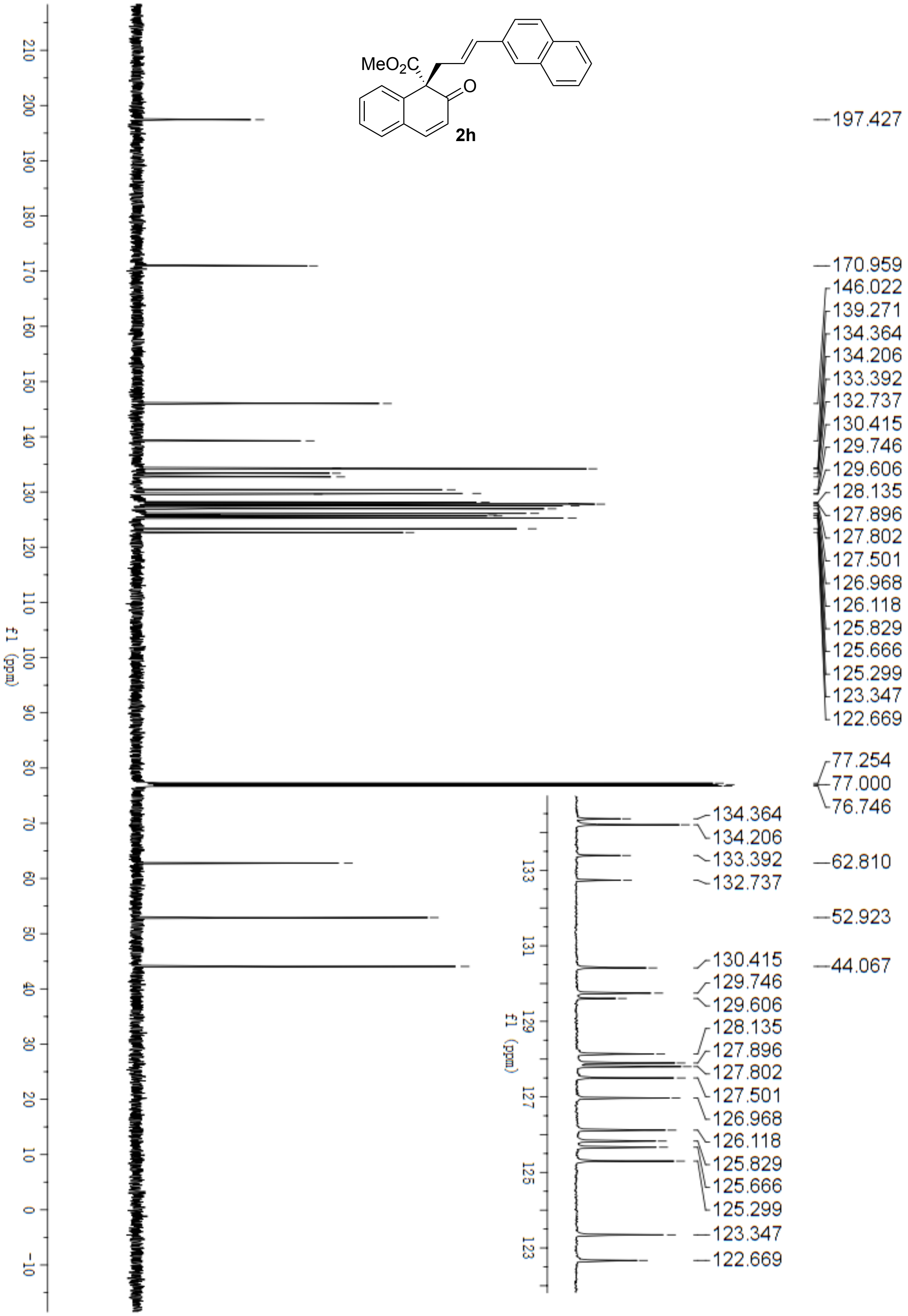

MeO2C
O
2h
197.427
170.959
146.022
139.271
134.364
134.206
133.392
132.737
130.415
129.746
129.606
128.135
127.896
127.802
127.501
126.968
126.118
125.829
125.666
125.299
123.347
122.669
77.254
77.000
76.746
62.810
52.923
44.067
f1 (ppm)

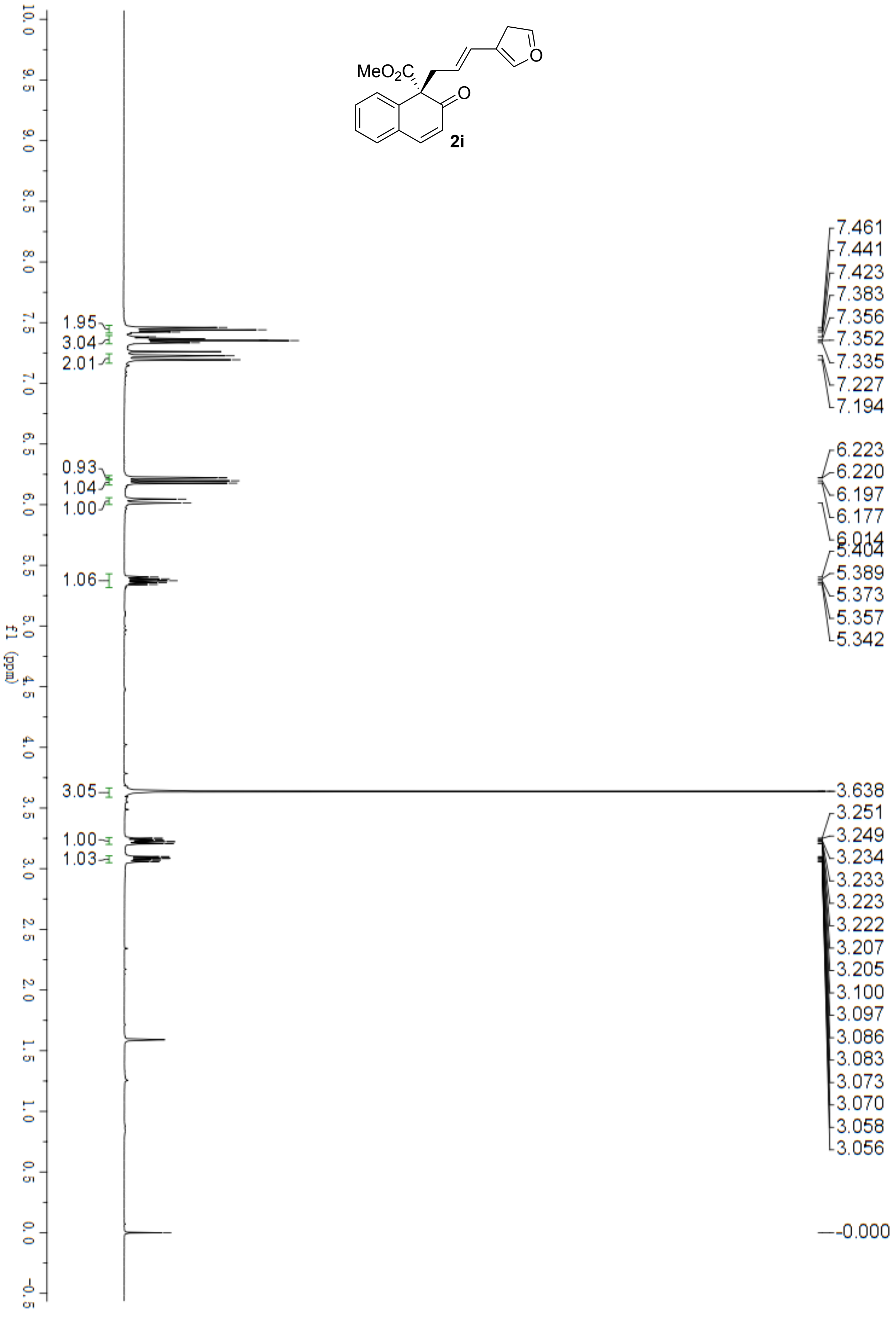

MeO2C
O
O
2i
f1 (ppm)
1.95
3.04
2.01
0.93
1.04
1.00
1.06
3.05
1.00
1.03
7.461
7.441
7.423
7.383
7.356
7.352
7.335
7.227
7.194
6.223
6.220
6.197
6.177
6.014
5.404
5.389
5.373
5.357
5.342
3.638
3.251
3.249
3.234
3.233
3.223
3.222
3.207
3.205
3.100
3.097
3.086
3.083
3.073
3.070
3.058
3.056
-0.000

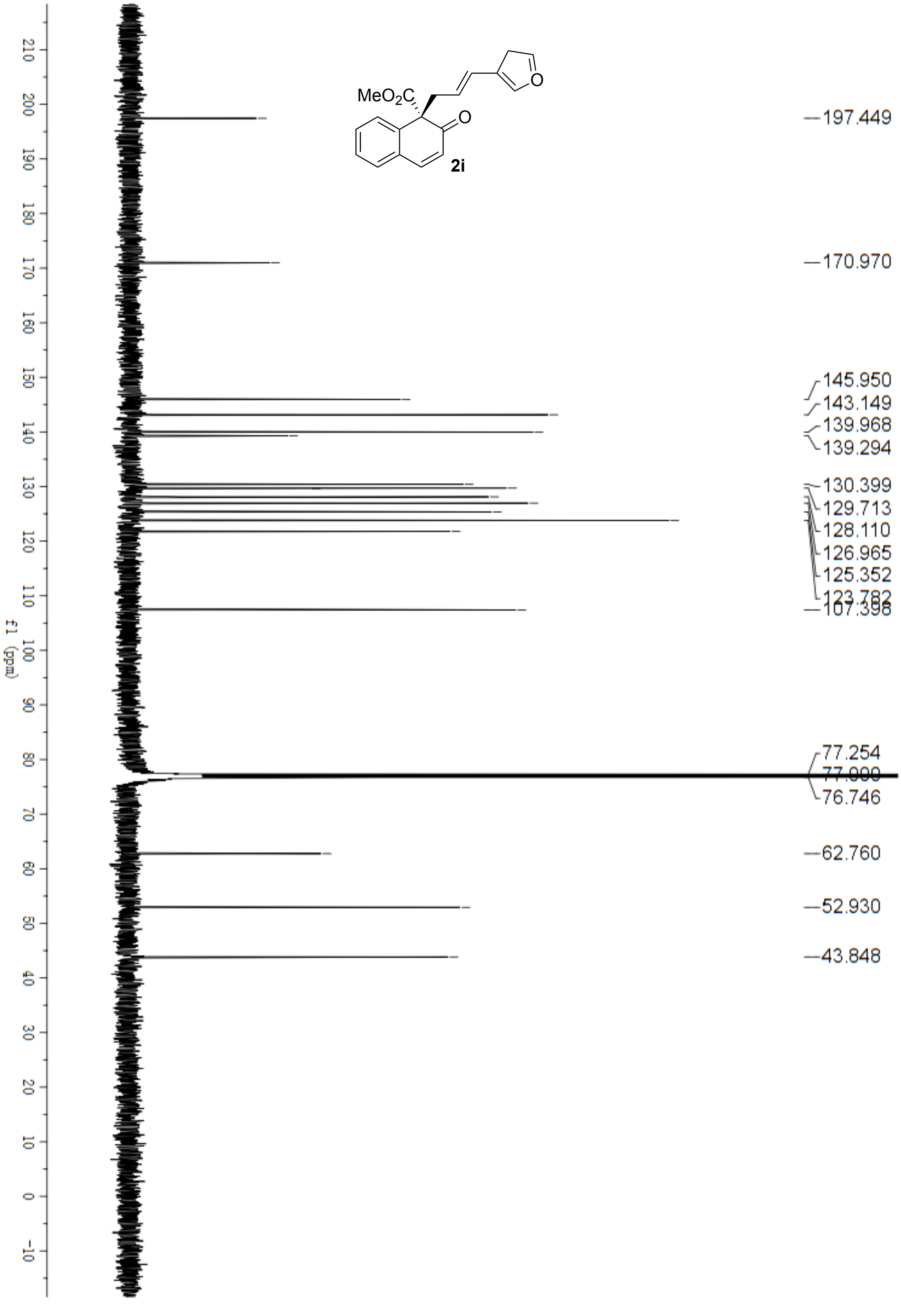
MeO2C
O
O
2i
197.449
170.970
145.950
143.149
139.968
139.294
130.399
129.713
128.110
126.965
125.352
123.782
107.398
77.254
77.000
76.746
62.760
52.930
43.848
f1 (ppm)

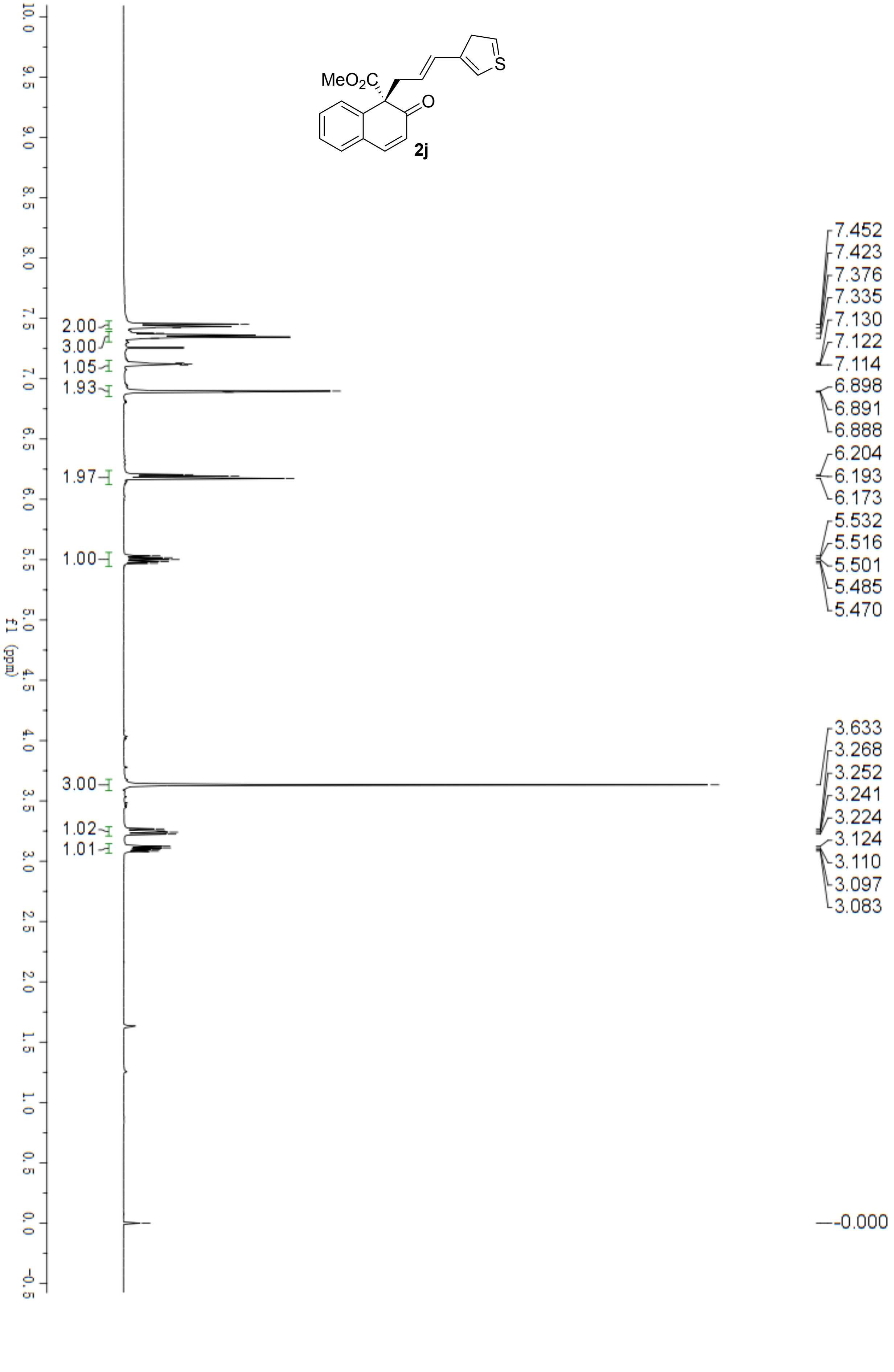
MeO2C
O
S
2j
7.452
7.423
7.376
7.335
7.130
7.122
7.114
6.898
6.891
6.888
6.204
6.193
6.173
5.532
5.516
5.501
5.485
5.470
3.633
3.268
3.252
3.241
3.224
3.124
3.110
3.097
3.083
-0.000
2.00
3.00
1.05
1.93
1.97
1.00
3.00
1.02
1.01
f1 (ppm)

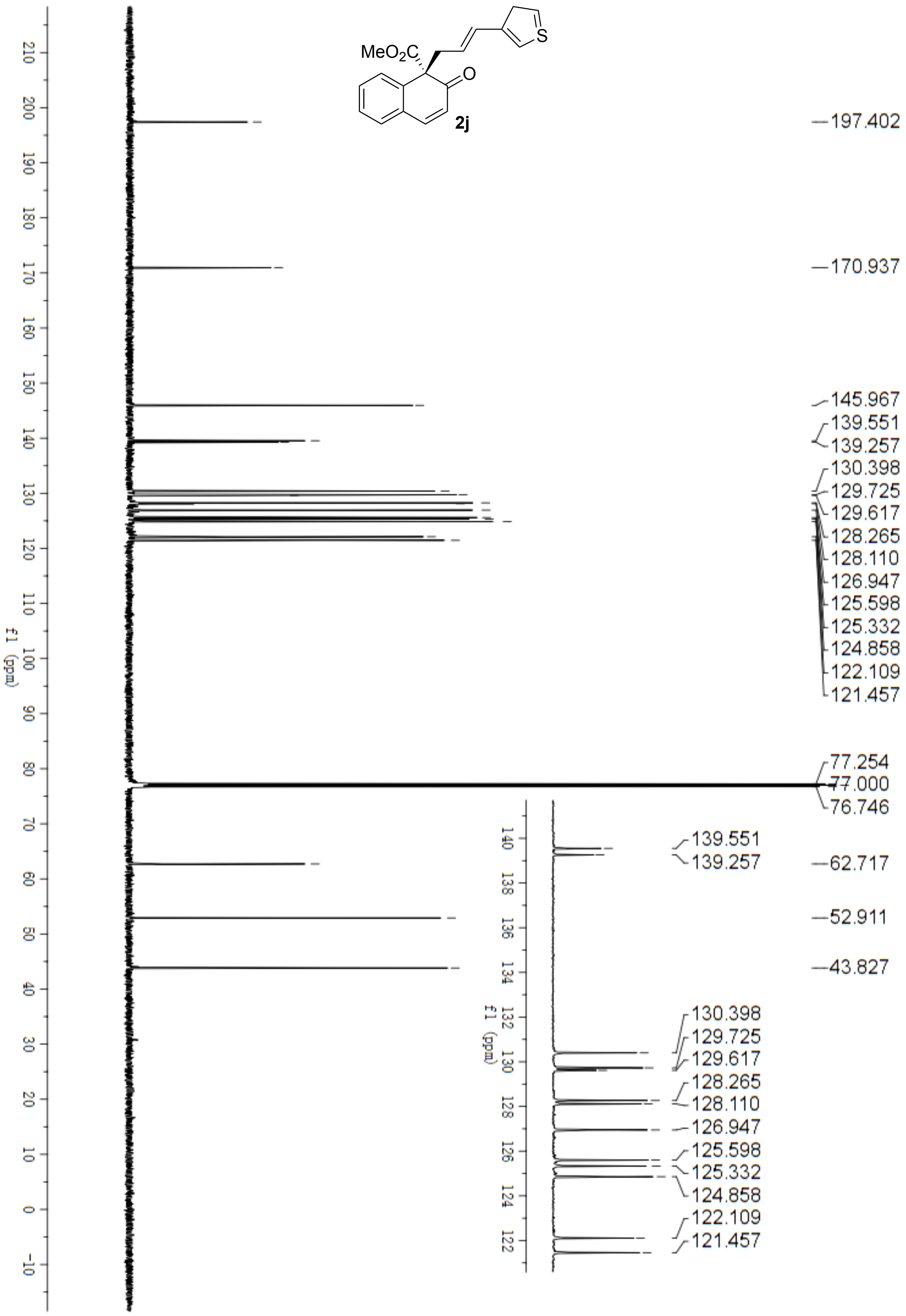
MeO2C
O
S
2j
197.402
170.937
145.967
139.551
139.257
130.398
129.725
129.617
128.265
128.110
126.947
125.598
125.332
124.858
122.109
121.457
77.254
77.000
76.746
62.717
52.911
43.827
f1 (ppm)

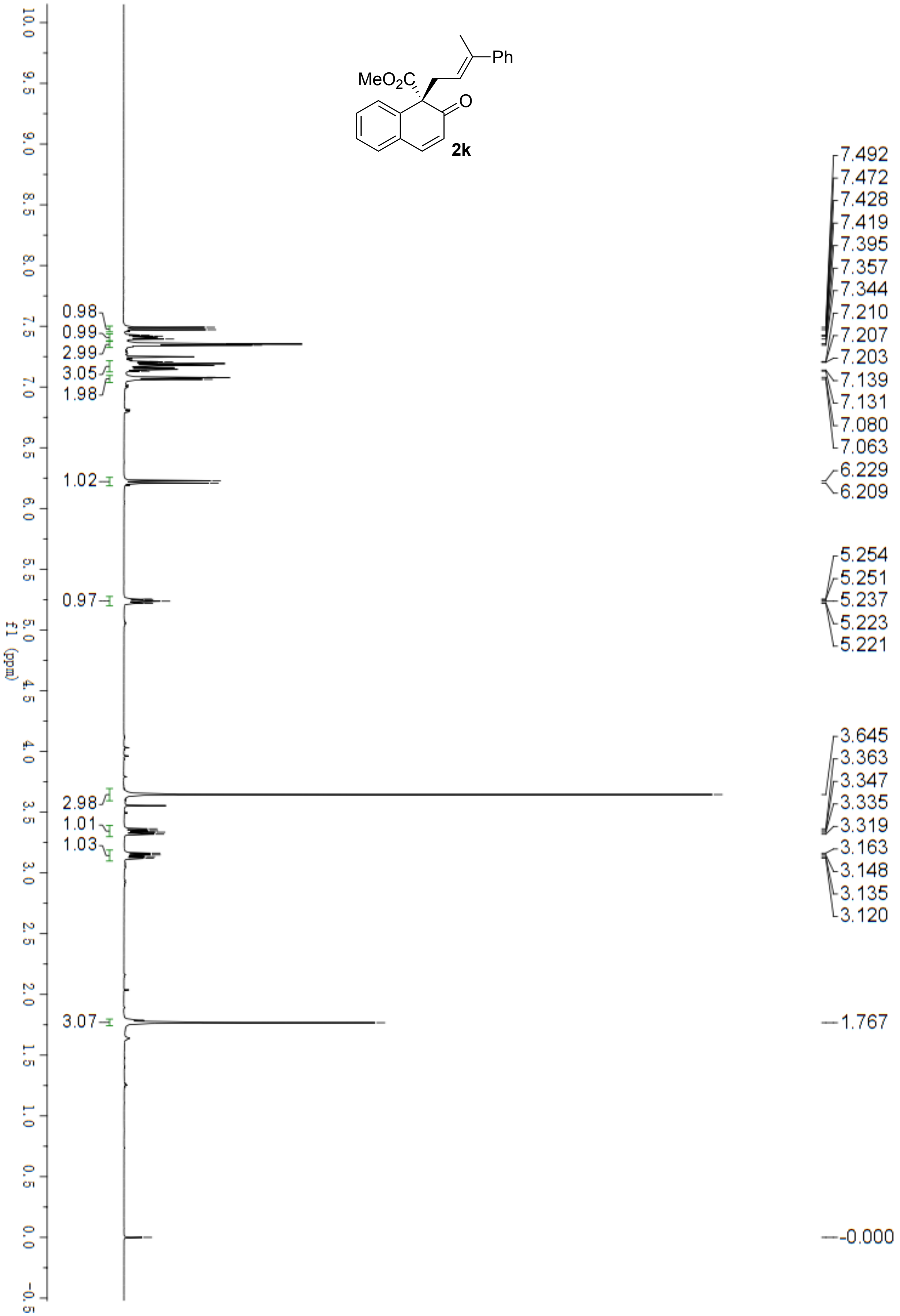
MeO2C
Ph
O
2k
7.492
7.472
7.428
7.419
7.395
7.357
7.344
7.210
7.207
7.203
7.139
7.131
7.080
7.063
6.229
6.209
5.254
5.251
5.237
5.223
5.221
3.645
3.363
3.347
3.335
3.319
3.163
3.148
3.135
3.120
1.767
-0.000
0.98
0.99
2.99
3.05
1.98
1.02
0.97
2.98
1.01
1.03
3.07
f1 (ppm)

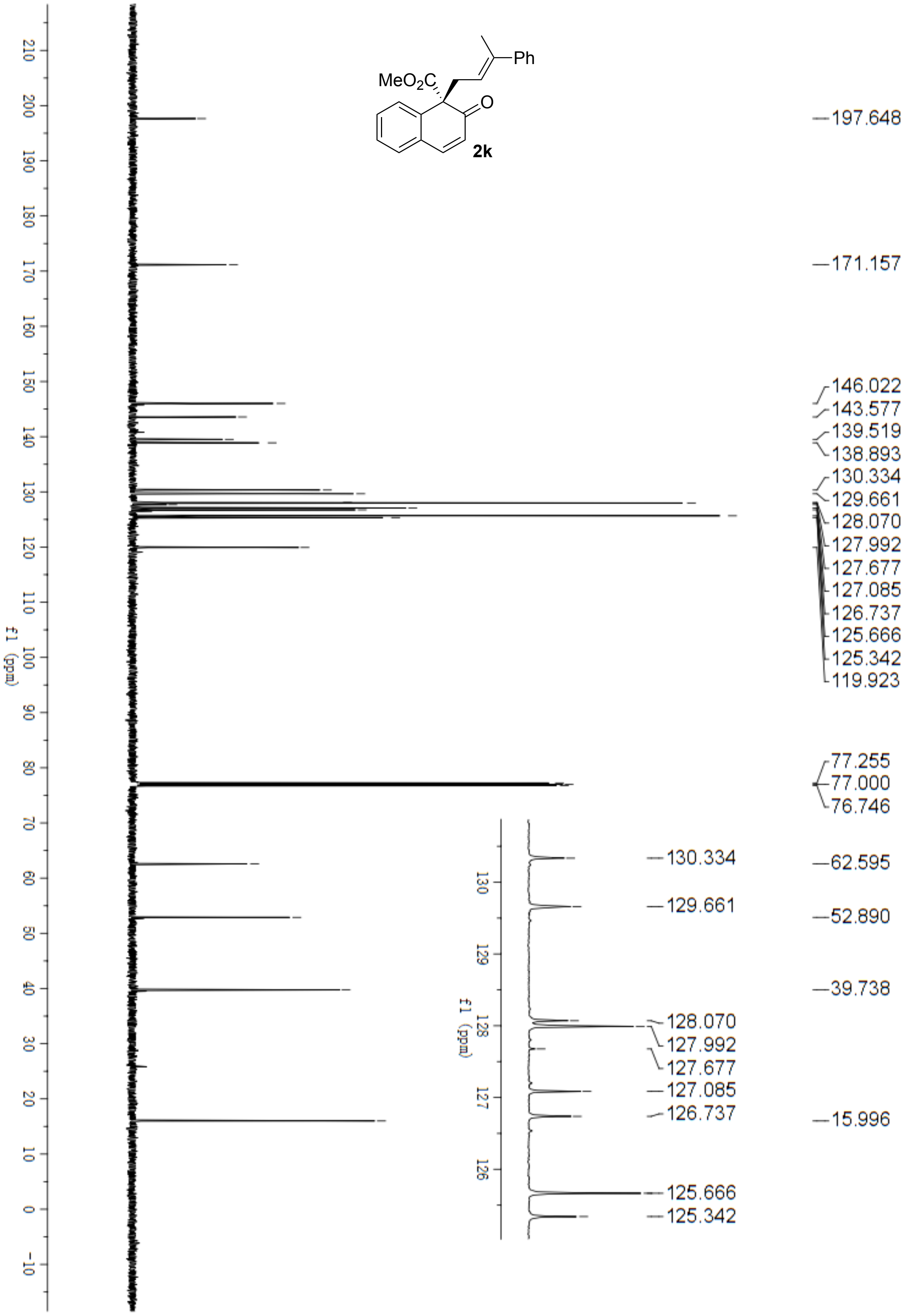
MeO2C
Ph
O
2k
197.648
171.157
146.022
143.577
139.519
138.893
130.334
129.661
128.070
127.992
127.677
127.085
126.737
125.666
125.342
119.923
77.255
77.000
76.746
62.595
52.890
39.738
15.996
f1 (ppm)

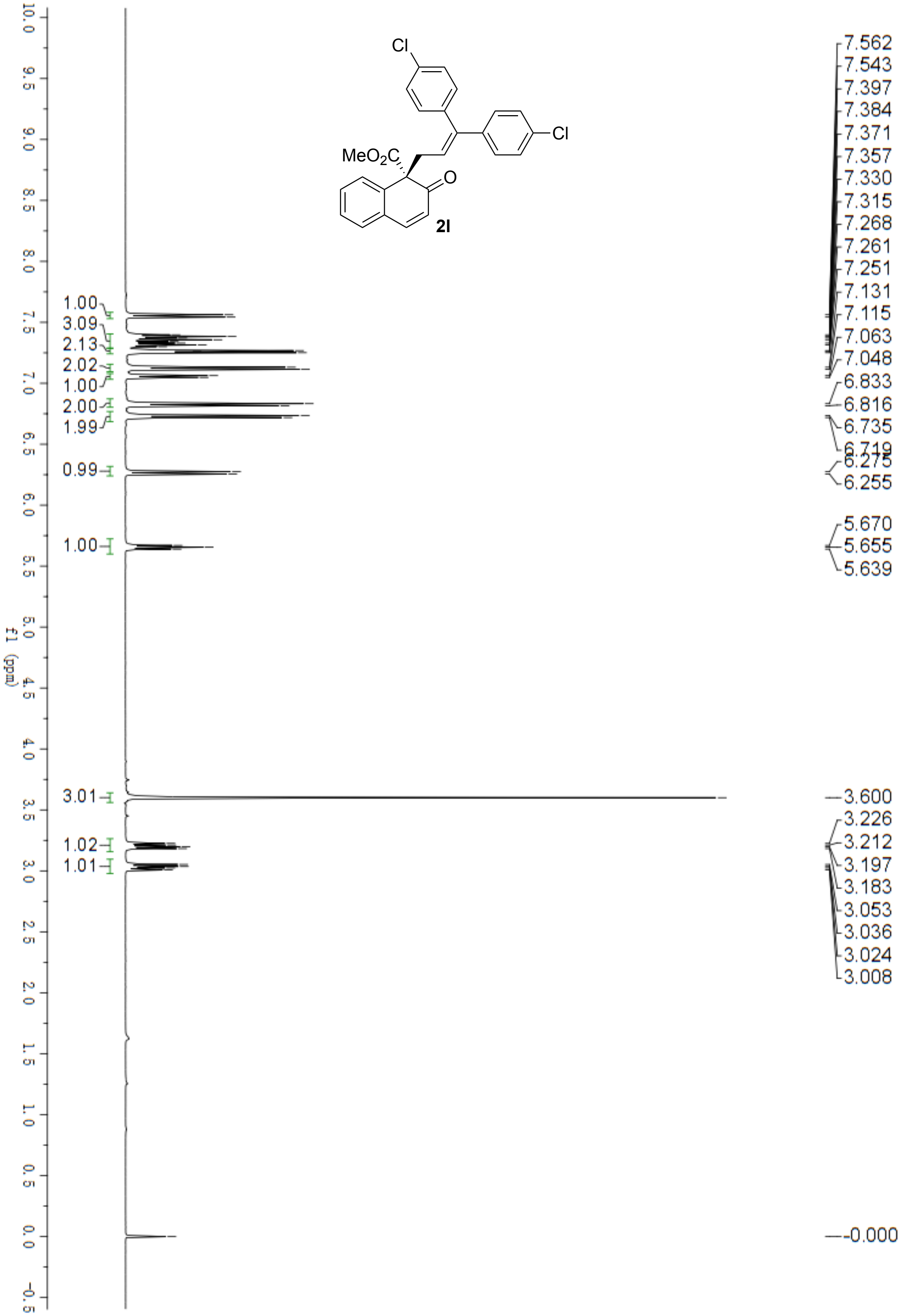
Cl
Cl
MeO2C
O
2l
7.562
7.543
7.397
7.384
7.371
7.357
7.330
7.315
7.268
7.261
7.251
7.131
7.115
7.063
7.048
6.833
6.816
6.735
6.719
6.275
6.255
5.670
5.655
5.639
3.600
3.226
3.212
3.197
3.183
3.053
3.036
3.024
3.008
-0.000
1.00
3.09
2.13
2.02
1.00
2.00
1.99
0.99
1.00
3.01
1.02
1.01
f1 (ppm)

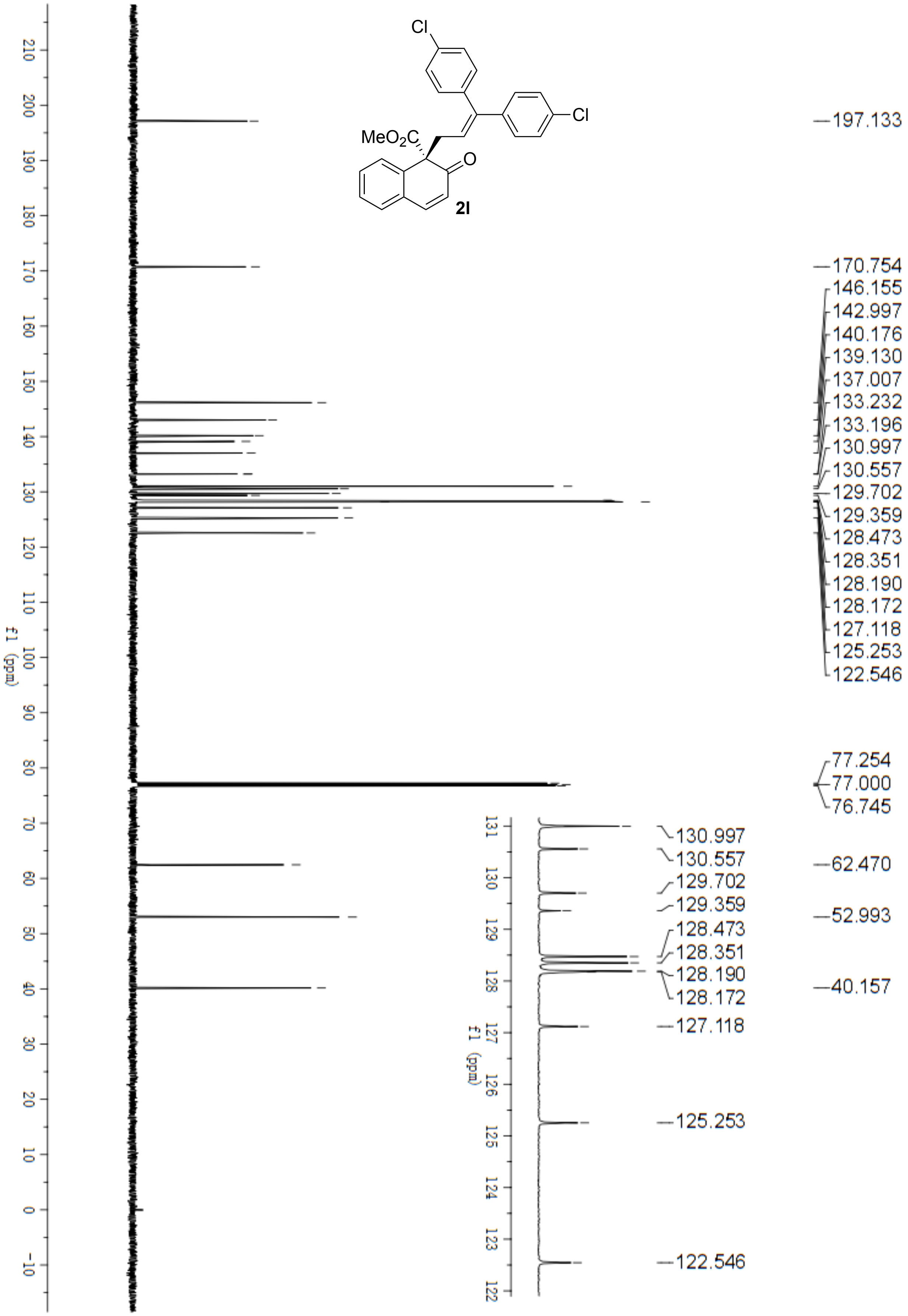
Cl
Cl
MeO2C
O
2l
197.133
170.754
146.155
142.997
140.176
139.130
137.007
133.232
133.196
130.997
130.557
129.702
129.359
128.473
128.351
128.190
128.172
127.118
125.253
122.546
77.254
77.000
76.745
62.470
52.993
40.157
f1 (ppm)

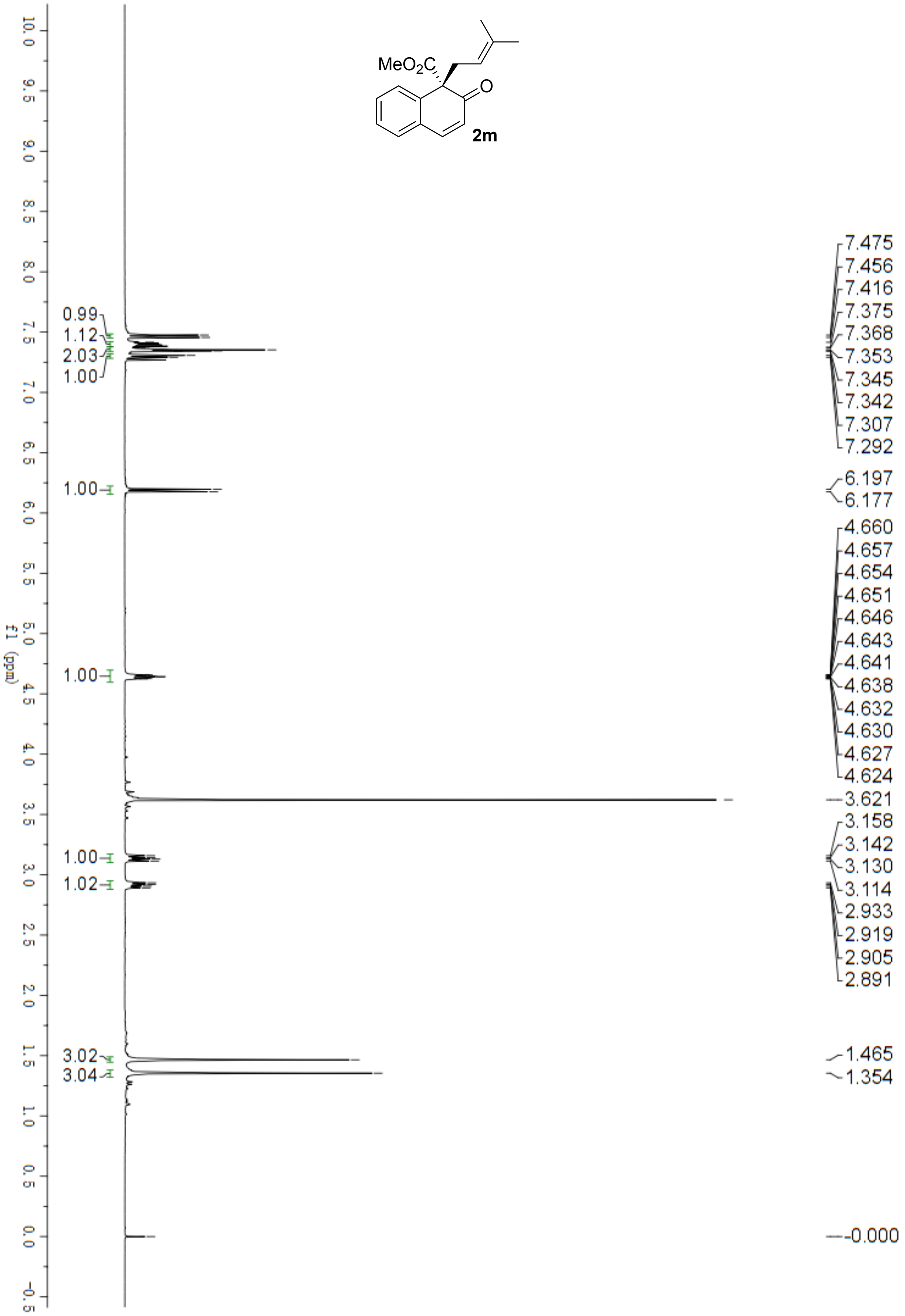
MeO2C
O
2m
f1 (ppm)
10.0
9.5
9.0
8.5
8.0
7.5
7.0
6.5
6.0
5.5
5.0
4.5
4.0
3.5
3.0
2.5
2.0
1.5
1.0
0.5
0.0
-0.5
0.99
1.12
2.03
1.00
1.00
1.00
1.00
1.02
3.02
3.04
7.475
7.456
7.416
7.375
7.368
7.353
7.345
7.342
7.307
7.292
6.197
6.177
4.660
4.657
4.654
4.651
4.646
4.643
4.641
4.638
4.632
4.630
4.627
4.624
3.621
3.158
3.142
3.130
3.114
2.933
2.919
2.905
2.891
1.465
1.354
-0.000

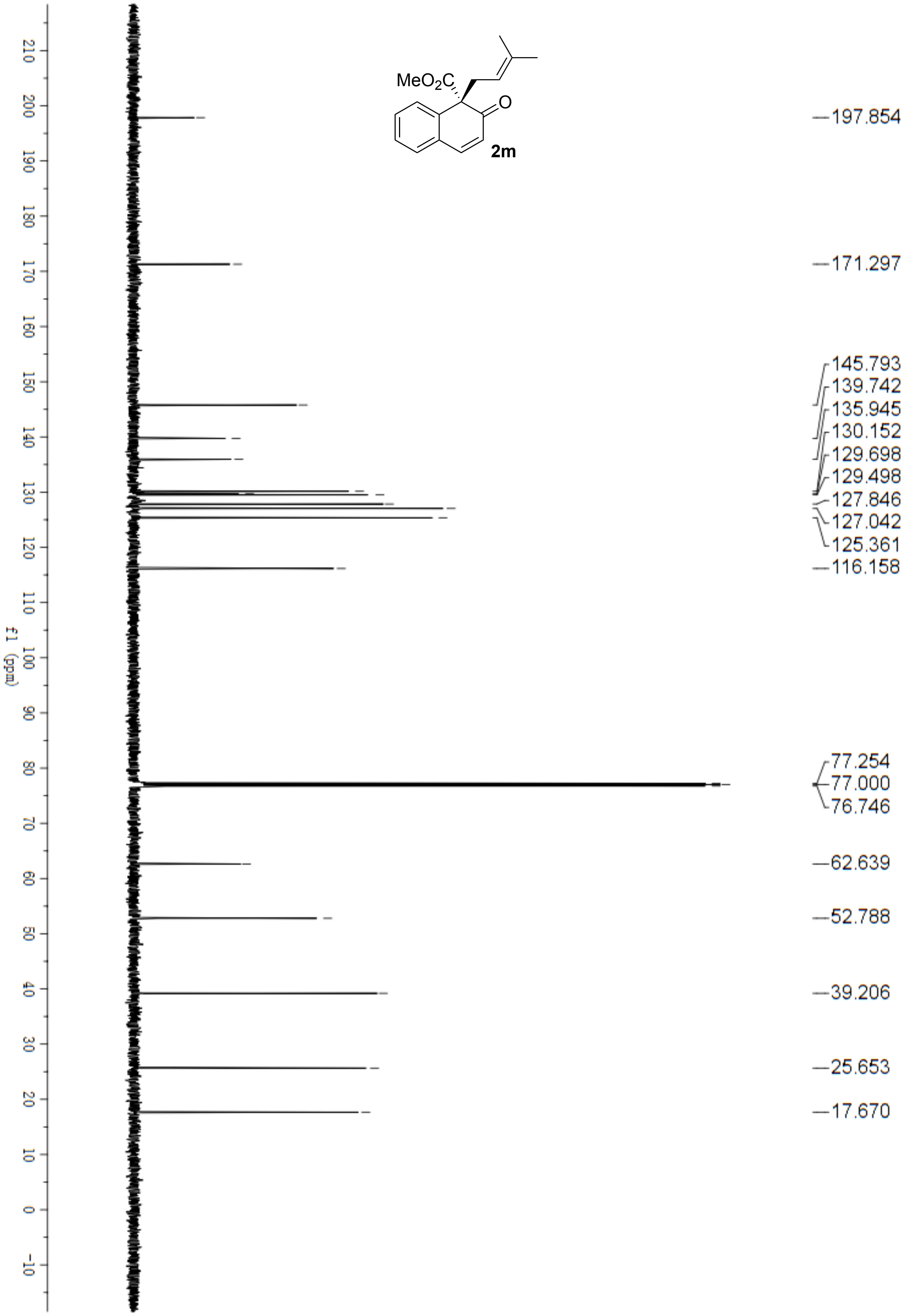
MeO2C
O
2m
197.854
171.297
145.793
139.742
135.945
130.152
129.698
129.498
127.846
127.042
125.361
116.158
77.254
77.000
76.746
62.639
52.788
39.206
25.653
17.670
f1 (ppm)

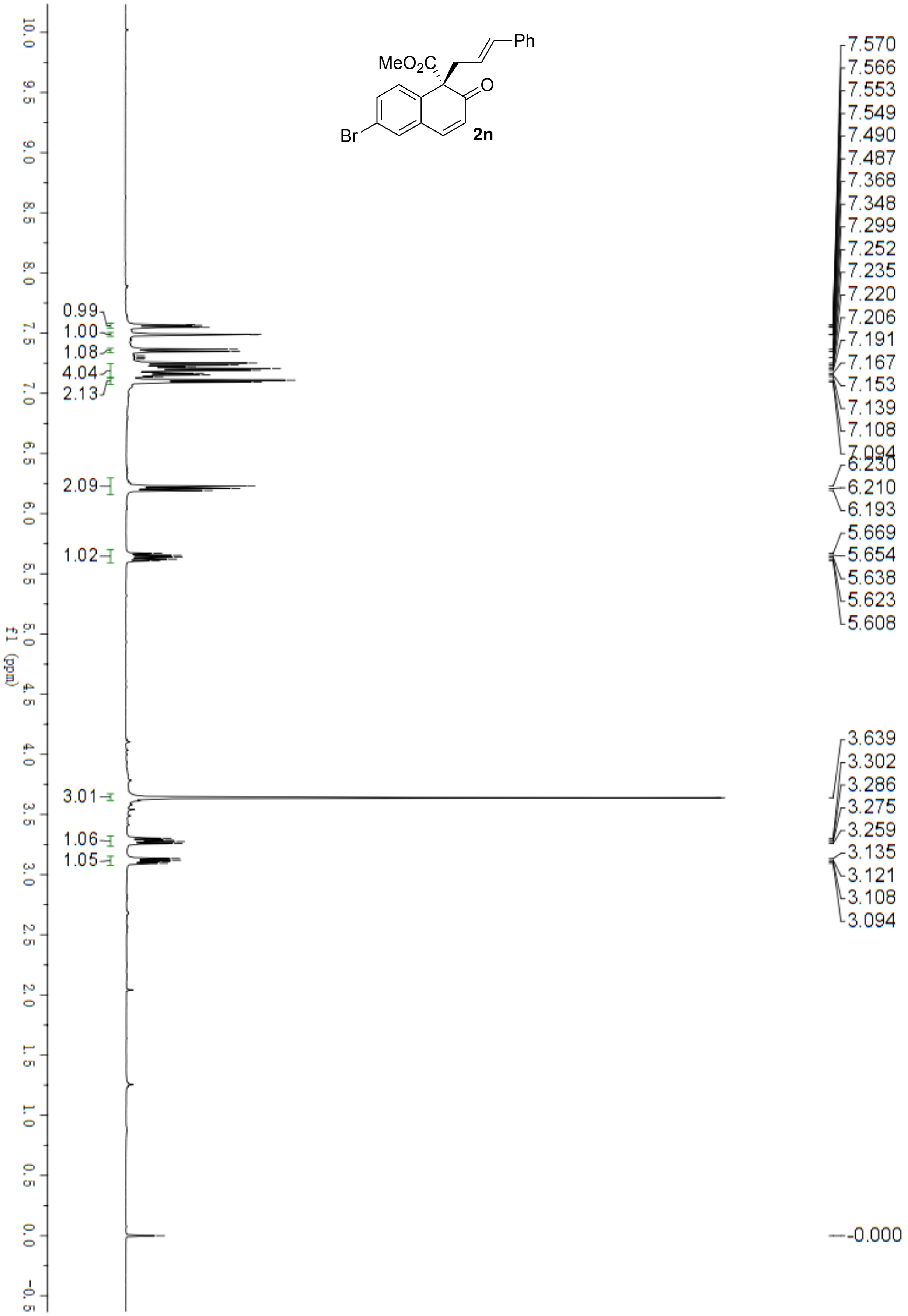
MeO2C
Ph
O
Br
2n
7.570
7.566
7.553
7.549
7.490
7.487
7.368
7.348
7.299
7.252
7.235
7.220
7.206
7.191
7.167
7.153
7.139
7.108
7.094
6.230
6.210
6.193
5.669
5.654
5.638
5.623
5.608
3.639
3.302
3.286
3.275
3.259
3.135
3.121
3.108
3.094
-0.000
0.99
1.00
1.08
4.04
2.13
2.09
1.02
3.01
1.06
1.05
f1 (ppm)

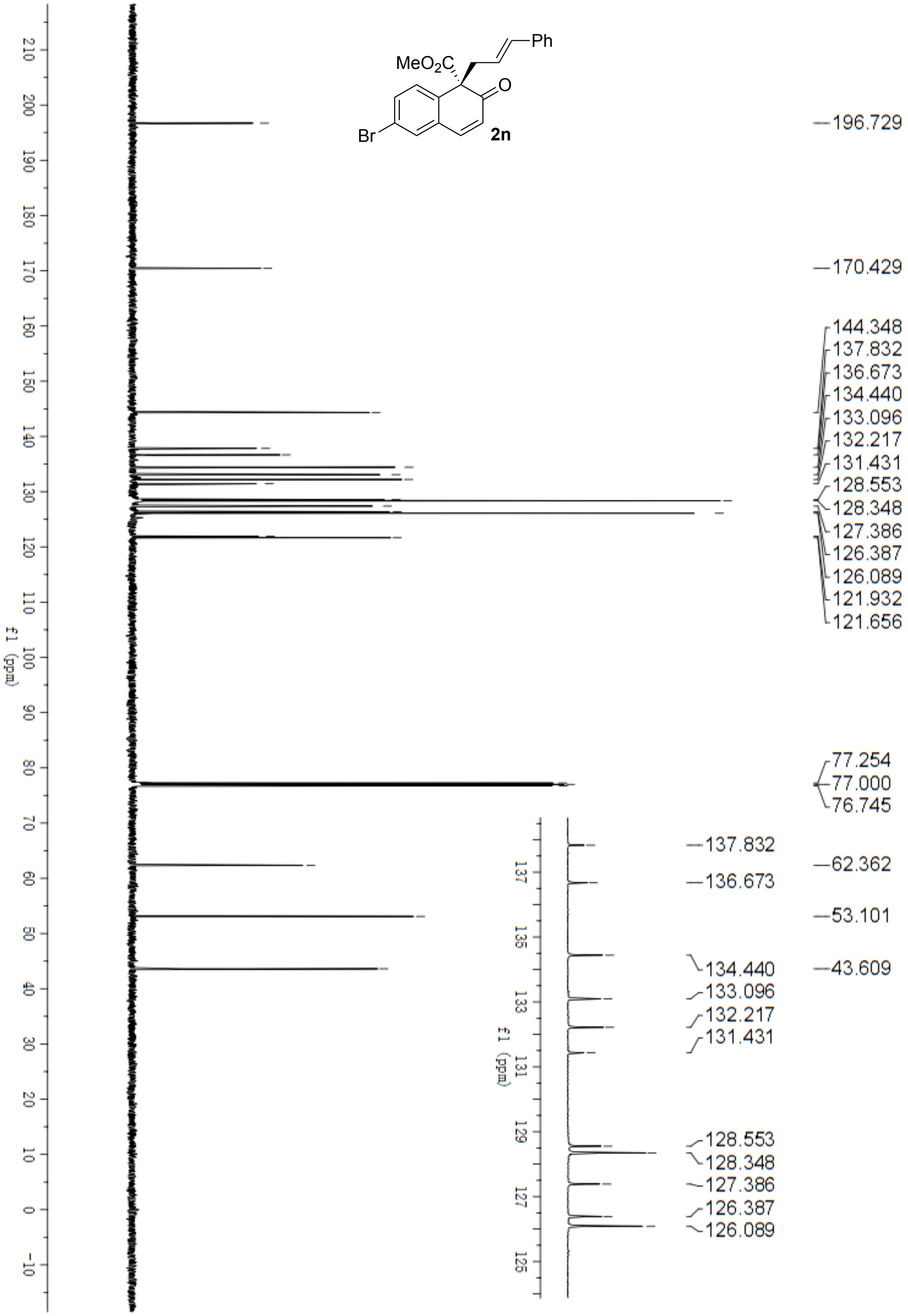
MeO2C
Ph
O
Br
2n
196.729
170.429
144.348
137.832
136.673
134.440
133.096
132.217
131.431
128.553
128.348
127.386
126.387
126.089
121.932
121.656
77.254
77.000
76.745
62.362
53.101
43.609
f1 (ppm)

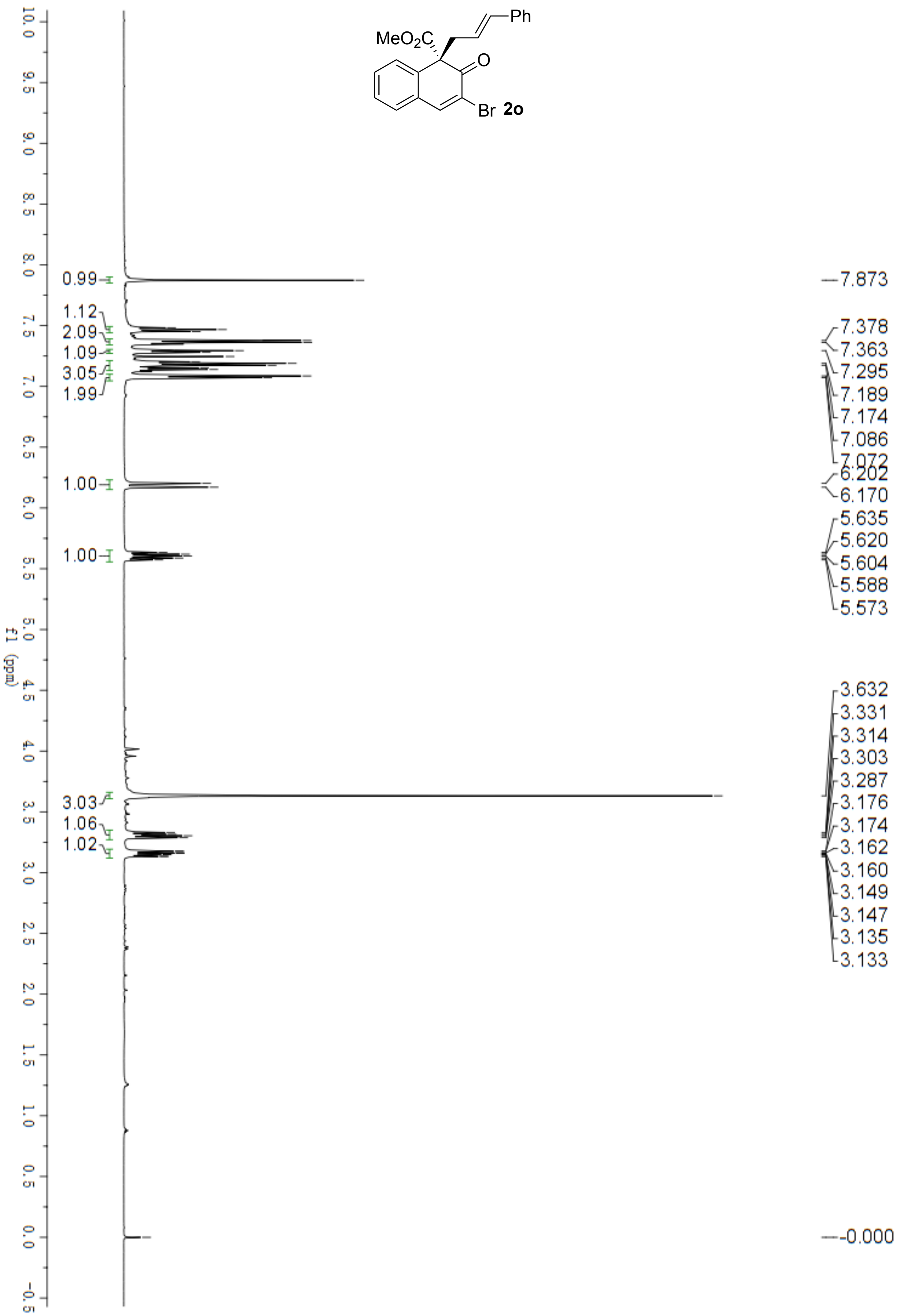
MeO2C
Ph
O
Br
2o
0.99
1.12
2.09
1.09
3.05
1.99
1.00
1.00
3.03
1.06
1.02
f1 (ppm)
10.0
9.5
9.0
8.5
8.0
7.5
7.0
6.5
6.0
5.5
5.0
4.5
4.0
3.5
3.0
2.5
2.0
1.5
1.0
0.5
0.0
-0.5
7.873
7.378
7.363
7.295
7.189
7.174
7.086
7.072
6.202
6.170
5.635
5.620
5.604
5.588
5.573
3.632
3.331
3.314
3.303
3.287
3.176
3.174
3.162
3.160
3.149
3.147
3.135
3.133
-0.000

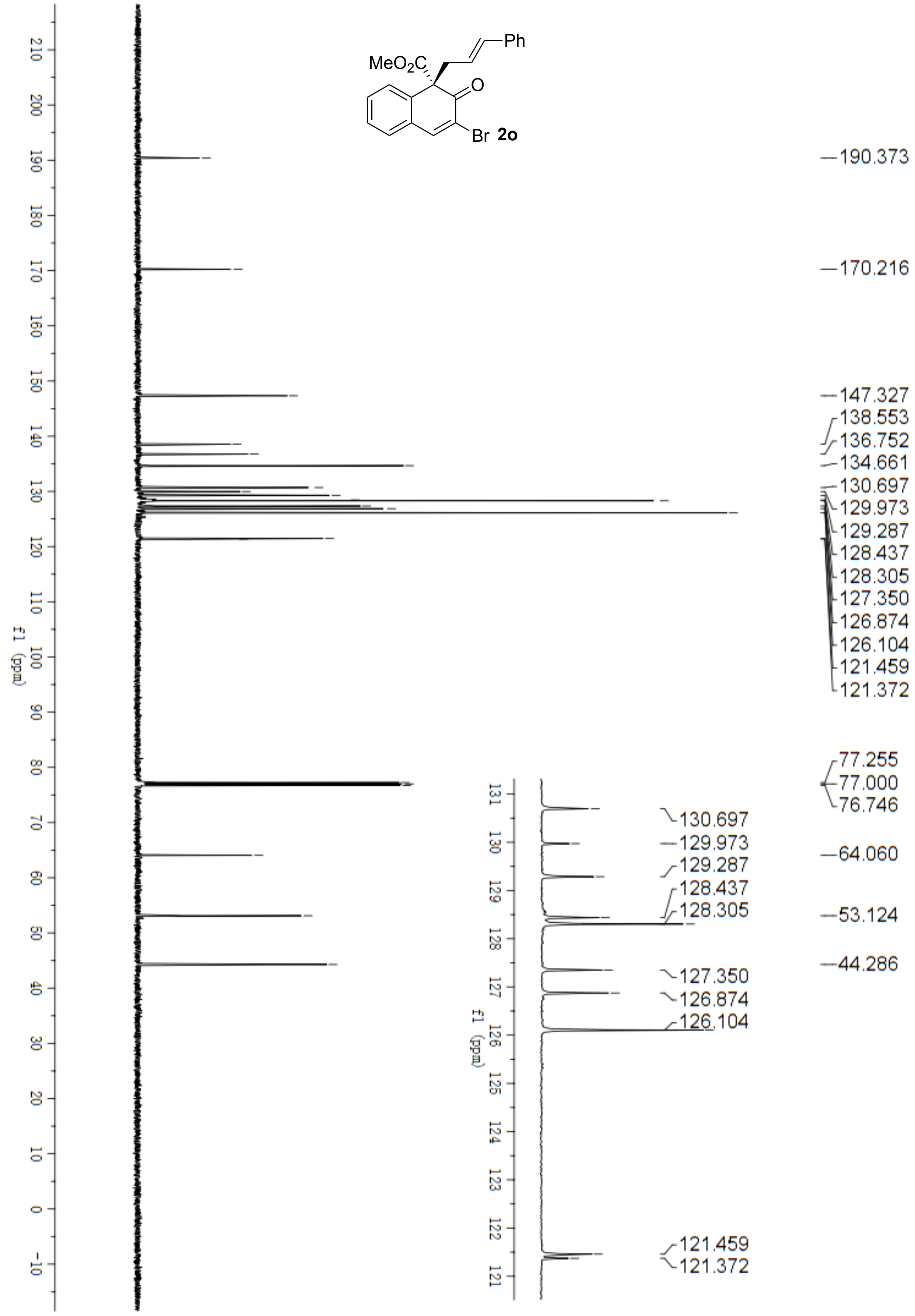

MeO2C
Ph
O
Br 2o
190.373
170.216
147.327
138.553
136.752
134.661
130.697
129.973
129.287
128.437
128.305
127.350
126.874
126.104
121.459
121.372
77.255
77.000
76.746
64.060
53.124
44.286
f1 (ppm)

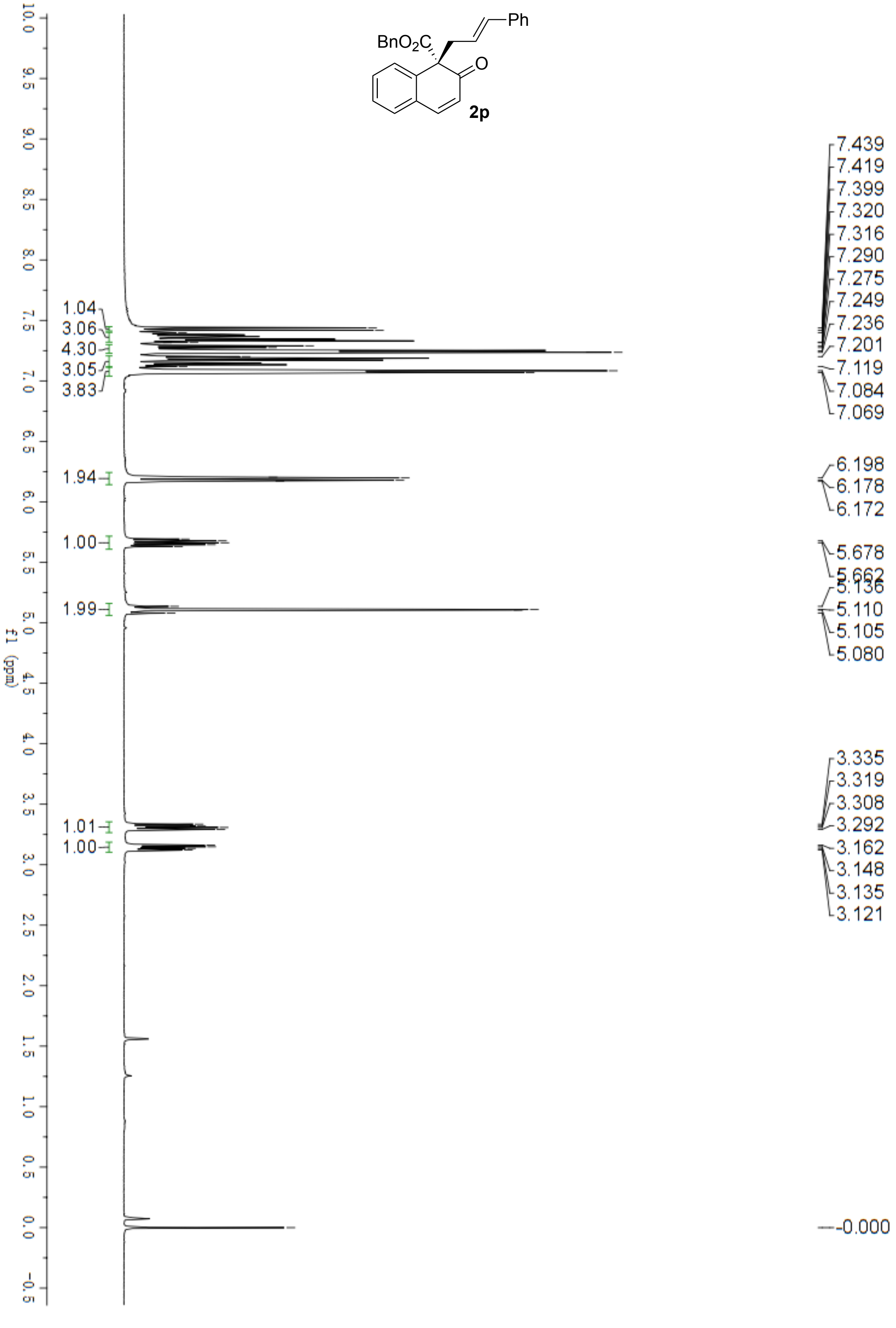

BnO2C
Ph
O
2p
7.439
7.419
7.399
7.320
7.316
7.290
7.275
7.249
7.236
7.201
7.119
7.084
7.069
6.198
6.178
6.172
5.678
5.662
5.136
5.110
5.105
5.080
3.335
3.319
3.308
3.292
3.162
3.148
3.135
3.121
-0.000
1.04
3.06
4.30
3.05
3.83
1.94
1.00
1.99
1.01
1.00
f1 (ppm)

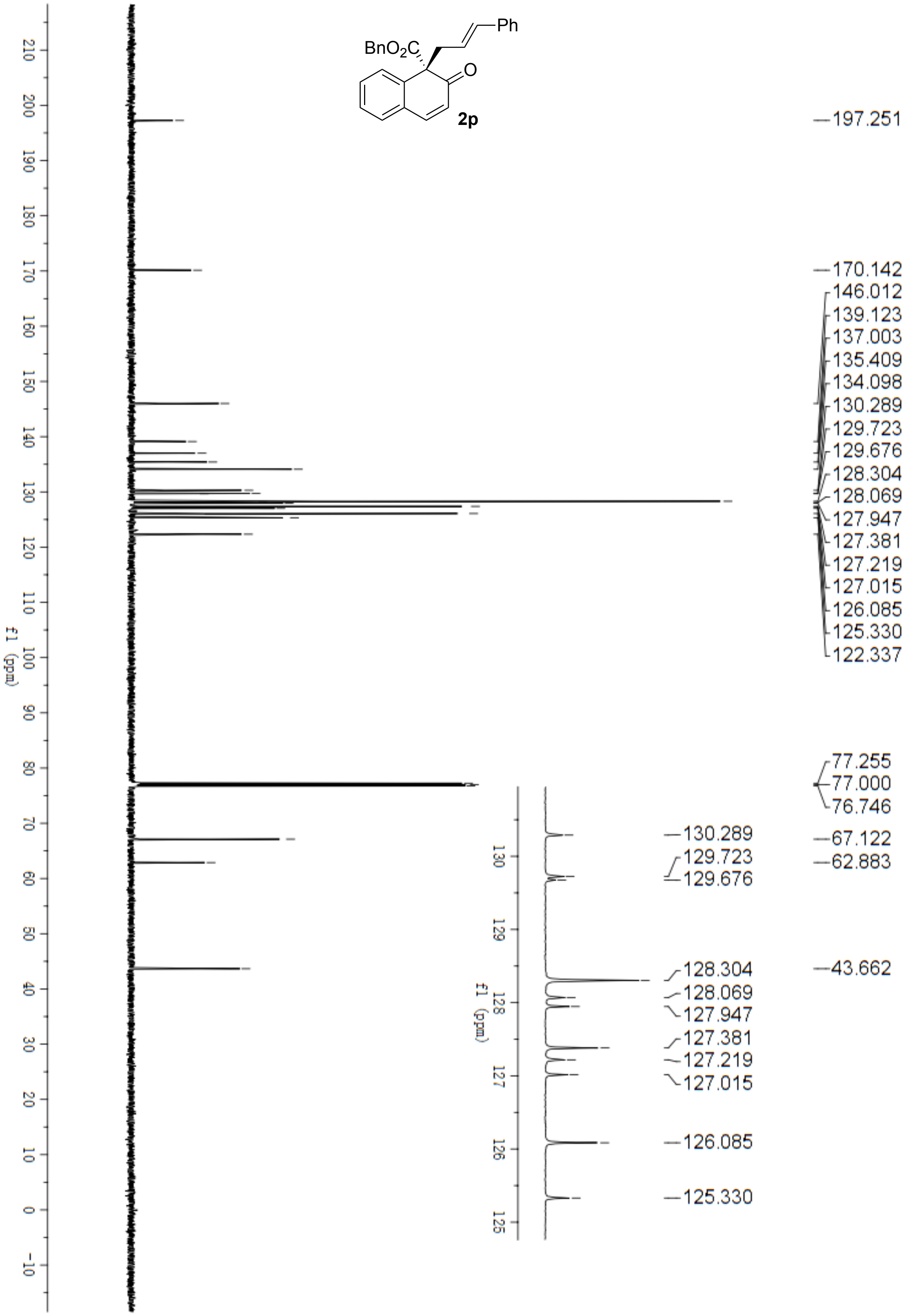

BnO2C
Ph
O
2p
197.251
170.142
146.012
139.123
137.003
135.409
134.098
130.289
129.723
129.676
128.304
128.069
127.947
127.381
127.219
127.015
126.085
125.330
122.337
77.255
77.000
76.746
67.122
62.883
43.662
f1 (ppm)
130.289
129.723
129.676
128.304
128.069
127.947
127.381
127.219
127.015
126.085
125.330
f1 (ppm)

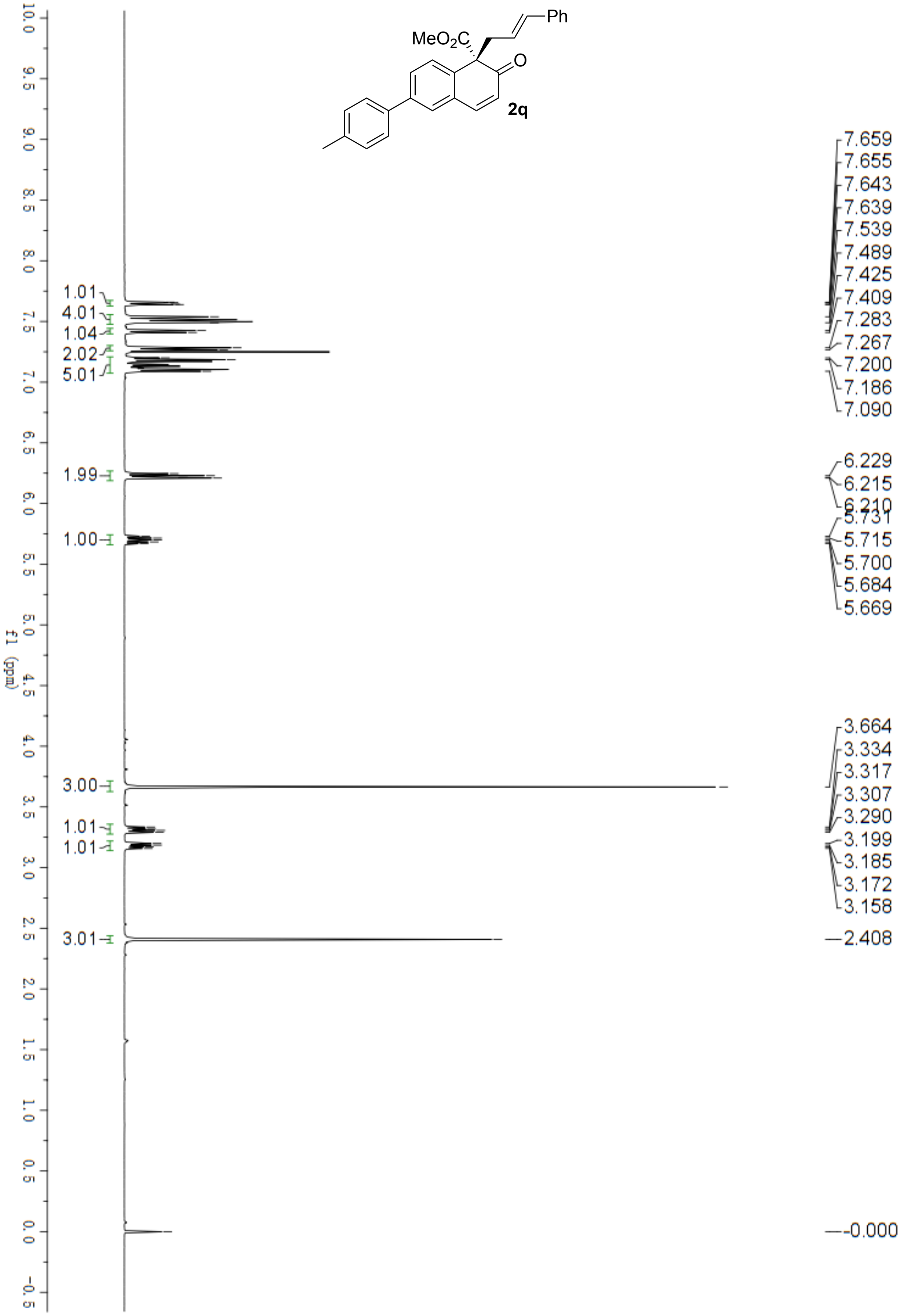
MeO2C
Ph
O
2q
7.659
7.655
7.643
7.639
7.539
7.489
7.425
7.409
7.283
7.267
7.200
7.186
7.090
6.229
6.215
6.210
5.731
5.715
5.700
5.684
5.669
3.664
3.334
3.317
3.307
3.290
3.199
3.185
3.172
3.158
2.408
-0.000
1.01
4.01
1.04
2.02
5.01
1.99
1.00
3.00
1.01
1.01
3.01
f1 (ppm)

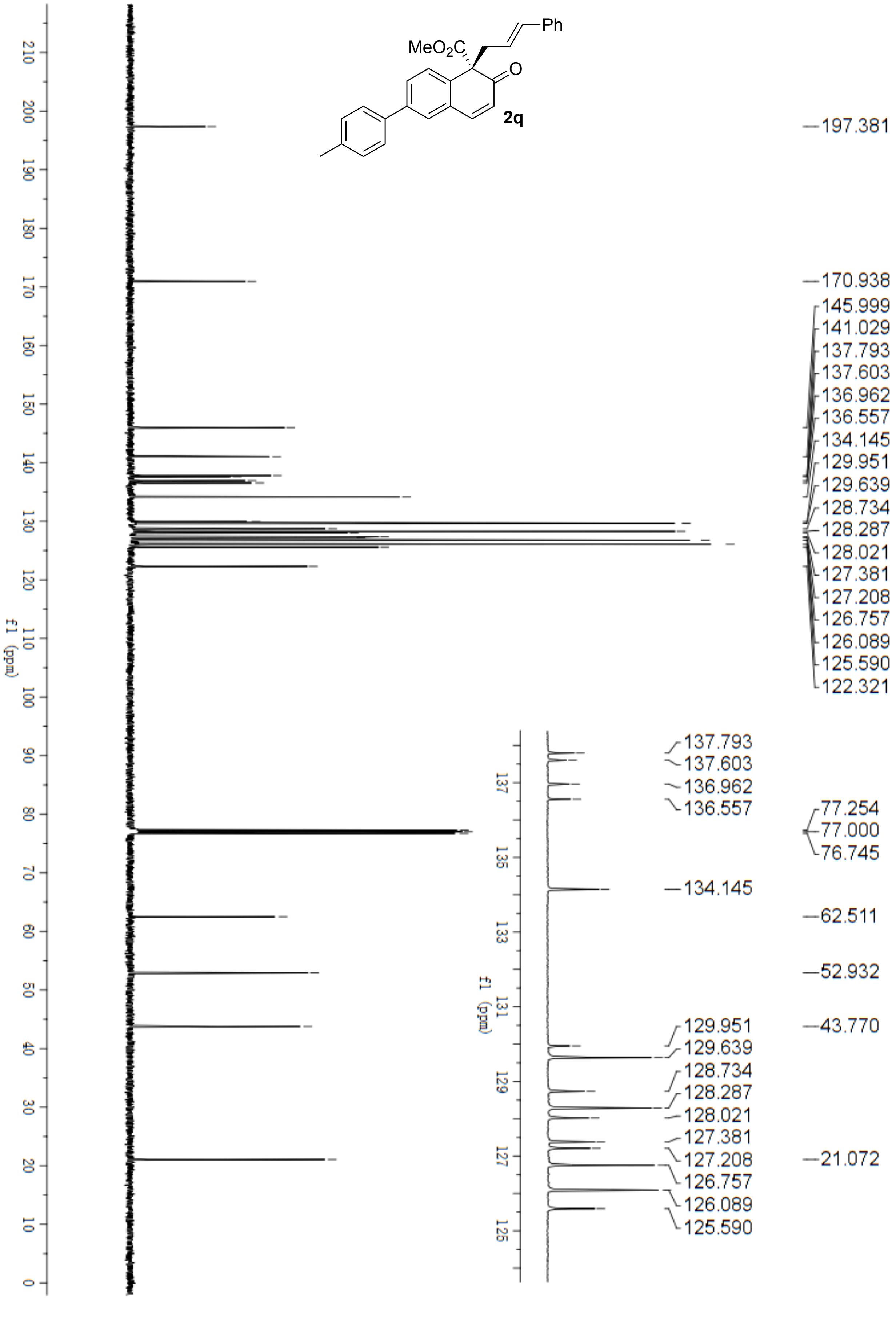
MeO2C
Ph
O
2q
197.381
170.938
145.999
141.029
137.793
137.603
136.962
136.557
134.145
129.951
129.639
128.734
128.287
128.021
127.381
127.208
126.757
126.089
125.590
122.321
77.254
77.000
76.745
62.511
52.932
43.770
21.072
f1 (ppm)

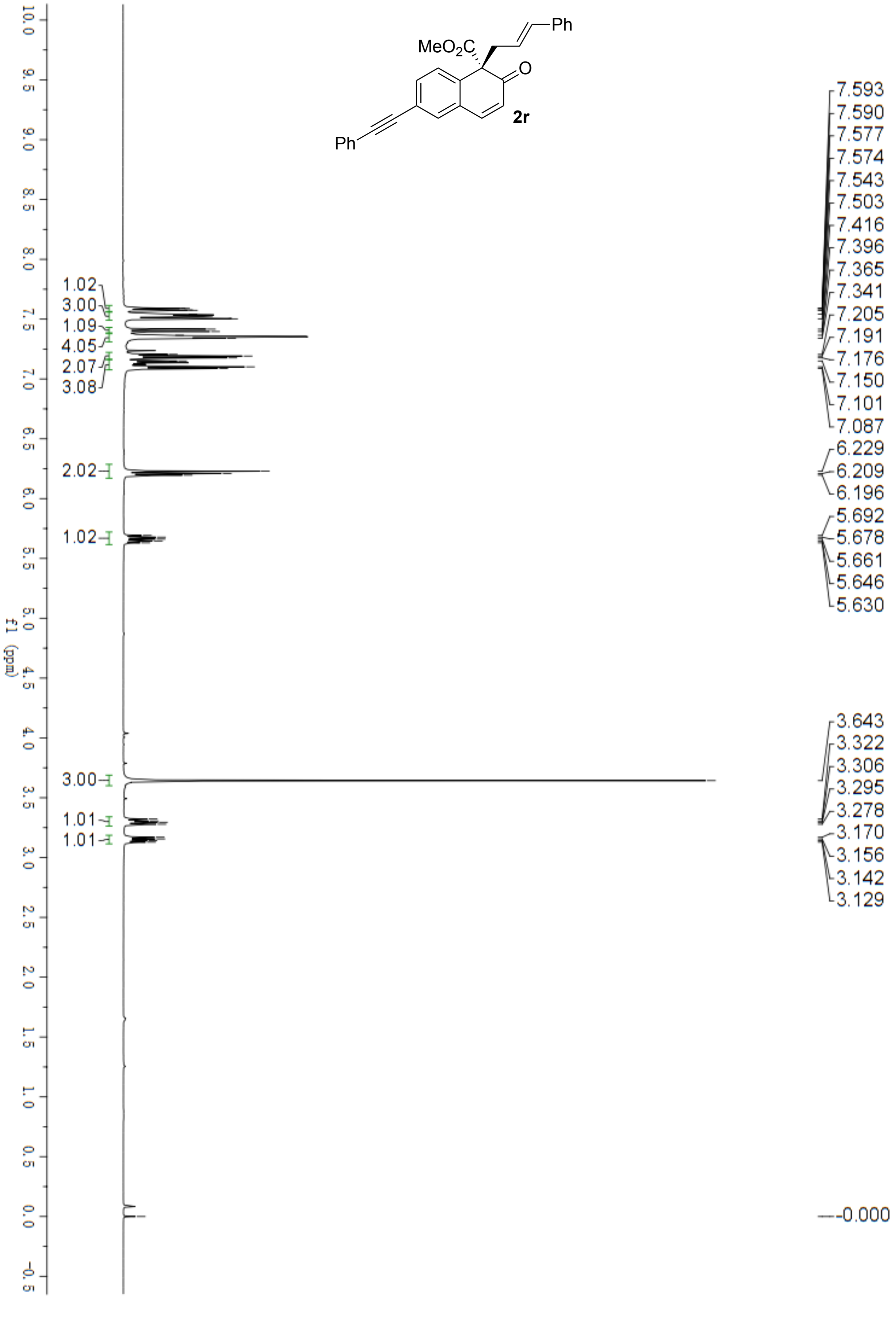
MeO2C
Ph
O
2r
Ph
7.593
7.590
7.577
7.574
7.543
7.503
7.416
7.396
7.365
7.341
7.205
7.191
7.176
7.150
7.101
7.087
6.229
6.209
6.196
5.692
5.678
5.661
5.646
5.630
3.643
3.322
3.306
3.295
3.278
3.170
3.156
3.142
3.129
-0.000
1.02
3.00
1.09
4.05
2.07
3.08
2.02
1.02
3.00
1.01
1.01
f1 (ppm)

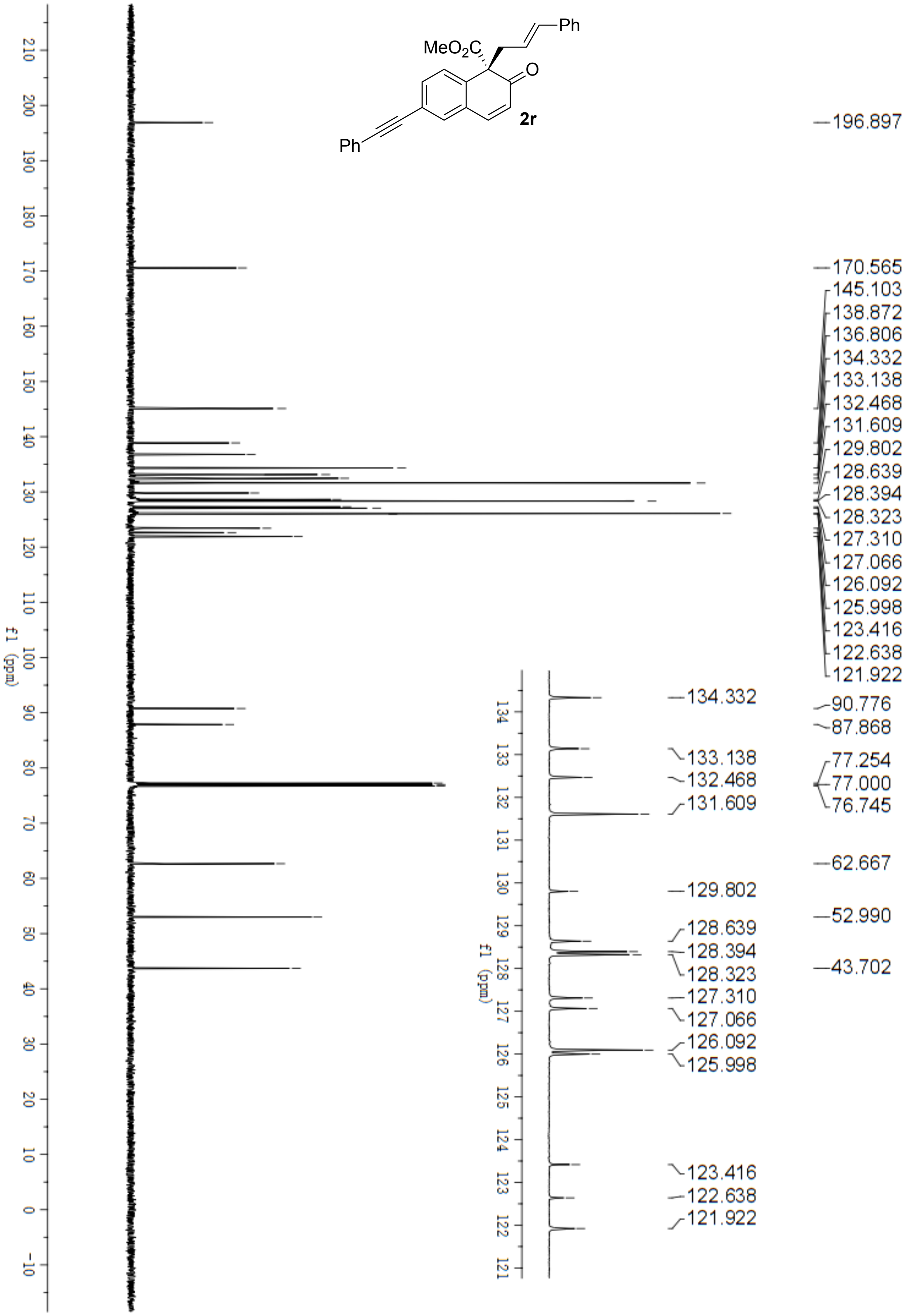
MeO2C
Ph
O
2r
Ph
196.897
170.565
145.103
138.872
136.806
134.332
133.138
132.468
131.609
129.802
128.639
128.394
128.323
127.310
127.066
126.092
125.998
123.416
122.638
121.922
90.776
87.868
77.254
77.000
76.745
62.667
52.990
43.702
f1 (ppm)

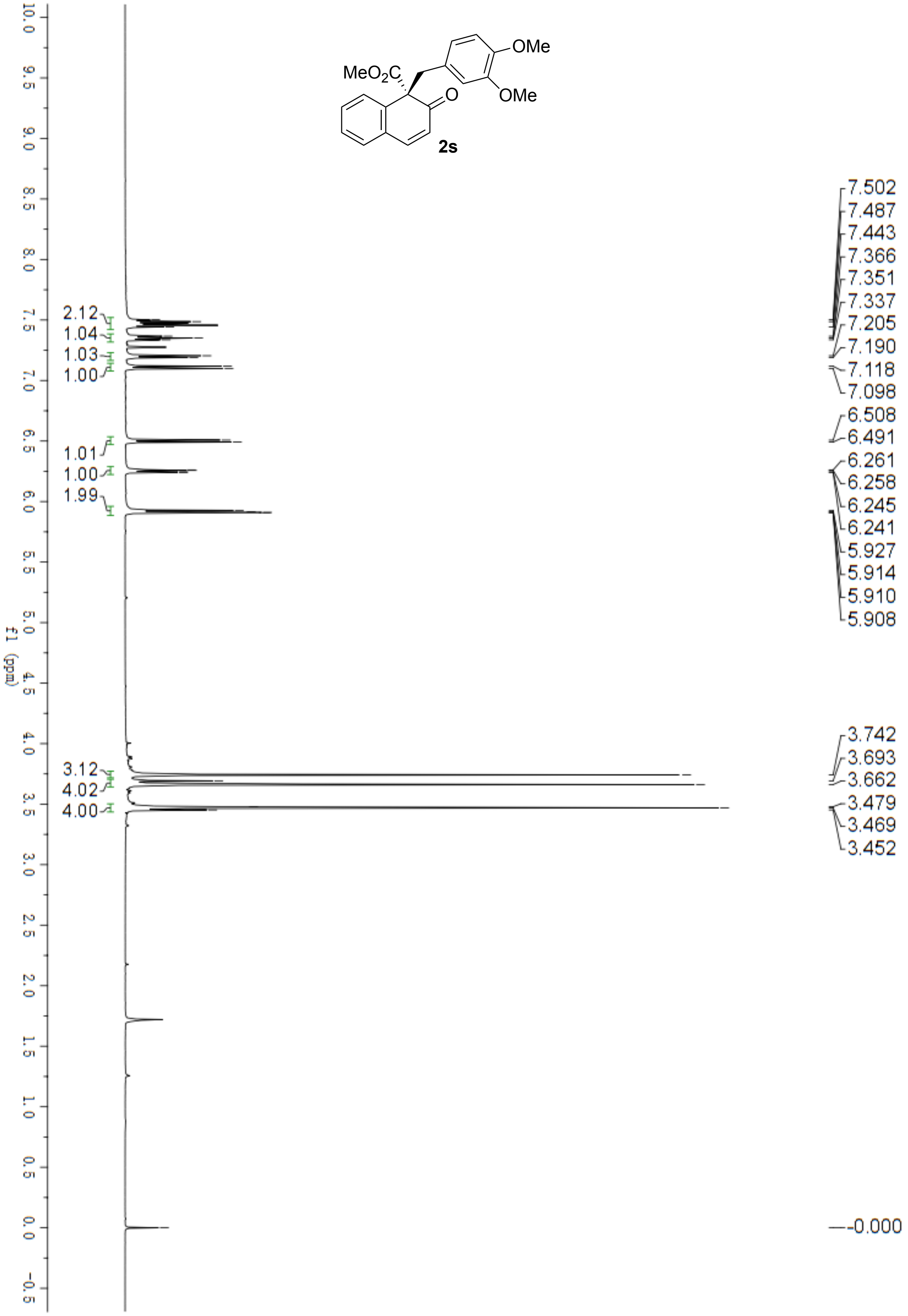
MeO2C
OMe
O
OMe
2s
7.502
7.487
7.443
7.366
7.351
7.337
7.205
7.190
7.118
7.098
6.508
6.491
6.261
6.258
6.245
6.241
5.927
5.914
5.910
5.908
3.742
3.693
3.662
3.479
3.469
3.452
-0.000
2.12
1.04
1.03
1.00
1.01
1.00
1.99
3.12
4.02
4.00
f1 (ppm)

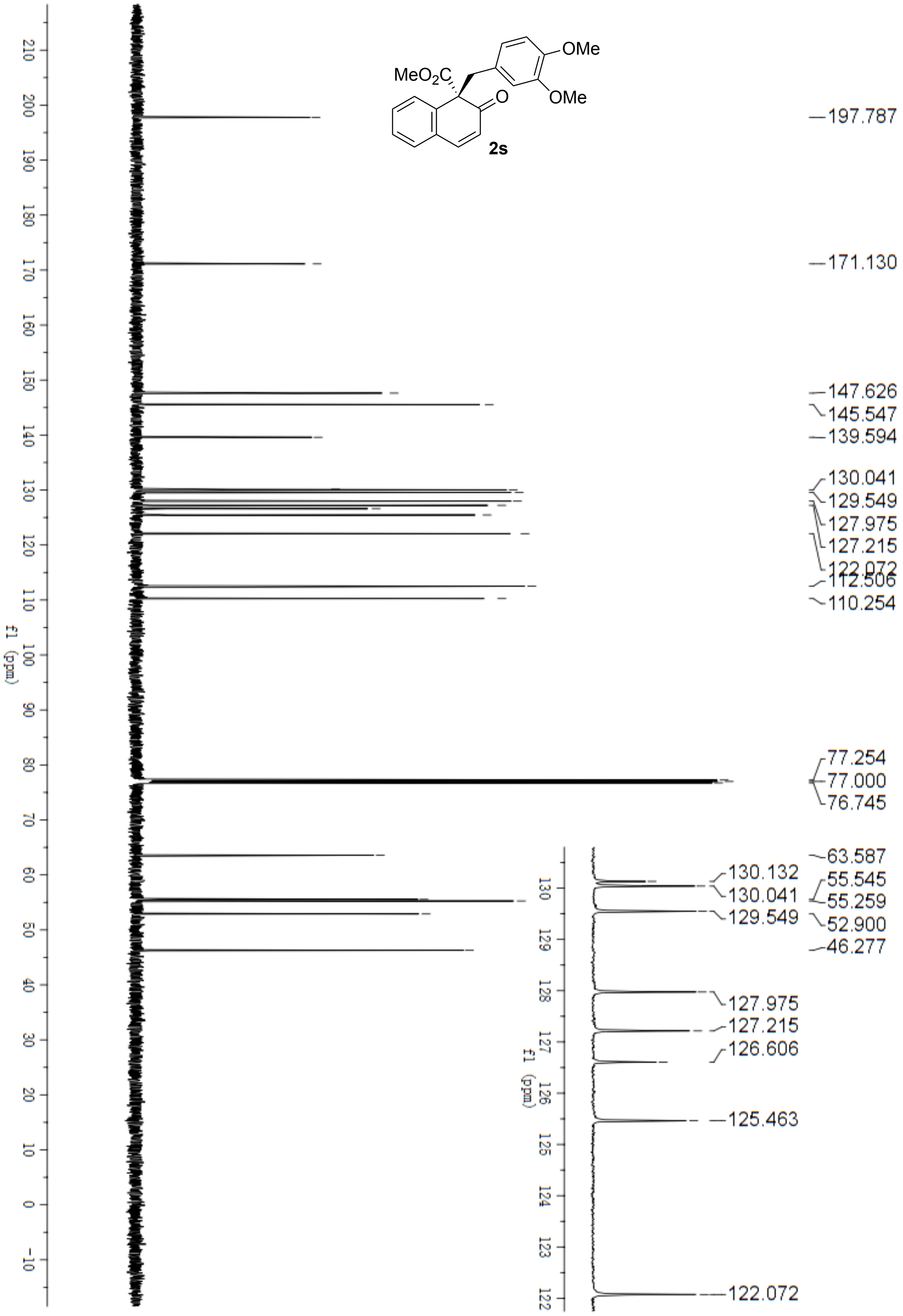
MeO2C
OMe
OMe
O
2s
197.787
171.130
147.626
145.547
139.594
130.041
129.549
127.975
127.215
122.072
112.506
110.254
77.254
77.000
76.745
63.587
55.545
55.259
52.900
46.277
130.132
130.041
129.549
127.975
127.215
126.606
125.463
122.072
f1 (ppm)

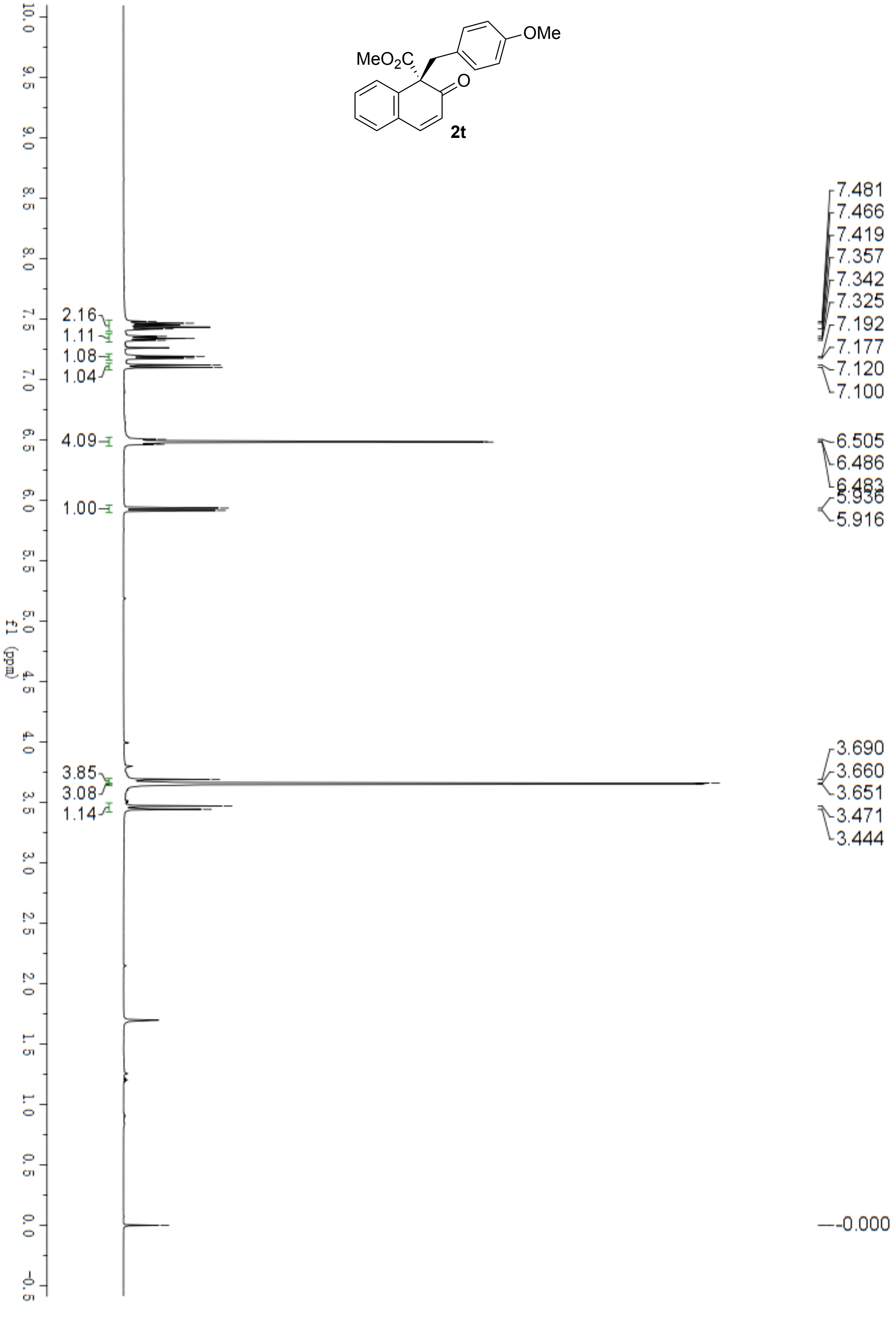
MeO2C
OMe
O
2t
7.481
7.466
7.419
7.357
7.342
7.325
7.192
7.177
7.120
7.100
6.505
6.486
6.483
5.936
5.916
3.690
3.660
3.651
3.471
3.444
-0.000
2.16
1.11
1.08
1.04
4.09
1.00
3.85
3.08
1.14
f1 (ppm)

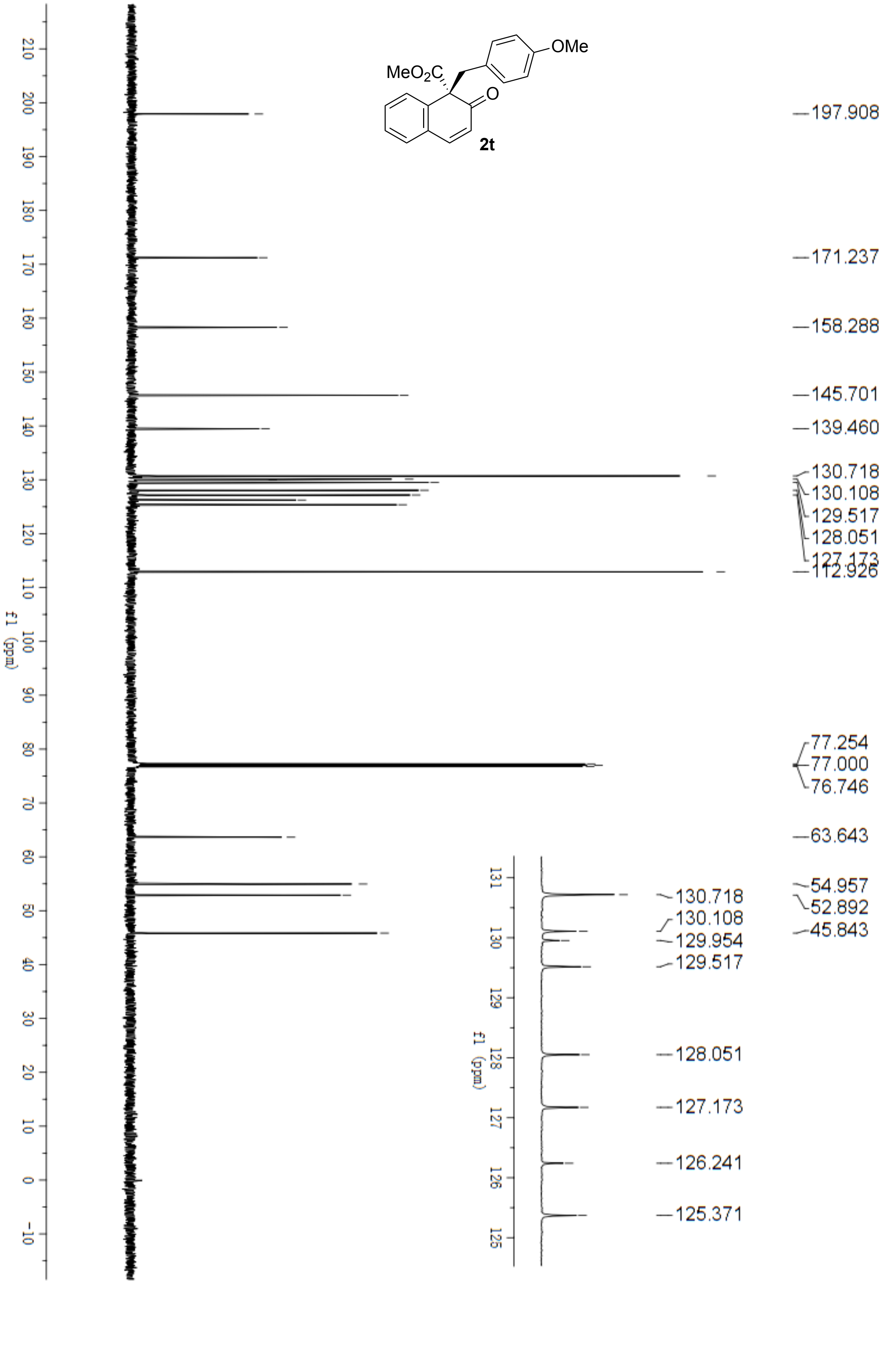
MeO2C
OMe
O
2t
197.908
171.237
158.288
145.701
139.460
130.718
130.108
129.517
128.051
127.173
112.926
77.254
77.000
76.746
63.643
54.957
52.892
45.843
130.718
130.108
129.954
129.517
128.051
127.173
126.241
125.371
f1 (ppm)

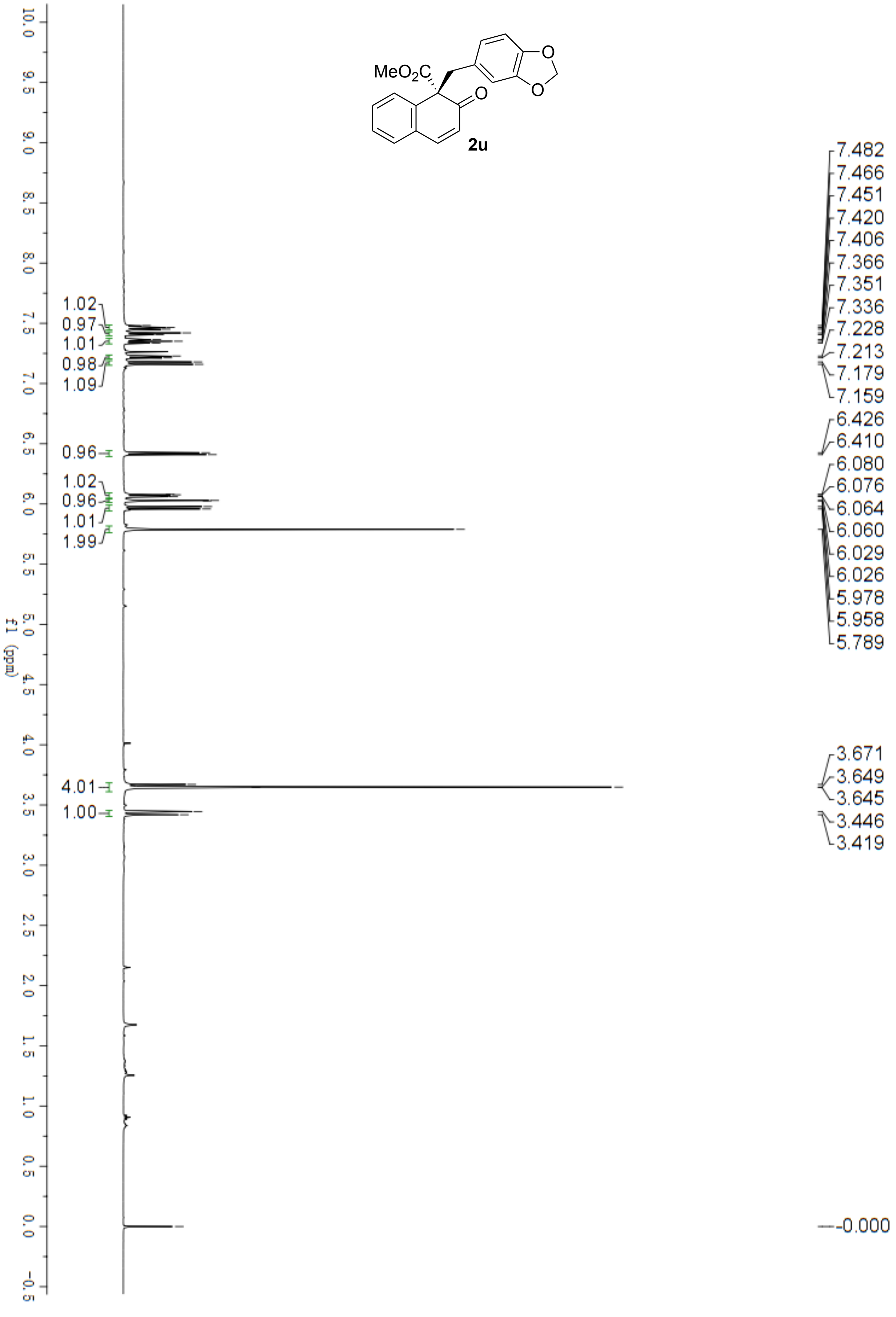
MeO2C
O
O
O
2u
7.482
7.466
7.451
7.420
7.406
7.366
7.351
7.336
7.228
7.213
7.179
7.159
6.426
6.410
6.080
6.076
6.064
6.060
6.029
6.026
5.978
5.958
5.789
3.671
3.649
3.645
3.446
3.419
-0.000
1.02
0.97
1.01
0.98
1.09
0.96
1.02
0.96
1.01
1.99
4.01
1.00
f1 (ppm)

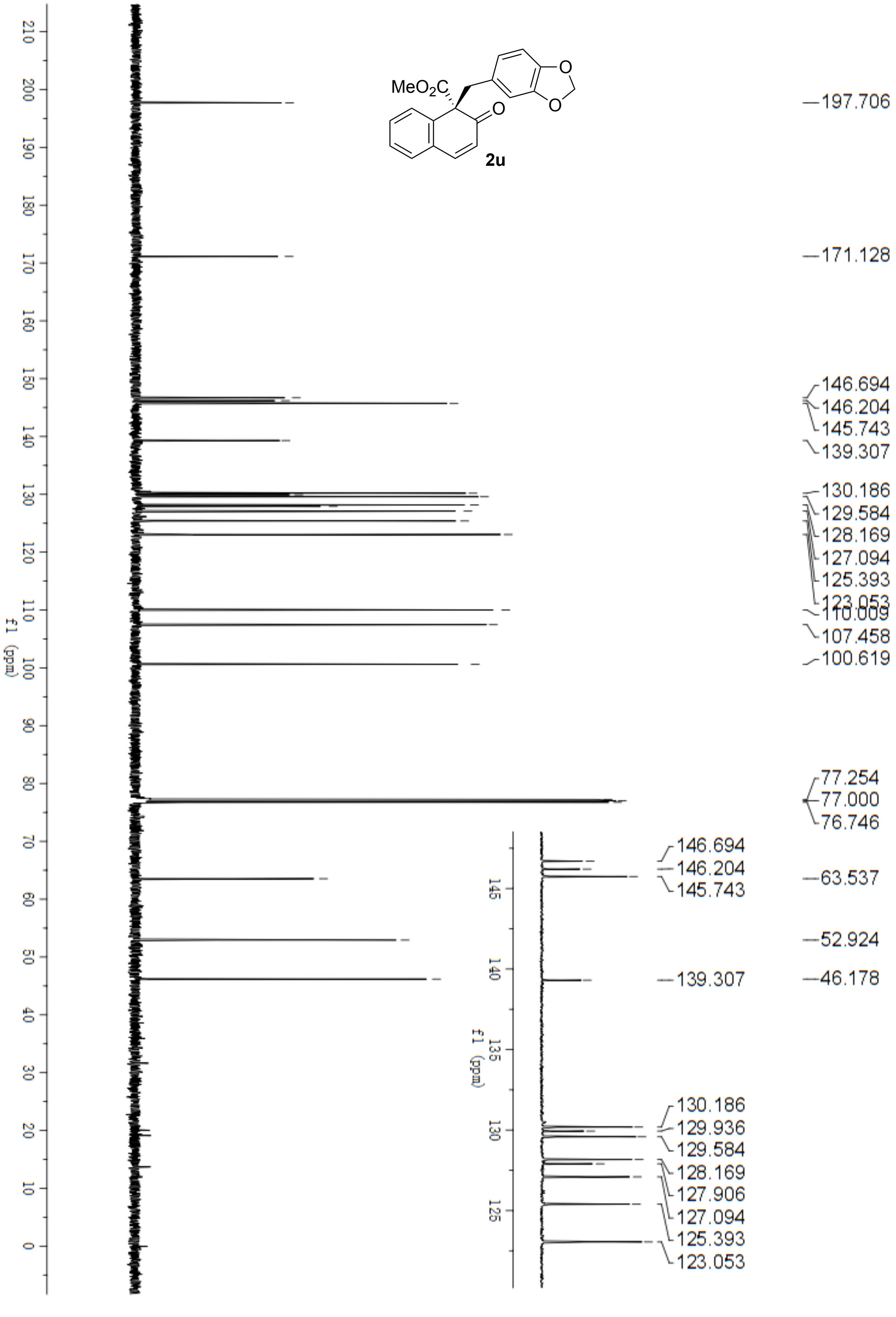
MeO2C
O
2u
197.706
171.128
146.694
146.204
145.743
139.307
130.186
129.584
128.169
127.094
125.393
123.053
110.009
107.458
100.619
77.254
77.000
76.746
63.537
52.924
46.178
146.694
146.204
145.743
139.307
130.186
129.936
129.584
128.169
127.906
127.094
125.393
123.053
f1 (ppm)

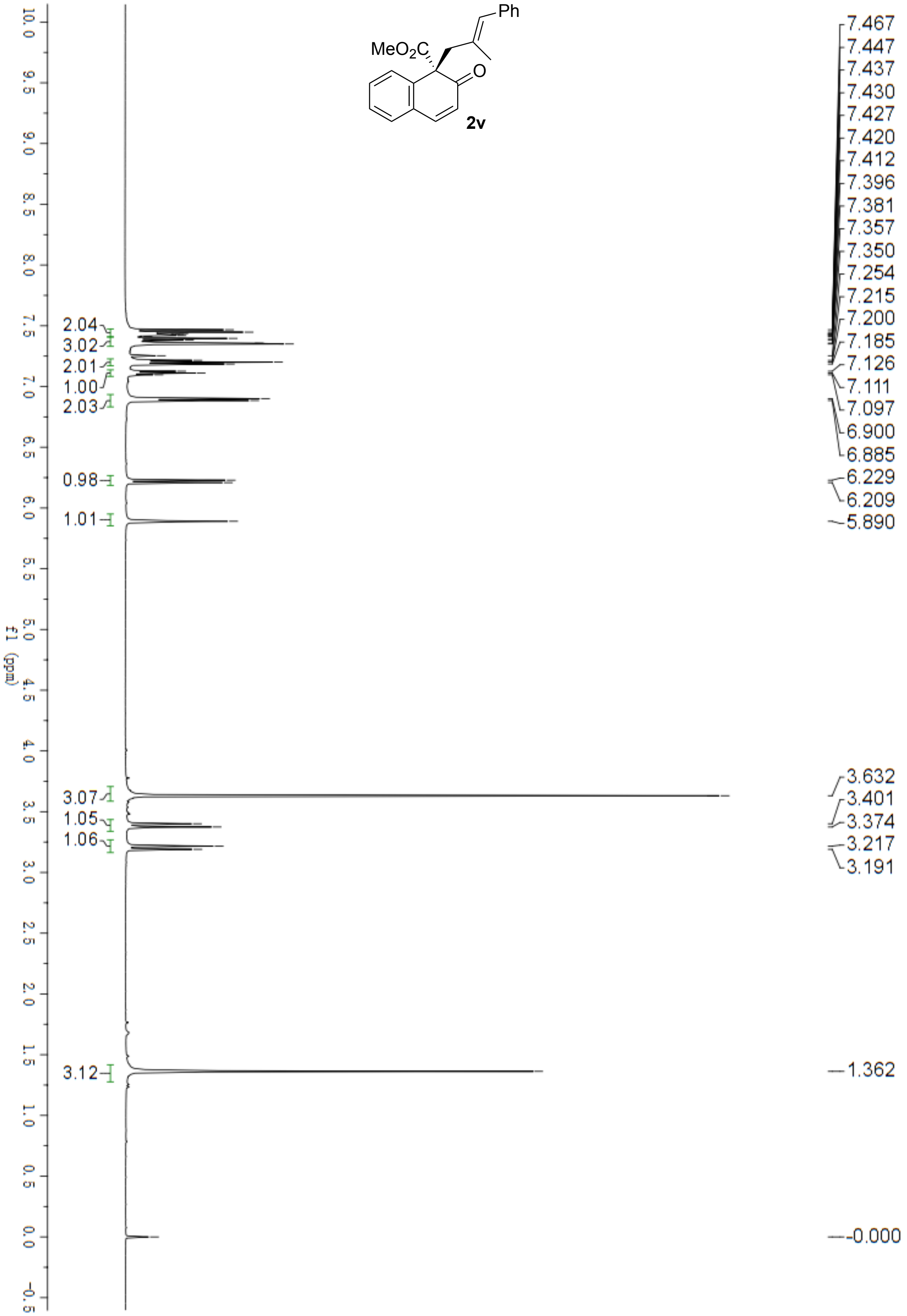
Ph
MeO2C
O
2v
7.467
7.447
7.437
7.430
7.427
7.420
7.412
7.396
7.381
7.357
7.350
7.254
7.215
7.200
7.185
7.126
7.111
7.097
6.900
6.885
6.229
6.209
5.890
3.632
3.401
3.374
3.217
3.191
1.362
-0.000
2.04
3.02
2.01
1.00
2.03
0.98
1.01
3.07
1.05
1.06
3.12
f1 (ppm)
10.0
9.5
9.0
8.5
8.0
7.5
7.0
6.5
6.0
5.5
5.0
4.5
4.0
3.5
3.0
2.5
2.0
1.5
1.0
0.5
0.0
-0.5

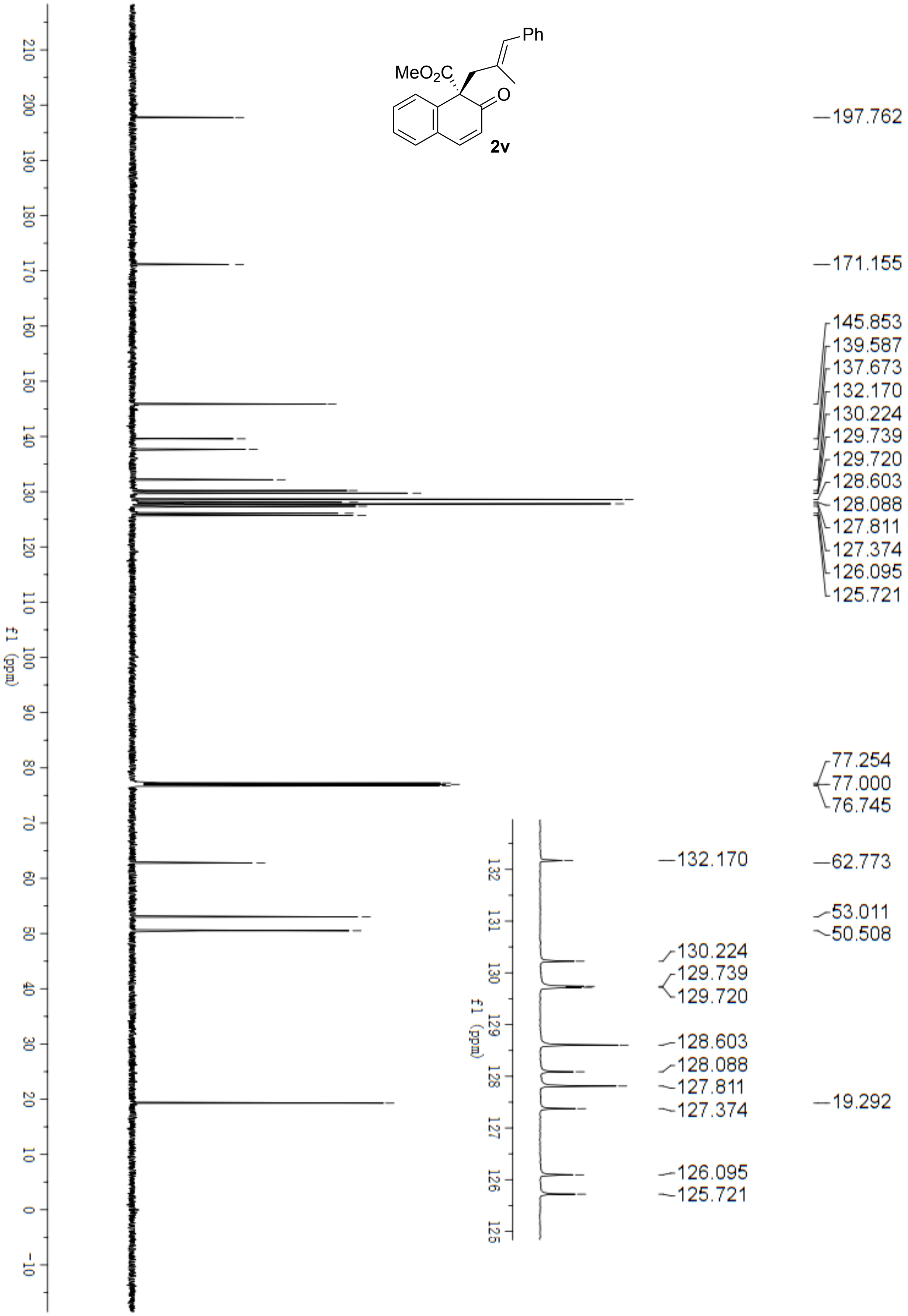
MeO2C
Ph
O
2v
197.762
171.155
145.853
139.587
137.673
132.170
130.224
129.739
129.720
128.603
128.088
127.811
127.374
126.095
125.721
77.254
77.000
76.745
62.773
53.011
50.508
19.292
f1 (ppm)

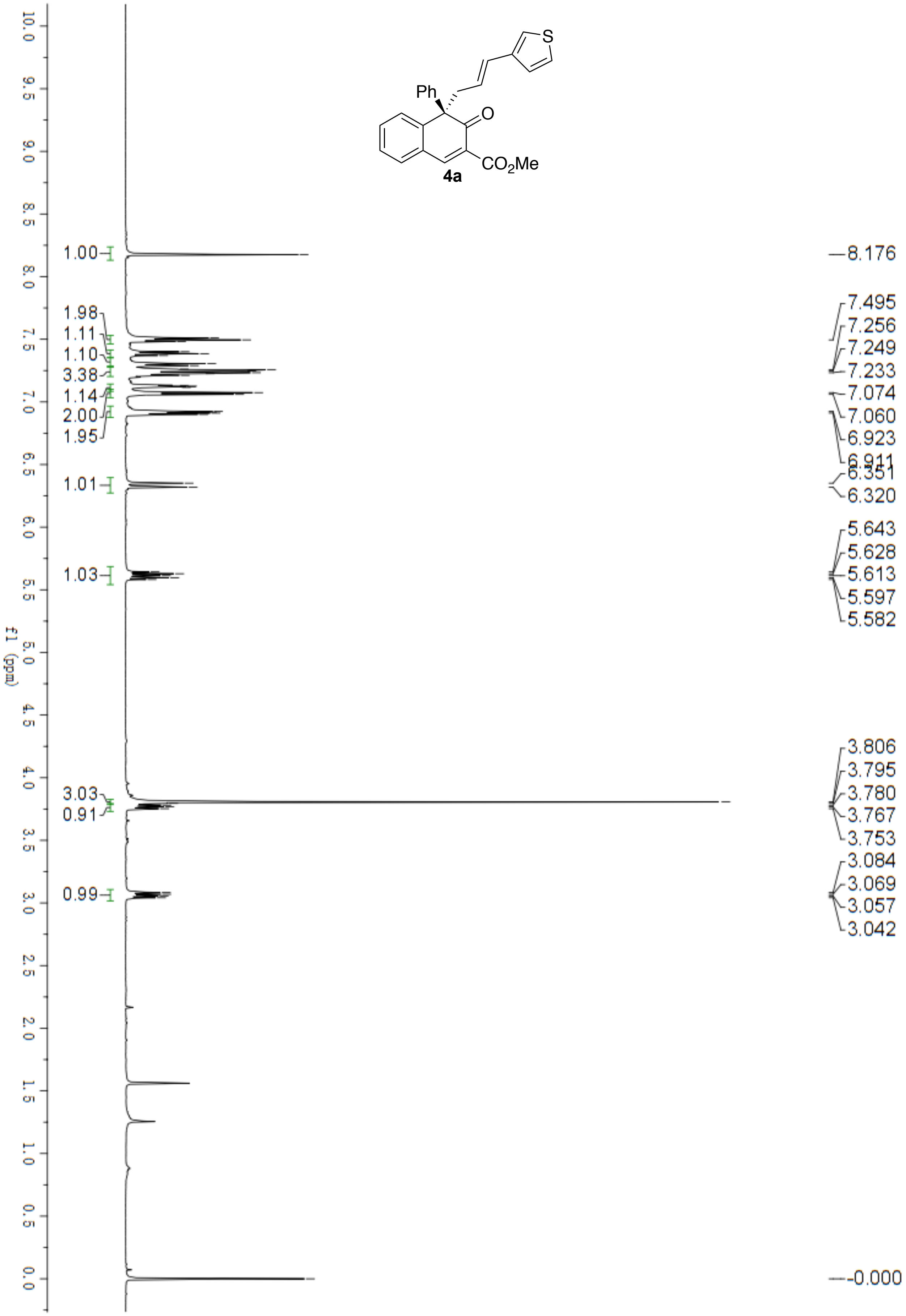
Ph
O
S
CO2Me
4a
f1 (ppm)
1.00
1.98
1.11
1.10
3.38
1.14
2.00
1.95
1.01
1.03
3.03
0.91
0.99
8.176
7.495
7.256
7.249
7.233
7.074
7.060
6.923
6.911
6.351
6.320
5.643
5.628
5.613
5.597
5.582
3.806
3.795
3.780
3.767
3.753
3.084
3.069
3.057
3.042
-0.000

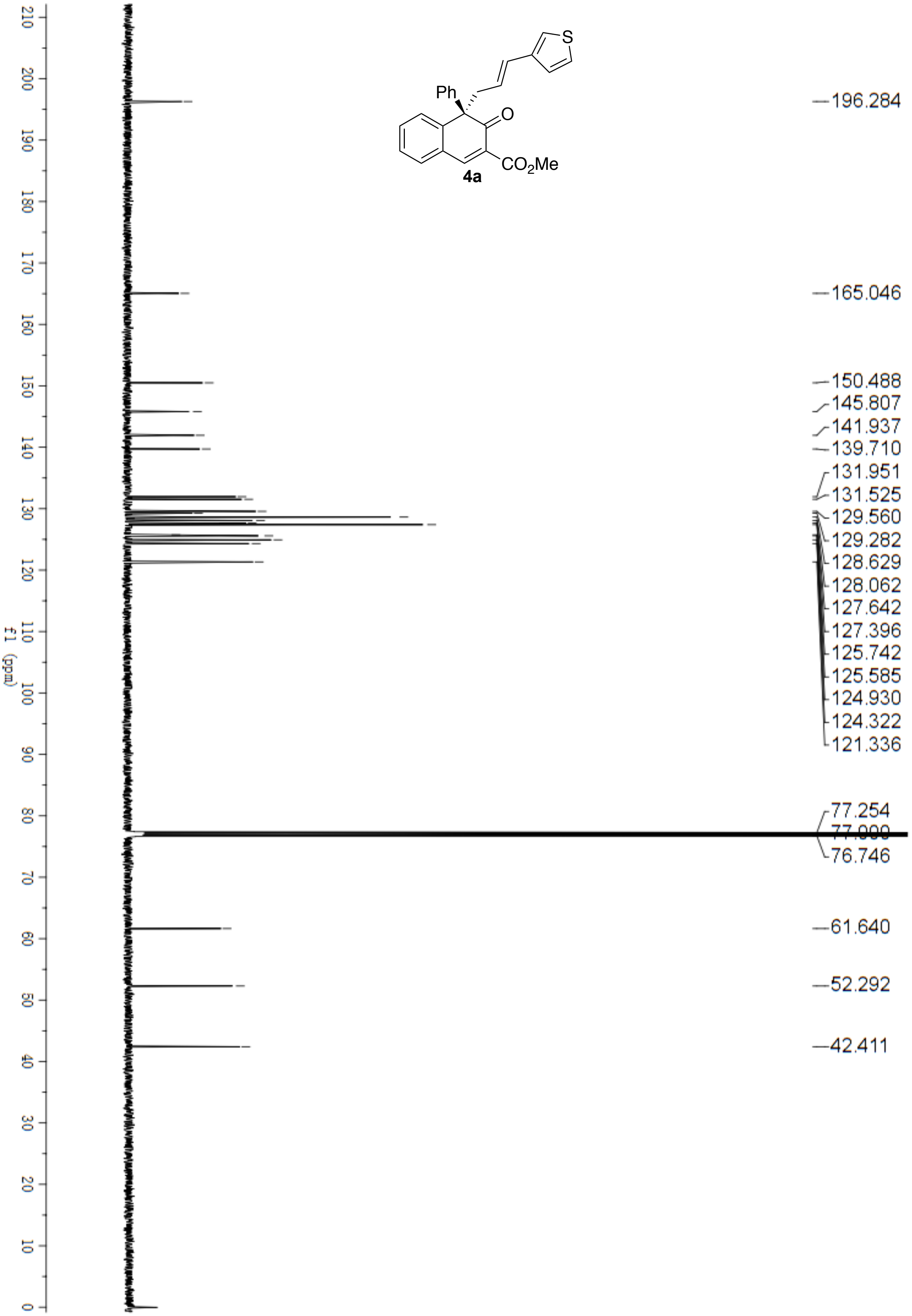
Ph
S
O
CO2Me
4a
196.284
165.046
150.488
145.807
141.937
139.710
131.951
131.525
129.560
129.282
128.629
128.062
127.642
127.396
125.742
125.585
124.930
124.322
121.336
77.254
77.000
76.746
61.640
52.292
42.411
f1 (ppm)
210
200
190
180
170
160
150
140
130
120
110
100
90
80
70
60
50
40
30
20
10
0

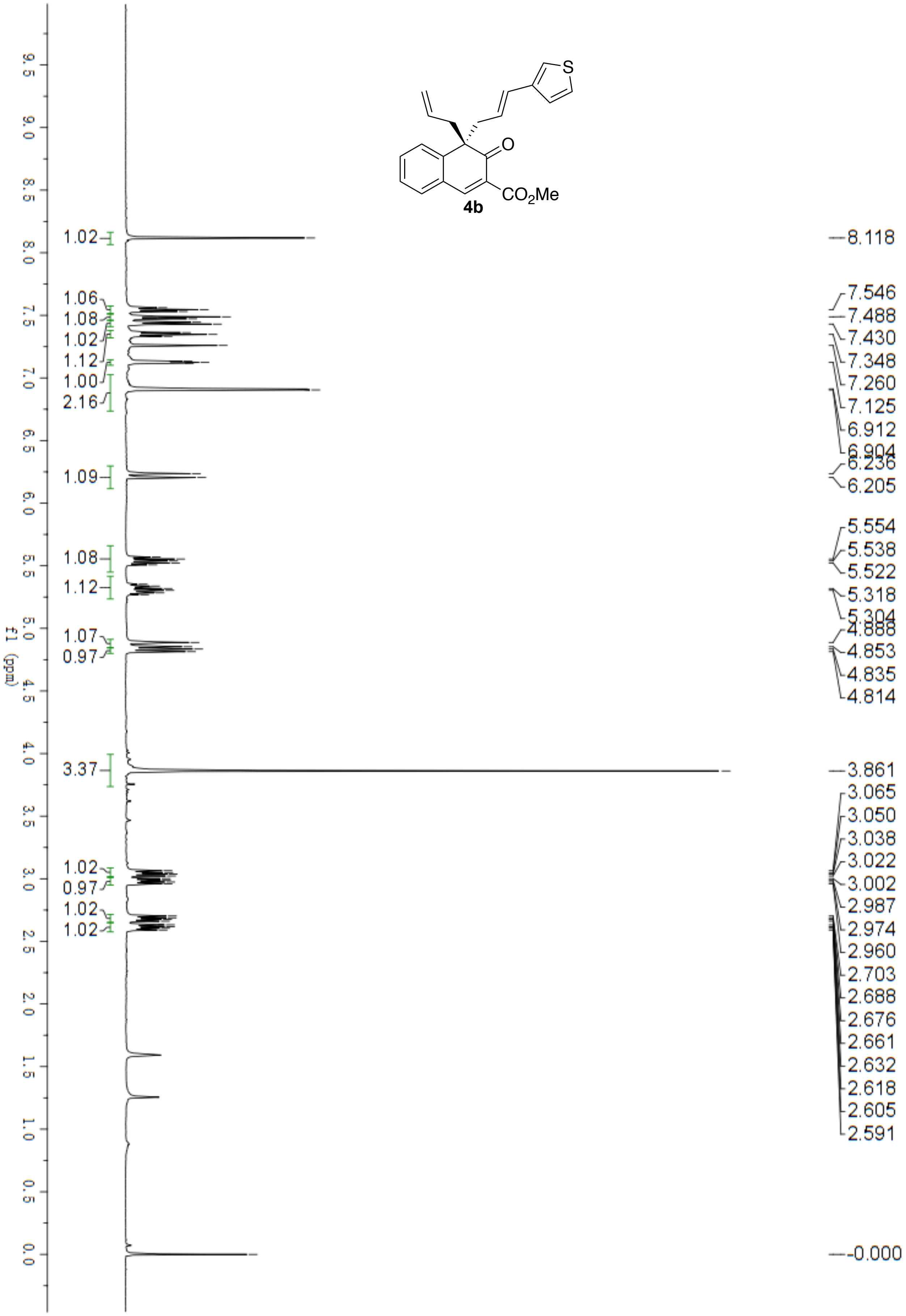
S
O
CO2Me
4b
f1 (ppm)
9.5
9.0
8.5
8.0
7.5
7.0
6.5
6.0
5.5
5.0
4.5
4.0
3.5
3.0
2.5
2.0
1.5
1.0
0.5
0.0
1.02
1.06
1.08
1.02
1.12
1.00
2.16
1.09
1.08
1.12
1.07
0.97
3.37
1.02
0.97
1.02
1.02
8.118
7.546
7.488
7.430
7.348
7.260
7.125
6.912
6.904
6.236
6.205
5.554
5.538
5.522
5.318
5.304
4.888
4.853
4.835
4.814
3.861
3.065
3.050
3.038
3.022
3.002
2.987
2.974
2.960
2.703
2.688
2.676
2.661
2.632
2.618
2.605
2.591
-0.000

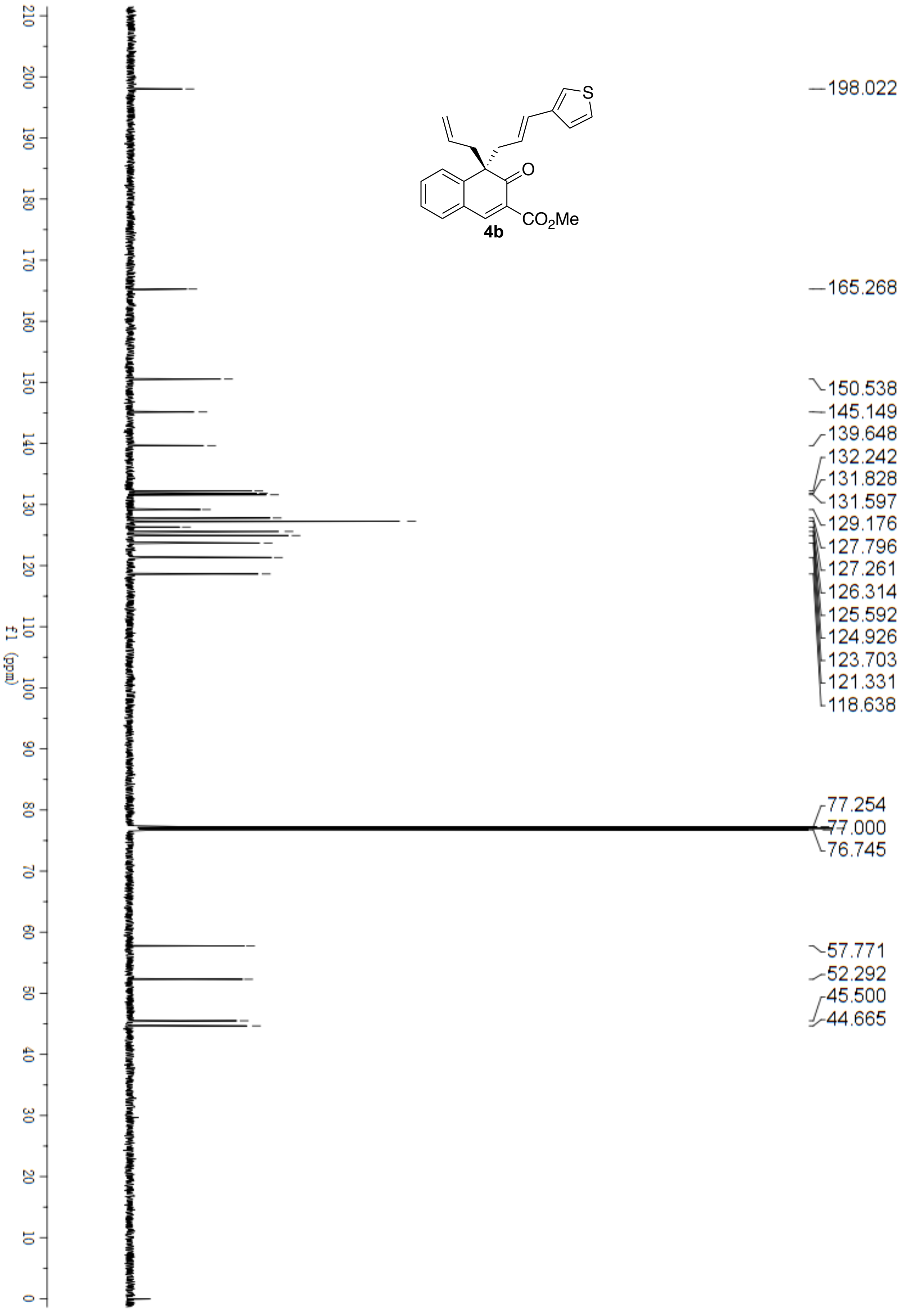
4b
CO2Me
O
S
198.022
165.268
150.538
145.149
139.648
132.242
131.828
131.597
129.176
127.796
127.261
126.314
125.592
124.926
123.703
121.331
118.638
77.254
77.000
76.745
57.771
52.292
45.500
44.665
f1 (ppm)
210
200
190
180
170
160
150
140
130
120
110
100
90
80
70
60
50
40
30
20
10
0

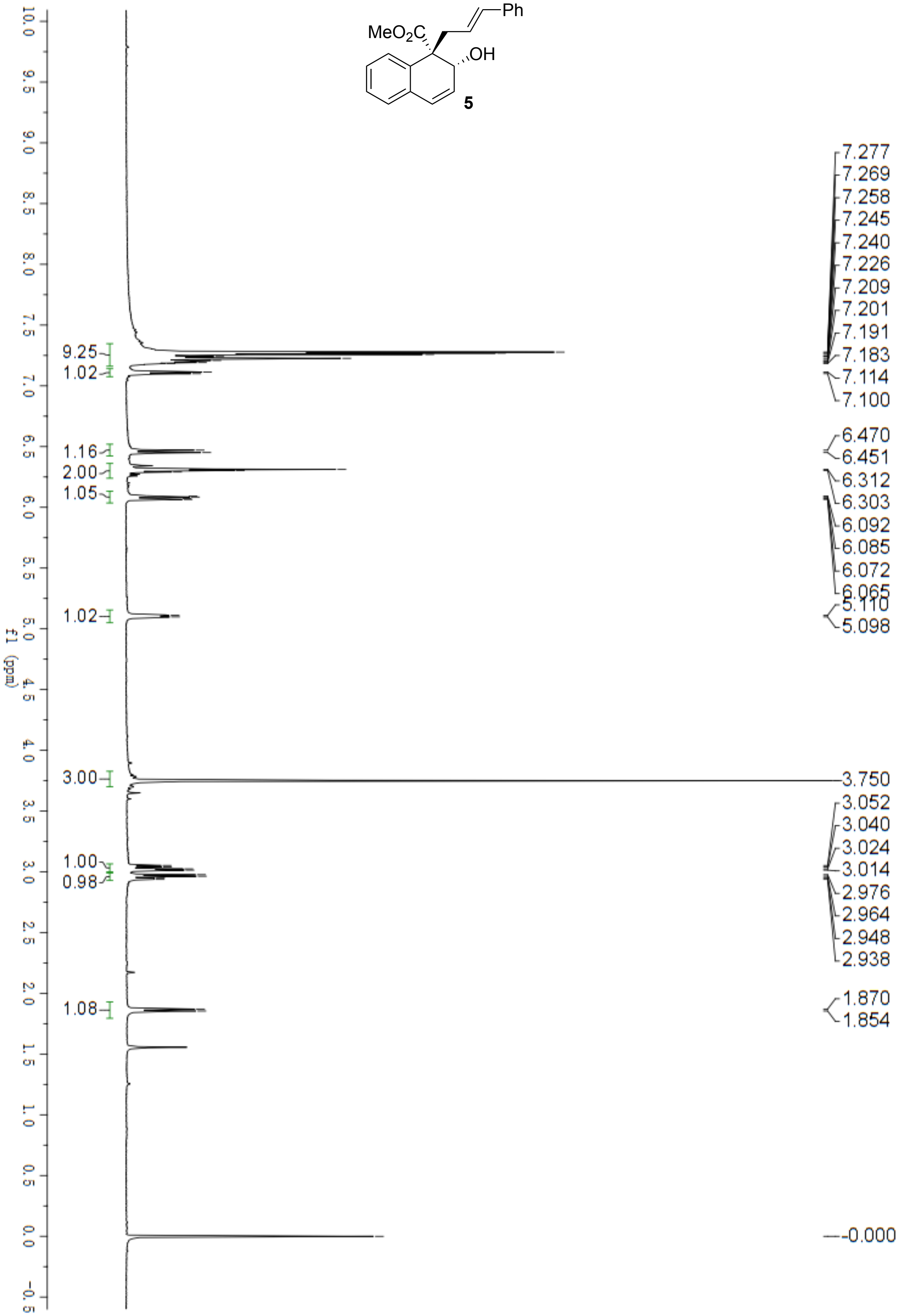
MeO2C
OH
Ph
5
7.277
7.269
7.258
7.245
7.240
7.226
7.209
7.201
7.191
7.183
7.114
7.100
6.470
6.451
6.312
6.303
6.092
6.085
6.072
6.065
5.110
5.098
3.750
3.052
3.040
3.024
3.014
2.976
2.964
2.948
2.938
1.870
1.854
-0.000
9.25
1.02
1.16
2.00
1.05
1.02
3.00
1.00
0.98
1.08
f1 (ppm)

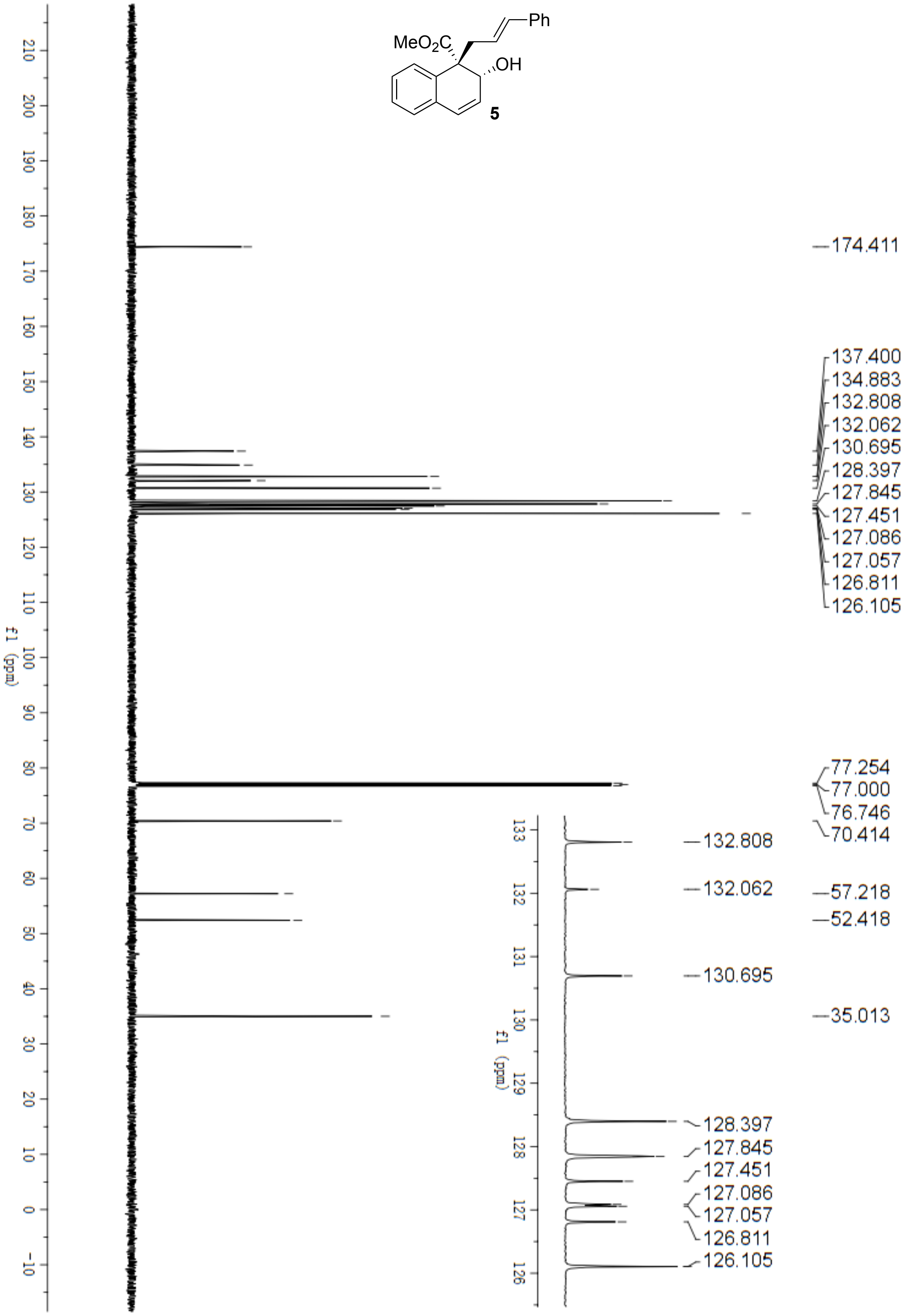
MeO2C
OH
Ph
5
174.411
137.400
134.883
132.808
132.062
130.695
128.397
127.845
127.451
127.086
127.057
126.811
126.105
77.254
77.000
76.746
70.414
57.218
52.418
35.013
f1 (ppm)

H-H COSY

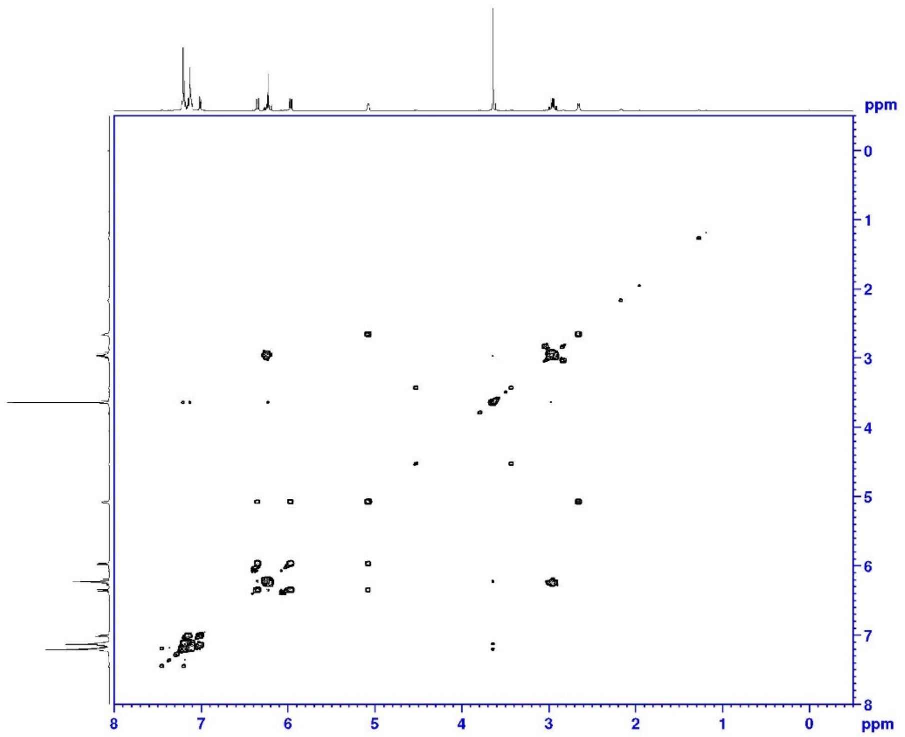
ppm
0
1
2
3
4
5
6
7
8
8 7 6 5 4 3 2 1 0
ppm

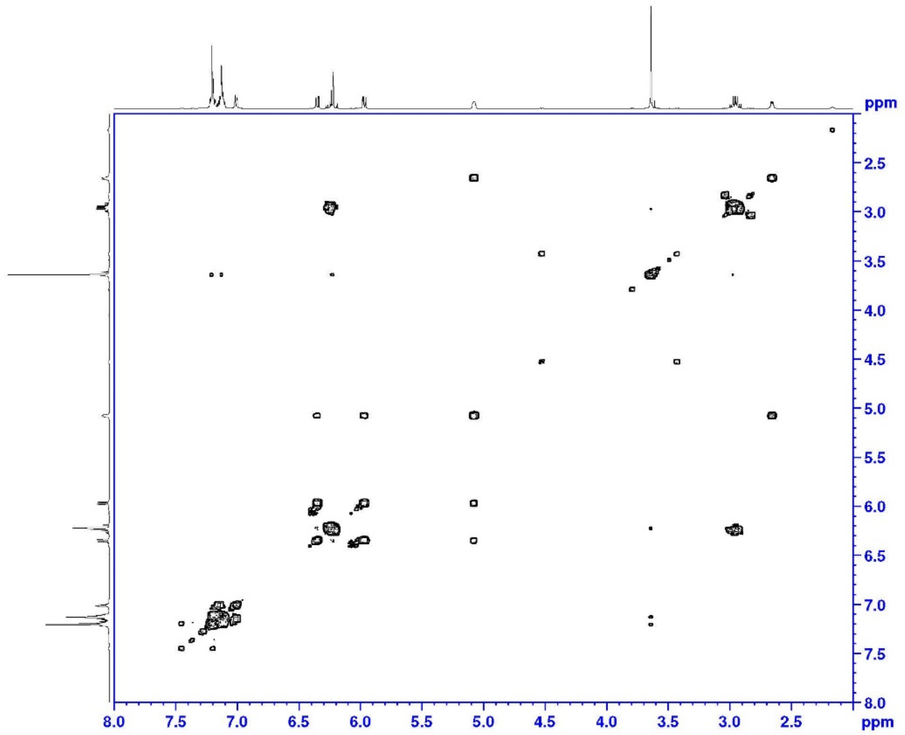
ppm
2.5
3.0
3.5
4.0
4.5
5.0
5.5
6.0
6.5
7.0
7.5
8.0
8.0 7.5 7.0 6.5 6.0 5.5 5.0 4.5 4.0 3.5 3.0 2.5
ppm

H-H NOESY

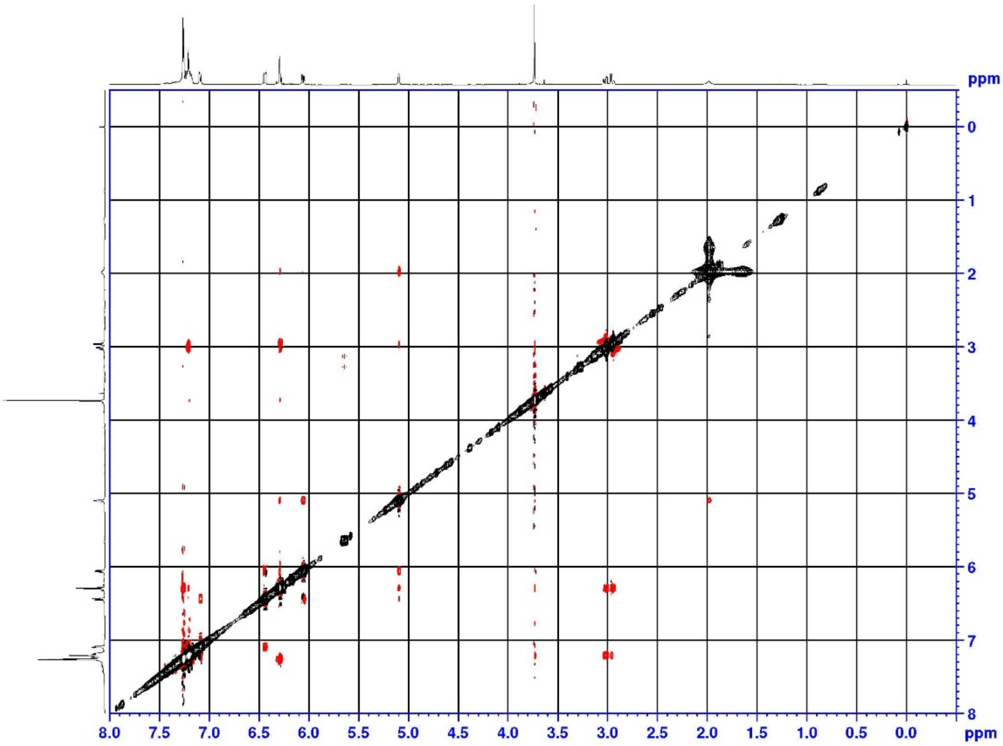
ppm
0
1
2
3
4
5
6
7
8
8.0 7.5 7.0 6.5 6.0 5.5 5.0 4.5 4.0 3.5 3.0 2.5 2.0 1.5 1.0 0.5 0.0
ppm

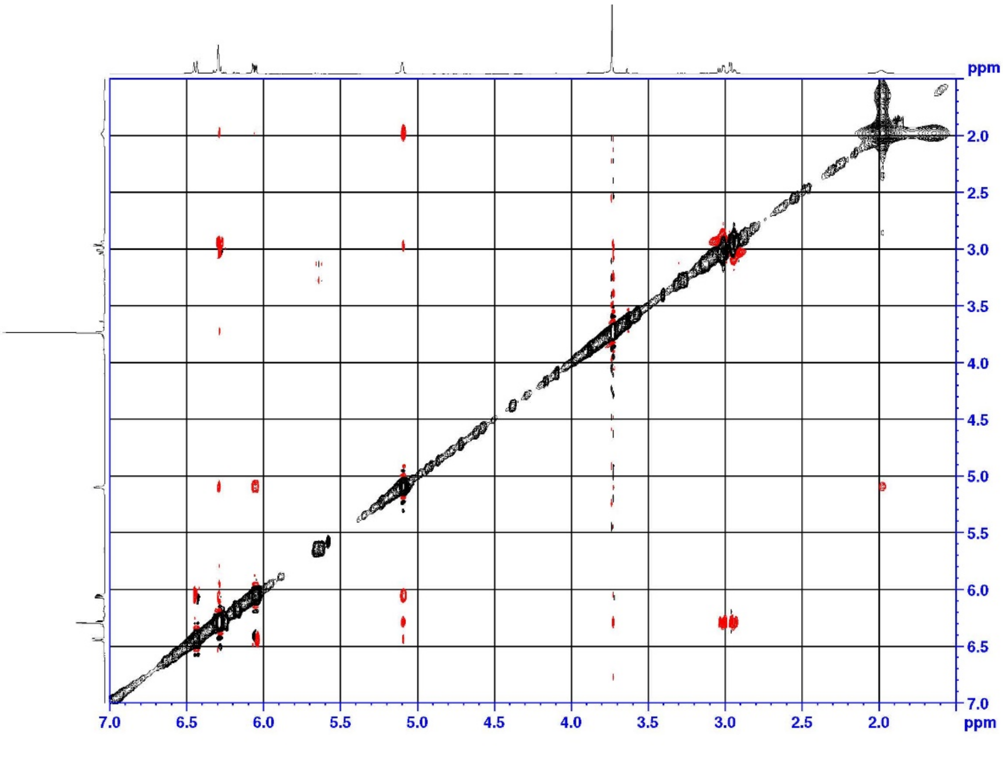
ppm
2.0
2.5
3.0
3.5
4.0
4.5
5.0
5.5
6.0
6.5
7.0
7.0 6.5 6.0 5.5 5.0 4.5 4.0 3.5 3.0 2.5 2.0
ppm

11. HPLC Chromatograms

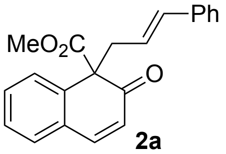

2a

=== Shimadzu LabSolutions ===

Sample :
ID :
Data Name :LY-5-3a-rac.lcd
Method Name :C4-20-80-1-15min.lcm
Batch Nam :LY-5-3a.lcb
Vial :1-20 サンプルタイプ :Unknown
Inj.Volume :1 uL
Acquisition Date :2017/10/05 16:23:52 分析者 :System Administrator
Modified Date :2017/10/05 16:58:21 解析者 :System Administrator

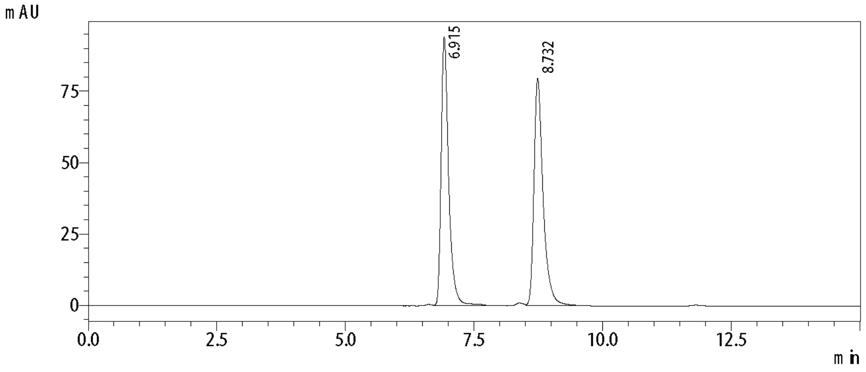

PDA Ch1 254nm

Peak No.	RT (min)	Area	Height	% Area
1	6.915	962268	94320	49.519
2	8.732	980963	79701	50.481
Total		1943230	174021	100.000

UVスペクトル

保持時間 :6.915 min

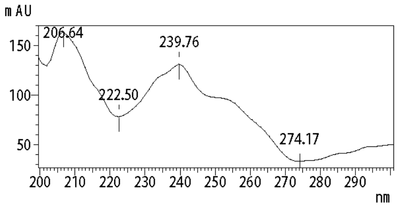

保持時間 :8.732 min

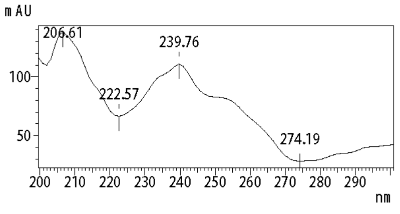

D:¥Data¥LY¥data¥LY-5-3a-rac.lcd

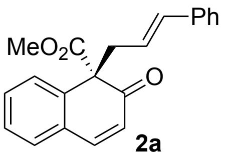

2a

=== Shimadzu LabSolutions ===

Sample :
ID :
Data Name :LY-6-5%cat.lcd
Method Name :安定化C4-20-80-1-20min.lcm
Batch Nam :LY-6-5%cat.lcb
Vial :1-21 サンプルタイプ :Unknown
Inj.Volume :1 uL
Acquisition Date :2018/05/28 10:13:55 分析者 :System Administrator
Modified Date :2018/05/28 10:33:58 解析者 :System Administrator

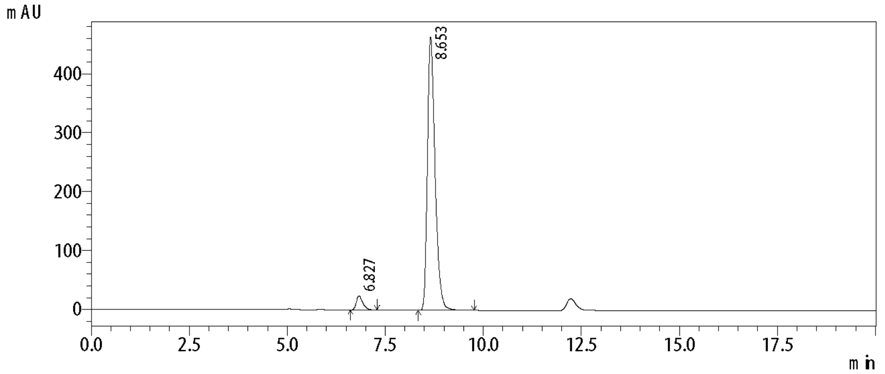

PDA Ch1 254nm

Peak No.	RT (min)	Area	Height	% Area
1	6.827	265476	23727	4.065
2	8.653	6265030	464021	95.935
Total		6530506	487749	100.000

UVスペクトル

保持時間 :6.827 min

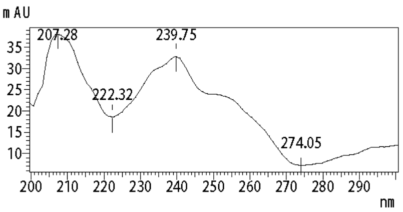

保持時間 :8.653 min

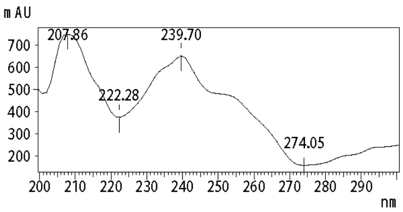

G:¥LY-6-5%cat.lcd

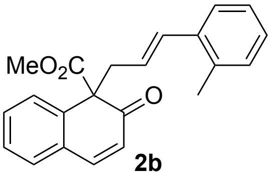

2b

=== Shimadzu LabSolutions ===

Sample :
ID :
Data Name :LY-5-3b-rac.lcd
Method Name :C4-20-80-1-15min.lcm
Batch Nam :LY-5-3b.lcb
Vial :1-20 サンプルタイプ :Unknown
Inj. Volume :1 uL
Acquisition Date :2017/10/04 19:36:20 分析者 :System Administrator
Modified Date :2017/10/04 20:09:52 解析者 :System Administrator

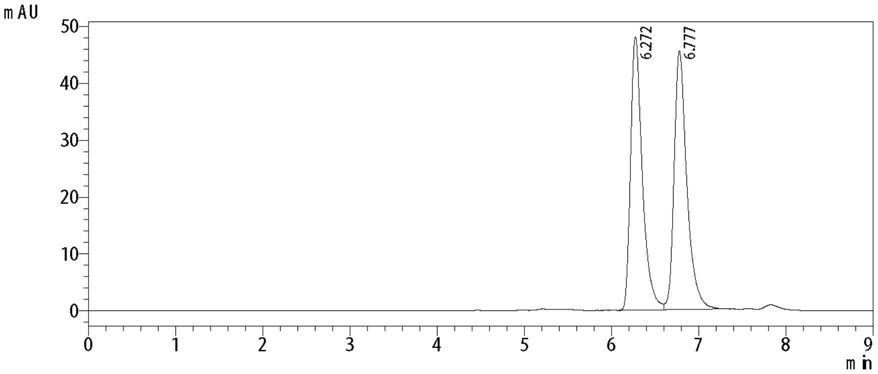

PDA Ch1 272nm

Peak No.	RT (min)	Area	Height	% Area
1	6.272	450135	48008	49.534
2	6.777	458608	45530	50.466
Total		908743	93538	100.000

UVスペクトル

保持時間 :6.272 min

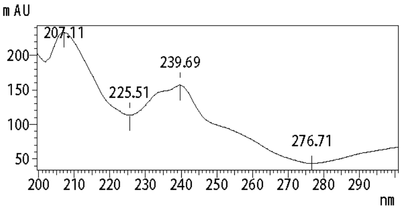

保持時間 :6.777 min

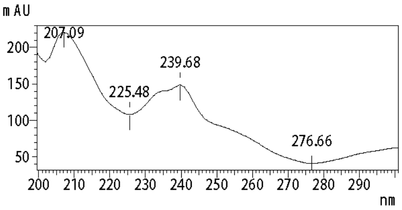

D:¥Data¥LY¥data¥LY-5-3b-rac.lcd

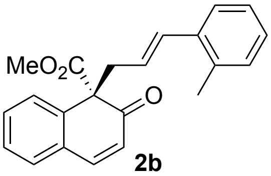

2b

=== Shimadzu LabSolutions ===

Sample :
ID :
Data Name :LY-5-3b.lcd
Method Name :C4-20-80-1-15min.lcm
Batch Nam :LY-5-3b.lcb
Vial :1-21 サンプルタイプ :Unknown
Inj. Volume :1 uL
Acquisition Date :2017/10/04 19:51:47 分析者 :System Administrator
Modified Date :2017/11/02 11:19:40 解析者 :System Administrator

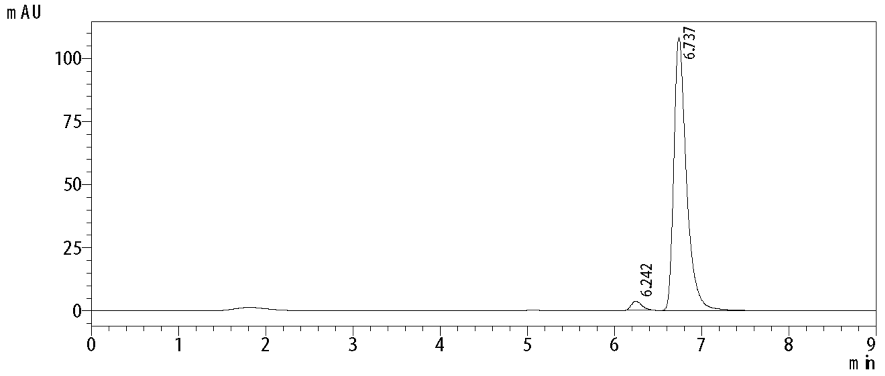

PDA Ch1 272nm

Peak No.	RT (min)	Area	Height	% Area
1	6.242	30269	3579	2.684
2	6.737	1097493	108603	97.316
Total		1127762	112182	100.000

UVスペクトル

保持時間 :6.242 min

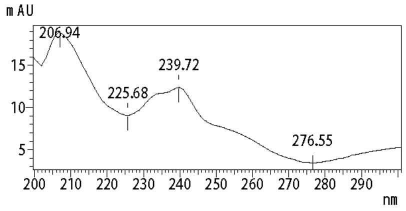

保持時間 :6.737 min

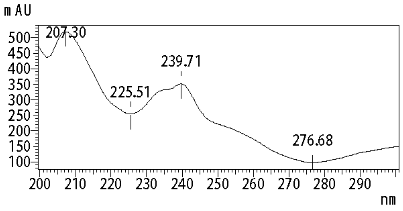

D:¥Data¥LY¥data¥LY-5-3b.lcd

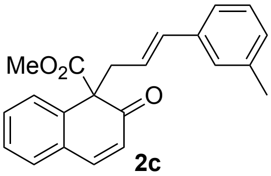

2c

=== Shimadzu LabSolutions ===

Sample :
ID :
Data Name :LY-5-3c-rac.lcd
Method Name :C4-20-80-1-15min.lcm
Batch Nam :LY-5-3c.lcb
Vial :1-18 サンプルタイプ :Unknown
Inj. Volume :1 uL
Acquisition Date :2017/10/04 18:25:01 分析者 :System Administrator
Modified Date :2017/11/02 11:28:34 解析者 :System Administrator

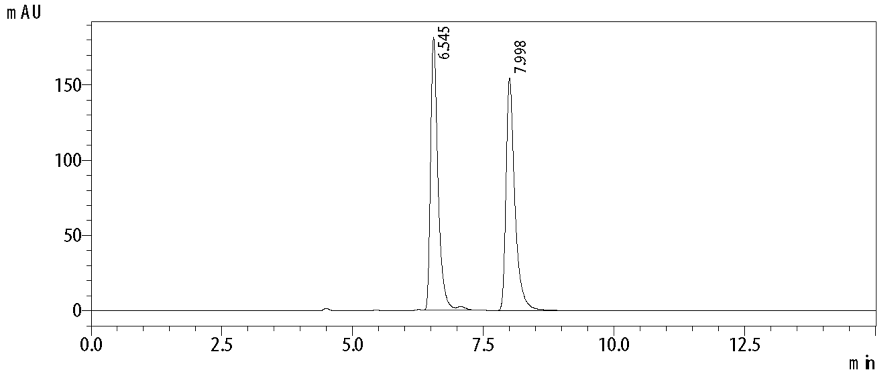

PDA Ch1 254nm

Peak No.	RT (min)	Area	Height	% Area
1	6.545	1794225	181395	49.647
2	7.998	1819723	154470	50.353
Total		3613948	335865	100.000

UVスペクトル

保持時間 :6.545 min

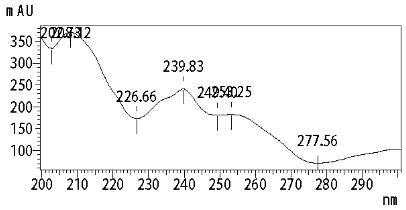

保持時間 :7.998 min

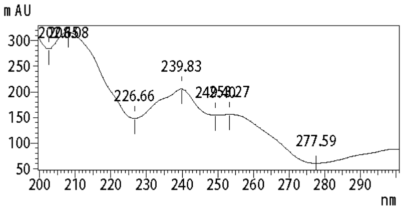

D:¥Data¥LY¥data¥LY-5-3c-rac.lcd

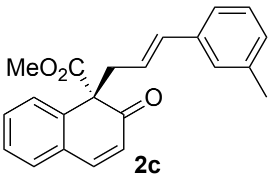

2c

=== Shimadzu LabSolutions ===

Sample :
ID :
Data Name :LY-5-3c.lcd
Method Name :C4-20-80-1-15min.lcm
Batch Nam :LY-5-3c.lcb
Vial :1-19
Inj. Volume :1 uL
Acquisition Date :2017/10/04 18:40:25
Modified Date :2017/10/04 19:27:42
サンプルタイプ :Unknown
分析者 :System Administrator
解析者 :System Administrator

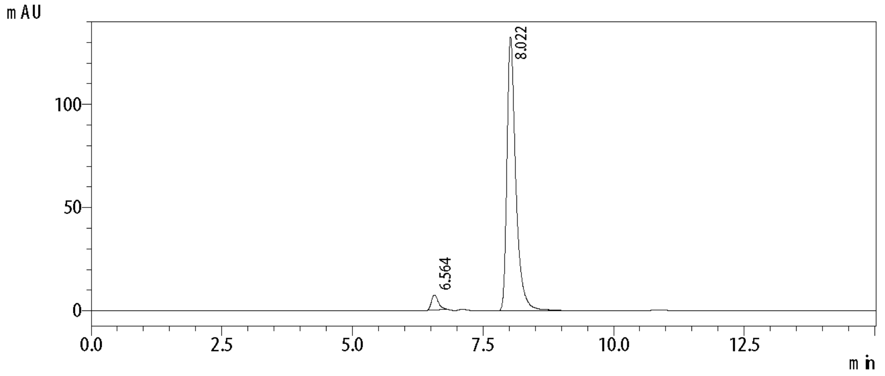

PDA Ch1 254nm

Peak No.	RT (min)	Area	Height	% Area
1	6.564	66673	7256	4.091
2	8.022	1563238	132710	95.909
Total		1629911	139966	100.000

UVスペクトル

保持時間 :6.564 min

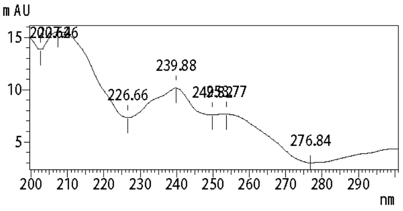

保持時間 :8.022 min

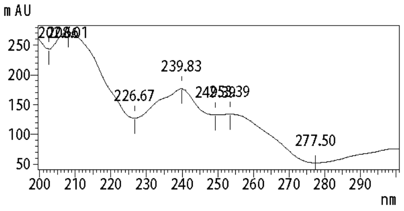

D:¥Data¥LY¥data¥LY-5-3c.lcd

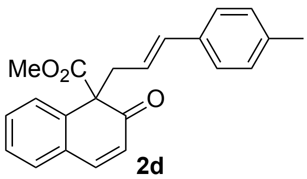

2d

=== Shimadzu LabSolutions ===

Sample	:		
ID	:		
Data Name	:LY-5-3d-rac.lcd		
Method Name	:C4-20-80-1-15min.lcm		
Batch Nam	:LY-5-3d.lcb		
Vial	:1-18	サンプルタイプ	:Unknown
Inj. Volume	:1 uL		
Acquisition Date	:2017/10/03 23:37:15	分析者	:System Administrator
Modified Date	:2017/10/04 10:43:04	解析者	:System Administrator

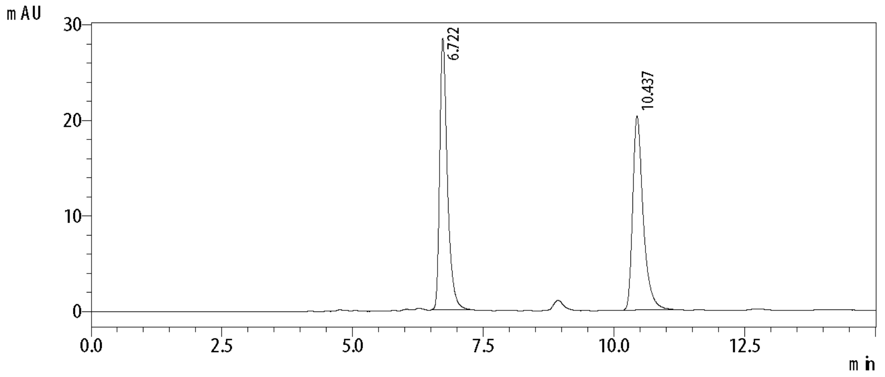

PDA Ch1 254nm

Peak No.	RT (min)	Area	Height	% Area
1	6.722	287877	28421	50.393
2	10.437	283392	20292	49.607
Total		571268	48713	100.000

UVスペクトル

保持時間 :6.722 min

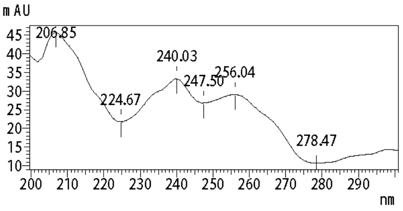

保持時間 :10.437 min

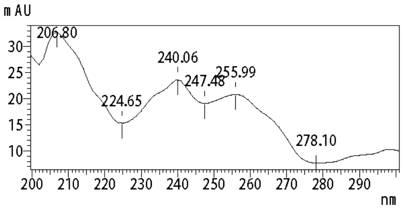

D:¥Data¥LY¥data¥LY-5-3d-rac.lcd

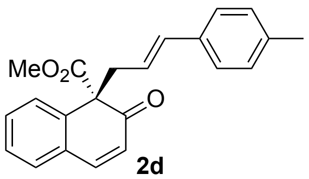

2d

=== Shimadzu LabSolutions ===

Sample	:		
ID	:		
Data Name	:LY-5-3d.lcd		
Method Name	:C4-20-80-1-15min.lcm		
Batch Nam	:LY-5-3d.lcb		
Vial	:1-19	サンプルタイプ	:Unknown
Inj. Volume	:1 uL		
Acquisition Date	:2017/10/03 23:52:40	分析者	:System Administrator
Modified Date	:2017/10/16 11:11:42	解析者	:System Administrator

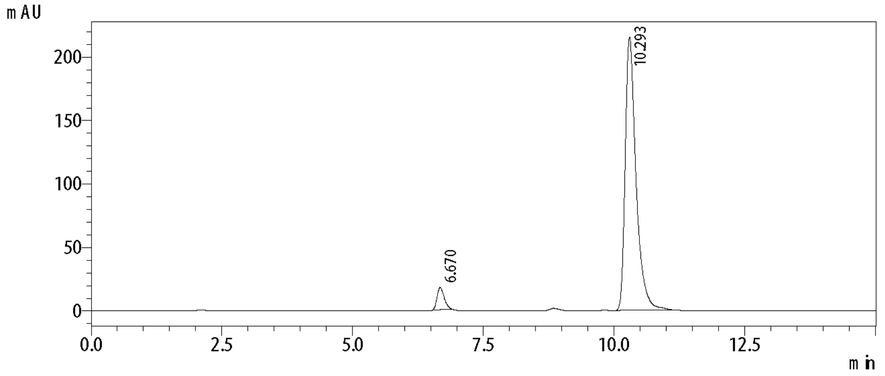

PDA Ch1 254nm

Peak No.	RT (min)	Area	Height	% Area
1	6.670	163485	17533	5.018
2	10.293	3094691	215581	94.982
Total		3258176	233115	100.000

UVスペクトル

保持時間 :6.670 min

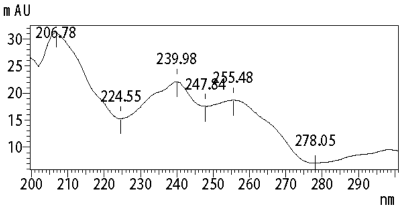

保持時間 :10.293 min

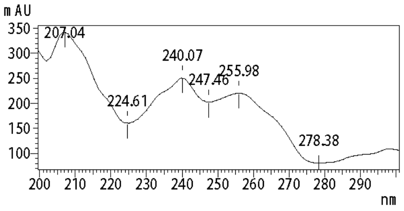

D:¥Data¥LY¥data¥LY-5-3d.lcd

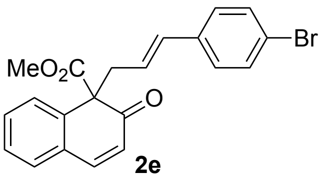

2e

=== Shimadzu LabSolutions ===

Sample :
ID :
Data Name :LY-5-3g-rac.lcd
Method Name :C4-20-80-1-20min.lcm
Batch Nam :LY-5-3g.lcb
Vial :1-20 サンプルタイプ :Unknown
Inj. Volume :1 uL
Acquisition Date :2017/10/18 12:30:32 分析者 :System Administrator
Modified Date :2017/10/18 14:38:47 解析者 :System Administrator

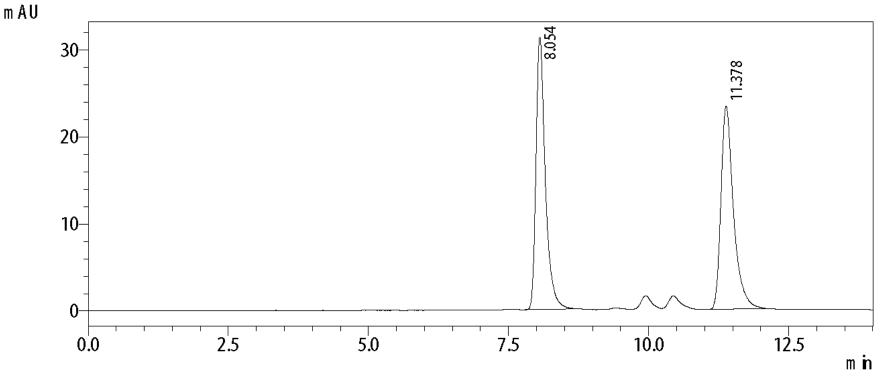

PDA Ch1 254nm

Peak No.	RT (min)	Area	Height	% Area
1	8.054	362225	31340	49.947
2	11.378	363001	23380	50.053
Total		725226	54720	100.000

UVスペクトル

保持時間 :8.054 min

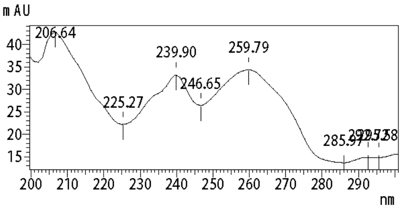

保持時間 :11.378 min

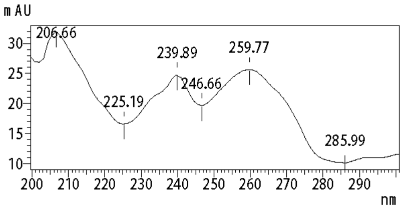

D:¥Data¥LY¥data¥LY-5-3g-rac.lcd

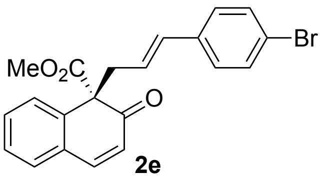

2e

3/1/2018 9:07:57 PM Page 1 / 1

=== Shimadzu LabSolutions ===

Sample	:		
ID	:		
Data Name	:LY-5-3g.lcd		
Method Name	:C4-20-80-1-20min.lcm		
Batch Nam	:LY-5-3g.lcb		
Vial	:1-21	サンプルタイプ	:Unknown
Inj. Volume	:1 uL		
Acquisition Date	:2017/10/18 12:50:57	分析者	:System Administrator
Modified Date	:2017/10/31 20:46:19	解析者	:System Administrator

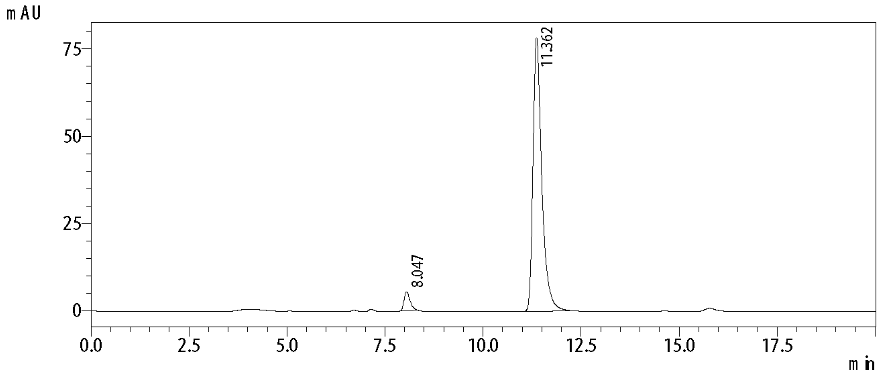

PDA Ch1 254nm

Peak No.	RT (min)	Area	Height	% Area
1	8.047	57733	5418	4.505
2	11.362	1223770	78413	95.495
Total		1281502	83831	100.000

UVスペクトル

保持時間 :8.047 min

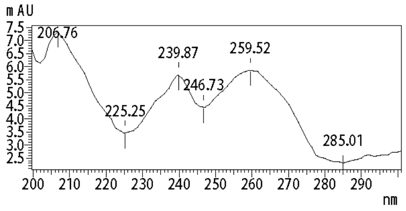

保持時間 :11.362 min

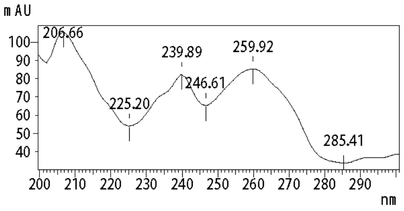

D:¥Data¥LY¥data¥LY-5-3g.lcd

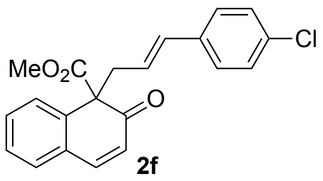

=== Shimadzu LabSolutions ===

Sample	:		
ID	:		
Data Name	:LY-5-3e-rac.lcd		
Method Name	:C4-20-80-1-15min.lcm		
Batch Nam	:LY-5-3e.lcb		
Vial	:1-18	サンプルタイプ	:Unknown
Inj. Volume	:1 uL		
Acquisition Date	:2017/10/05 17:35:14	分析者	:System Administrator
Modified Date	:2017/10/05 19:31:52	解析者	:System Administrator

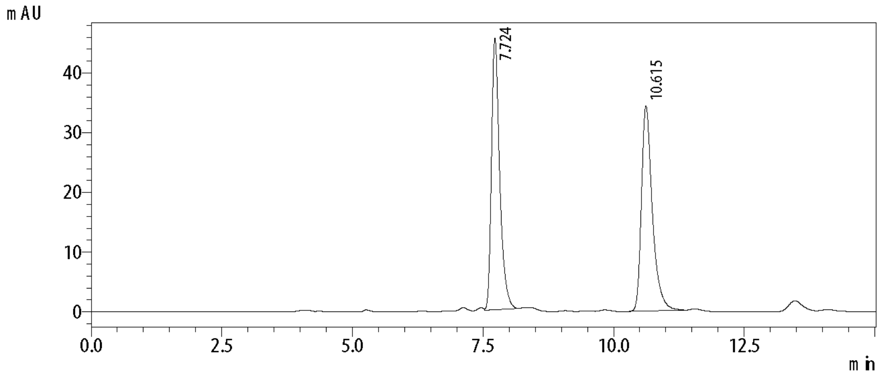

PDA Ch1 254nm

Peak No.	RT (min)	Area	Height	% Area
1	7.724	494481	45546	49.788
2	10.615	498700	34401	50.212
Total		993181	79947	100.000

UVスペクトル

保持時間 :7.724 min

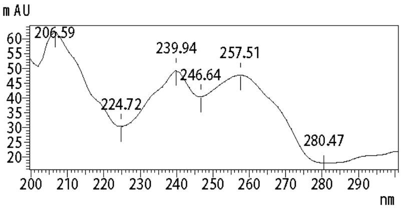

保持時間 :10.615 min

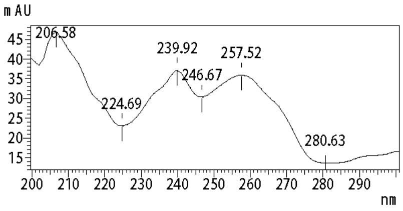

D:¥Data¥LY¥data¥LY-5-3e-rac.lcd

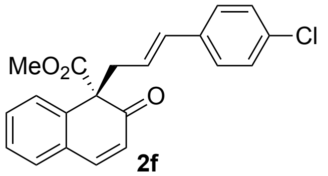

2f

=== Shimadzu LabSolutions ===

Sample	:
ID	:
Data Name	:LY-5-3e.lcd
Method Name	:C4-20-80-1-15min.lcm
Batch Nam	:LY-5-3e.lcb
Vial	:1-19
Inj. Volume	:1 uL
Acquisition Date	:2017/10/05 17:50:39
Modified Date	:2017/10/05 19:31:36

サンプルタイプ	:Unknown
分析者	:System Administrator
解析者	:System Administrator

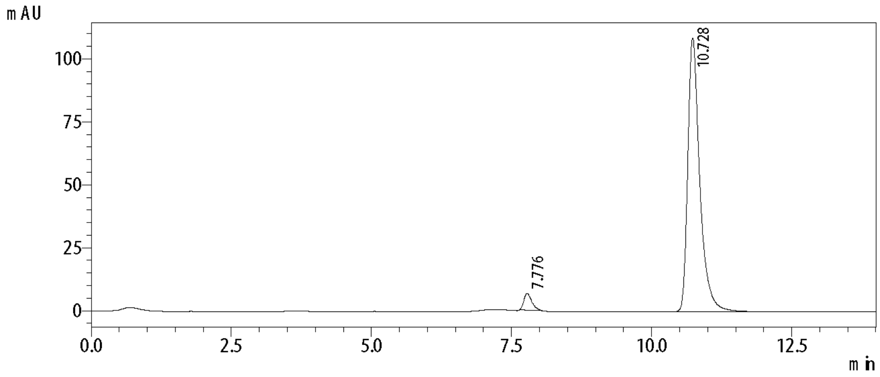

PDA Ch1 254nm

Peak No.	RT (min)	Area	Height	% Area
1	7.776	65545	6539	3.919
2	10.728	1606866	108680	96.081
Total		1672411	115219	100.000

UVスペクトル

保持時間 :7.776 min

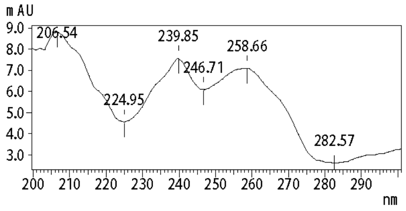

保持時間 :10.728 min

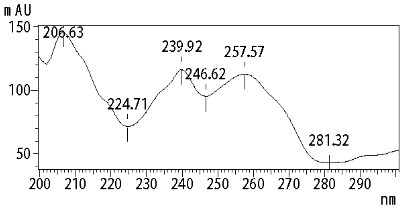

D:¥Data¥LY¥data¥LY-5-3e.lcd

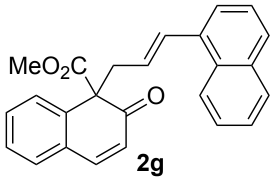

2g

=== Shimadzu LabSolutions ===

Sample	:		
ID	:		
Data Name	:LY-5-3i-rac.lcd		
Method Name	:安定化C4-20-80-1-20min.lcm		
Batch Nam	:LY-5-3i.lcb		
Vial	:1-21	サンプルタイプ	:Unknown
Inj. Volume	:1 uL		
Acquisition Date	:2018/05/25 16:09:55	分析者	:System Administrator
Modified Date	:2018/05/25 16:29:59	解析者	:System Administrator

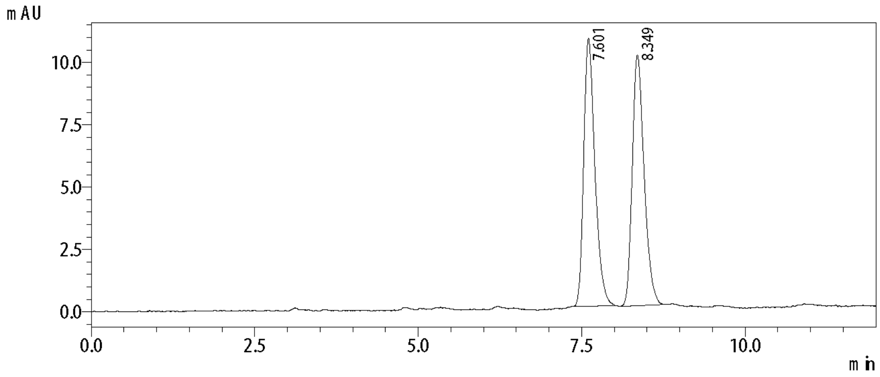

PDA Ch1 254nm

Peak No.	RT (min)	Area	Height	% Area
1	7.601	127764	10759	50.189
2	8.349	126801	10055	49.811
Total		254564	20814	100.000

UVスペクトル

保持時間 :7.601 min

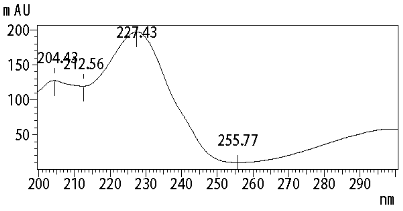

保持時間 :8.349 min

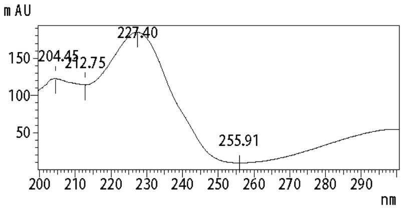

G:¥LY-5-3i-rac.lcd

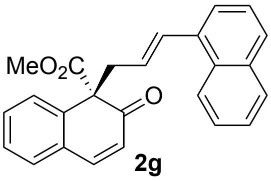

2g

=== Shimadzu LabSolutions ===

Sample :
ID :
Data Name :LY-5-3i.lcd
Method Name :安定化C4-20-80-1-20min.lcm
Batch Nam :LY-5-3i.lcb
Vial :1-22 サンプルタイプ :Unknown
Inj. Volume :1 uL
Acquisition Date :2018/05/25 15:49:37 分析者 :System Administrator
Modified Date :2018/05/25 16:09:40 解析者 :System Administrator

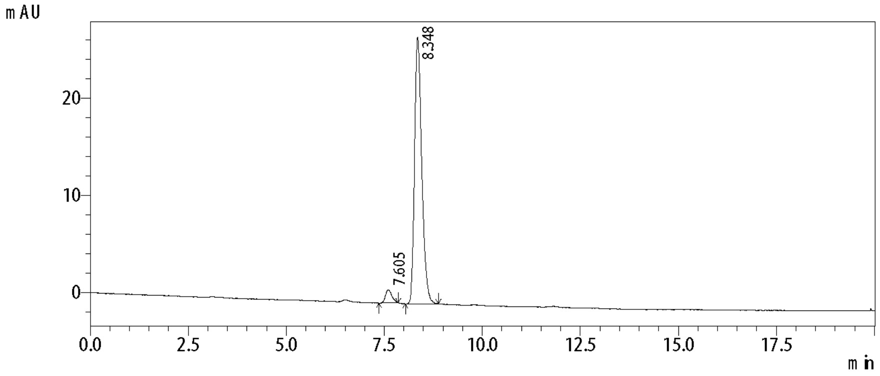

PDA Ch1 254nm

Peak No.	RT (min)	Area	Height	% Area
1	7.605	15259	1357	4.167
2	8.348	350911	27474	95.833
Total		366170	28831	100.000

UVスペクトル

保持時間 :7.605 min

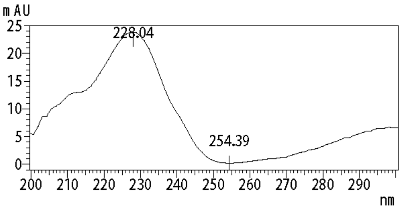

保持時間 :8.348 min

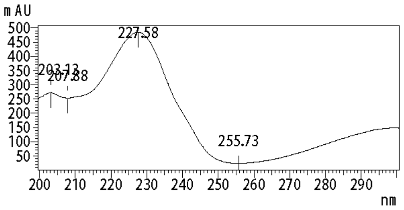

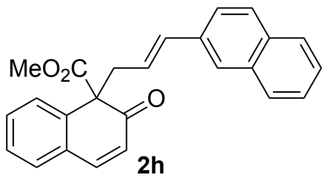

2h

=== Shimadzu LabSolutions ===

Sample :
ID :
Data Name :LY-5-3h-rac.lcd
Method Name :C4-20-80-1-20min.lcm
Batch Nam :LY-5-3h.lcb
Vial :1-18 サンプルタイプ :Unknown
Inj. Volume :1 uL
Acquisition Date :2017/10/12 21:41:32 分析者 :System Administrator
Modified Date :2017/10/13 15:01:40 解析者 :System Administrator

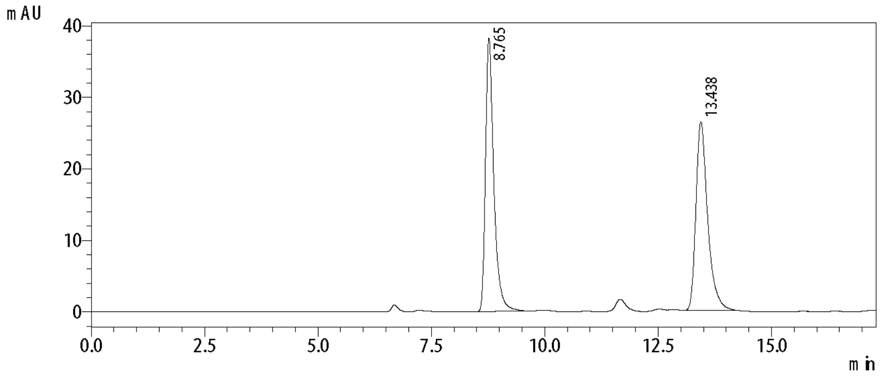

PDA Ch1 254nm

Peak No.	RT (min)	Area	Height	% Area
1	8.765	486146	38285	50.458
2	13.438	477312	26397	49.542
Total		963458	64682	100.000

UVスペクトル

保持時間 :8.765 min

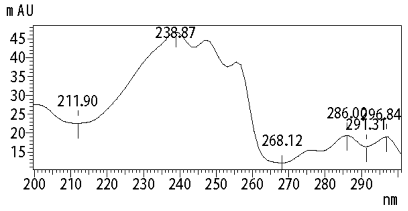

保持時間 :13.438 min

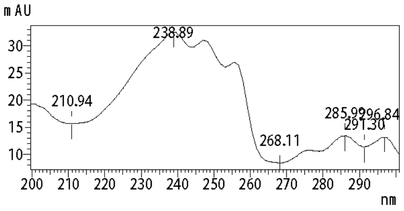

D:¥Data¥LY¥data¥LY-5-3h-rac.lcd

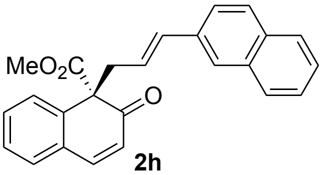

2h

=== Shimadzu LabSolutions ===

Sample	:		
ID	:		
Data Name	:LY-5-3h.lcd		
Method Name	:C4-20-80-1-20min.lcm		
Batch Nam	:LY-5-3h.lcb		
Vial	:1-19	サンプルタイプ	:Unknown
Inj. Volume	:1 uL		
Acquisition Date	:2017/10/12 22:01:58	分析者	:System Administrator
Modified Date	:2017/10/13 14:54:16	解析者	:System Administrator

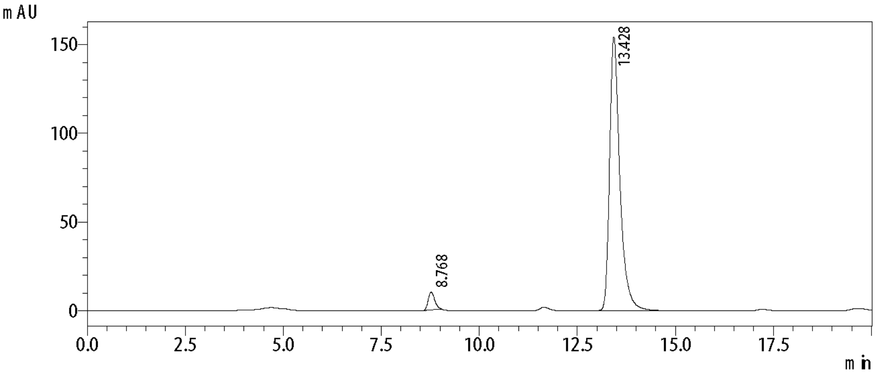

PDA Ch1 254nm

Peak No.	RT (min)	Area	Height	% Area
1	8.768	114650	10013	3.931
2	13.428	2802111	154136	96.069
Total		2916760	164149	100.000

UVスペクトル

保持時間 :8.768 min

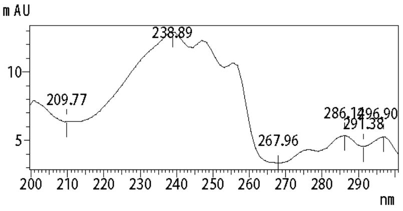

保持時間 :13.428 min

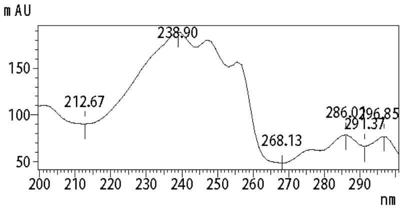

D:¥Data¥LY¥data¥LY-5-3h.lcd

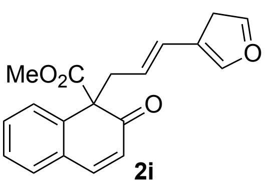

2i

=== Shimadzu LabSolutions ===

Sample :
ID :
Data Name :LY-5-31-rac001.lcd
Method Name :C4-20-80-1-20min.lcm
Batch Nam :LY-5-31.lcb
Vial :1-18
Inj. Volume :1 uL
Acquisition Date :2017/11/04 18:58:30
Modified Date :2017/11/04 19:18:33
サンプルタイプ :Unknown
分析者 :System Administrator
解析者 :System Administrator

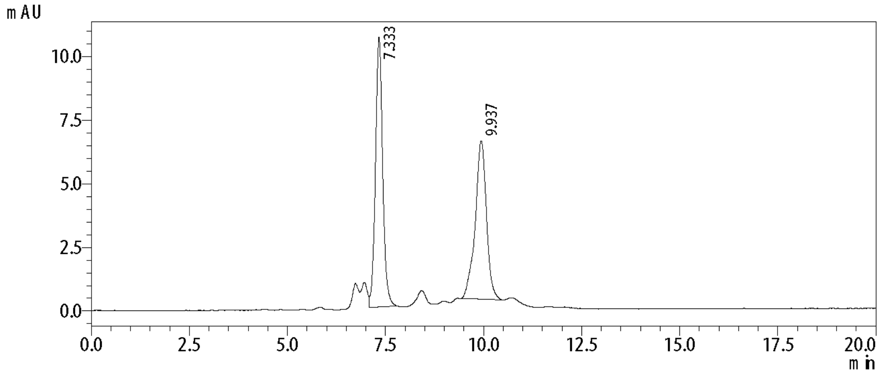

PDA Ch1 254nm

Peak No.	RT (min)	Area	Height	% Area
1	7.333	135221	10621	52.241
2	9.937	123619	6221	47.759
Total		258841	16842	100.000

UVスペクトル

D:¥Data¥LY¥data¥LY-5-31-rac001.lcd

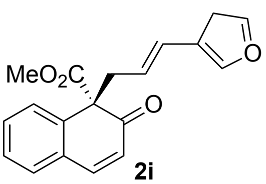

2i

=== Shimadzu LabSolutions ===

Sample :
ID :
Data Name :LY-5-31003.lcd
Method Name :C4-20-80-1-20min.lcm
Batch Nam :LY-5-31-1.lcb
Vial :1-62
Inj. Volume :1 uL
Acquisition Date :2018/03/16 13:18:52
Modified Date :2018/03/16 14:07:21
サンプルタイプ :Unknown
分析者 :System Administrator
解析者 :System Administrator

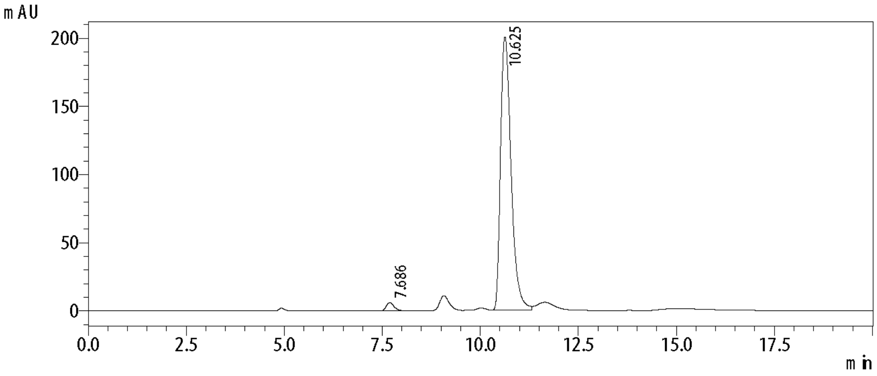

PDA Ch1 254nm

Peak No.	RT (min)	Area	Height	% Area
1	7.686	78221	5876	2.044
2	10.625	3748648	200591	97.956
Total		3826869	206467	100.000

UVスペクトル

保持時間 :7.686 min

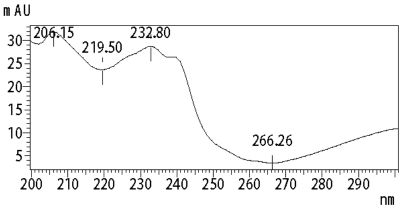

保持時間 :10.625 min

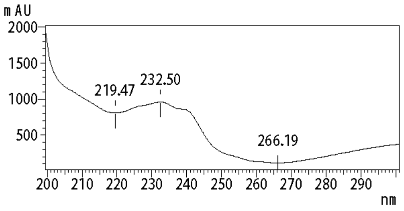

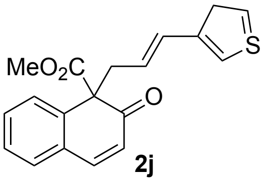

2j

=== Shimadzu LabSolutions ===

Sample	:		
ID	:		
Data Name	:LY-5-3n001.lcd		
Method Name	:C4-20-80-1-20min.lcm		
Batch Nam	:LY-5-3n.lcb		
Vial	:1-63	サンプルタイプ	:Unknown
Inj. Volume	:1 uL		
Acquisition Date	:2018/03/02 0:24:36	分析者	:System Administrator
Modified Date	:2018/03/02 0:44:39	解析者	:System Administrator

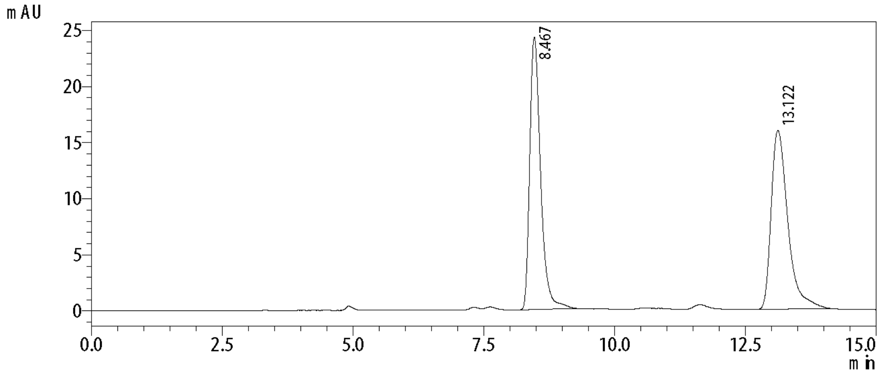

PDA Ch1 254nm

Peak No.	RT (min)	Area	Height	% Area
1	8.467	348833	24301	49.764
2	13.122	352148	15927	50.236
Total		700981	40229	100.000

UVスペクトル

保持時間 :8.467 min

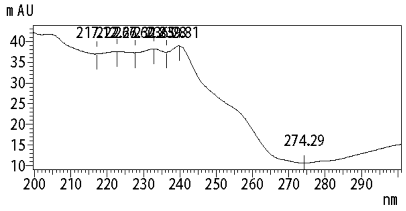

保持時間 :13.122 min

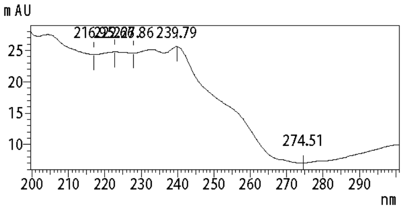

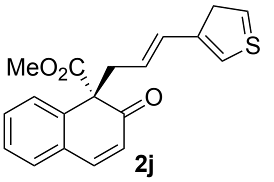

2j

=== Shimadzu LabSolutions ===

Sample :
ID :
Data Name :LY-5-3n-rac001.lcd
Method Name :C4-20-80-1-20min.lcm
Batch Nam :LY-5-3n.lcb
Vial :1-64 サンプルタイプ :Unknown
Inj. Volume :1 uL
Acquisition Date :2018/03/02 0:45:02 分析者 :System Administrator
Modified Date :2018/03/12 15:26:22 解析者 :System Administrator

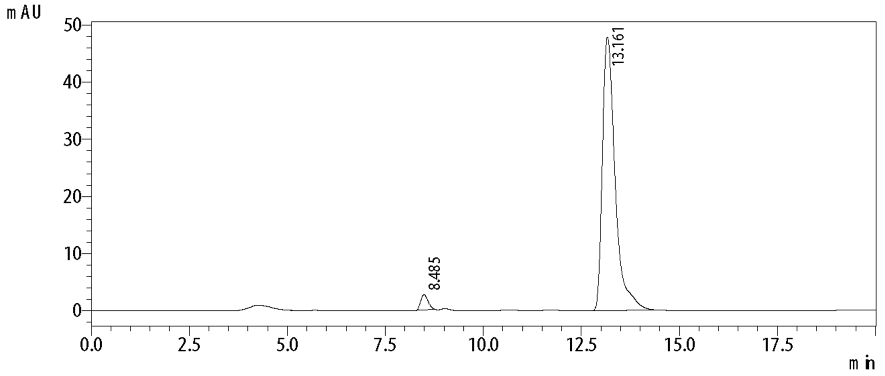

PDA Ch1 254nm

Peak No.	RT (min)	Area	Height	% Area
1	8.485	34432	2660	3.084
2	13.161	1082004	47925	96.916
Total		1116436	50585	100.000

UVスペクトル

保持時間 :8.485 min

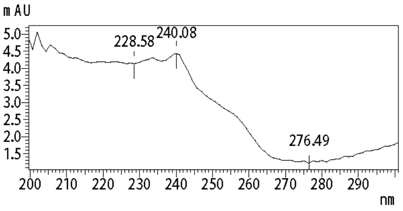

保持時間 :13.161 min

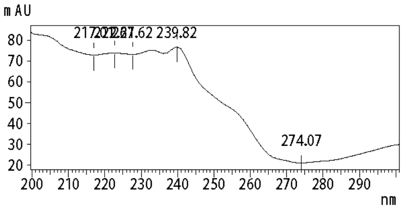

D:¥Data¥LY¥data¥LY-5-3n-rac001.lcd

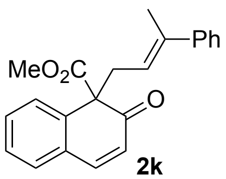

2k

=== Shimadzu LabSolutions ===

Sample	:		
ID	:		
Data Name	:LY-5-1a-rac.lcd		
Method Name	:C3-80-20-1ml-20min.lcm		
Batch Nam	:LY-5-3x.lcb		
Vial	:1-18	サンプルタイプ	:Unknown
Inj. Volume	:1 uL		
Acquisition Date	:2017/10/11 21:42:39	分析者	:System Administrator
Modified Date	:2017/10/11 22:02:42	解析者	:System Administrator

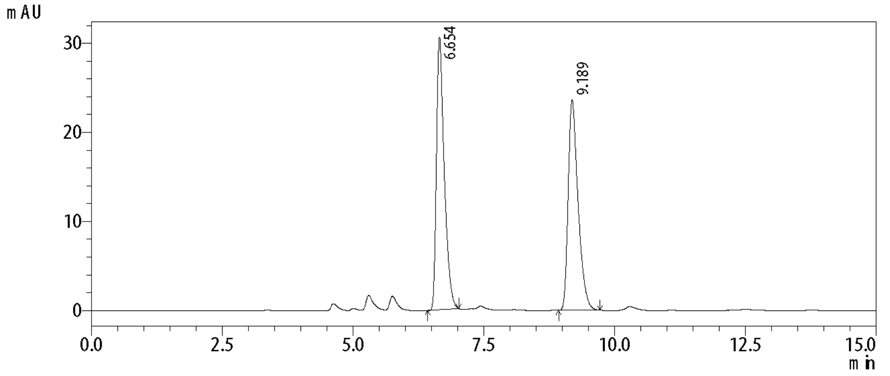

PDA Ch1 254nm

Peak No.	RT (min)	Area	Height	% Area
1	6.654	320470	30647	50.823
2	9.189	310094	23635	49.177
Total		630565	54283	100.000

UVスペクトル

保持時間 :6.654 min

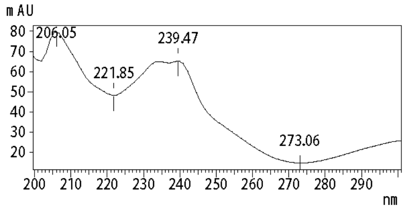

保持時間 :9.189 min

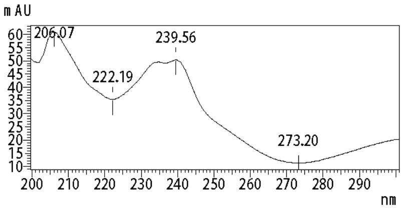

D:¥Data¥LY¥data¥LY-5-1a-rac.lcd

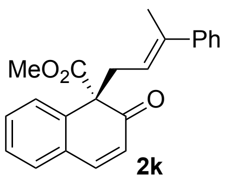

2k

=== Shimadzu LabSolutions ===

Sample :
ID :
Data Name :LY-5-1a.lcd
Method Name :C3-80-20-1ml-20min.lcm
Batch Nam :LY-5-3x.lcb
Vial :1-19
Inj. Volume :1 uL
Acquisition Date :2017/10/11 22:03:05
Modified Date :2017/10/11 22:23:08

サンプルタイプ :Unknown
分析者 :System Administrator
解析者 :System Administrator

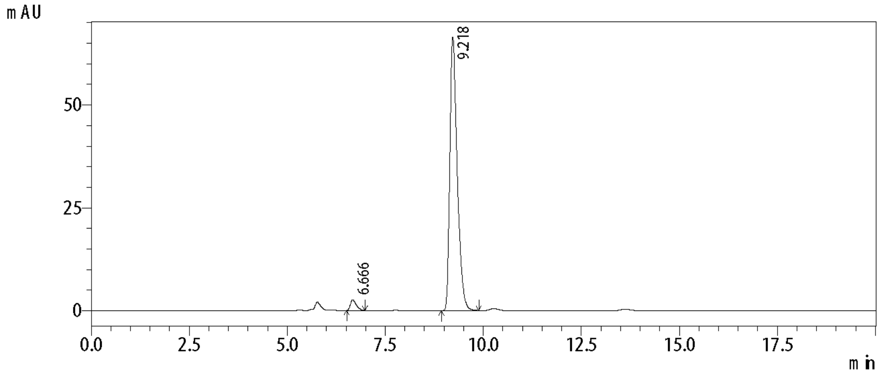

PDA Ch1 254nm

Peak No.	RT (min)	Area	Height	% Area
1	6.666	27512	2647	3.056
2	9.218	872661	66544	96.944
Total		900173	69191	100.000

UVスペクトル

保持時間 :6.666 min

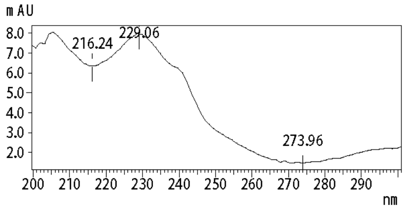

保持時間 :9.218 min

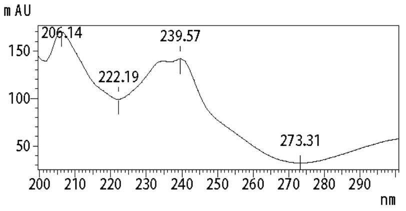

D:¥Data¥LY¥data¥LY-5-1a.lcd

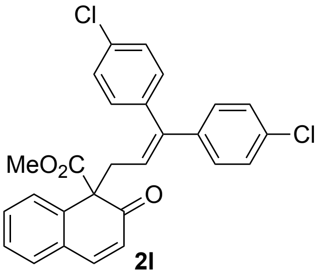

2l

=== Shimadzu LabSolutions ===

Sample	:		
ID	:		
Data Name	:LY-5-1b-rac.lcd		
Method Name	:C3-80-20-1ml-20min.lcm		
Batch Nam	:LY-5-1b.lcb		
Vial	:1-18	サンプルタイプ	:Unknown
Inj. Volume	:1 uL		
Acquisition Date	:2017/11/01 14:06:09	分析者	:System Administrator
Modified Date	:2017/11/13 20:33:01	解析者	:System Administrator

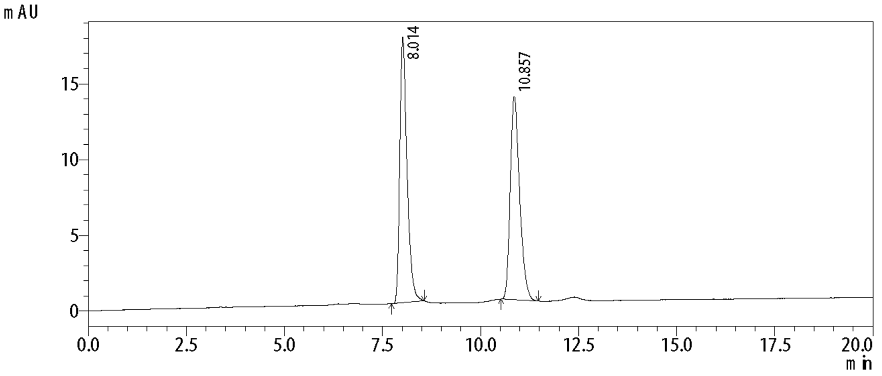

PDA Ch1 254nm

Peak No.	RT (min)	Area	Height	% Area
1	8.014	224968	17545	50.266
2	10.857	222589	13394	49.734
Total		447557	30939	100.000

UVスペクトル

保持時間 :8.014 min

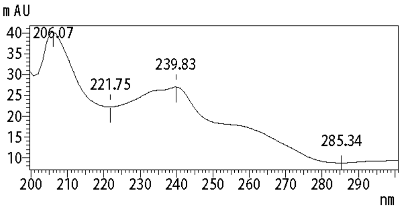

保持時間 :10.857 min

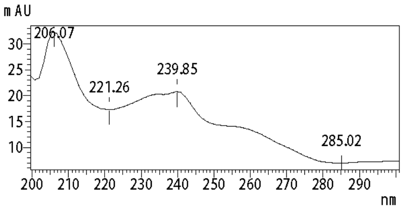

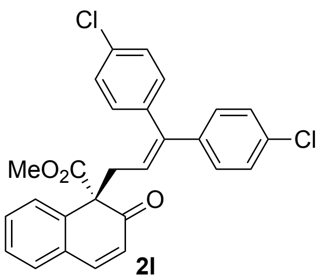

2l

=== Shimadzu LabSolutions ===

Sample	:		
ID	:		
Data Name	:LY-5-1b.lcd		
Method Name	:C3-80-20-1ml-20min.lcm		
Batch Nam	:LY-5-1b.lcb		
Vial	:1-19	サンプルタイプ	:Unknown
Inj. Volume	:1 uL		
Acquisition Date	:2017/11/01 14:26:33	分析者	:System Administrator
Modified Date	:2017/11/01 15:05:56	解析者	:System Administrator

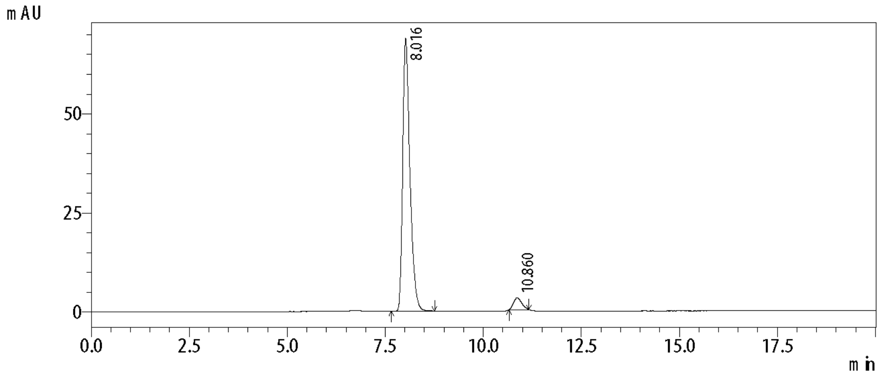

PDA Ch1 254nm

Peak No.	RT (min)	Area	Height	% Area
1	8.016	881458	68974	95.205
2	10.860	44399	3003	4.795
Total		925857	71977	100.000

UVスペクトル

保持時間 :8.016 min

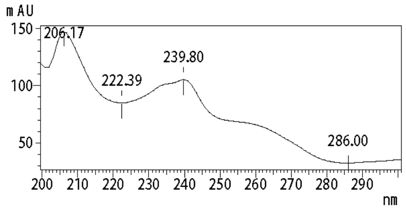

保持時間 :10.860 min

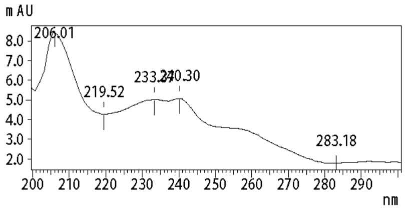

D:¥Data¥LY¥data¥LY-5-1b.lcd

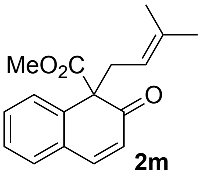

2m

=== Shimadzu LabSolutions ===

Sample	:		
ID	:		
Data Name	:LY-5-1d-rac.lcd		
Method Name	:C2-20-80-1-20min.lcm		
Batch Nam	:LY-5-1d-1.lcb		
Vial	:1-18	サンプルタイプ	:Unknown
Inj. Volume	:1 uL		
Acquisition Date	:2017/11/20 22:32:58	分析者	:System Administrator
Modified Date	:2017/11/20 22:53:01	解析者	:System Administrator

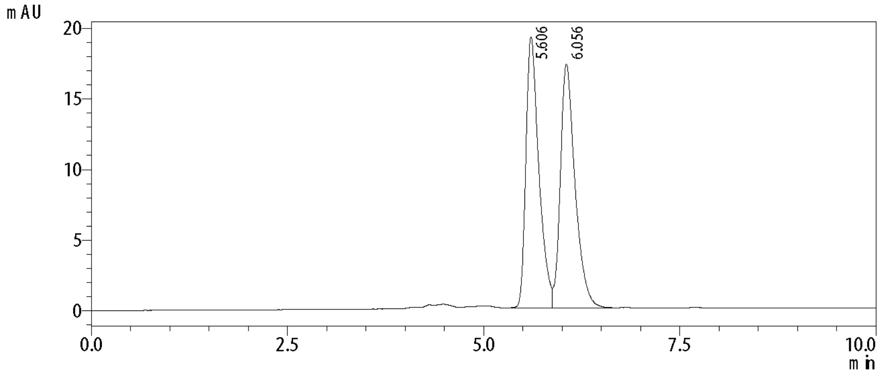

PDA Ch1 290nm

Peak No.	RT (min)	Area	Height	% Area
1	5.606	220751	19172	49.563
2	6.056	224645	17254	50.437
Total		445395	36426	100.000

UVスペクトル

保持時間 :5.606 min

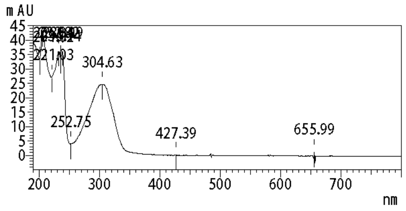

保持時間 :6.056 min

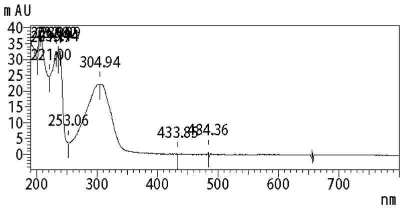

D:¥Data¥LY¥data¥LY-5-1d-rac.lcd

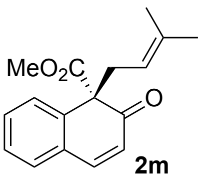

2m

=== Shimadzu LabSolutions ===

Sample	:		
ID	:		
Data Name	:LY-5-1d.lcd		
Method Name	:C2-20-80-1-20min.lcm		
Batch Nam	:LY-5-1d-1.lcb		
Vial	:1-19	サンプルタイプ	:Unknown
Inj. Volume	:1 uL		
Acquisition Date	:2017/11/20 22:53:32	分析者	:System Administrator
Modified Date	:2017/11/21 21:53:03	解析者	:System Administrator

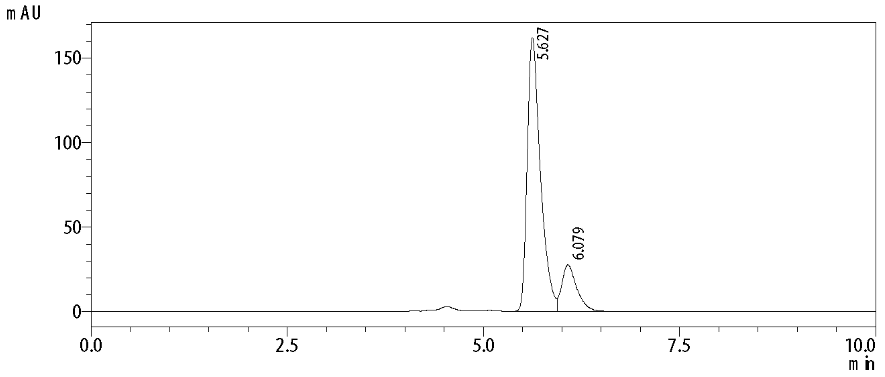

PDA Ch1 290nm

Peak No.	RT (min)	Area	Height	% Area
1	5.627	1888999	161852	84.027
2	6.079	359074	27495	15.973
Total		2248073	189347	100.000

UVスペクトル

保持時間 :5.627 min

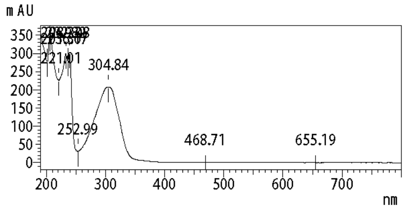

保持時間 :6.079 min

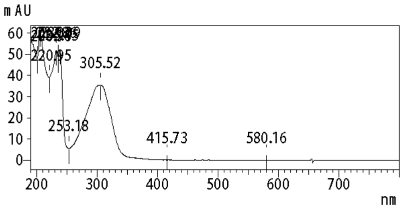

D:¥Data¥LY¥data¥LY-5-1d.lcd

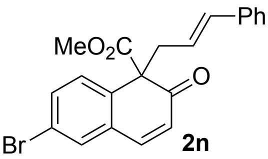

2n

=== Shimadzu LabSolutions ===

Sample :
ID :
Data Name :LY-5-130a-rac.lcd
Method Name :C4-20-80-1-20min.lcm
Batch Nam :LY-5-130a.lcb
Vial :1-18
サンプルタイプ :Unknown
Inj. Volume :1 uL
Acquisition Date :2017/11/06 10:52:03
分析者 :System Administrator
Modified Date :2017/11/06 11:12:06
解析者 :System Administrator

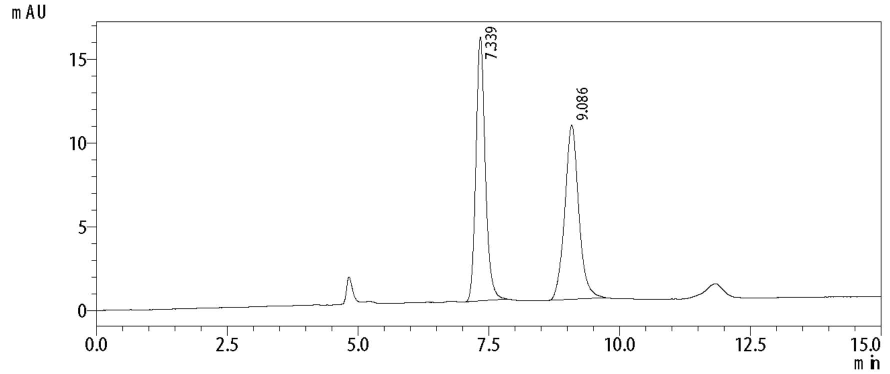

PDA Ch1 254nm

Peak No.	RT (min)	Area	Height	% Area
1	7.339	194816	15741	50.325
2	9.086	192296	10382	49.675
Total		387111	26123	100.000

UVスペクトル

保持時間 :7.339 min

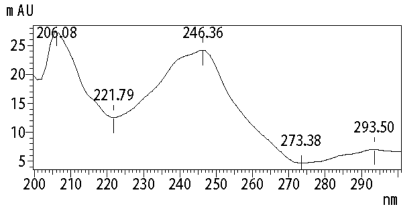

保持時間 :9.086 min

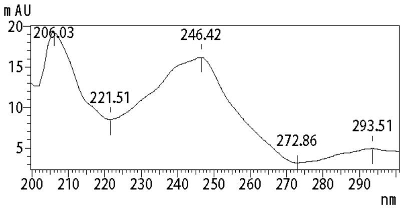

D:¥Data¥LY¥data¥LY-5-130a-rac.lcd

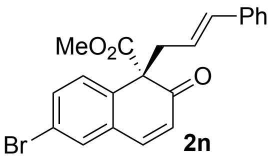

2n

=== Shimadzu LabSolutions ===

Sample :
ID :
Data Name :LY-5-130a.lcd
Method Name :C4-20-80-1-20min.lcm
Batch Nam :LY-5-130a.lcb
Vial :1-19 サンプルタイプ :Unknown
Inj. Volume :1 uL
Acquisition Date :2017/11/06 11:12:29 分析者 :System Administrator
Modified Date :2017/11/06 11:32:31 解析者 :System Administrator

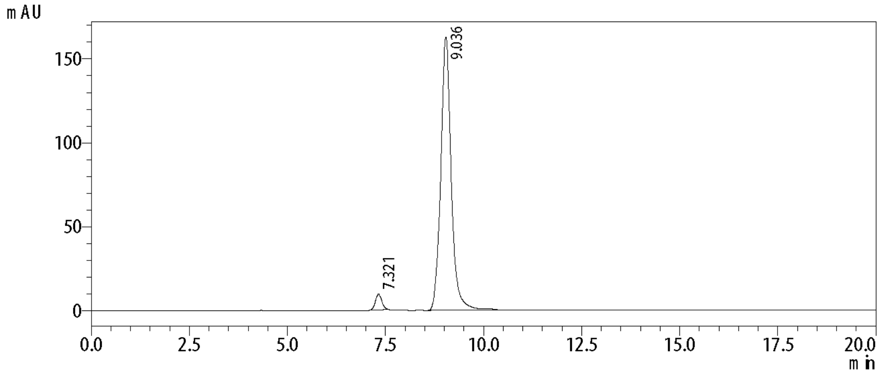

PDA Ch1 254nm

Peak No.	RT (min)	Area	Height	% Area
1	7.321	108106	9406	3.447
2	9.036	3028153	162708	96.553
Total		3136260	172113	100.000

UVスペクトル

保持時間 :7.321 min

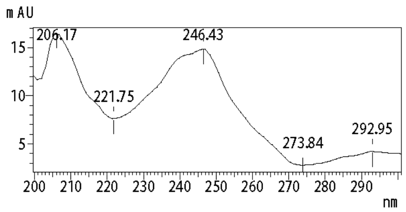

保持時間 :9.036 min

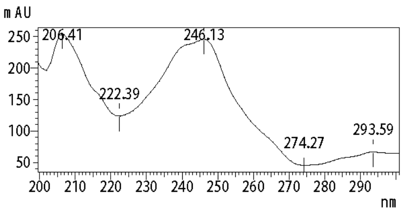

D:¥Data¥LY¥data¥LY-5-130a.lcd

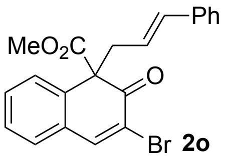

2o

=== Shimadzu LabSolutions ===

Sample :
ID :
Data Name :LY-5-130b-rac.lcd
Method Name :C4-20-80-1-20min.lcm
Batch Nam :LY-5-130b.lcb
Vial :1-18 サンプルタイプ :Unknown
Inj. Volume :1 uL
Acquisition Date :2017/12/14 16:04:51 分析者 :System Administrator
Modified Date :2017/12/14 17:04:45 解析者 :System Administrator

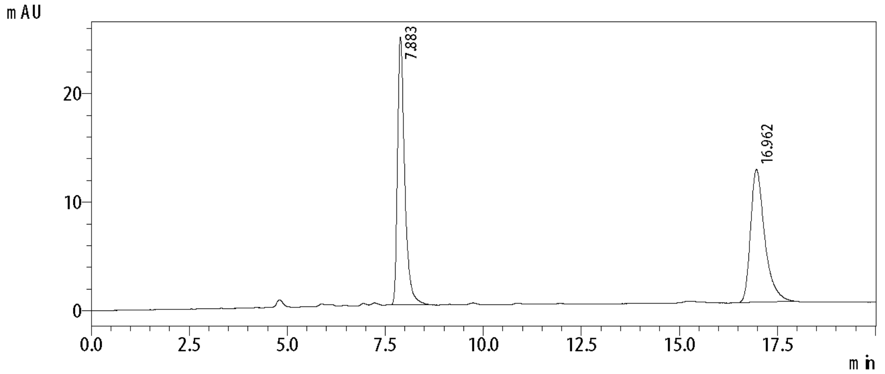

PDA Ch1 254nm

Peak No.	RT (min)	Area	Height	% Area
1	7.883	311886	24649	50.439
2	16.962	306459	12228	49.561
Total		618344	36878	100.000

UVスペクトル

保持時間 :7.883 min

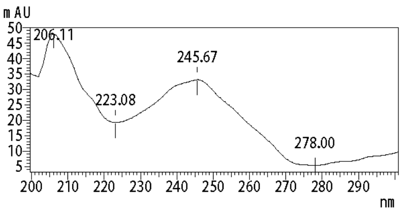

保持時間 :16.962 min

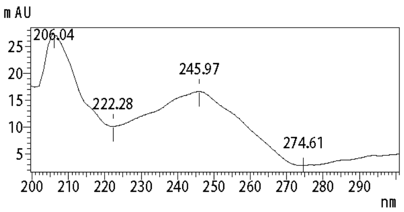

D:¥Data¥LY¥data¥LY-5-130b-rac.lcd

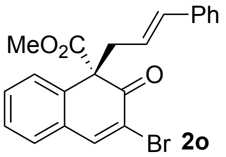

2o

=== Shimadzu LabSolutions ===

Sample	:		
ID	:		
Data Name	:LY-5-130b.lcd		
Method Name	:C4-20-80-1-20min.lcm		
Batch Nam	:LY-5-130b.lcb		
Vial	:1-19	サンプルタイプ	:Unknown
Inj. Volume	:1 uL		
Acquisition Date	:2017/12/14 16:25:16	分析者	:System Administrator
Modified Date	:2017/12/14 17:05:55	解析者	:System Administrator

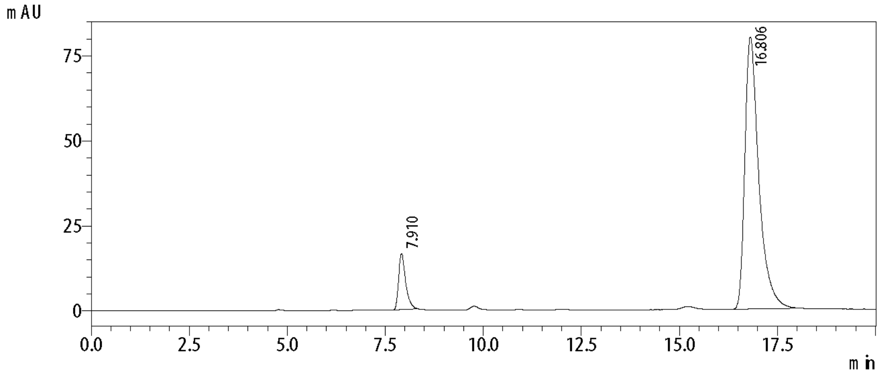

PDA Ch1 254nm

Peak No.	RT (min)	Area	Height	% Area
1	7.910	203379	16509	9.291
2	16.806	1985642	80097	90.709
Total		2189021	96606	100.000

UVスペクトル

保持時間 :7.910 min

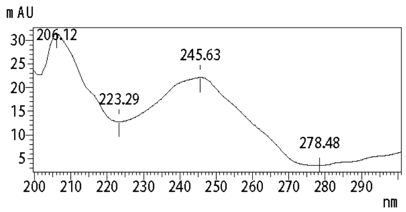

保持時間 :16.806 min

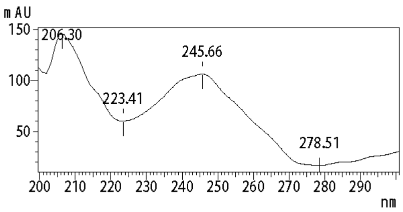

D:¥Data¥LY¥data¥LY-5-130b.lcd

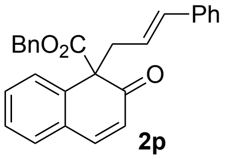

2p

=== Shimadzu LabSolutions ===

Sample :
ID :
Data Name :LY-5-130g-rac.lcd
Method Name :C3-80-20-1ml-20min.lcm
Batch Nam :LY-5-130g.lcb
Vial :1-18 サンプルタイプ :Unknown
Inj. Volume :1 uL
Acquisition Date :2017/12/23 16:17:31 分析者 :System Administrator
Modified Date :2017/12/23 16:37:33 解析者 :System Administrator

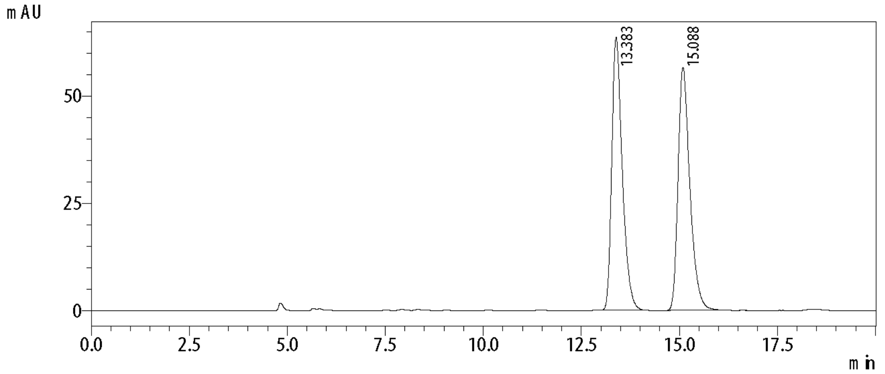

PDA Ch1 254nm

Peak No.	RT (min)	Area	Height	% Area
1	13.383	1184174	63605	49.935
2	15.088	1187247	56507	50.065
Total		2371421	120112	100.000

UVスペクトル

保持時間 :13.383 min

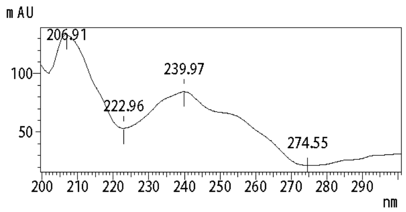

保持時間 :15.088 min

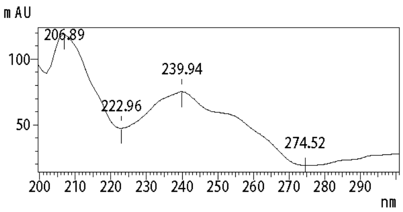

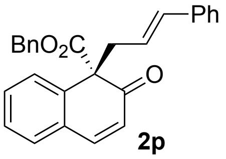

2p

12/25/2017 10:53:58 AM Page 1 / 1

=== Shimadzu LabSolutions ===

Sample	:		
ID	:		
Data Name	:LY-5-130g.lcd		
Method Name	:C3-80-20-1ml-20min.lcm		
Batch Nam	:LY-5-130g.lcb		
Vial	:1-19	サンプルタイプ	:Unknown
Inj. Volume	:1 uL		
Acquisition Date	:2017/12/23 16:37:55	分析者	:System Administrator
Modified Date	:2017/12/23 16:57:58	解析者	:System Administrator

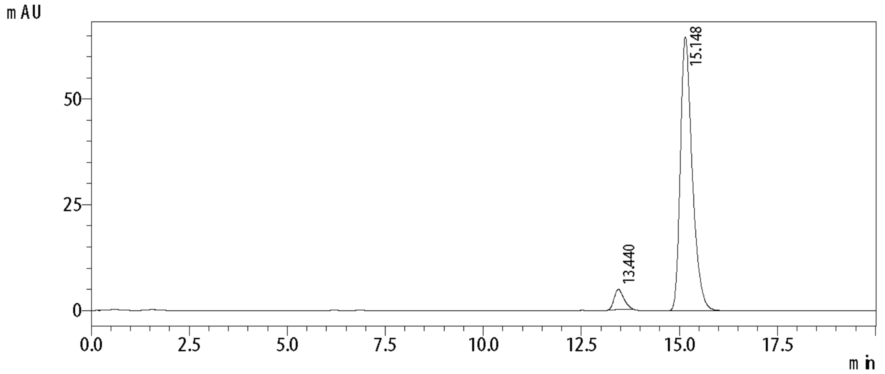

PDA Ch1 254nm

Peak No.	RT (min)	Area	Height	% Area
1	13.440	81218	4794	5.633
2	15.148	1360501	64755	94.367
Total		1441719	69550	100.000

UVスペクトル

保持時間 :13.440 min

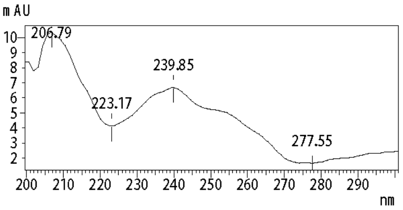

保持時間 :15.148 min

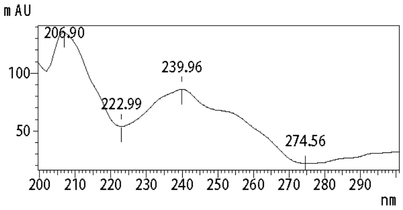

D:¥Data¥LY¥data¥LY-5-130g.lcd

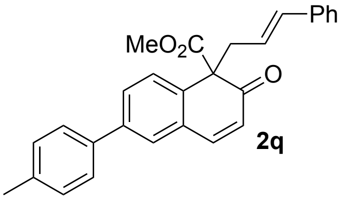

2q

3/1/2018 8:53:22 PM Page 1 / 1

=== Shimadzu LabSolutions ===

Sample	:		
ID	:		
Data Name	:LY-5-130d-rac.lcd		
Method Name	:C3-80-20-1ml-20min.lcm		
Batch Nam	:LY-5-130d.lcb		
Vial	:1-18	サンプルタイプ	:Unknown
Inj. Volume	:1 uL		
Acquisition Date	:2017/12/15 12:42:13	分析者	:System Administrator
Modified Date	:2017/12/15 13:36:27	解析者	:System Administrator

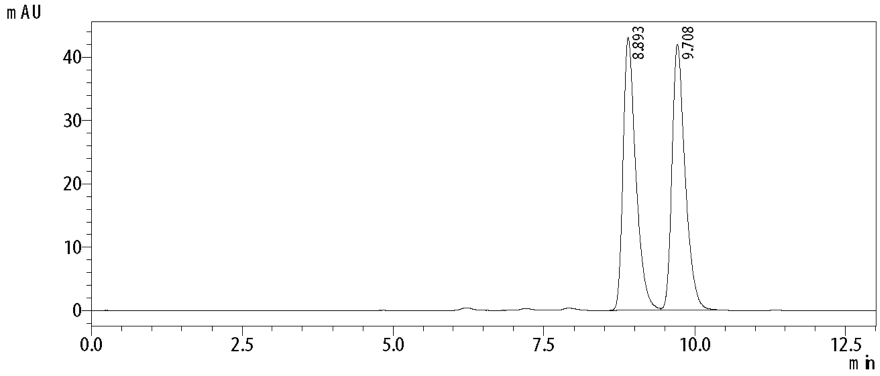

PDA Ch1 254nm

Peak No.	RT (min)	Area	Height	% Area
1	8.893	631616	43199	49.870
2	9.708	634918	41978	50.130
Total		1266534	85177	100.000

UVスペクトル

保持時間 :8.893 min

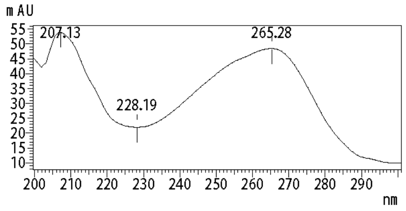

保持時間 :9.708 min

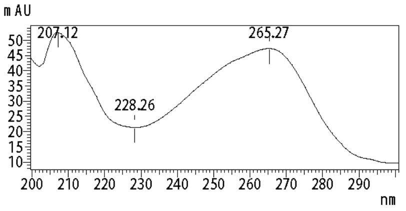

D:¥Data¥LY¥data¥LY-5-130d-rac.lcd

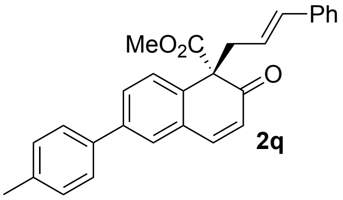

2q

=== Shimadzu LabSolutions ===

Sample	:		
ID	:		
Data Name	:LY-5-130d.lcd		
Method Name	:C3-80-20-1ml-20min.lcm		
Batch Nam	:LY-5-130d.lcb		
Vial	:1-19	サンプルタイプ	:Unknown
Inj. Volume	:1 uL		
Acquisition Date	:2017/12/15 13:02:37	分析者	:System Administrator
Modified Date	:2017/12/15 13:36:18	解析者	:System Administrator

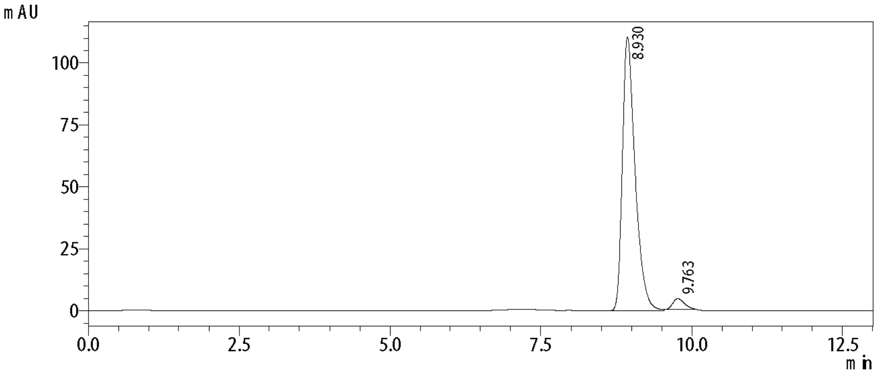

PDA Ch1 254nm

Peak No.	RT (min)	Area	Height	% Area
1	8.930	1611821	110398	96.447
2	9.763	59369	4339	3.553
Total		1671190	114737	100.000

UVスペクトル

保持時間 :8.930 min

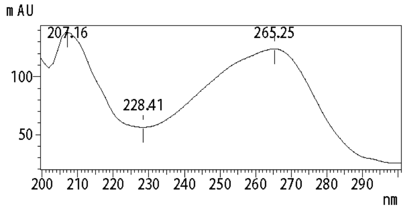

保持時間 :9.763 min

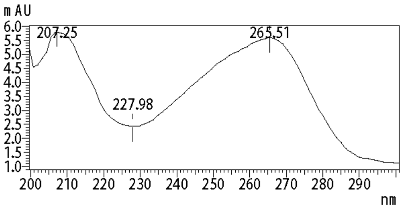

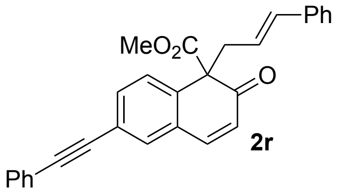

2r

=== Shimadzu LabSolutions ===

Sample :
ID :
Data Name :LY-5-130c-rac.lcd
Method Name :C4-20-80-1-20min.lcm
Batch Nam :LY-5-130c.lcb
Vial :1-18 サンプルタイプ :Unknown
Inj. Volume :1 uL
Acquisition Date :2017/12/14 20:59:44 分析者 :System Administrator
Modified Date :2017/12/14 21:19:46 解析者 :System Administrator

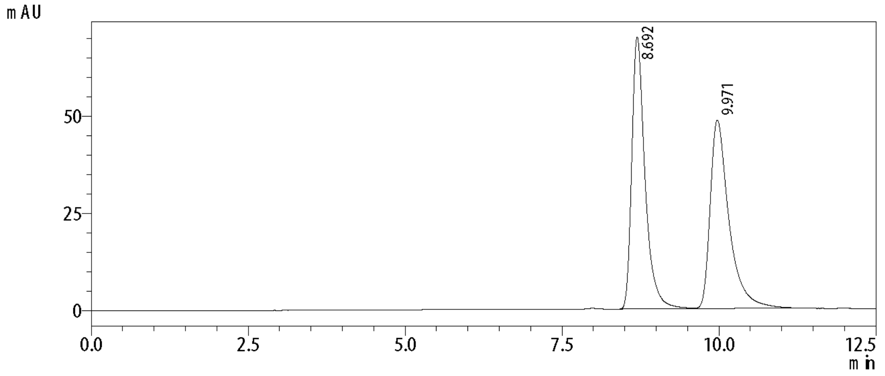

PDA Ch1 254nm

Peak No.	RT (min)	Area	Height	% Area
1	8.692	1029843	69996	50.576
2	9.971	1006395	48478	49.424
Total		2036238	118475	100.000

UVスペクトル

保持時間 :8.692 min

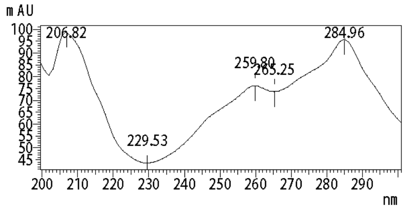

保持時間 :9.971 min

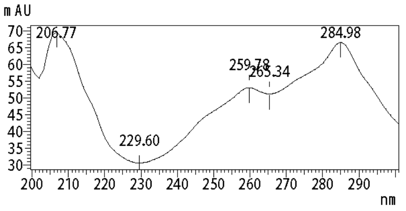

D:¥Data¥LY¥data¥LY-5-130c-rac.lcd

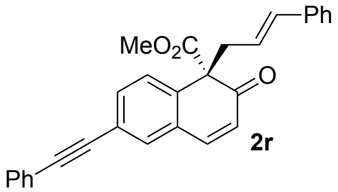

2r

=== Shimadzu LabSolutions ===

Sample	:
ID	:
Data Name	:LY-5-130c.lcd
Method Name	:C4-20-80-1-20min.lcm
Batch Nam	:LY-5-130c.lcb
Vial	:1-19
Inj. Volume	:1 uL
Acquisition Date	:2017/12/14 21:20:09
Modified Date	:2017/12/14 21:40:11
サンプルタイプ	:Unknown
分析者	:System Administrator
解析者	:System Administrator

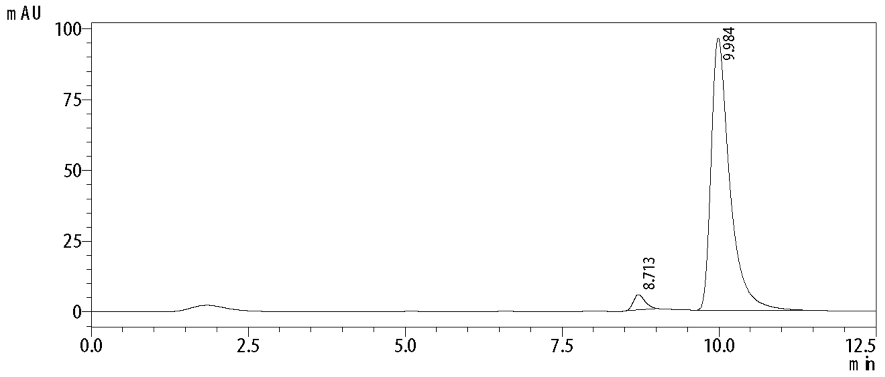

PDA Ch1 254nm

Peak No.	RT (min)	Area	Height	% Area
1	8.713	70842	5415	3.454
2	9.984	1980226	96241	96.546
Total		2051068	101656	100.000

UVスペクトル

保持時間 :8.713 min

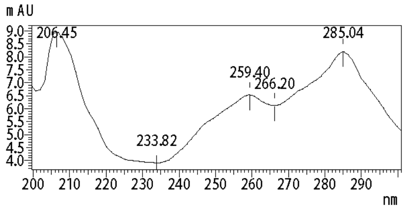

保持時間 :9.984 min

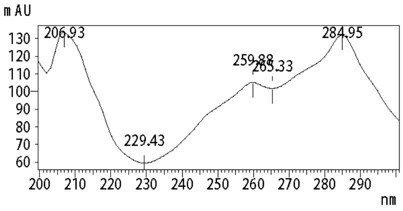

D:¥Data¥LY¥data¥LY-5-130c.lcd

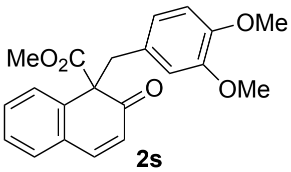

2s

=== Shimadzu LabSolutions ===

Sample	:
ID	:
Data Name	:LY-6-102c-rac.lcd
Method Name	:C2-20-80-1-20min.lcm
Batch Nam	:LY-6-102c.lcb
Vial	:1-61
Inj. Volume	:1 uL
Acquisition Date	:2018/06/12 22:39:17
Modified Date	:2018/06/13 10:01:41

サンプルタイプ	:Unknown
分析者	:System Administrator
解析者	:System Administrator

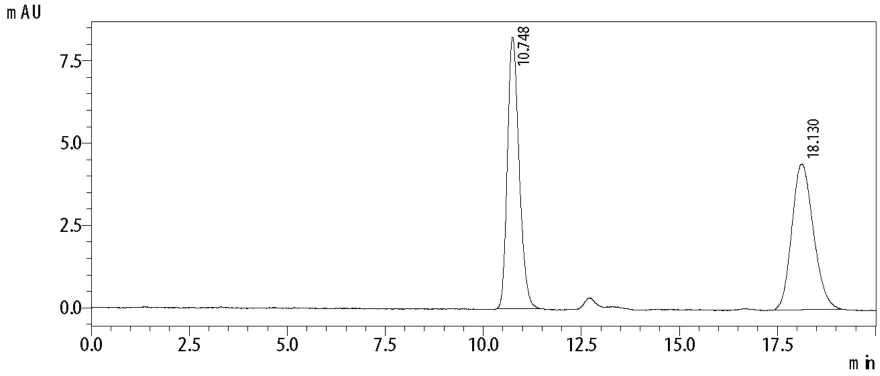

PDA Ch1 300nm

Peak No.	RT (min)	Area	Height	% Area
1	10.748	171078	8258	50.328
2	18.130	168849	4423	49.672
Total		339926	12682	100.000

UVスペクトル

保持時間 :10.748 min

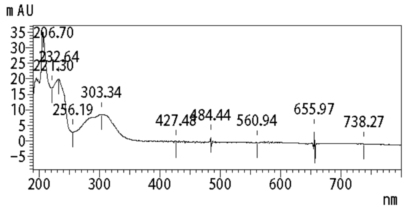

保持時間 :18.130 min

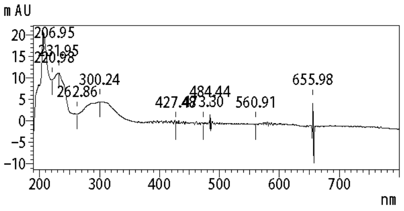

D:¥Data¥LY¥data¥LY-6-102c-rac.lcd

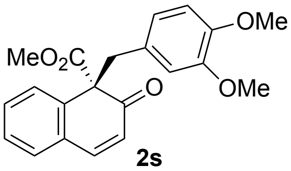

2s

=== Shimadzu LabSolutions ===

Sample	:		
ID	:		
Data Name	:LY-6-102c.lcd		
Method Name	:C2-20-80-1-20min.lcm		
Batch Nam	:LY-6-102c.lcb		
Vial	:1-62	サンプルタイプ	:Unknown
Inj.Volume	:1 uL		
Acquisition Date	:2018/06/12 22:59:50	分析者	:System Administrator
Modified Date	:2018/06/13 10:02:01	解析者	:System Administrator

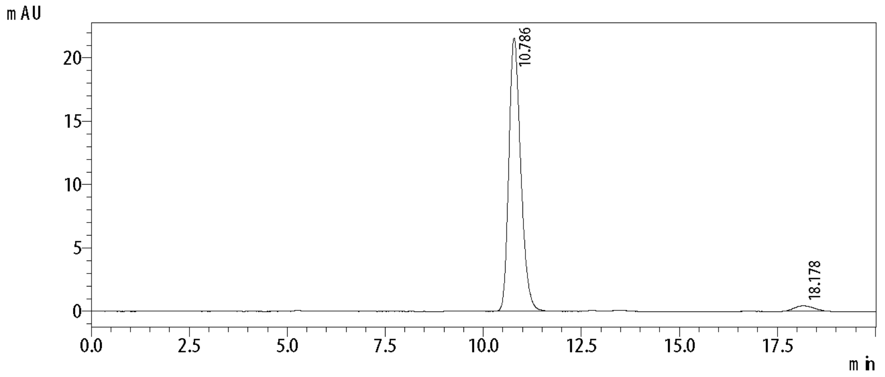

PDA Ch1 300nm

Peak No.	RT (min)	Area	Height	% Area
1	10.786	453985	21559	97.099
2	18.178	13563	412	2.901
Total		467549	21970	100.000

UVスペクトル

保持時間 :10.786 min

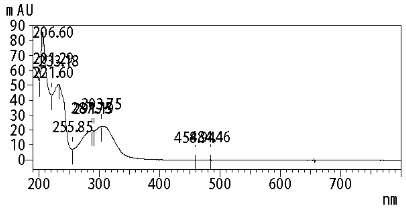

保持時間 :18.178 min

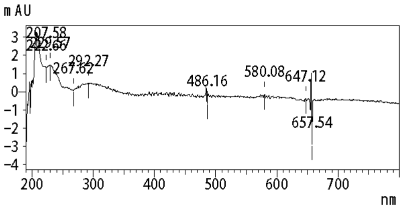

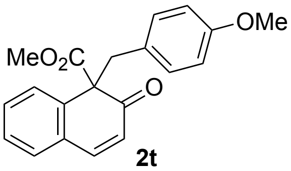

2t

=== Shimadzu LabSolutions ===

Sample :
ID :
Data Name :LY-6-102b-rac001.lcd
Method Name :C3-80-20-1ml-20min.lcm
Batch Nam :LY-6-102b.lcb
Vial :1-48 サンプルタイプ :Unknown
Inj. Volume :1 uL
Acquisition Date :2018/06/14 18:40:48 分析者 :System Administrator
Modified Date :2018/06/14 20:18:46 解析者 :System Administrator

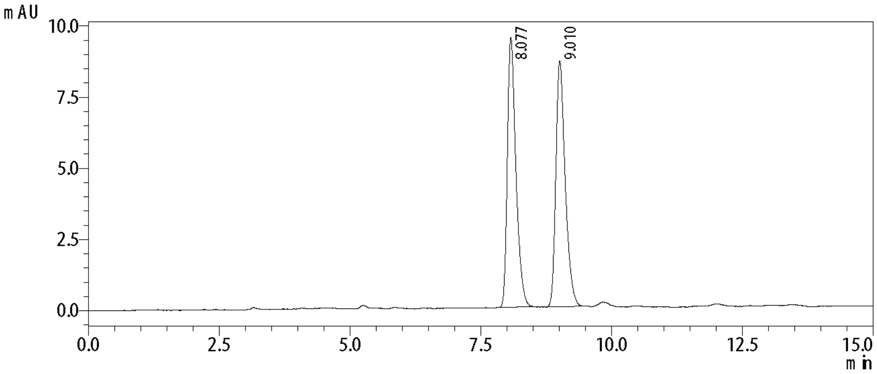

PDA Ch1 254nm

Peak No.	RT (min)	Area	Height	% Area
1	8.077	107981	9498	50.281
2	9.010	106773	8639	49.719
Total		214754	18138	100.000

UVスペクトル

保持時間 :8.077 min

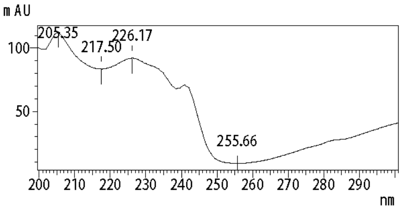

保持時間 :9.010 min

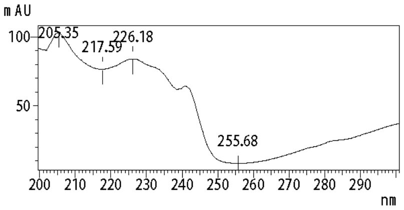

D:¥Data¥LY¥data¥LY-6-102b-rac001.lcd

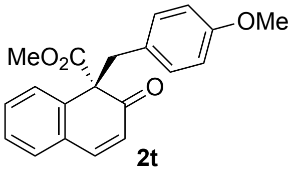

2t

=== Shimadzu LabSolutions ===

Sample :
ID :
Data Name :LY-6-102b001.lcd
Method Name :C3-80-20-1ml-20min.lcm
Batch Nam :LY-6-102b.lcb
Vial :1-49
Inj. Volume :1 uL
Acquisition Date :2018/06/14 19:01:13
Modified Date :2018/06/14 20:19:28
サンプルタイプ :Unknown
分析者 :System Administrator
解析者 :System Administrator

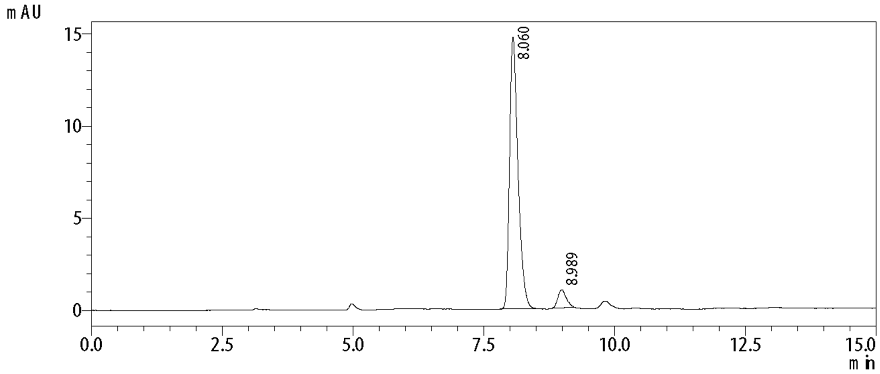

PDA Ch1 254nm

Peak No.	RT (min)	Area	Height	% Area
1	8.060	167113	14765	93.869
2	8.989	10915	974	6.131
Total		178028	15739	100.000

UVスペクトル

保持時間 :8.060 min
保持時間 :8.989 min

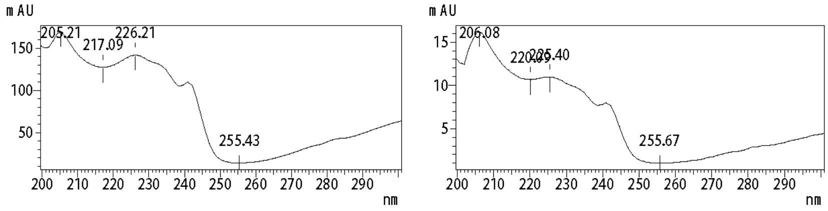

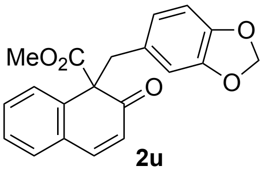

2u

=== Shimadzu LabSolutions ===

Sample :
ID :
Data Name :LY-6-102a-rac.lcd
Method Name :C3-80-20-1ml-20min.lcm
Batch Nam :LY-6-102.lcb
Vial :1-46 サンプルタイプ :Unknown
Inj. Volume :1 uL
Acquisition Date :2018/06/12 19:56:20 分析者 :System Administrator
Modified Date :2018/06/12 21:45:28 解析者 :System Administrator

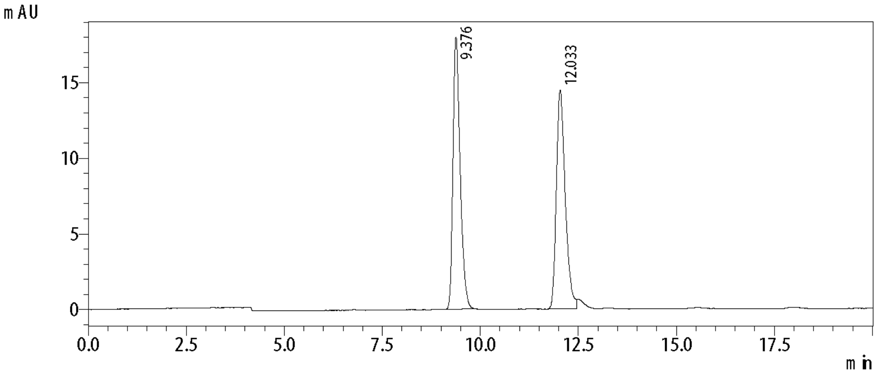

PDA Ch1 300nm

Peak No.	RT (min)	Area	Height	% Area
1	9.376	228066	17993	50.041
2	12.033	227696	14441	49.959
Total		455761	32434	100.000

UVスペクトル

保持時間 :9.376 min

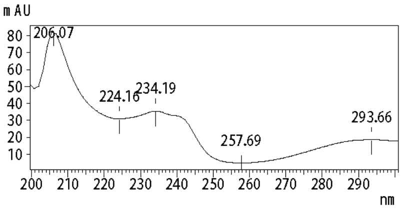

保持時間 :12.033 min

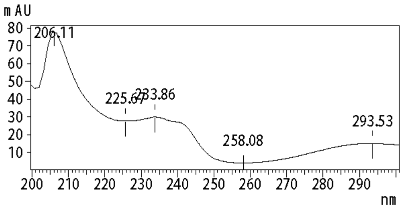

D:¥Data¥LY¥data¥LY-6-102a-rac.lcd

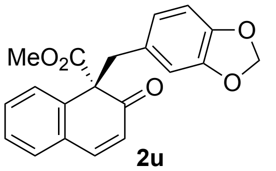

2u

7/7/2018 4:25:58 PM Page 1 / 1

=== Shimadzu LabSolutions ===

Sample	:		
ID	:		
Data Name	:LY-6-102a.lcd		
Method Name	:C3-80-20-1ml-20min.lcm		
Batch Nam	:LY-6-102.lcb		
Vial	:1-47	サンプルタイプ	:Unknown
Inj. Volume	:1 uL		
Acquisition Date	:2018/06/12 20:16:45	分析者	:System Administrator
Modified Date	:2018/06/12 21:46:55	解析者	:System Administrator

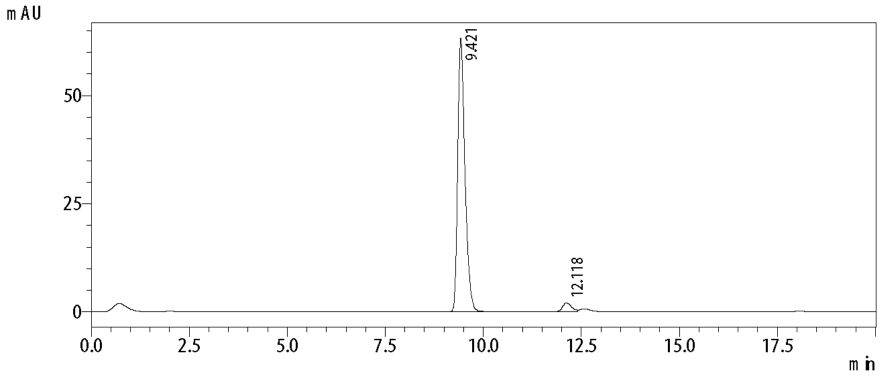

PDA Ch1 300nm

Peak No.	RT (min)	Area	Height	% Area
1	9.421	805445	63233	96.256
2	12.118	31327	2029	3.744
Total		836772	65263	100.000

UVスペクトル

保持時間 :9.421 min

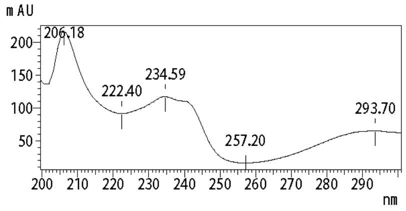

保持時間 :12.118 min

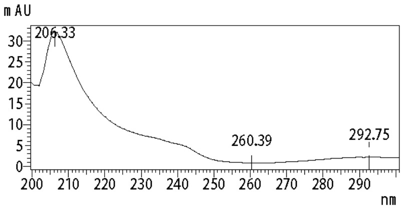

D:¥Data¥LY¥data¥LY-6-102a.lcd

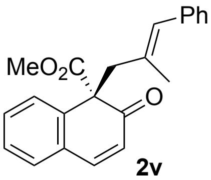

2v

=== Shimadzu LabSolutions ===

Sample	:		
ID	:		
Data Name	:LY-5-1c-rac001.lcd		
Method Name	:C2-20-80-1-20min.lcm		
Batch Nam	:LY-5-1c.lcb		
Vial	:1-18	サンプルタイプ	:Unknown
Inj. Volume	:1 uL		
Acquisition Date	:2017/11/13 19:38:05	分析者	:System Administrator
Modified Date	:2017/11/13 20:22:38	解析者	:System Administrator

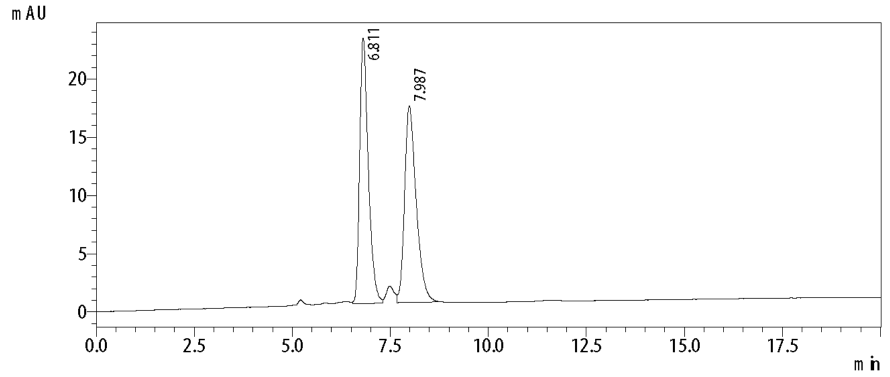

PDA Ch1 254nm

Peak No.	RT (min)	Area	Height	% Area
1	6.811	349496	22811	50.149
2	7.987	347423	16876	49.851
Total		696919	39686	100.000

UVスペクトル

保持時間 :6.811 min

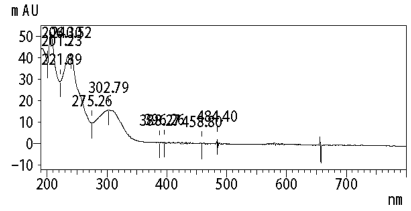

保持時間 :7.987 min

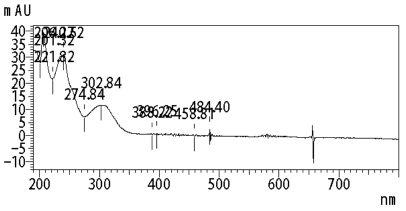

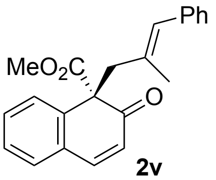

2v

3/15/2018 8:07:32 PM Page 1 / 1

=== Shimadzu LabSolutions ===

Sample :
ID :
Data Name :LY-5-1c001.lcd
Method Name :C2-20-80-1-20min.lcm
Batch Nam :LY-5-1c.lcb
Vial :1-19
Inj. Volume :1 uL
Acquisition Date :2017/11/13 19:58:36
Modified Date :2018/03/01 21:05:36

サンプルタイプ :Unknown
分析者 :System Administrator
解析者 :System Administrator

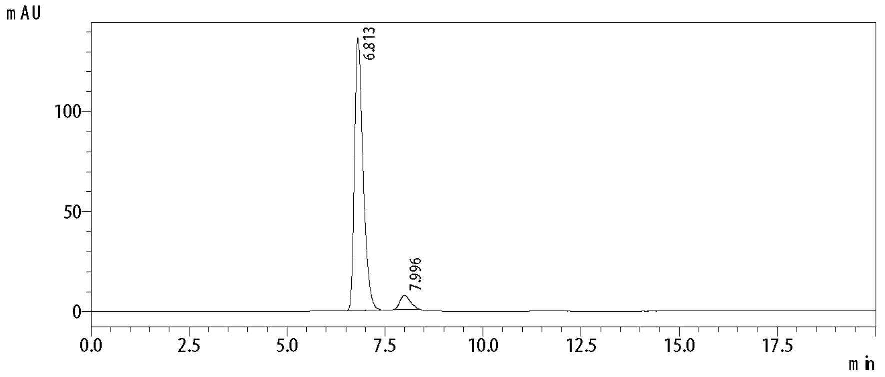

PDA Ch1 254nm

Peak No.	RT (min)	Area	Height	% Area
1	6.813	2077139	136531	94.102
2	7.996	130190	7069	5.898
Total		2207329	143600	100.000

UVスペクトル

保持時間 :6.813 min

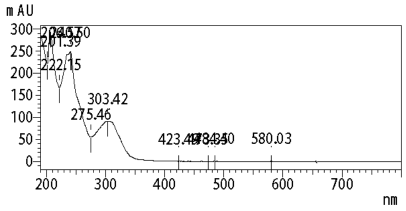

保持時間 :7.996 min

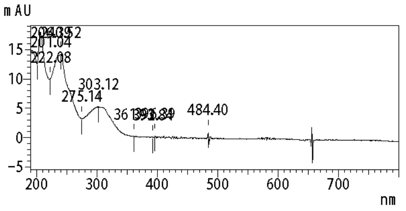

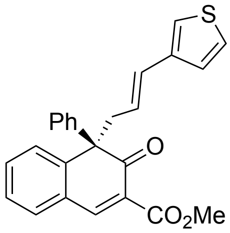

4a

11/12/2018 2:30:21 PM Page 1 / 1

=== Shimadzu LabSolutions ===

Sample	:		
ID	:		
Data Name	:LY-7-75-RAC.lcd		
Method Name	:C2-20-80-1-30min.lcm		
Batch Nam	:LY-7-75.lcb		
Vial	:1-61	サンプルタイプ	:Unknown
Inj. Volume	:1 uL		
Acquisition Date	:2018/10/26 18:36:38	分析者	:System Administrator
Modified Date	:2018/10/26 21:22:32	解析者	:System Administrator

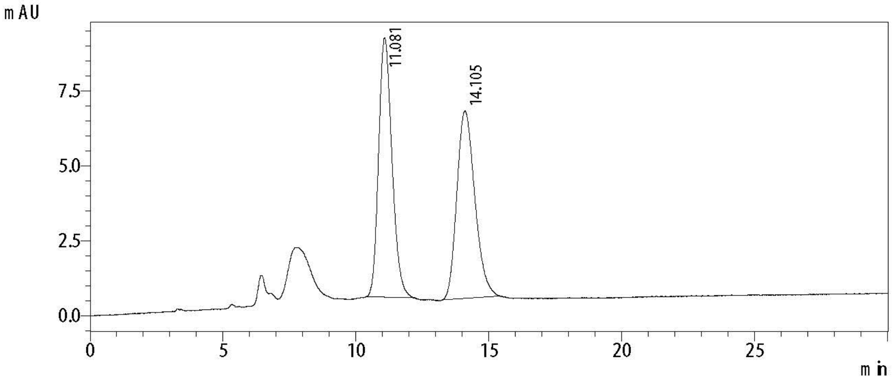

PDA Ch1 254nm

Peak No.	RT (min)	Area	Height	% Area
1	11.081	302759	8659	50.688
2	14.105	294539	6253	49.312
Total		597299	14912	100.000

UVスペクトル

保持時間 :11.081 min

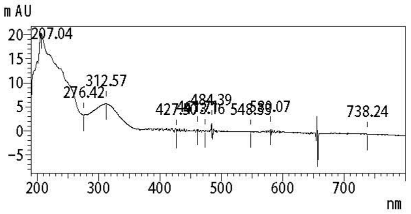

保持時間 :14.105 min

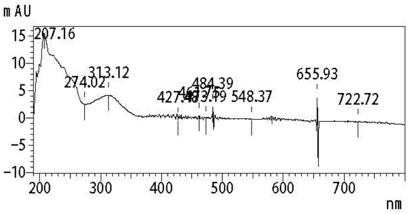

D:¥Data¥LY¥data¥LY-7-75-RAC.lcd

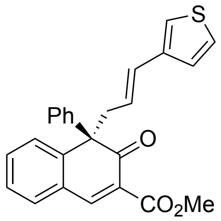

4a

=== Shimadzu LabSolutions ===

Sample	:		
ID	:		
Data Name	:LY-7-75-92EE.lcd		
Method Name	:C2-20-80-1-20min.lcm		
Batch Nam	:LY-7-75.lcb		
Vial	:-1	サンプルタイプ	:Unknown
Inj. Volume	:1 uL		
Acquisition Date	:2018/11/07 15:58:23	分析者	:System Administrator
Modified Date	:2018/11/07 16:20:04	解析者	:System Administrator

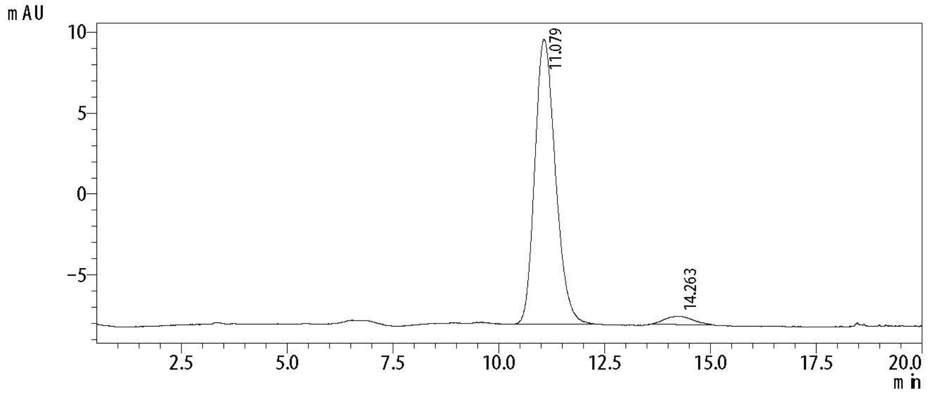

PDA Ch1 254nm

Peak No.	RT (min)	Area	Height	% Area
1	11.079	597031	17600	96.150
2	14.263	23903	525	3.850
Total		620934	18125	100.000

UVスペクトル

保持時間 :11.079 min

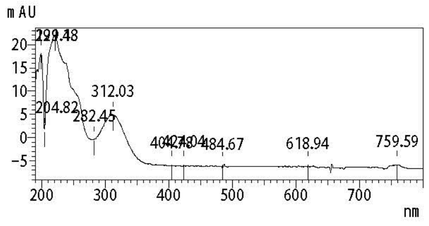

保持時間 :14.263 min

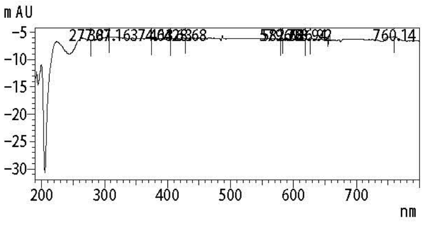

D:¥Data¥LY¥data¥LY-7-75-92EE.lcd

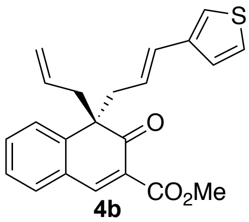

4b

=== Shimadzu LabSolutions ===

Sample	:		
ID	:		
Data Name	:LY-7-110-RAC-od.lcd		
Method Name	:C1-20-80-1-20min.lcm		
Batch Nam	:LY-7-110-RAC.lcb		
Vial	:1-61	サンプルタイプ	:Unknown
Inj. Volume	:1 uL		
Acquisition Date	:2018/11/08 10:28:54	分析者	:System Administrator
Modified Date	:2018/11/09 11:33:52	解析者	:System Administrator

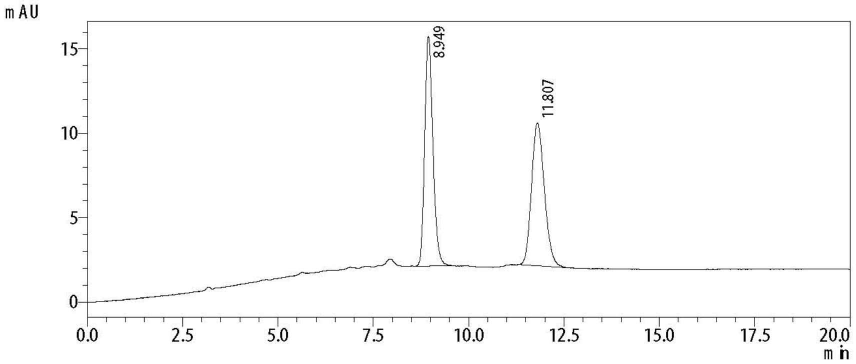

PDA Ch1 254nm

Peak No.	RT (min)	Area	Height	% Area
1	8.949	203401	13634	50.713
2	11.807	197679	8456	49.287
Total		401079	22090	100.000

UVスペクトル

保持時間 :8.949 min　　保持時間 :11.807 min

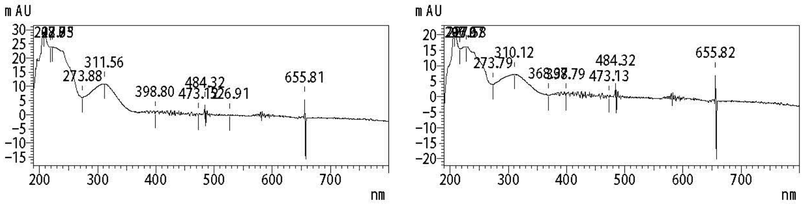

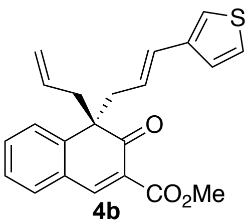

4b

=== Shimadzu LabSolutions ===

Sample	:		
ID	:		
Data Name	:LY-7-110.lcd		
Method Name	:C1-20-80-1-20min.lcm		
Batch Nam	:LY-7-110.lcb		
Vial	:1-61	サンプルタイプ	:Unknown
Inj. Volume	:1 uL		
Acquisition Date	:2018/11/08 22:33:36	分析者	:System Administrator
Modified Date	:2018/11/09 11:33:38	解析者	:System Administrator

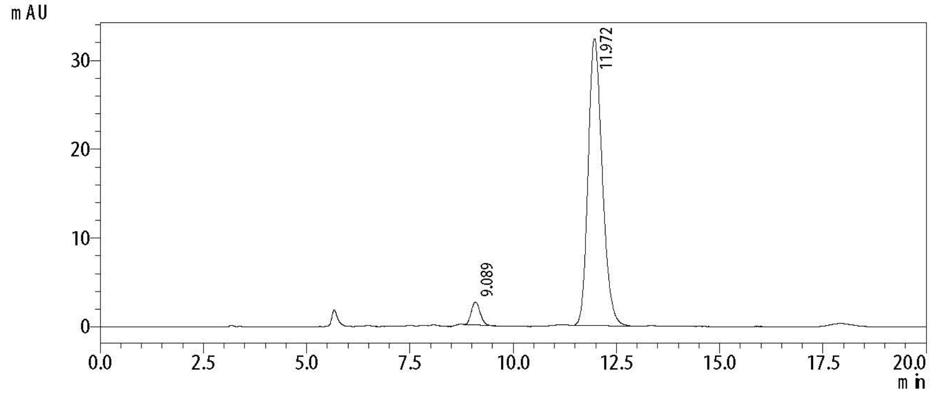

PDA Ch1 254nm

Peak No.	RT (min)	Area	Height	% Area
1	9.089	37835	2603	4.691
2	11.972	768730	32338	95.309
Total		806565	34941	100.000

UVスペクトル

保持時間 :9.089 min

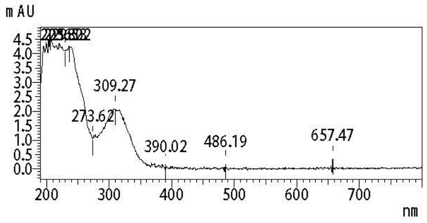

保持時間 :11.972 min

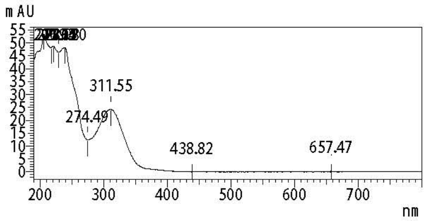

D:¥Data¥LY¥data¥LY-7-110.lcd

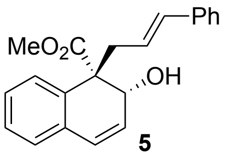

5

=== Shimadzu LabSolutions ===

Sample	:
ID	:
Data Name	:LY-5-110-rac.lcd
Method Name	:C3-80-20-1ml-20min.lcm
Batch Nam	:LY-5-110.lcb
Vial	:1-32
Inj. Volume	:1 uL
Acquisition Date	:2018/02/02 11:02:21
Modified Date	:2018/02/02 11:22:24

サンプルタイプ	:Unknown
分析者	:System Administrator
解析者	:System Administrator

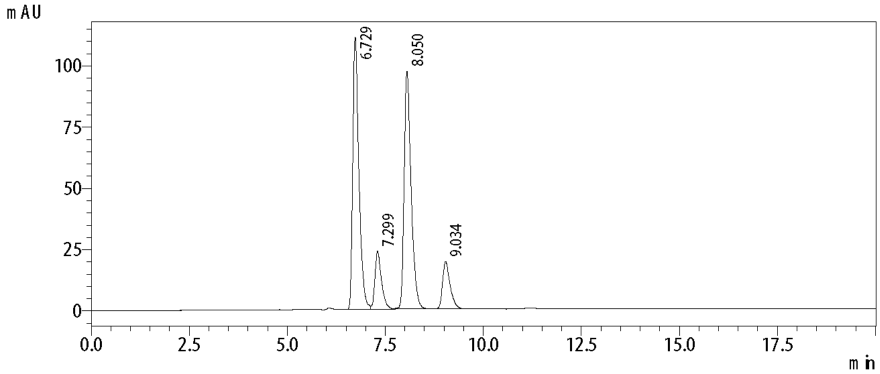

PDA Ch1 254nm

Peak No.	RT (min)	Area	Height	% Area
1	6.729	1211638	111252	41.099
2	7.299	276803	23681	9.389
3	8.050	1200750	97013	40.730
4	9.034	258889	19230	8.782
Total		2948081	251176	100.000

UVスペクトル

保持時間 :6.729 min

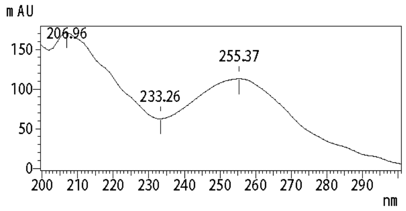

保持時間 :7.299 min

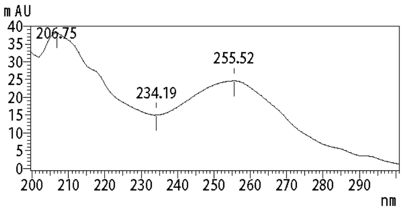

D:¥Data¥LY¥data¥LY-5-110-rac.lcd

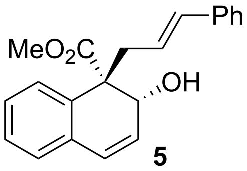

5

=== Shimadzu LabSolutions ===

Sample	:
ID	:
Data Name	:LY-5-110.lcd
Method Name	:C3-80-20-1ml-20min.lcm
Batch Nam	:LY-5-110.lcb
Vial	:1-33
Inj. Volume	:1 uL
Acquisition Date	:2018/02/02 11:22:47
Modified Date	:2018/02/02 11:42:50

サンプルタイプ	:Unknown
分析者	:System Administrator
解析者	:System Administrator

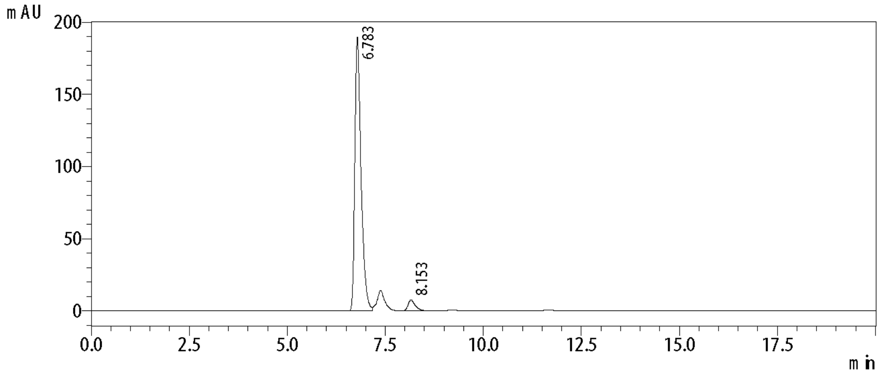

PDA Ch1 254nm

Peak No.	RT (min)	Area	Height	% Area
1	6.783	2081513	189743	95.803
2	8.153	91177	7321	4.197
Total		2172690	197064	100.000

UVスペクトル

保持時間 :6.783 min

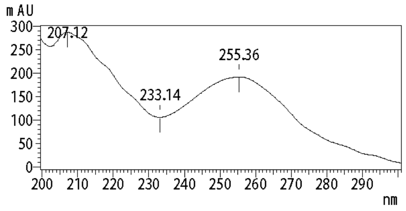

保持時間 :8.153 min

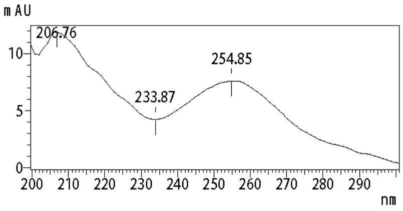

D:¥Data¥LY¥data¥LY-5-110.lcd
